# A new haplotype-resolved turkey genome to enable turkey genetics and genomics research

**DOI:** 10.1093/gigascience/giad051

**Published:** 2023-07-21

**Authors:** Carolina P Barros, Martijn F L Derks, Jeff Mohr, Benjamin J Wood, Richard P M A Crooijmans, Hendrik-Jan Megens, Marco C A M Bink, Martien A M Groenen

**Affiliations:** Wageningen University and Research, P.O. Box 338, 6700 AH, Wageningen, The Netherlands; Wageningen University and Research, P.O. Box 338, 6700 AH, Wageningen, The Netherlands; Hybrid Turkeys, 650 Riverbend Drive Suite C, Kitchener, ON N2K 3S2, Canada; Hybrid Turkeys, 650 Riverbend Drive Suite C, Kitchener, ON N2K 3S2, Canada; School of Veterinary Science, University of Queensland, Gatton, QLD 4343, Australia; Wageningen University and Research, P.O. Box 338, 6700 AH, Wageningen, The Netherlands; Wageningen University and Research, P.O. Box 338, 6700 AH, Wageningen, The Netherlands; Hendrix Genetics Research, Technology & Services, Boxmeer, AC 5830, The Netherlands; Wageningen University and Research, P.O. Box 338, 6700 AH, Wageningen, The Netherlands

**Keywords:** genome assembly, turkey genomics, trio-binning, animal breeding

## Abstract

**Background:**

The domesticated turkey (*Meleagris gallopavo*) is a species of significant agricultural importance and is the second largest contributor, behind broiler chickens, to world poultry meat production. The previous genome is of draft quality and partly based on the chicken (*Gallus gallus*) genome. A high-quality reference genome of *M. gallopavo* is essential for turkey genomics and genetics research and the breeding industry.

**Results:**

By adopting the trio-binning approach, we were able to assemble a high-quality chromosome-level F1 assembly and 2 parental haplotype assemblies, leveraging long-read technologies and genome-wide chromatin interaction data (Hi-C). From a total of 40 chromosomes (2n = 80), we captured 35 chromosomes in a single scaffold, showing much improved genome completeness and continuity compared to the old assembly build. The 3 assemblies are of higher quality than the previous draft quality assembly and comparable to the chicken assemblies (GRCg7) shown by the largest contig N50 (26.6 Mb) and comparable BUSCO gene set completeness scores (96–97%). Comparative analyses confirm a previously identified large inversion of around 19 Mbp on the Z chromosome not found in other Galliformes. Structural variation between the parent haplotypes was identified, which poses potential new target genes for breeding.

**Conclusions:**

We contribute a new high-quality turkey genome at the chromosome level, benefiting turkey genetics and other avian genomics research as well as the turkey breeding industry.

## Introduction

The domesticated turkey (*Meleagris gallopavo*, NCBI:txid9103) is an important agricultural species and the second largest contributor to world poultry production [1]. The turkey is a member of the Phasianidae family within the order Galliformes. Turkeys and chickens diverged about 25–40 million years ago [2]. Despite the relatively long divergence time, the genome synteny and karyotype of both are highly conserved [3]. The turkey has 2n = 80 compared to the chicken with 2n = 78. The turkey karyotype consists of 7 macrochromosomes (>50 Mb), 4 intermediate chromosomes (>20 Mb, <40 Mb), and the rest being microchromosomes (<20 Mb). The turkey karyotype is very similar to the chicken, except that chicken chromosome 2 is homologous to 2 turkey chromosomes (chromosomes 3 and 6), and chicken chromosome 4 is homologous to turkey chromosomes 4 and 9 [4]. Zhang et al. [5] identified a large inversion on the Turkey lineage compared to chicken. In addition, a high degree of synteny has also been observed between the chicken and turkey genomes [6].

The first turkey genome assembly (UMD2), published in 2010 [6], was among the first to be done almost exclusively based on second-generation sequencing data and by current standards would be considered of draft quality given the low contig N50 (27.1 kb) and lack of long-read sequences [7]. The authors produced a chromosome-level assembly and assembled 30 autosomal and 2 sex chromosomes. The assembly included linkage data based on a low-density genetic map, and the placement of scaffolds to chromosomes relied considerably on conserved synteny assumptions with the better assembled chicken (*Gallus gallus*) genome. However, that version of the chicken genome had many microchromosomes missing altogether or only partially characterized. Avian microchromosomes have proved to be difficult to assemble even today [7]. Reliance on an incomplete chicken genome and the general difficulty in assembling the avian microchromosomes resulted in a poor representation of microchromosomes in that first UMD2 turkey genome. An updated version of the turkey genome (Turkey_5.1; GCA_000,146,605.4) has been available since 2019, although it still shows low gene completeness and an incomplete set of microchromosomes.

The problems in characterizing microchromosomes are partly due to sequence characteristics (i.e., high GC and repeat content in microchromosomes) and partly due to their extremely small size and lack of genetic linkage group markers to differentiate the microchromosomes from other chromosomes [7]. Hence, ongoing efforts in producing high-quality assemblies of the microchromosomes in many avian genomes have been unsuccessful due to abovementioned causes. However, a novel chicken reference genome containing all autosomes and both sex chromosomes was published, with all gaps closed except for the W [8].

High-quality genome sequences are an essential resource for research and applications in the life sciences. In domestic animal breeding, genome-wide marker panels are routinely used to support genomic selection, and this significantly accelerates genetic progress [9]. An improved genome sequence facilitates ongoing genomic breeding programs. Furthermore, an improved genome assembly will greatly enhance functional interpretation of genomic variation in those breeding populations. For instance, improved annotation of (non)coding genes benefits the functional interpretation of genome-wide association studies (GWASs) and aids in identifying targets for gene editing [10].

Currently, more species in the Galliformes have high-quality long-read–based assemblies, including the chicken [8], Japanese quail [11], Gunnison sage-grouse [12], and the helmeted guineafowl [13], allowing for comparative studies within the Galliformes and an in-depth comparison between the 2 most important avian agricultural species (chicken and turkey).

Third-generation sequencing techniques have made it possible to produce high-quality chromosome-based assemblies. The chicken individual broiler (GRCg7b) and layer (GRCg7w) assemblies and, more recently, the complete chicken genome assembly [8] have been produced from long-read sequencing techniques. These new chicken assemblies show superior metrics of quality and completeness to previous genome assemblies. In this study, we use a relatively new technique, the trio-binning approach, to construct high-quality haplotype-resolved turkey assemblies [14]. A similar approach was also applied to create the recent chicken genome assemblies. In the trio-binning approach, short reads from each parent are used to resolve the F1 long reads into groups of long reads belonging to each parent. Each haplotype is then assembled independently, resulting in 3 high-quality genome assemblies, one from both parental haplotypes and one F1 assembly (the primary assembly). This approach is especially powerful to assess structural variation between the parental haplotypes and works well with high heterozygosity rates as this aids in the resolution of the parent haplotypes in the F1 assembly.

In this study, our aims were to use the trio-binning approach to produce a chromosome-level turkey assembly (F1) and 2 parental haplotype assemblies. We further aim to compare the 2 parental haplotypes to identify structural differences. A good reference genome is essential for many research and commercial applications. In this study, we highlight how our new turkey genome can benefit both research and the breeding industry.

## Results

### Data and assembly of mgal_WUR_HG_1.0

We used a trio-sequencing [14] approach to assemble the diploid genome of a male turkey. The F1 was sequenced with long reads, while the parents were sequenced with short reads to employ the trio-binning. The 2 parental animals derive from 2 distinct commercial lines from the breeding company Hybrid Turkeys, a Hendrix Genetics company. The F1 animal was sequenced with a depth of 270× using PacBio single-molecule real-time (SMRT) sequencing technology. Approximately 12.25 million subreads were produced with a mean length of 22.5 kb and N50 read length of 32.5 kb. Reads were assembled using wtdgb2 assembler [15], resulting in an initial assembly comprising 315 contigs with an N50 of 26.68 Mb. The assembly was further scaffolded using Hi-C with HiRise [16]. Additional scaffolding was performed using SALSA (with Hi-C) [17] and Redundans [18]. The scaffolded assembly was subsequently polished with short reads (3 rounds) to produce a final chromosome-level assembly consisting of 151 scaffolds and 232 contigs with a scaffold N50 of 70 Mbp and contig N50 of 26.55 Mbp (Table 1). This captures the majority of the chromosomes in a single scaffold and chromosome arms in a single contig (Supplementary File 1: Table S1). The Hi-C contact map can be found in Supplementary File 1: Fig. S1.

**Table 1:** Assembly statistics: Summary statistics for the new Mgal_WU_HG_1.0 and parental assemblies and comparison with previous turkey assembly (Turkey_5.1) and recent broiler assembly (GRCg7b)

	Mgal_WU_HG_1.0	Turkey_5.1	GRCg7b	Parent 1	Parent 2
Total sequence length (bp)	1,001,818,376	1,115,474,681	1,053,332,251	1,051,251,094	1,085,657,715
Length ungapped (bp)	1,001,806,830	1,080,180,254	1,049,948,333	1,050,601,018	1,085,166,758
No. of scaffolds	151	187,695	214	415	489
No. of unplaced scaffolds	115	187,662	172	379	453
No. of chromosomes	36	33	42	36	36
Scaffold N50 (bp)	70,339,173	3,898,092	90,861,225	71,046,337	71,481,950
Scaffold L50	5	80	4	4	4
No. of contigs	232	250,220	677	738	675
Contig N50 (bp)	26,554,504	27,076	18,834,961	9,174,806	19,817,032
Contig L50	12	11,318	18	34	13

#### Telomeres and centromeres

Telomeres and centromeres are generally enriched for simple repeats. Telomeric repeats (TTAGGG) were identified on the tail(s) of 18 chromosomes, supporting further completeness of the genome assembly (Supplementary File 1: Fig. S2). A 41-bp TM repeat was previously identified in turkey to be abundant in centromeric and (sub)telomeric regions, especially on the microchromosomes [19]. Zhang et al. [5] showed that there is a clear trend in turkey toward telocentric chromosomes, meaning that the centromere is located very close to the end of the chromosome and that the p-arms would not, or barely, be visible. The only clearly metacentric chromosomes in turkey are chromosome 1 and the sex chromosomes. We predicted that the centromere of chromosome 1 is located at 74.12–74.16 Mb enriched for TTAGGG and TM repeats (Supplementary File 1: Fig. S2). Overall, we observed an enrichment of TM repeat clusters at the tails of chromosomes and in the microchromosomes. Furthermore, we identified clusters of TM repeats on one tail of macrochromosomes 2, 3, and 4, which are likely telocentric with very short p-arms [5]. The enrichment of TM repeats at one chromosome tail indicates that the centromeric regions of these chromosomes likely comprise of clusters of the 41-bp TM repeat, while the other tail comprises telomeric repeats (Supplementary File 1: Fig. S2). In addition, we identified clusters of TM repeats at the tail(s) of intermediate chromosomes 8, 9, and 10 and microchromosomes 12, 20, 24, 25, 26, 29, 30, and 31 (Supplementary File 1: Fig. S2). The intermediate chromosomes 7 to 14 are all predicted to be telocentric [5]. We found that chromosomes 8, 9, 10, and 12 show enrichment of TM repeats on one tail of the chromosome, likely indicating centromeric regions at the chromosome ends. The majority of the microchromosomes have at least telomeric repeats at one tail of the chromosome and several exhibit TM repeat clusters at the other end, supporting the likely telocentric structure of these microchromosomes.

#### Haplotype assemblies

As part of the trio-binning approach, both parental haplotypes were assembled with TrioCanu [14]. We were able to map 110× of the PacBio reads to parent 1 and 137× of the PacBio reads to parent 2, resulting in 2 parental haplotype assemblies with contig N50 of 9,174,806 bp and 19,855,975 bp for parents 1 and 2, respectively. We performed further scaffolding using LRscaf [20] and anchored the assemblies to the F1 assembly using RagTag [21]. The consensus quality value (QV) values indicate high quality and completness of the assemblies evaluated by Mercury [22] (Supplementary File 1: Table S2). The final statistics of the assemblies are shown in Table 1.

#### Assembly accuracy and completeness

The completeness and accuracy of the assemblies were assessed using BUSCO [23] and whole-genome alignments. All 3 assemblies contained over 96% of the expected avian and vertebrate gene sets, comparable to the GRCg6a and GRCg7b chicken genomes and covering 5.4% more gene space compared to the previous turkey genome assembly (Turkey_5.1), as shown in Table 2.

**Table 2:** Assembly completeness measured in BUSCO scores: Percentage of aligned genes for the vertebrae (*n* = 3,354) and avian (*n* = 8,338) gene set in the turkey and chicken assemblies

	Mgal_WU_HG_1.0		Turkey_5.1		GRCg7b		Parent 1		Parent 2	
	*Avian*	*Vertebrate*	*Avian*	*Vertebrate*	*Avian*	*Vertebrate*	*Avian*	*Vertebrate*	*Avian*	*Vertebrate*
Complete	96.7	96.4	91.3	88.4	97.0	96.5	96.6	96.0	96.8	96.4
Complete and single copy	96.4	95.9	91.1	87.9	96.7	95.7	94.8	93.9	94.1	93.2
Complete and duplicated	0.3	0.5	0.2	0.5	0.3	0.8	1.8	2.1	2.7	3.2
Fragmented	0.9	1.0	4.1	5.8	0.9	1.2	0.9	1.1	0.9	1.0
Missing	2.4	2.6	4.6	5.8	2.1	2.3	2.5	2.9	2.3	2.6

**Figure 1:**
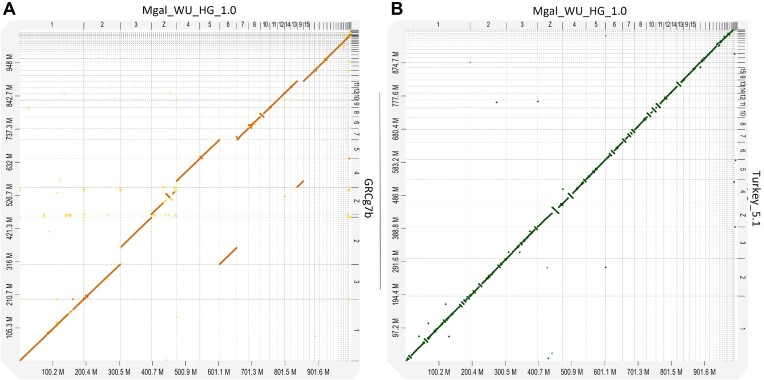
Genome-wide alignment plots. (A) Mgal_WU_HG_1.0 aligned with GRCg7b. Alignment shows high structural coherence between both genomes. (B) Mgal_WU_HG_1.0 aligned with the old turkey genome build Turkey_5.1. Alignment shows multiple contigs that were placed in the wrong orientation in the previous Turkey_5.1 build.

The turkey genome is highly congruent with the chicken genome (Fig. 1A), indicating a high degree of conserved synteny. The main exception was a large ∼19-Mbp inversion on the Z chromosome (approximate coordinates ∼44,493,000–63,950,000 bp). This inversion was also not present in the previous turkey build, Turkey_5.1, as seen in the alignment (Fig. 1B). The alignment further shows that in the Turkey_5.1 assembly, many contigs were placed in the wrong orientation (resulting in a “zigzag” alignment pattern).

### Repeat and gene annotation

#### Repeat content

We annotated the repeats using a custom repeat library built using RepeatModeler [24]. Repeats were found to cover 10.45% of the genome. The most common were long interspersed nuclear elements (LINEs), covering 6.35% of the genome. Furthermore, 0.76% of bases were DNA transposons, 0.53% long terminal repeats (LTRs), and 1.58% low-complexity and simple repeats. The remaining 1.23% of the repeats remained unclassified. A complete overview of the repeats per chromosome is listed in Supplementary File 2.

#### Gene annotation

**Figure 2:**
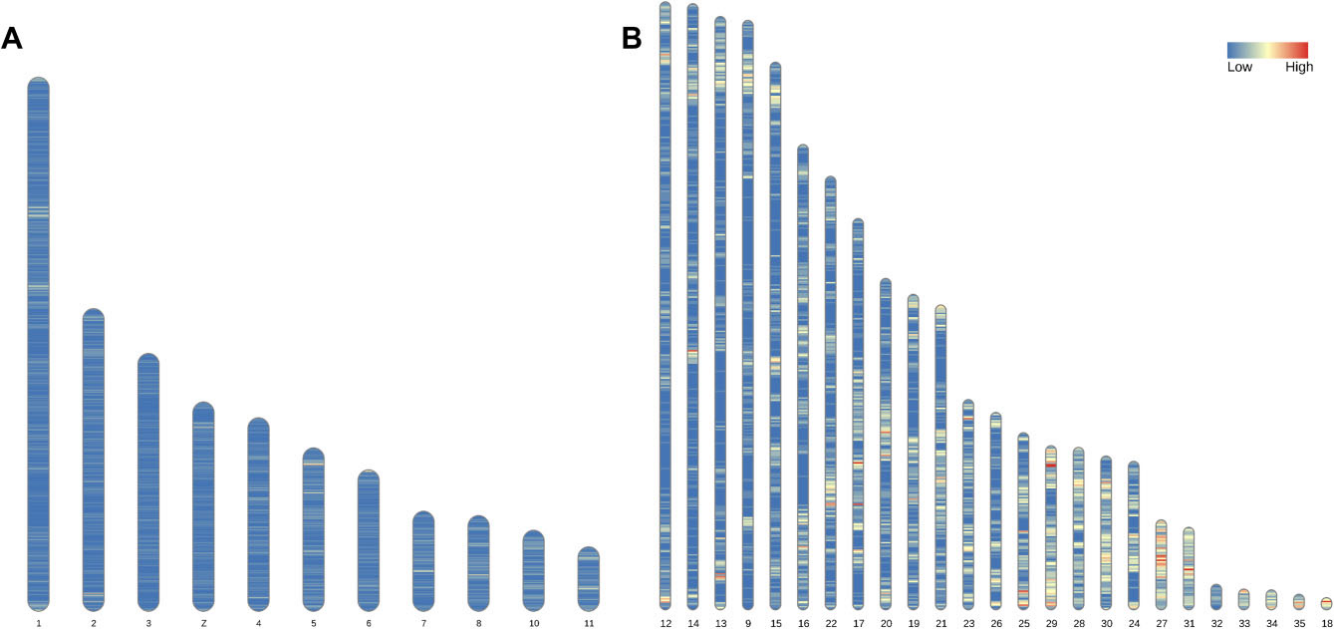
Ideogram showing gene density. (A) Macro (1–6, Z) and intermediate chromosomes (7, 8, 10, 11). (B) Microchromosomes (9, 12–35) in the Mgal_WU_HG_1.0 genome.

The Ensembl annotation pipeline was used to annotate Mgal_WU_HG_1.0 [25]. The present annotation includes fewer annotated genes compared to Turkey_5.1 and the chicken annotations but does include more noncoding genes, as shown in Table 3. As expected, microchromosomes show higher gene density compared to macrochromosomes and intermediate chromosomes (*P* < 0.00001, Fig. 2). The density generally increases with decreasing microchromosome size.

**Table 3:** Annotation statistics for the turkey (Mgal_WU_HG_1.0, Turkey_5.1) and chicken (GRCg6a, GRCg7b) genomes: BUSCO scores show percentage of aligned proteins for the avian (*n* = 8,338) and vertebrate (*n* = 3,354) protein set in the turkey and chicken assemblies

Annotation	Mgal_WU_HG_1.0	Turkey_5.1	GRCg6a	GRCg7b
Coding genes	16,127	16,226	16,878	17,007
Noncoding genes	7,736	1,585	7,166	13,040
Small noncoding genes	504	543	1,525	1,089
Long noncoding genes	7,228	1,038	5,506	11,946
Miscellaneous noncoding genes	4	4	135	5
Pseudogenes	45	159	312	61
Gene transcripts	53,441	30,708	39,288	72,689
**Completeness BUSCO (avian/vertebrate)**				
% Complete	97.9/97.0	87.5/80.8	95.1/93.8	98.3/97.0
% Fragmented	0.6/1.1	5.2/10.3	2.0/2.9	0.5/1.0
% Missing	1.5/1.9	7.3/8.9	2.9/3.3	1.2/2.0

We identified chicken and Turkey_5.1 homologs of the Mgal_WU_HG_1.0 genes (Supplementary File 1: Table S3). Most of the protein-coding genes have a 1:1 ortholog in the Turkey_5.1 (82.4%) or in the GRCg6a (86.3%) genome assemblies. The higher number of genes orthologous to the most recent chicken assemblies supports our assertion of a significant improvement of assembly and annotation quality compared to Turkey_5.1.

#### Gene family analysis

OrthoFinder [26] was used to infer orthogroups from the following set of bird species—turkey, chicken, Japanese quail (*Coturnix japonica*) [11], helmeted guineafowl (*Numida meleagris*) [13], and zebra finch (*Taeniopygia guttata*) [27]. From the 16,127 protein-coding genes in the Mgal_WU_HG_1.0 gene set, 98% were found to be in an orthogroup. This was the highest percentage of any of the species tested (Table 4). Of the 15,417 orthogroups found, 91% include Mgal_WU_HG_1.0 genes. There are also 10 orthogroups that contain only Mgal_WU_HG_1.0 genes, of which 2 have homologs in the nr database (*MANBAL* and *POL3*) (Supplementary File 1: Table S4).

**Table 4:** Number of orthogroups found and proportion of genes assigned to each orthogroup per species. Species included turkey (Mgal_WU_HG_1.0, Turkey_5.1), chicken (GRCg6a, GRCg7b), Japanese quail (Coturnix_japonica_2.0), helmeted guineafowl (NumMel1.0), and zebra finch (bTaeGut1_v1.p)

Species assembly	Mgal_WU_HG_1.0	Turkey_5.1	GRCg6a	GRCg7b	Coturnix_japonica_2.0	NumMel1.0	bTaeGut1_v1.p
No genes	16,127	16,226	16,878	17,007	15,732	15,661	16,619
No genes in orthogroups	15,843	15,365	16,359	16,583	15,342	15,306	15,971
No unassigned genes	284	861	519	424	390	355	648
Genes in orthogroups (%)	98.2	94.7	96.9	97.5	97.5	97.7	96.1
Unassigned genes (%)	1.8	5.3	3.1	2.5	2.5	2.3	3.9
No orthogroups containing species	14,033	13,350	13,800	14,156	13,801	13,695	13,390
Orthogroups containing species (%)	91	86.6	89.5	91.8	89.5	88.8	86.9
No species-specific orthogroups	10	63	23	24	4	7	110
No genes in species-specific orthogroups	50	178	120	95	9	67	428
Genes in species-specific orthogroups (%)	0.3	1.1	0.7	0.6	0.1	0.4	2.6

#### Contractions and expansions in orthologous groups

While most orthogroups studied showed no change in the copy number of protein-coding genes, 71 groups showed expansions or contractions of gene families predicted using CAFE5 software [28] (61 expansions, 10 contractions) (Supplementary File 3). Expanded orthogroups contained proteins involved in important processes in bird development and growth, including gene families involved in cytoskeleton (proteins for feather keratin) (OG0000026, OG0000030), reproduction (involved in spermatogenesis/spermiogenesis) (OG0000005), response to stress (OG0000111), and immunity (OG0000001). Orthogroup OG0000005 shows an expansion of the turkey PHD finger protein 7 (PHF7) gene, which has been shown to be a highly duplicated gene family in the chicken genome [29]. The contracted gene families include 1 immunoglobulin (OG0000001), a homeobox B8 (OG0000526) gene family, and an olfactory receptor gene family (OG0000407).

### Structural variation between parental haplotypes

#### Structural variation

The F1 and the paternal haplotypes are completely colinear (Supplementary File 1: Fig. S3). There are no large structural differences (>1 Mbps) between the 2 parental haplotypes, except for a 1.47-Mbp inversion on chromosome 1 (74.28–75.74 Mb, Supplementary File 4) comprising 25 protein-coding genes and 15 long noncoding RNA (lncRNA) genes. This inversion is in the centromeric region of chromosome 1, shown by an excess of telomeric and TM repeats between 74.12 and 74.16 Mb. Table 5 shows an overview of the number and cumulative length of each type of structural variation.

**Table 5:** Structural variation between the 2 parental haplotype assemblies. The parent 1 assembly was used as reference and the parent 2 assembly used as the query. Copygain: Copy gain in the query genome, copyloss: copy loss in the query genome

Variation type	Count	Length parent 1	Length parent 2
Syntenic regions	85	990,480,776	989,217,672
Inversions	19	1,728,932	1,525,862
Translocations	68	895,801	867,550
Duplications	397	870,354	3,179,922
Copy gains	40	—	305,148
Copy losses	58	1,268,056	—

Copy gains are regions that have extra copies in the parent 2 haplotype, while copy losses show regions with fewer copies in parent 2 (and thereby higher copies in parent 1). The distribution of copy gains and copy losses is in Supplementary File 1: Fig. S4. In total, 231 large structural variations (>10 kb) have been identified between the 2 parental haplotypes (Supplementary File 5). From these, 81 affect the coding sequence of protein-coding genes, of which 40 have a 1:1 ortholog in chicken. Interestingly, an inversion affecting the coding sequence of the *BLB2* gene, which is duplicated within the MHC-B region in chicken and plays a crucial role in disease resistance or susceptibility [30], was found in parent 2 compared to parent 1 (Supplementary File 1: Fig. S5). We further identified duplications in the parent 2 haplotype comprising the *TRIM36, GRIA2*, and *MAN2B2* genes. Specifically, the parent 2 haplotype exhibits a 20-kb duplication of the 3′ end of *MAN2B2* (Supplementary File 1: Fig. S6), a gene that in pigs is associated with ovulation rate [31]. In addition, a 34-Kbp duplication affecting the *GEMIN8* gene in parent 1 was identified (Supplementary File 1: Fig. S7). The *GEMIN8* gene product is part of the survival motor neuron (SMN) complex. Moreover, a 53-Kbp duplication was found affecting the 3′ end of the *RIMKLB* gene (Supplementary File 1: Fig. S8), resulting in a copy number of 3 in parent 1 but a copy number of >5 in parent 2. In addition, a 100-kb translocation that comprises the *RALYL* gene was identified. The translocated region is found at around 68.2 Mbp on chromosome 5 in parent 1, while it is found at a position around 90.1 Mbp on the same chromosome in parent 2. Finally, an inversion on chromosome 30 of length 187 kb comprises 2 protein-coding genes and 1 lncRNA.

A full overview of structural variation between the parental haplotypes is provided in Supplementary File 5.

#### Loss-of-function variation

The most common effect of selection is to alter gene expression, leading to phenotypic changes. However, a small proportion of phenotypic variation is due to impaired gene functioning [32]. We assessed the presence of loss-of-function variation (LoF), specifically stop-gained variants affecting genes in either of the 2 parental haplotypes (Supplementary File 6). In total, 138 stop-gained variants affecting 92 genes between the parent 1 and parent 2 haplotypes were identified. Genes carrying LoF mutations that are especially noteworthy include the *RYR2* gene, which is affected by 4 LoF variants in parent 2, likely leading to an impaired RYR2 protein. Mutations in the *RYR2* gene are associated with stress in broiler chickens [33]. A second gene worth highlighting is *LRRC41*, which, in the parent 2 haplotype, contains a stop-gained variant. Knockouts of this gene lead to increased lean body mass in mice, and hence this gene poses an interesting candidate for selection for body weight in turkey [34].

### Mapping of single-nucleotide polymorphism chip markers

Single-nucleotide polymorphism (SNP) chips are useful to study variation between individuals and are widely applied in genomic selection. We mapped SNP chip markers from a 65,000 SNP array (64,800 SNPs; Illumina, Inc.) to Mgal_WU_HG_1.0 (Supplementary File 1: Table S5) using a custom SNP mapping pipeline (see Methods). We mapped 64,536 (99.4%) of the markers to Mgal_WU_HG_1.0. From these, 1,532 markers that were located on unplaced contigs in Turkey_5.1 were mapped to specific chromosomes in Mgal_WU_HG_1.0, and 415 markers were placed on the new chromosomes 31–35, indicating a higher completeness. More specifically, we were able to place a significant number of new markers, especially on chromosomes 1 (412), 27 (120), 31 (192), and Z (594).

### Distinct genomic landscapes of turkey micro- and macrochromosomes

Avian genomes are known to vary greatly in genomic features, especially between the micro- and macrochromosomes [35]. We evaluated the genomic landscape of the turkey chromosomes in terms of repeat content, gene density, and gene expression between macrochromosomes (>40 Mbp), intermediate chromosomes(>40 Mbp, <20 Mbp), and microchromosomes (<20 Mbp). We found that the repeat content of each repeat class in macro-, micro-, and intermediate chromosomes varied highly along the chromosomes (Supplementary File 1: Figs. S9–S16, Supplementary File 2). Macrochromosomes are enriched for DNA transposons (*P* < 0.01) and LINE elements (*P* = 0.0281) compared to the intermediate and microchromosomes (Supplementary File 1: Figs. S10–S11). In addition, LINE CR1 elements are especially enriched at the tails of macrochromosomes. Microchromosomes are enriched for low-complexity (*P* < 0.01, Supplementary File 1: Fig. S11), simple (*P* < 0.01, Supplementary File 1: Fig. S11), and unknown (*P* = 0.062, Supplementary File 1: Fig. S16) repeats compared to intermediate and macrochromosomes, the latter especially at the tails of the chromosomes.

**Figure 3:**
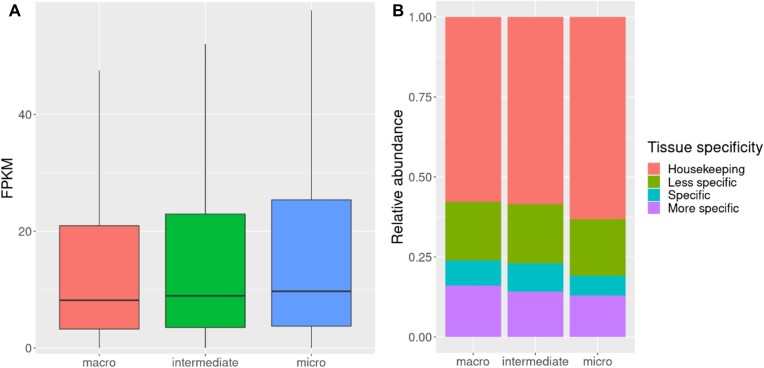
(A) Overview of gene expression in macrochromosomes, intermediate chromosomes, and microchromosomes. (B) Relative abundance of tissue-specific genes in each chromosome class. Microchromosomes show a higher relative abundance of housekeeping genes when compared with macrochromosomes and intermediate chromosomes. Number of tissues tested: 16. Housekeeping genes: expressed in at least 13 tissues; less specific genes: expressed in at least 5 tissues and fewer than 13 tissues; specific: expressed in 2 to 5 tissues; more specific: expressed in 1 or 2 tissues.

In order to assess whether there was a distinction between the type of genes (e.g., tissue specific or housekeeping) in chromosome types, we analyzed RNA sequencing (RNA-seq) datasets from 16 tissues (mapping rates in Supplementary File 1: Table S6). Similar to findings in the chicken genome by Huang et al. [8], microchromosomes showed on average higher gene expression than macro- and intermediate chromosomes (Fig. 3A), as well as having a higher relative abundance of housekeeping genes, defined here as genes expressed in at least 13 of the 16 studied tissues included in this study (Fig. 3B).

### Conserved synteny within the Galliformes clade

**Figure 4:**
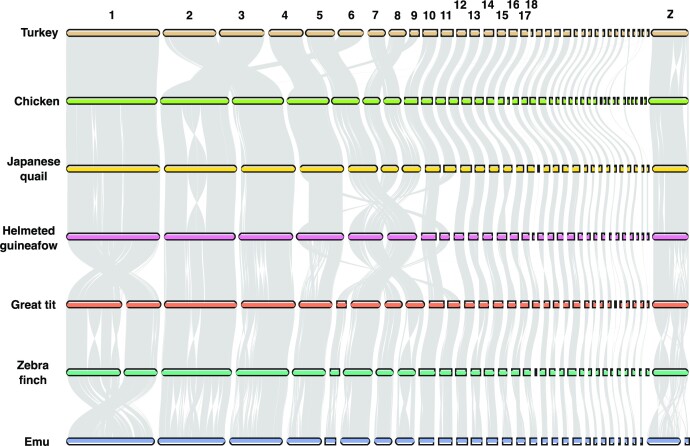
Chromosomal rearrangements across several avian species. Pairwise synteny comparison across 7 birds shows several chromosomal rearrangements. Gray segments represent conserved synteny. Species: turkey (*Meleagris gallopavo*), chicken (*Gallus gallus*), Japanese quail (*Coturnix japonica*), helmeted guineafowl (*Numida meleagris*), great tit (*Parus major*), zebra finch (*Taeniopygia guttata*), and emu (*Dromaius novaehollandiae*).

We performed synteny analysis to assess chromosomal and structural rearrangements within a wide range of avian species. Four Galliformes were included: turkey, chicken, Japanese quail, and helmeted guineafowl. Furthermore, 2 Passeriformes, zebra finch and great tit, and emu, a species from the Casuariiformes order, were included. The multispecies synteny plot shows a high degree of synteny between the avian species on both macro- and microchromosomes, despite the large evolutionary distances (Fig. 4), supported by other studies [36, 37].

**Figure 5:**
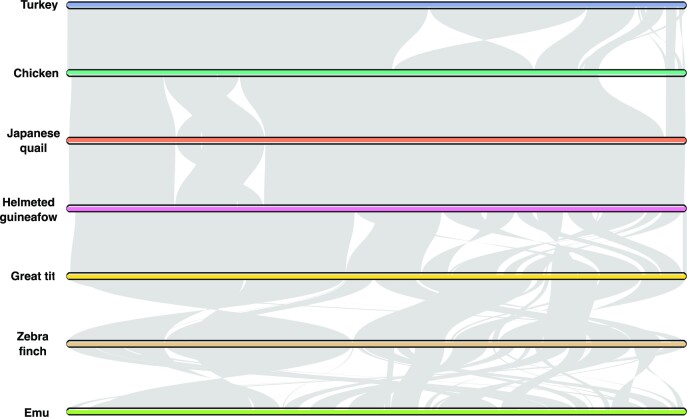
Chromosome Z rearrangements across 7 avian species. Pairwise synteny comparison of the Z chromosome across avian species reveals a large inversion in turkey. Gray segments represent conserved synteny. Species: turkey (*Meleagris gallopavo*), chicken (*Gallus gallus*), Japanese quail (*Coturnix japonica*), helmeted guineafowl (*Numida meleagris*), great tit (*Parus majo*r), zebra finch (*Taeniopygia guttata*), and emu (*Dromaius novaehollandiae*).

Of all chromosomes, it is evident that especially the Z chromosome has been prone to large chromosomal rearrangements between avian orders (Fig. 5) [38]. Interestingly, we found a large inversion of around 19 Mbp on the turkey Z chromosome not found in the other Galliformes and songbirds [5] (Supplementary File 1: Fig. S17). The inversion was supported by a normal alignment at the approximate breakpoints (Supplementary File 1: Table S7–Fig. S18), and the HiC contact map confirmed the accuracy of the assembly (Supplementary File 1: Fig. S19). This is especially striking since rearrangements on the Z chromosome are uncommon within the Galliformes. One region at the tail of the chicken Z chromosome lacks synteny with other Galliformes altogether [39]. This region is enriched in repeat sequences in both chicken and turkey (Supplementary File 1: Fig. S20), as described previously in Bellott et al. [39].

## Discussion

We present a new, chromosome-level, high-quality reference assembly for *M. gallopavo*, Mgal_WU_HG_1.0. The trio-binning approach has been proven to be a robust method to characterize the 2 haplotypes of F1 individuals [14]. The chromosome-level assembly (Supplementary File 1: Table S1) presented in this study confirms the value of this method in not only providing a quality assembly but also uncovering structural genomic variation. The Mgal_WU_HG_1.0 assembly is a large improvement over the previous turkey assembly, Turkey_5.1 [6]. The assembly is now comparable in quality and completeness to the chicken GRCg7 genomes but not as complete as the latest chicken genome [8]. Note that we sequenced a male animal and therefore are lacking the W chromosome. One major limitation of previous turkey assemblies was that they relied on assumptions of high turkey–chicken retained synteny to achieve a chromosome-level assembly. Such assumptions can result in bias, especially when comparing turkey to chicken. Mgal_WU_HG_1.0 does not rely on such comparisons.

Combining long reads and genome-wide chromatin interaction data (Hi-C) enables the capture of chromosome arms in a single contig, resulting in a highly continuous and contiguous chromosome-level assembly. Furthermore, long reads can span long repetitive regions, including DNA transposons and LINE elements, as well as large structural variants. We observe an enrichment of telomeric and TM repeats at the tails of chromosomes, likely indicating telomeric and centromeric regions, as most of the turkey chromosomes are likely telocentric [5]. We show that the centromeres located at chromosome ends mostly comprise TM repeat clusters. Thanks to these recent sequencing technologies, we are able to correct a number of wrongly oriented contigs in Turkey_5.1, a phenomenon often observed in short-read–based assemblies. The improvements in genome quality, completeness, and continuity allow for a more thorough annotation of repeats and gene models. The increase in complete BUSCO genes in Mgal_WU_HG_1.0, compared to Turkey_5.1, indicates a much-improved gene space in the current genome assembly, comparable to the latest chicken genome builds.

Improving genome assemblies improves all analyses that depend on them. One of the reasons to improve the turkey assembly was to better map SNP chip markers to the genome. SNP chips are widely used in genomic selection, and a better genome representation and gene annotation directly affect its use for breeding. Specifically, the new turkey genome build overcomes the lack of SNPs mapped to gene-dense microchromosomes, as 85.3% of the SNP markers previously mapped to unplaced scaffolds on Turkey_5.1 are now mapped to chromosomes on Mgal_WU_HG_1.0, especially improving the representation of microchromosomes 31 to 35.

Turkey breeding is done on pure elite lines, which can be selected for different purposes. In our study, 1 parent was from a female breeding line, with more focus on egg production and conformation, whereas the other parent was from a male breeding line focusing on growth and production traits. In producing a commercial product, lines are crossed to produce hybrid offspring that show the benefit of the breeding goals of both parental lines. In addition, the hybrid offspring benefit from hybrid vigor, resulting from 2 relatively differentiated lines. For the trio-binning method, having parents that are genetically distinct helps in resolving the haplotypes. Nevertheless, in this study, we present 2 high-quality parental haplotype assemblies where the low heterozygosity of the parents presented no obstacle to resolving the parental haplotypes.

Interestingly, we found specific structural variation in *BLB2* (inversion); *TRIM36, GRIA2*, and *MAN2B2* (all duplications); and a loss-of-function variant in the *LRRC41* gene in the parental haplotype from the male line. An additional duplication of the *GEMIN8* gene was identified in the parental haplotype from the female line. The *BLB2* gene plays an important role in the presentation of extracellular antigen and initiation of an immune response [30]. However, the consequence and frequency of the inversion in the parental line remain unclear. The *TRIM36* gene is associated with the spermatozoa acrosome reaction in mice, and knockouts are incapable of *in vitro* fertilization [40]. Hence, the duplication of this gene in the paternal line might have implications on male fertility that require further study. The *GRIA2* gene is a excitatory neurotransmitter associated with various neurodevelopmental disorders in humans [41]. The *MAN2B2* gene is associated with ovulation rate in pigs [31], while in humans, this gene is associated with a disorder of glycosylation [42]. However, the role of this gene and its duplications in avian species remains unclear. The *GEMIN8* gene encodes a protein that is part of the SMN complex, which is necessary for spliceosomal small nuclear ribonucleoproteins (snRNP) assembly in the cytoplasm and pre-mRNA splicing in the nucleus [43]. The *LRRC41* gene is likely knocked out in the male parental haplotype. Knockout mice of the *LRRC41* gene show increased circulating calcium and glucose levels and increased lean body mass [34]. Therefore, this gene is an interesting target gene for breeding, and the identified stop-gained mutation likely causes a loss of function of the protein in the parental line, thereby enhancing growth. However, to further validate this hypothesis, we need to evaluate the frequency in the population and functional consequence of the variant.

Among the remaining challenges in variation analysis is the characterization of structural variants. The challenge is 2-fold. First, these large-scale variants are often not robustly detected using short-read sequencing. Second, individuals usually have a sequence that is population specific and may not be present in a reference assembly. This can make such large insertions hard to characterize, even by resequencing. In the process of assembling Mgal_WU_HG_1.0, we now have reference assemblies for 2 distinct breeding lines, which should greatly aid in variation analysis. Even though such large structural variants appear to be uncommon between breeding lines, we demonstrate how genes potentially important in breeding may be affected. These genes can be further prioritized in routine genomic breeding practice.

As more genomes are characterized with high accuracy and at a chromosome level, comparative genomics is increasingly used to study the function of genes and variants, including copy number variants. The new Mgal_WU_HG_1.0 genome assembly was applied to identify orthogroups that have expanded or contracted in turkey compared to other avian species. Expanded orthogroups included various distinct keratin families, encoding major structural proteins of feathers and claws [44]. One gene family comprising the PHF7 was significantly expanded in turkey. *PHF7* acts during spermiogenesis for histone-to-histone protamine exchange and is a determinant of male fertility in *Drosophila* and mouse [45], and it is highly expressed in rooster testis [46]. This gene family was found to be expanded in chicken as well, with distinct gene clusters on 5 chromosomes [29]. In addition, genes related to immunity and response to stress are expanded in turkey. Further research is needed to disentangle the exact function of these complex gene families.

A characteristic of avian genomes is that they comprise a huge range of chromosome sizes. Interestingly, bird genome organization may be ancestral to all vertebrates [47]. Among the peculiar outcomes is a wide range in, for example, recombination rates, GC bias, gene densities, and variation density throughout the genome [35]. The distinct nature of these features is particularly difficult to study in microchromosomes as they have proven so difficult to characterize. The distinct patters of both gene density and repeat content between the macro- and microchromosomes have been described previously by Kapusta et al. [48]. The Mgal_WU_HG_1.0 assembly, though, has a better representation of the microchromosomes, allowing a better understanding of functional aspects of genes and other genome elements. We have shown that the microchromosomes have a unique repeat landscape enriched for low-complexity, simple, and unknown repeats, especially at the tails of the chromosomes. Together, these efforts provide new insights in microchromosome composition and evolution.

Bird genomes have very high retained synteny [49]. This pattern was confirmed in our analysis of the conserved synteny between several Galliformes (turkey, chicken, Japanese quail, helmeted guineafowl) and 3 outgroups (zebra finch, great tit, emu). Despite the long divergence time that separates turkey and chicken [2], both species have relatively similar karyotypes, confirmed by the high structural continuity and relatively little rearrangements between the 2 birds, even in the microchromosomes. The latter is noteworthy because of the very high recombination rates generally observed in microchromosomes [50], which would suggest that a higher rate of chromosomal rearrangements might be expected but is not observed. Expanding observations to other Galliformes suggest similar degrees of conserved synteny, although comparisons for microchromosomes are less accurate due to the more incomplete assembly of these other Galliformes species.

The Z chromosome presents a moderate yet striking deviation from the observed evolutionary stability. This chromosome exhibits a few rearrangements within the Galliformes, and in line with the findings of Zhang et al. [5], we observed and validated a large inversion in the turkey Z chromosome. As with the Mgal_WU_HG_1.0 assembly, the exact breakpoints of this 19-Mbp inversion on the Z chromosome can now be pinpointed. This inversion is unique for the turkey lineage and not found in any of the other Galliformes.

In conclusion, the new turkey genome presented here (Mgal_WU_HG_1.0) (and the 2 parental haplotype assemblies) represents a substantial improvement over the previous assembly and is an important resource with many applications in research and in the turkey breeding industry.

## Methods

### Data and assembly

To create a high-quality chromosome-level genome assembly of *M. gallopavo*, 3 individuals were sequenced using the trio-binning approach—2 parents and 1 F1. The 2 parents come from 2 distinct commercial lines from Hendrix Genetics, 1 male line (parent 1) and 1 female line (parent 2). The F1 turkey was sequenced by Dovetail Genomics using PacBio SMRT sequencing technology (PacBio Sequel System, RRID:SCR_017989) with a total depth of 270×. We generated short-read sequencing data from the F1 (90.4× coverage) and both parents (35.4× and 39.7× coverage) on an Illumina HiSeq 4000 (HiSeq 4000 System, RRID:SCR_016386). In addition, Hi-C data were generated with a coverage of 32×. An initial assembly was created by Dovetail Genomics using wtdgb2 (WTDBG, RRID:SCR_017225) [15], polished with the PacBio long reads using wtpoa-cns, and scaffolded using the Dovetail *De Novo* Assembly Process, which uses Chicago and Dovetail Hi-C proximation ligation methods and the HiRise scaffolder as described in [16].

### Polishing

Pilon v1.23 (RRID:SCR_014731) [51] was used to polish SNPs and indels based on the short Illumina reads from the F1 (twice with parameters–diploid –mindepth 0.7 –fix bases –changes) and indels with the Illumina reads from parent 2 because of the higher coverage compared to parent 1 (–fix indels).

### Scaffolding

We scaffolded the F1 assembly received by Dovetail Genomics using the Hi-C reads and the PacBio long reads, both from the F1. The Hi-C reads were mapped to the polished assembly based on the Arima Mapping pipeline [52], using BWA-MEM v0.7.17 (RRID:SCR_010910) [53] with default parameters. The filter_five_end.pl script was used to filter and keep the 5′-end. After filtering, the reads were sorted and paired using the two_read_bam_combiner.pl script. This results in a sorted, paired-end BAM file that has been filtered by mapping quality (mapping quality filter = 10). Picard Tools v2.23.4 (RRID:SCR_006525) [54]—AddOrReplaceReadGroups and MarkDuplicates was used to add a read group and remove duplicates. The mapped Hi-C reads were used to scaffold the assembly with SALSA v2.2 (RRID:SCR_022013) [17], which is a scaffolder that uses long-range contact information (Hi-C) with parameters -e “GATC.” Redundans v0.14a [18] was used to scaffold the assembly with the PacBio reads with length >40 Kbp and remove redundant contigs from the final assembly. The parameters -l <long reads> –nogaplosing –noscaffolding were used (–noscaffolding skips short read scaffolding). QV values were calculated using Merqury (RRID:SCR_022964) [22].

### Hi-C validation—misassemblies

To validate our F1 assembly and look for misassemblies, we used Hi-C contact maps.

Juicer v1.6 (RRID:SCR_017226) [55] was used to generate Hi-C contact maps from the Hi-C reads (Supplementary File 1: Fig. S1) and 3D-DNA v180922, a 3-dimensional (3D) *de novo* assembly pipeline (3D de novo assembly, RRID:SCR_017227), to scaffold our assembly. Juicebox v1.11.08 (RRID:SCR_021172) [56] was used to visualize the Hi-C contact map and identify misassemblies. Each breakpoint in the macrochromosomes was manually checked with Juicebox and JBrowse 1.16.9 ( RRID:SCR_001004) [57] to visualize the PacBio read coverage at the breakpoints.

### Haplotype assemblies using trio-binning

TrioCanu (a module from the Canu assembler, v2.1.1) (Canu, RRID:SCR_015880) [14] was used to bin the parental reads to construct parental haplotype assemblies. TrioCanu was run with the short reads from each parent and the F1 PacBio reads with the following options: -p asm genomesize-1.1 g.

The corrected reads from TrioCanu were mapped to the Triocanu assembly with Minimap2 v2.17-r941 (RRID:SCR_018550) [58], options -x map-pb (mapping PacBio). LRScaff v1.1.10 [20] was used to scaffold each parent assembly. For both parents, the scaffolding was done with these parameters: min_contig_length = 500, identity = 1, min_overlap_length = 400, max_overhang_length = 500, max_end_length = 500, min_supported_links = 2, iqr_time = 3. Duplicated sequences were removed using seqkit. RagTag v1.1.1 [21] was used for reference-guided scaffolding of each parental assembly, using the F1 assembly as reference. The scaffold module from RagTag was used with default parameters.

### Completeness

#### Busco

BUSCO v4.1.2 (RRID:SCR_015008) [23] was run to assess the completeness of the assembly in terms of gene space. BUSCO was run in the genome mode (-m genome) and with the vertebrae (vertebrata_odb10) and aves (aves_odb10) datasets (using the flag -l <dataset>).

### Genome comparison—alignment

Genome assembly alignments were generated using D-GENIES v1.3.0 (RRID:SCR_018967) [59], using minimap2 as the aligner. The chromosomes were sorted on length, and noise (short repeat alignments) was removed from the alignment plot.

### Structural variation (parents)

Structural variation between the 2 parental haplotypes was discovered using SyRI v1.5.4 [60]. First, we aligned the 2 haplotype assemblies using minimap2 with settings -ax asm5 –eqx. Next, we used SyRI to identify structural variation using the minimap2 alignment. Results were plotted using plotsr tool v0.5.3 [61]. Large structural variants were manually validated in JBrowse 1.16.9 [57].

### Remapping and variant calling

The short Illumina reads from the F1 individual were mapped back to the assembly using BWA-MEM v0.7.17 (RRID:SCR_010910) [53]. Samblaster v0.1.26 (RRID:SCR_000468) [62] was used to mark duplicates and Samtools v1.14 (RRID:SCR_002105) [63] to sort and index the BAM files. FreeBayes v1.3.1 (RRID:SCR_010761) [64] was used for variant calling with –use-best-n-alleles 4 –min-base-quality 10 –min-alternate-fraction 0.2 –haplotype-length 0 –ploidy 2 –min-alternate-count 2. The vcffilter module from vcflib v0.00.2019.07.10 [65] was used to discard variants with a low phred quality score (<20). Tabix, a module from htslib v1.9 (SAMTOOLS, RRID:SCR_002105) [65], was used to index the VCF files. The stats module from BCFtools v1.9 (SAMtools/BCFtools, RRID:SCR_005227) [66] was used to compute summary statistics of the variant calling. The same process was followed to call variants for each parent. Alignment quality control statistics were computed with QualiMap v.2.2.2-dev (RRID:SCR_001209) [67].

### SNP chip

In order to map SNP markers from the 65,000 SNP array (Illumina, Inc.) to the new genome build, we first aligned the 2 genome builds (Turkey_5.1 and Mgal_WU_HG_1.0) using nucmer v4.0.0rc1 (MUMmer, RRID:SCR_018171) [68]. Next we converted the delta file to a chain file using mugsy v1.2.3 delta2maf and maf-convert (Mugsy, RRID:SCR_001414) [69]. We used CrossMap v0.6.1 (RRID:SCR_001173) [59] to identify SNP locations on the query Mgal_WU_HG_1.0 assembly. We further performed a blastn v2.11.0+ search (BLASTN, RRID:SCR_001598) [70] to identify the locations of SNPs that could not be mapped from the previous build using the SNP probe sequences.

### Annotation and repeats

Tandem repeats were identified using the TRF tool [71], and telomeric and TM repeats were identified using the tidk package [72]. The genome was annotated with the ENSEMBL annotation pipeline and is available as part of the Ensembl Rapid Release (RRID:SCR_002344) [25]. The transcriptome and proteome evidence used in the annotation are listed in Supplementary File 7. We used a custom Python script to query the Ensembl rapid release homolog gene page to identify Turkey_5.1 and GRCg6a homologs of all the Mgal_WU_HG_1.0 genes. The BuildDatabase tool from RepeatModeler v1.0.11 (RRID:SCR_015027) [24] was used to build a *de novo* repeat library from our assembly using the Recon and RepeatScout tools. RepeatMasker v4.0.7 (RRID:SCR_012954) [73] was used to identify repeats together with the custom build repeat library from RepeatModeler.

### Orthologs

The proteomes of 5 bird species were used to infer orthogroups (option -og) using OrthoFinder v2.5.4 (RRID:SCR_017118) [74]. The proteomes of the following assemblies were downloaded from Ensembl release 106: turkey—Turkey_5.1; chicken—GRCg6a; Japanese quail—Coturnix_japonica_2.0; helmeted guineafowl—NumMel1.0; and zebra finch—bTaeGut1_v1.p. The proteomes for Mgal_WU_HG_1.0 (turkey) and GRCg7b (chicken) were downloaded from the Ensembl rapid release (March 2022). For each orthogroup, the protein isoform with the best alignment based on species similarity, score, and expect value was chosen. Turkey-specific orthogroups were analyzed by running BLASTP v2.11.0+ (RRID:SCR_001010) [70] against the NR database to identify homologous genes from a wider range of species.

### Gene family contractions and expansions of protein-coding gene families

Expansions and contractions of protein-coding gene families were assessed by CAFE5 [28]. The phylogenetic tree was obtained using the BirdTree database [75].

### Distinct genomic landscapes of turkey micro- and macrochromosomes

To better understand the differences between macrochromosomes (>40 Mbp), intermediate chromosomes (>40 Mbp, <20 Mbp), and microchromosomes (<20 Mbp), we investigated repeat content, gene structure, and gene expression. A Welch *t*-test was used to test for difference of repeat content and families between macrochromosomes, intermediate chromosomes, and microchromosomes.

### Repeats

A custom repeat library created with RepeatModeler and custom R scripts was used to investigate the differences in repeat content between macrochromosomes, intermediate chromosomes, and microchromosomes. Each chromosome was split into bins (each bin corresponding to 2% of the chromosome length), allowing us to compare the chromosomes by relative length. We calculated the average repeat content in each bin. An ideogram of the density of each repeat feature was created for macrochromosomes, intermediate chromosomes, and microchromosomes with the R v4.0.2 (R Project for Statistical Computing, RRID:SCR_001905) [76] package RIdeogram v0.2.2 [77]. RIdeogram calculates feature density in sliding windows (100 Kbp for macrochromosomes and intermediate chromosomes, 50 Kbp for microchromosomes).

### Tissue specificity

Expression data for 16 turkey tissues (jejunum, proventriculus, thigh, testis, ileum, pancreas, spleen, breast, brain, heart, thymus, liver, gizzard, duodenum, cecal tonsil, bursa) from a male individual at 3 developmental stages (14, 21, and 28 days posthatch) were downloaded from Bioproject PRJNA259229. Not all tissues were available at all stages: testis was not available at day 21 and cecal tonsil at day 28. HISAT2 v2.2.1 (RRID:SCR_015530) [78] was used to index the assembly (hisat2-build) and align the RNA-seq reads to the assembly. StringTie v2.1.7 (RRID:SCR_016323) [79] was used to assemble transcripts using the aligned reads and Ensembl gene annotation with options -A and -B. A nonredundant set of transcripts was generated with StringTie's merge option (–merge), which creates a unified set of transcripts from several samples. StringTie was run once more, now using this new set of transcripts as the reference annotation file. The resulting table containing the gene abundance of all genes was used in our analysis. We analyzed the results through custom R (v4.0.2) scripts. We started by filtering the gene abundance table to keep only the genes that are expressed (fragments per kilobase million >1). Then we classified genes into housekeeping (expressed in at least 13 tissues), less specific (expressed in at least 5 and in fewer than 13 tissues), specific (expressed in 2 to 5 tissues), and more specific genes (expressed in 1 or 2 tissues). The relative abundance of housekeeping/specific genes was calculated by counting the number of genes in these categories in macrochromosomes, intermediate chromosomes, and microchromosomes and dividing that by the total amount of genes in each chromosome type.

### Gene structure

We used RIdeogram v0.2.2 [77] and R (v 4.0.2) to compare the gene density between the chromosome classes. RIdeogram calculates gene density in sliding windows, 100 Kbp for macrochromosomes and intermediate chromosomes, 50 Kbp for microchromosomes. Gene density per megabase was calculated by dividing the number of annotated genes on a chromosome by its length. A Welch *t*-test was used to test for difference of gene densities between macrochromosomes, intermediate chromosomes, and microchromosomes.

### Synteny

The MCScan Python pipeline from the JCVI utility libraries v1.1.11 (MCScan, RRID:SCR_017650) [80] was used to study chromosomal rearrangements between several bird species: turkey (*M. gallopavo*), chicken (*G. gallus*), Japanese quail (*C. japonica*), helmeted guineafowl (*N. meleagris*), great tit (*P. major*), zebra finch (*T. guttata*), and emu (*D. novaehollandiae*).

The genome (fasta coding DNA sequence, CDS) and annotation files for these species were obtained from Ensembl release 106. The files for Mgal_WU_HG_1.0 and GRCg7b were obtained from the Ensembl rapid release (April 2022). The annotation file for the emu assembly ZJU1.0 was shared with us from [81]. This annotation file, in combination with the FASTA file obtained from NCBI, was used to create the CDS fasta file necessary for the pipeline.

We started by trimming the accession IDs in the FASTA file and converting the GFF3 annotation file to BED format. The jcvi.compara.catalog ortholog and jcvi.compara.synteny screen (with parameters –simple) were used to create the necessary input files for plotting. The synteny plots were created with jcvi.graphics.karyotype using parameter –basepair. To validate the chromosome Z inversion, first we manually checked the inversion breakpoints (reads spanning) using JBrowse 1.16.9.

## Supplementary Material

giad051_GIGA-D-22-00193_Original_Submission

giad051_GIGA-D-22-00193_Revision_1

giad051_GIGA-D-22-00193_Revision_2

giad051_GIGA-D-22-00193_Revision_3

giad051_Response_to_Reviewer_Comments_Original_Submission

giad051_Response_to_Reviewer_Comments_Revision_1

giad051_Reviewer_1_Report_Original_Submission Yunyun Lv -- 8/22/2022 Reviewed

giad051_Reviewer_1_Report_Revision_1 Yunyun Lv -- 12/27/2022 Reviewed

giad051_Reviewer_2_Report_Original_Submission Luohao Xu -- 9/8/2022 Reviewed

giad051_Reviewer_2_Report_Revision_1 Luohao Xu -- 12/24/2022 Reviewed

giad051_Reviewer_2_Report_Revision_2 Luohao Xu -- 4/29/2023 Reviewed

giad051_Supplemental_Files

## Data Availability

The genome assemblies and sequencing data have been deposited in ENA under Bioproject accession PRJEB42643. The turkey genome and annotations are available through ENSEMBL Rapid Release [82]. All supporting data and materials are available in the *GigaScience* GigaDB database [83].

## Additional files

**Supplementary File 1: Table S1**. Genome assembly and annotation overview.

**Supplementary File 1: Table S2**. QV values indicating assembly quality and completeness.

**Supplementary File 1: Table S3**. Protein homology between Mgal_WU_HG_1.0, Turkey_5.1, and chicken (GRCg6a).

**Supplementary File 1: Table S4**. Blast results of proteins in Mgal_WU_HG_1.0–specific orthogroups.

**Supplementary File 1: Table S5**. Mapping of 65,000 markers on Mgal5.1 and Mgal_WU_HG_1.0.

**Supplementary File 1: Table S6**. Mapping rate of RNA-seq datasets from 16 tissues to Mgal_WU_HG_1.0. Tissues (jejunum, proventriculus, thigh, testis, ileum, pancreas, spleen, breast, brain, heart, thymus, liver, gizzard, duodenum, cecal tonsil, bursa) are from a male individual at 3 developmental stages (14, 21, and 28 days posthatch).

**Supplementary File 1: Table S7**. Mummer alignment between Turkey_5.1 and Mgal_WUR_HG_1.0 of the first and the second breakpoints of the 19.4-Mbp inversion on the Z chromosome.

**Supplementary File 1: Fig. S1**. Hi-C contact map of the Mgal_WU_HG_1.0 assembly.

**Supplementary File 1: Fig. S2**. Overview of tandem repeats identified in the turkey genome.

**Supplementary File 1: Fig. S3**. Parent 1 vs. parent 2 alignment.

**Supplementary File 1: Fig. S4**. Distribution of copy gains and copy losses in parent 2 compared to parent 1 haplotype.

**Supplementary File 1: Fig. S5**. Inversion comprising the start of the *BLB2* gene in parent 2 compared to the parent 1 haplotype.

**Supplementary File 1: Fig. S6**. Duplication affecting the tail of the *MAN2B2* gene in parent 2 compared to the parent1 haplotype.

**Supplementary File 1: Fig. S7**. Duplication affecting *GEMIN8* gene in parent 2 compared to the parent1 haplotype.

**Supplementary File 1: Fig. S8**. Duplication affecting *RIMKLB* gene with higher copy number in parent 2 compared to parent 1 haplotype.

**Supplementary File 1: Fig. S9**. Average DNA repeat content along the chromosomes for macrochromosomes, intermediate chromosomes, and microchromosomes.

**Supplementary File 1: Fig. S10**. Average LINE repeat content along the chromosomes for macrochromosomes, intermediate chromosomes, and microchromosomes.

**Supplementary File 1: Fig. S11**. Average low-complexity repeat content along the chromosomes for macrochromosomes, intermediate chromosomes, and microchromosomes.

**Supplementary File 1: Fig. S12**. Average LTR repeat content along the chromosomes for macrochromosomes, intermediate chromosomes, and microchromosomes.

**Supplementary File 1: Fig. S13**. Average simple repeat content along the chromosomes for macrochromosomes, intermediate chromosomes, and microchromosomes.

**Supplementary File 1: Fig. S14**. Average SINE repeat content along the chromosomes for macrochromosomes, intermediate chromosomes, and microchromosomes.

**Supplementary File 1: Fig. S15**. Average snRNA repeat content along the chromosomes for macrochromosomes, intermediate chromosomes, and microchromosomes.

**Supplementary File 1: Fig. S16**. Average unknown repeat content along the chromosomes for macrochromosomes, intermediate chromosomes, and microchromosomes.

**Supplementary File 1: Fig. S17**. Chromosome Z alignment showing inversion with GRCg7b (A) and Turkey_5.1 (B).

**Supplementary File 1: Fig. S18**. Alignment of corrected PacBio reads at the approximate breakpoints of the ∼19.4-Mbp inversion on the Z chromosome.

**Supplementary File 1: Fig. S19**. HiC contact map of the Z chromosome.

**Supplementary File 1: Fig. S20**. Schematic view of Gal7b chromosome Z and representation of several biotypes of genes and genomic features (Ensembl, rapid release 15 June 2022, accessed 27 June 2022).

**Supplementary File 2**. Repeat annotation.

**Supplementary File 3**. Gene family expansions and contractions.

**Supplementary File 4**. Syri output showing structural variation between the 2 parent haplotypes.

**Supplementary File 5**. Structural variation between parent haplotypes.

**Supplementary File 6**. Stop-gained variants identified in either or 1 of 2 parent haplotypes.

**Supplementary File 7**. Transcriptome and proteome evidence used for ENSEMBL annotation.

## Abbreviations

BED: Browser Extensible Data; BLAST: Basic Local Alignment Search Tool; BLASTN: BLAST search of nucleotide database(s); BLASTP: BLAST search protein databases using a protein query; bp: base pairs; BUSCO: Benchmarking Universal Single-Copy Orthologs; BWA: Burrows–Wheeler Aligner; CDS: coding sequence; F1: Filial 1, first offspring from a cross; GC: guanine-cytosine; GFF3: general feature format, version 3; GWAS: genome-wide association study; Hi-C: chromosome conformation capture; indel: insertion or deletion; Kbp: kilobase pairs; LINE: long interspersed nuclear element; lnRNA: long noncoding RNA; LoF: loss of function; Mbp: megabase pairs; NCBI: National Center for Biotechnology Information; PacBio: Pacific Biosciences; SMN: survival motor neuron; SMRT: single molecule real time; SNP: single-nucleotide polymorphism; VCF: variant call format.
