# Supplementary material for: A new haplotype-resolved turkey genome to enable turkey genetics and genomics research
Source: Gigascience. 2023 Jul 21;12:giad051. doi: 10.1093/gigascience/giad051 (PMC10360393; doi:10.1093/gigascience/giad051)
Supplement: giad051_GIGA-D-22-00193_Revision_1 [file giad051_giga-d-22-00193_revision_1.pdf]

GigaScience

A new haplotype-resolved turkey genome to enable turkey genetics and genomics research

--Manuscript Draft--

**Manuscript Number:**	GIGA-D-22-00193R1
**Full Title:**	A new haplotype-resolved turkey genome to enable turkey genetics and genomics research
**Article Type:**	Research
**Funding Information:**	Stichting voor de Technische Wetenschappen (14283) — Mr Martien A.M. Groenen
**Abstract:**	Background<br>The domesticated turkey (Meleagris gallopavo) is a species of significant agricultural importance and is the second largest contributor, behind broiler chickens, to world poultry meat production. The previous genome is of draft quality and partly based on the chicken (Gallus gallus) genome. A high-quality reference genome of Meleagris gallopavo is essential for turkey genomics and genetics research and the breeding industry.<br>Results<br>By adopting the trio-binning approach, we were able to assemble a high-quality chromosome-level F1 assembly and two parental haplotype assemblies, leveraging long-read technologies and genome-wide chromatin interaction data (Hi-C). From a total of 40 chromosomes (2n=80), we capture 35 chromosomes in a single complete scaffold, and show much improved genome completeness and continuity compared to the old assembly build. The three assemblies are of higher quality than the previous draft quality assembly and comparable to the current chicken assemblies (GRCg6a and GRCg7) shown by the largest contig N50 (26.6 Mb) and comparable BUSCO gene set completeness scores (96-97%). Comparative analyses reveal a large inversion of around 19 Mbp on the Z chromosome not found in other Galliformes. Structural variation between the parent haplotypes were identified which pose potential new target genes for breeding.<br>Conclusions<br>We contribute a new high-quality turkey genome at chromosome-level, benefiting turkey genetics and other avian genomics research as well as turkey breeding industry.<br>.
**Corresponding Author:**	Martijn Derks, Ph.D.<br>Wageningen University & Research<br>Wageningen, NETHERLANDS
**Corresponding Author Secondary Information:**	
**Corresponding Author's Institution:**	Wageningen University & Research
**Corresponding Author's Secondary Institution:**	
**First Author:**	Carolina Pita Barros
**First Author Secondary Information:**	
**Order of Authors:**	Carolina Pita Barros
	Martijn Derks, Ph.D.
	Jeff Mohr
	Benjamin Wood
	Richard P.M.A. Crooijmans
	Hendrik-Jan Megens

Powered by Editorial Manager® and ProduXion Manager® from Aries Systems Corporation

	Marco C.A.M. Bink
	Martien A.M. Groenen, Professor
**Order of Authors Secondary Information:**	
**Response to Reviewers:**	Reviewer reports:<br><br>Reviewer #1: The turkey has importance for agriculture as it is the second contributor to word poultry meat production. This study completes a chromosome-scale genome assembly with long reads sequencing and use trio-binning approach to generate a haplotype-resolved turkey genome, which give scientific significance to further genetic studies within this species. However, I feel the content within this article need improvement. Some parts were unclear and hard to follow, I list some of them as below. After substantial revisions, I will suggest the publication.<br><br>Dear reviewer, thanks for the useful suggestions, we have now addressed your comments as good as possible. We specifically now provide additional metrics to support the quality of our assembly and annotation. For example, we now also include the BUSCO values for the annotation and the QV values of the assemblies. We also now improved our gene family analysis and provided further evidence of the structural variation. We hope that hereby we have sufficiently addressed your comments and concerns.<br><br>In abstract,<br><br>The sentence "These assemblies cover 35 chromosomes in a single scaffold and show improved genome completeness and continuity" seems weird and hard to understand directly. Please revise it and make it clear.<br><br>We agree this sentence was not completely clear, we now updated the sentence to make it more clear and refer to N50 and BUSCO scores to support that. Line X-X<br><br>"The three assemblies are of higher quality than the previous draft quality assembly and comparable to the current chicken assemblies (GRCg6a and GRCg7)." Please indicate the parameters used for comparison clearly and how prove them with a higher quality.<br><br>Again, we now emphasize on metrics to support our statements.<br><br>"Structural variation between the parent haplotypes were identified in genes involved in growth providing new target genes for breeding." The theoretical context of this sentence is not clear, so I suggest more information added to make it clear.<br><br>We agree that given we did not do a GWAS, we cannot state that the gene is involved in growth in this line directly. Hence, we chose to put less emphasize on this and rewrite the sentence. We did however include screenshots and additional evidence to support our identied SVs.<br><br>Considering no statistic in the conclusion, I suggest the conclusion sentence can be revised as "we contribute a new high-quality turkey genome at chromosome-level, benefiting turkey genetics and other avian genomics research as well as turkey breeding industry."<br><br>Thank you for this suggestion, we updated this accordingly.<br><br>In the introduction,<br>"Most of the chromosomes are small microchromosomes, while only a few macrochromosomes are present in the karyotype." Please clearly indicate how many microchormosomes in turkeys and chicken. "most of" is uninformative for readers.<br><br>We agree we should be more precise. However, the defeninition of a macro, intermediate or microchromosome is not always clear. Nevertheless, we rephrased this sentence: “The turkey karyotype consists of 7 macrochromosomes (>50 Mb), four intermediate chromosomes (>20 Mb, <40 Mb), and the rest being microchromosomes

(<20Mb)."

"and by current standards would be considered of draft quality". What is the current standards? Please indicate it clearly.

We have now emphasized that the genome is of draft quality given the low contig and N50 and lack of third generation long read sequencing data.

"Ongoing efforts in producing high quality assemblies of the microchromosomes in avian genomes have been unsuccessful due to multiple causes" what the multiple causes represent for? Or the features of microchromsomes leads to the unsuccessful assembly as mentioned above?

The main issues are indeed mentioned above in the paragraph (high GC content, repeat content, and lack of linkage group markers). We now mention this at the tail of the paragraph.

"For instance, improved annotation of (non)-coding genes benefits the functional interpretation of genome wide association studies (GWAS), and aids in identifying targets for gene editing", why are non-coding genes (I understand the non-coding genes are referred as regulatory regions, but actually, they are not real genes.) benefits …? Why protein-coding genes (structural genes) can not undertake the roles?

We do not agree that non-coding RNAs are not real genes. Yes, they do not code for a protein, but they are genes often referred to as RNA genes. Yes, protein coding genes are the prime genes to look at when doing finemapping of GWAS results. However, sometimes the causal variant is not within a protein coding gene but is found to be a e.g. long non coding RNA (lncRNA). We therefore also put (non) between parenthesis in the sentence. Together, we are convinced that a better annotation of non-coding genes will benefit studies that do finemapping of causal variants from QTL regions.

"The genome assemblies of turkey (this paper) and chicken, however, are of considerably higher quality compared to other Galliforme species. This provides opportunities for an in-depth comparison between the two most important avian agricultural species." I cannot follow the logic of why the placement of this sentence is here. Obviously, it should be part of discussion after the comparison of turkey genome with other avian genomes.

We agree with your suggestion, and discarded the sentence that was more discussion.

"In this study we use a relatively new technique, the trio-binning approach, to construct high quality haplotype-resolved turkey assemblies." I feel it is necessary to give an explanation of the term "trio-binning approach" as many readers do not understand what is standard for? And the long-reads sequencing technology within it also connect the former theoretical context closely.

We now explain the trio-binning approach in detail at the tail of the paragraph, we feel that we provide sufficient information for the reader to understand the trio-binning approach.

In results,

Have you used other assemblers to complete the genome assembly? Such as flye, or nextdenovo, or mecat2 that may have better performance.

No we did not use other primary assembly tools. One reason is that we already got a good quality primary assembly (contig N50: 26.5 Mb). We did do, however, extensive scaffolding both with HiC (SALSA) and with the pacbio read data (Redundans). We therefore believe the assembly and annotation is of very high (chromosome-level) quality.

Have you ever try 3D-dna for chromosome-scale assembly? which may be better as my experience.

Powered by Editorial Manager® and ProduXion Manager® from Aries Systems Corporation

Yes, we have used 3D-dna. However, in our case we had a chromosome-scale assembly after the scaffolding (all chromosomes in 1 scaffold except for a few very small microchromosomes), therefore we did use 3D-dna to further refine our assembly.

The gene annotation should be assessed by BUSCOs.

This is a good suggestion. We have now run BUSCO on our proteins dataset and the other protein datasets as well. Find the results in Table 3. As indicated, the BUSCO scores of our annotation as about similar to the GRCg7b gene annotation and much better compared to Gal6 and the previous turkey genome assembly.

In discussion,

"The quality of the assemblies presented in this study confirms the value of this method in not only providing a quality assembly but also in uncovering structural genomic variation." Please indicate which quality index that reflect your genomic assembly.

We agree that we can be more precise in the quality measures. We therefore now also included the QV values in the supplementary material. Hence, we now stated that we have a chromosome level assembly and added a table to the supplementary file 1 that provides an overview of the assembly and annotation (Table S1).

"Thanks to these recent sequencing technologies, we are able to correct a number of wrongly oriented contigs in Turkey_5.1, a phenomenon often observed in short-read based
assemblies." I feel this sentence is not formal in writing.

Short read based assemblies are known to be fragmented and can put contigs in wrong orientation towards each other. We think we describe this accurately in this sentence and we do not see how to improve this.

Reviewer #2: This manuscript by Barros et al. presents a high-quality dipoid turkey genome assembly which shows significant improvement relative to the previous one. This new assembly is timely and will likely be used as the reference turkey genome, but the authors should acknowledge that the W chromosome is absent (because the F1 individual was a male?). This manuscript fits more with "Data Note" than "Research" as I see most results are descriptive and confirmatory.

Dear reviewer 2. We would like to thank you for your useful comments. We believe we have included many additional analysis and support to improve our manuscript and we also believe we now provide sufficient evidence of the quality of our genome assembly and structural variations. We also included in the first paragraph of the discussion that W chromosome is missing because we sequenced a male bird. We do not think this manuscript is more like a Data Note. We performed a thorough analysis of the genome and the two parental haplotypes. First we evaluated the quality of the assembly and annotation, which we believe is very important in a genome paper, especially given the agricultural importance of this species. Moreover, we provide now more details and evidence on the structural variation between the parent haplotypes and we provide a more in depth gene family (using CAFE5) and marco-micro chromosome feature comparisons including statistical tests. We also provide a set of additional evidence supporting the Z-chromosome inversion (HiC map and breakpoint alignments). All in all, we believe that we have much improved the manuscript and we hope we adhere to the comments and suggestions raised by the reviewer.

While the chromosomal assembly is relatively complete, I am concerned whether it still contains assembly errors (because of not being polished by long reads?) which led to fewer genes annotated.

We did actually polish the assembly also with the long reads using wtpoa-cns, which is part of the wtdgb2 assembler. We now included this in the material and methods. In addition we did three round of polishing using the Illumina short reads. We are convinced that our assembly does not contain much errors anymore. This is also reflected by the high genome BUSCO score and high proteome BUSCO score (even higher than Gal6 and comparable to Gal7) given in Table 3.

Powered by Editorial Manager® and ProduXion Manager® from Aries Systems Corporation

This assembly metric needs to be taken into accounts if this assembly were to be used as a reference. The authors need to provide the QV value (see the VGP standard), and evaluate indel errors in coding regions.

We ran Mercury to obtain the QV values for the F1 assembly and the two parental assemblies. The QV values are very high. We find a value of 38.7 for the F1 assembly and a value >42 for the parental haplotypes. Also completeness is high >95% for the parental assemblies. The values are given in Supplementary File 1: Table S2.

Some of the results are very brief without showing details or a figure, so difficult for assessment, for instance those SVs affecting genes.

Thank you for this remark, we have elaborated our results of the structural variation and the gene family analysis. We now provide figures showing the structural variation in the supplementary material.

Page 4, "two most important avian agricultural species", I think duck should be the second most important poultry species?

According to citation 1: “Association of Poultry Processors and Poultry Trade in the EU Countries (AVEC)” table 11 and table 13, Turkey is slightly more consumed and bred compared to duck worldwide.

Page 5, I believe the "F1 assembly" refers to the primary assembly or collapsed assembly - please define it more clearly.
We have now indicated that the F1 is the primary assembly.

Page 6, it's unclear how the 36 chromosome models are defined, particularly for small microchromosomes (29-35). According to the karyotype of turkey (2n=80), a few chromosomal models are missing.

Yes, we are still missing a few microchromosomes. We lack linkage data to separate the scaffolds into different chromosomes. We defined the chromosomes 29-35 by size. We also checked homology with the Gal7 genome (Supplementary file 1: Table S1). We now added a table in supplementary material indicating this homology, although there was not always clear homology with Gal7, especially for the very small microchromosomes <1Mb. The last 5 microchromosomes are likely too small to define as sperate chromosomes, and we also lack linkage data to prove these are separate microchromosomes. Hence, the last few “missing” microchromosomes are likely in the assembly as an unplaced scaffold.

Page 6, "This captures the chromosome arms in a single contig" does it apply to all chromosomes? This is unlikely, and data is not shown.

This is a valid point, we changed the sentence accordingly and we have put the number of spanned gaps in the supplementary material (Supplementary File1: Table S1). Most chromosomes have between 0-2 gaps in the scaffold, indicating that yes we do cover most chromosome arms in a single contig (except for chromosome 1, 27 and Z) and for many chromosomes we even span the complete centromere (zero gaps).

Page 6, any idea why the coverage of two parents differs (110X vs. 137X)?
No, we do not know why we have higher coverage for the parent 2 haplotype.

Page 6, "anchored the assemblies to the F1 assembly using RagTag". This suggests and chromosomal assembly of the two haplotypes was not independent, and replied on the F1 assembly. This can potentially lead to missing structural variations between two haplotypes (inversions, translocations).
This is a valid point. Note that we mostly find inversions within single contigs and therefore we do not expect that we miss very large inversions or other structural variation due to the anchoring of the assembly to the F1. We still capture translocations because we do not lose any contigs after anchoring and therefore we will still be able to map all translocations. The anchoring of the contigs is especially useful to provide a clear pairwise comparison of the two parental haplotypes on a chromosome level (as in

Supplementary File 4). However, we do see the point that the location of the breakpoints might be incorrect if we anchor the assembly. Nevertheless we think this might only be the case if there are very large structural differences between the two parental haplotypes, which we did not observe at all, also given that our parental lines are genetically relatively similar.

Page 7, please show more data to support the correct assembly of the chrZ inversion, including Hi-C heatmap, and long-read alignment spanning the inversion breakpoints. Note the Z chromosome inversion has been reported in Zhang et al. 2011 (BMC genomics), which is not cited until in the Discussion.

Thank you for raising this point. We now cite the paper of Zhang et al already in the introduction. In addition, we now provide the chrZ alignment between the old turkey genome and the chicken genome. Also we provide the approximate coordinates of the breakpoint and the HiC contact map of the Z-chromosome and a screenshot of the pacbio read alignment at the approximate breakpoints of the inversion All results support the inversion.

Page 8, it's possible some genes were not annotated because of the presence of indels in coding regions. The genome assembly QV value can be calculated to measure the error frequency (Rhie et al, 2021 Nature).

We now provide the QV value (in Supplementary file 1 Table S3, as described above). The QV values indicate high quality and completeness of all assemblies. Moreover, we ran BUSCO on the gene annotation and we show much higher completeness compared to Gal6 and comparable to the new Gal7 genome build.

Page 8, please provide a statistical result for gene density comparison.
We now perform a statistical test to show significant difference in gene dens ities. In addition, we provide the gene density per Mb in Table S1. As expected the difference in gene densities between the microchromosomes and the macro-intermediate chromosomes is highly significant as described in the results: “As expected, microchromosomes show higher gene density compared to macro and intermediate chromosomes ($P<0.00001$, Figure 2). The density generally increases with decreasing microchromosome size.”

Page 8, at the bottom, please cite the sources of these bird genomes.
We have now added the citations of the species used in the analysis.

Page 9, "Gene family contractions and expansions". These analyses were a bit crude. " Orthologous groups" is not equivalent to "gene family".
We now provide a more thorough analysis of gene family expansions and contractions using CAFE5. We now also find more gene families that are expended or contracted specifically for the turkey lineage.

Page 10, the phrase "F1 and parent assemblies" is confusing. Both haploid assemblies are derived from the diploid F1. Consider changing to "paternal and maternal genomes".

We changed this according to your suggestion.
Also, as I commented above, both parental chromosomal assemblies are based on the same reference (Mgal_WU_HG_1.0), so the contigs were ordered and placed in the same way.
As mentioned above, the contigs are already very large from the parental haplotypes. So we would be able to identify most of the structural variation, for comparative analysis we thought it would be good anchor the parental haplotypes to the F1 assembly. We still believe we capture the structural variation, despite the anchoring of the scaffolds. Note also that the (structural) variation between the two parent haplotypes is fairly limited, hence, we do not expect extremely large structural differences, and we also have no evidence to support this.
This process could mask the potential non-co-linear segments. For a more appreciated way to independently assemble two chromosome-level assemblies, see the marmoset diploid genome paper (Yang et al., 2021 Nature).

Powered by Editorial Manager® and ProduXion Manager® from Aries Systems Corporation

We now provide an overview of the structural variation between the parental haplotypes in the supplementary material.

Page 10, please use a figure to show the SV over the BLB2 gene.
We have included a figure showing the inversion affecting the BLB2 gene.

Page 11, again, please visualize the result on the MAN2B2, GEMIN8, RIMKLB and RALYL cases.

We now provide figures in the supplementary material supporting these structural variation events.

Page 11, "Loss of function variation", I am wondering whether variations mentioned in this part are fixed in the corresponding populations?
Unfortunately we cannot confirm this as we do not have further whole genome sequence data from the populations under study.

Page 11, "Knockouts of this gene lead.." reference is needed.
We now added a reference.

Page 12, "Avian genomes are known to…" references are missing.
We now added a reference.

Page 12, "Distinct genomic landscapes of turkey micro and macrochromosomes", some patterns have been described in the literature, for instance, 10.1111/nyas.13295. Please also perform some statistical analyses to support the claims, not just a figure.
We now performed statistical tests for support the enrichment of specific repeats in macro-and micro chromosomes. In addition, we provide a comprehensive overview of the repeat content per chromosome and per repeat class (Supplementary file 2). We also discuss the scientific paper indicated by the reviewer in the discussion now.

Page 13, "Conserved synteny within the Galliformes clade", please cite 10.1159/000078570 and 10.1007/s00412-018-0685-6

Citations are added

Page 13, "it is evident that especially the Z chromosome" also observed in 10.1038/s41559-019-0850-1
We have now cited this paper.

Page 13, "inversion of around 19 Mbp on the turkey Z" also reported in 10.1186/1471-2164-12-447
We have now cited this paper

Page 14, "tail of the chicken Z chromosome lacks synteny" also reported in 10.1038/nature09172. This means figure S11 does not provide a novel finding.
We agree that this has been described previously, but we think it is important to indicate this because it can improve the interpretation of the alignments. We do, however, now cite this paper in the same sentence.

Page 14, "Combining long reads and genome-wide chromatin interaction data (Hi-C) enables the capture of chromosome arms in a single contig", again, is that correct, chromosome arms in a single contig?
Yes, we have generally between 0 and 3 contigs per chromosome (Supplementary file 1: Table S1). Hence, we are convinced that we capture chromosome arms in a single scaffold for many chromosomes.

Page 18, it's known wtdgb2 assembly tends to contain errors, but it looks the authors did not use long reads for polishing, but only used short reads?
As mentioned above. We did actually polish the assembly also with the long reads using wtpoa-cns, which is part of the wtdgb2 assembler. We now included this in the material and methods. In addition we did three round of polishing using the illumina short reads. We are convinced that our assembly does not contain much errors anymore. This is also reflected by the high genome BUSCO score and high proteome

Powered by Editorial Manager® and ProduXion Manager® from Aries Systems Corporation

	BUSCO score (even higher than e.g. Gal6 and comparable to Gal7).<br><br>Page 20, "The corrected reads from TrioCanu were mapped to the Triocanu assembly with Minimap2 v2.17-r941 (Minimap2, RRID:SCR_018550) [45], options -x map-pb", what was is used for?<br>The option map-bp is used to map back pacbio reads to the assembly. We further used the mapping to validate structural variation in the genome browser JBrowse.<br><br>Page 20, "Duplicated sequences were removed." How was this done?<br>We used seqkit for this, we now added this in the material and methods.
**Additional Information:**	
**Question**	**Response**
Are you submitting this manuscript to a special series or article collection?	No
**Experimental design and statistics**<br><br>Full details of the experimental design and statistical methods used should be given in the Methods section, as detailed in our Minimum Standards Reporting Checklist. Information essential to interpreting the data presented should be made available in the figure legends.<br><br>Have you included all the information requested in your manuscript?	Yes
**Resources**<br><br>A description of all resources used, including antibodies, cell lines, animals and software tools, with enough information to allow them to be uniquely identified, should be included in the Methods section. Authors are strongly encouraged to cite Research Resource Identifiers (RRIDs) for antibodies, model organisms and tools, where possible.<br><br>Have you included the information requested as detailed in our Minimum Standards Reporting Checklist?	Yes
**Availability of data and materials**<br><br>All datasets and code on which the	Yes

Powered by Editorial Manager® and ProduXion Manager® from Aries Systems Corporation

conclusions of the paper rely must be either included in your submission or deposited in publicly available repositories (where available and ethically appropriate), referencing such data using a unique identifier in the references and in the "Availability of Data and Materials" section of your manuscript.<br><br>Have you have met the above requirement as detailed in our Minimum Standards Reporting Checklist?	

Powered by Editorial Manager® and ProduXion Manager® from Aries Systems Corporation

Manuscript including track changes

Click here to access/download;Manuscript;Manuscript_Turkey_Genome_W

A new haplotype-resolved turkey genome to enable turkey genetics and genomics research

Carolina P. Barros[1], Martijn F.L. Derks[1*], Jeff Mohr[2], Benjamin Wood[2,3], Richard P.M.A. Crooijmans[1], Hendrik-Jan Megens[1], Marco C.A.M. Bink[4], Martien A.M. Groenen[1]

[1]Wageningen University and Research, Wageningen, Netherlands

[2]Hybrid Turkeys, Kitchener, ON, Canada

[3]School of Veterinary Science, University of Queensland, Gatton, QLD, Australia

[4]Hendrix Genetics Research, Technology & Services, Boxmeer, Netherlands

* Correspondence:

Martijn F.L. Derks

martijn.derks@wur.nl

Background

The domesticated turkey (*Meleagris gallopavo*) is a species of significant agricultural importance and is the second largest contributor, behind broiler chickens, to world poultry meat production. The previous genome is of draft quality and partly based on the chicken (*Gallus gallus*) genome. A high-quality reference genome of *Meleagris gallopavo* is essential for turkey genomics and genetics research and the breeding industry.

Results

By adopting the trio-binning approach, we were able to assemble a high-quality chromosome-level F1 assembly and two parental haplotype assemblies, leveraging long-read technologies and genome-wide chromatin interaction data (Hi-C). ~~These assemblies cover~~ From a total of 40 chromosomes (2n=80), we capture 35 chromosomes in a single complete scaffold, and show much improved genome completeness and continuity compared to the old assembly build. The three assemblies are of higher quality than the previous draft quality assembly and comparable to the current chicken assemblies (GRCg6a and GRCg7) shown by the largest contig N50 (26.6 Mb) and comparable BUSCO gene set completeness scores (96-97%). Comparative analyses reveal a large inversion of around 19 Mbp on the Z chromosome not found in other Galliformes. Structural variation between the parent haplotypes were identified ~~in genes involved in growth providing~~which pose potential new target genes for breeding.

Conclusions

We contribute a new high-quality turkey genome at chromosome-level, benefiting turkey genetics and other avian genomics research as well as turkey breeding industry. ~~Collectively, we present a new high quality chromosome level turkey genome, which will significantly contribute to turkey and avian genomics research and benefit the turkey breeding industry~~.

Keywords: Genome assembly, Turkey genomics, trio-binning, animal breeding

Introduction

The domesticated turkey (*Meleagris gallopavo*) is an important agricultural species and the second largest contributor to world poultry production [1]~~[1]~~. The turkey is a member of the Phasianidae family within the order Galliformes. Turkeys and chickens diverged about 25-40 million years ago [2]~~[2]~~. Despite the relative long divergence time, the genome synteny and karyotype of both are highly conserved [3]~~(Griffin et al., 2007)~~. The turkey has 2n=80 compared to the chicken with 2n=78. The turkey karyotype consists of 7 macrochromosomes (>50 Mb), four intermediate chromosomes (>20 Mb, <40 Mb), and the rest being microchromosomes (<20Mb)~~Most of the chromosomes are small microchromosomes, while only a few macrochromosomes are present in the karyotype~~. The turkey karyotype is very similar to the chicken, except that chicken chromosome 2 is homologous to two turkey chromosomes (chromosomes 3 and 6) and chicken chromosome 4 is homologous to turkey chromosomes 4 and 9 [4]~~[4]~~. Zhang et al. (2011) identified a large inversion on the Turkey lineage compared to chicken [5]. In addition, a high degree of synteny has also been observed between the chicken and turkey genomes [6]~~[5]~~.

The first turkey genome assembly (UMD2), published in 2010 [6], was among the first to be done almost exclusively based on second generation sequencing data, and by current standards would be considered of draft quality given the low contig N50 (27.1 kb) and lack of long read sequences [7]~~[5]~~. The authors produced a chromosome level assembly and assembled 30 autosomal and two sex chromosomes. The assembly included linkage data based on a low-density genetic map and the placement of scaffolds to chromosomes relied considerably on conserved synteny assumptions with the better assembled chicken (*Gallus gallus*) genome. However, that version of the chicken genome had many microchromosomes missing altogether or only partially characterized. Avian microchromosomes have proved to be difficult to assemble even today [7]. Reliance on an incomplete chicken genome and the general difficulty in assembling the avian microchromosomes resulted in a

poor representation of microchromosomes in that first UMD2 turkey genome. An updated version of the turkey genome (Turkey_5.1; GCA_000146605.4) has been available since 2019, though it still shows low gene completeness and an incomplete set of microchromosomes.

The problems in characterizing microchromosomes are partly due to sequence characteristics, i.e., high GC and repeat content in microchromosomes, and partly due to their extremely small size and lack of genetic linkage group markers to differentiate the microchromosomes from other chromosomes [7][6]. Hence, Oongoing efforts in producing high quality assemblies of the microchromosomes in avian genomes have been unsuccessful due to multiple causesabove mentioned causes [6].

High quality genome sequences are an essential resource for research and applications in the life sciences. In domestic animal breeding, genome wide marker panels are routinely used to support genomic selection and this significantly accelerates genetic progress [8][7] . An improved genome sequence facilitates ongoing genomic breeding programs. Furthermore, an improved genome assembly will greatly enhance functional interpretation of genomic variation in those breeding populations. For instance, improved annotation of (non)-coding genes benefits the functional interpretation of genome wide association studies (GWAS), and aids in identifying targets for gene editing [9][8].

Currently, more species in the Galliformes have high quality long-read based assemblies, including the Chicken, Japanese quail [10][9], Gunnison sage-grouse [11][10], and the helmeted guineafowl [12][11], allowing for comparative studies within the Galliformes . The genome assemblies of turkey (this paper) and chicken, however, are of considerably higher quality compared to other Galliforme species. This provides opportunities forand an in-depth comparison between the two most important avian agricultural species (chicken and turkey).

Third generation sequencing techniques have made it possible to produce high quality chromosome-based assemblies. The chicken GRCg6a assembly and more recently individual broiler (GRCg7b) and layer (GRCg7w) assemblies have been produced from long read sequencing techniques. The GRCg7 genomes now include (parts of) all microchromosomes. These new chicken assemblies show superior metrics of quality and completeness to previous genome assemblies. In this study we use a relatively new technique, the trio-binning approach, to construct high quality haplotype-resolved turkey assemblies– [13][12]. A similar approach was also applied to create the GRCg7 chicken genome assemblies. In the trio-binning approach, sShort reads from each parent are used to resolve the F1 long reads into groups of long reads belonging to each parent. Each haplotype is then assembled independently resulting in three high quality genome assemblies, one from both parental haplotypes, and one F1 assembly (the primary assembly). This approach is especially powerful to assess structural variation between the parental haplotypes and works well with high heterozygosity rates as this aides in the resolution of the parent haplotypes in the F1 assembly.

In this study our aims were to use the trio-binning approach to produce a chromosome-level turkey assembly (F1), and two parental haplotype assemblies. We further aim to compare the two parental haplotypes to identify structural differences. A good reference genome is essential for many research and commercial applications. In this study we highlight how our new turkey genome can benefit both research and the breeding industry.

Results

Data and assembly of Mgal_WUR_HG_1.0

Three individual turkeys (two parents and one F1) were sequenced using the trio binning approach [13][12]. The two parental animals derive from two distinct commercial lines from the breeding

company Hybrid Turkeys, a Hendrix Genetics company. The F1 animal was sequenced with a depth of 270x using PacBio single-molecule real-time (SMRT) sequencing technology. Approximately 12.25 million subreads were produced with a mean length of 22.5 kb, and N50 read length of 32.5 kb. Reads were assembled using wtdgb2 assembler [14]~~[13]~~ resulting in an initial assembly comprising of 315 contigs with an N50 of 26.68 Mb. The assembly was further scaffolded using Hi-C with HiRise~~ ~~ [15]~~[14]~~. Additional scaffolding was performed using SALSA (with Hi-C) [16] ~~[15]~~ and Redundans~~ ~~ [17]~~[16]~~. The scaffolded assembly was subsequently polished with short reads (three rounds) to produce a final chromosome-level assembly consisting of 151 scaffolds and 232 contigs with a scaffold N50 of 70 Mbp and contig N50 of 26.55 Mbp (**Table 1**). This captures ~~the~~ the majority of the chromosomes ~~chromosome~~ in a single scaffold and chromosome arms in a single contig (**Supplementary File 1: Table S1**). The Hi-C contact map can be found in **Supplementary File 1: Figure S1**.

Haplotype assemblies

As part of the trio-binning approach, both parental haplotypes were assembled with TrioCanu [13]~~[12]~~. We were able to map 110X of the PacBio reads to parent 1 and 137X of the PacBio reads to parent 2, resulting in two parental haplotype assemblies with contig N50 of 9,174,806 bp and 19,855,975 bp for parents 1 and 2, respectively. We performed further scaffolding using LRscaf [18]~~[17]~~ and anchored the assemblies to the F1 assembly using RagTag~~ ~~ [19]~~[18]~~. The QV values indicate high quality and compleness of the assemblies evaluated by Mercury [20] (**Supplementary File 1: Table S2**). The final statistics of the assemblies are shown in **Table 1**.

Formatted: Font: Bold

Table 1: Assembly statistics. Summary statistics for the new Mgal_WU_HG_1.0 and parental assemblies, and comparison with previous turkey assembly (Turkey_5.1) and recent broiler assembly (GRCg7b).

	Mgal_WU_HG_1.0	Turkey_5.1	GRCg7b	Parent 1	Parent 2
**Total sequence length (bp)**	1,001,818,376	1,115,474,681	1,053,332,251	1,051,251,094	1,085,657,715
**Length ungapped (bp)**	1,001,806,830	1,080,180,254	1,049,948,333	1,050,601,018	1,085,166,758
**No. of scaffolds**	151	187,695	214	415	489
**No. of unplaced scaffolds**	115	187,662	172	379	453
**No. of chromosomes**	36	33	42	36	36
**Scaffold N50 (bp)**	70,339,173	3,898,092	90,861,225	71,046,337	71,481,950
**Scaffold L50**	5	80	4	4	4
**No. of contigs**	232	250,220	677	738	675
**Contig N50 (bp)**	26,554,504	27,076	18,834,961	9,174806	19,817,032

**Contig L50**	12	11,318	18	34	13

Assembly accuracy and completeness

The completeness and accuracy of the assemblies were assessed using BUSCO [21][19] and whole-genome alignments. All three assemblies contained over 96% of the expected avian and vertebrate gene sets, comparable to the GRCg6a and GRCg7b chicken genomes and covering 5.4% more gene space compared to the previous turkey genome assembly (Turkey_5.1), as shown in Table 2.

Table 2: Assembly completeness measured in BUSCO scores. Percentage of aligned genes for the vertebrae (n=3354) and avian (n=8338) gene set in the turkey and chicken assemblies.

	**Mgal_WU_HG_1.0**		**Turkey_5.1**		**GRCg7b**		**Parent 1**		**Parent 2**	
	Avian	*Vertebrate*	*Avian*	*Vertebrate*	*Avian*	*Vertebrate*	*Avian*	*Vertebrate*	*Avian*	*Vertebrate*
**Complete**	96.7	96.4	91.3	88.4	97.0	96.5	96.6	96.0	96.8	96.4
**Complete and single-copy**	96.4	95.9	91.1	87.9	96.7	95.7	94.8	93.9	94.1	93.2
**Complete and duplicated**	0.3	0.5	0.2	0.5	0.3	0.8	1.8	2.1	2.7	3.2
**Fragmented**	0.9	1.0	4.1	5.8	0.9	1.2	0.9	1.1	0.9	1.0
**Missing**	2.4	2.6	4.6	5.8	2.1	2.3	2.5	2.9	2.3	2.6

Second, sequence alignments of the F1 assembly were made to the GRCg7b chicken assembly and the Turkey_5.1 assembly (Figure 1). The alignment is highly congruent with the chicken genome (**Figure 1A**), indicating a high degree of conserved synteny. The main exception was a large ~19 Mbp inversion on the Z-chromosome (approximate coordinates ~44,493,000517565-63393669 63,950,000 bp). This inversion was also not present in the previous turkey build, Turkey_5.1, as seen in the alignment (**Figure 1B**). The alignment further shows that in the Turkey_5.1 assembly many contigs were placed in the wrong orientation (resulting in a “zigzag” alignment pattern).

Figure 1: Genome-wide alignment plots. A) Mgal_WU_HG_1.0 aligned with GRCg7b. Alignment shows high structural coherence between both genomes. B) Mgal_WU_HG_1.0 aligned with the old turkey genome build Turkey_5.1. Alignment shows multiple contigs that were placed in the wrong orientation in the previous Turkey_5.1 build.

Repeat and gene annotation

Repeat content

We annotated the repeats using a custom repeat library built using RepeatModeler [22]~~[20]~~. Repeats were found to cover 10.45% of the genome. The most common were LINE elements, covering 6.35% of the genome. Furthermore, 0.76% of bases were DNA transposons, 0.53% long terminal repeats (LTRs), and 1.58% low complexity and simple repeats. The remaining 1.23% of the repeats remained unclassified. A complete overview of the repeats per chromosome is listed in **Supplementary File 2**.

Gene Annotation

The Ensembl annotation pipeline was used to annotate Mgal_WU_HG_1.0 [23]~~[21]~~. The present annotation includes fewer annotated genes compared to Turkey_5.1 and the chicken annotations, but does include more non-coding genes, as shown in Table 3. Hence, the annotation provides a comprehensive overview of the turkey transcriptome with a large increase in transcripts compared to Turkey_5.1 and GRCg6a (Table 3). As expected, microchromosomes show higher gene density compared to macro and intermediate chromosomes ($P<0.00001$, Figure 2). The density generally increases with decreasing microchromosome size.

Table 3: Annotation statistics for the turkey (Mgal_WU_HG_1.0, Turkey_5.1) and chicken (GRCg6a, GRCg7b) genomes. BUSCO scores show percentage of aligned proteins for the avian (n=8338) and vertebrate (n=3354) protein set in the turkey and chicken assemblies.

Annotation	Mgal_WU_HG_1.0	Turkey_5.1	GRCg6a	GRCg7b
Coding genes	16,127	16,226	16,878	17,007
Non-coding genes	7,736	1,585	7,166	13,040
Small non-coding genes	504	543	1,525	1,089
Long non-coding genes	7,228	1,038	5,506	11,946
Misc non-coding genes	4	4	135	5
Pseudogenes	45	159	312	61
Gene transcripts	53,441	30,708	39,288	72,689
Completeness BUSCO (avian / vertebrate)				
% Complete	97.9/97.0	87.5/80.8	95.1/93.8	98.3/97.0
% Fragmented	0.6/1.1	5.2/10.3	2.0/2.9	0.5/1.0
% Missing	1.5/1.9	7.3/8.9	2.9/3.3	1.2/2.0

We identified chicken and Turkey_5.1 homologues of the Mgal_WU_HG_1.0 genes (**Supplementary File 1: Table S3~~1~~**). The majority of the protein-coding genes have a 1:1 orthologue in the Turkey_5.1 (82.4%) or in the GRCg6a (86.3%) genome assemblies. The higher number of genes orthologous to the

Formatted: Font: Bold
Formatted: Font: Not Bold
Formatted Table
Formatted: Font: Not Bold
Formatted: Font: Not Bold
Formatted: Font: Not Bold
Formatted: Font: Not Bold
Formatted: Font: Not Bold
Formatted: Font: Not Bold
Formatted: Font: Not Bold
Formatted: Left
Formatted: Font: Not Bold
Formatted: Font: Not Bold
Formatted: Font: Not Bold

most recent chicken assemblies supports our assertion of a significant improvement of assembly and annotation quality compared to Turkey_5.1

Figure 2: Ideogram showing gene density. A) macro (1-6, Z) and intermediate chromosomes (7,8, 10, 11). B) micro chromosomes (9,12-35) in the Mgal_WU_HG_1.0 genome.

Lineage specific expansion and contraction of protein-coding gene families

OrthoFinder [24][22] was used to infer orthogroups from the following set of bird species - turkey, chicken, Japanese quail (*Coturnix japonica*) [10], helmeted guineafowl (*Numida meleagris*) [12] -and zebra finch (*Taeniopygia guttata*) [25]. From the 16,127 protein-coding genes in the Mgal_WU_HG_1.0 gene set, 98% were found to be in an orthogroup. This was the highest percentage of any of the species tested (**Table 4**). Of the 15,417 orthogroups found, 91% include Mgal_WU_HG_1.0 genes. There are also 10 orthogroups that contain only Mgal_WU_HG_1.0 genes, of which two have homologs in the nr database (*MANBAL* , and *POL3*) (**Supplementary File 1: Table S42**).

Table 4: Number of orthogroups found and proportion of genes assigned to each orthogroup per species. Species included: turkey (Mgal_WU_HG_1.0, Turkey_5.1), chicken (GRCg6a, GRCg7b), Japanese quail (Coturnix_japonica_2.0), helmeted guineafowl (NumMel1.0), and zebra finch (bTaeGut1_v1.p)

Species assembly	Mgal_WU_HG_1.0	Turkey_5.1	GRCg6a	GRCg7b	Coturnix_japonica_2.0	NumMel1.0	bTaeGut1_v1.p
No genes	16127	16226	16878	17007	15732	15661	16619
No genes in orthogroups	15843	15365	16359	16583	15342	15306	15971
No unassigned genes	284	861	519	424	390	355	648
Genes in orthogroups (%)	98.2	94.7	96.9	97.5	97.5	97.7	96.1
Unassigned genes (%)	1.8	5.3	3.1	2.5	2.5	2.3	3.9
No orthogroups containing species	14033	13350	13800	14156	13801	13695	13390
Orthogroups containing species (%)	91	86.6	89.5	91.8	89.5	88.8	86.9
No species-specific orthogroups	10	63	23	24	4	7	110
No genes in species-specific orthogroups	50	178	120	95	9	67	428
Genes in species-specific orthogroups (%)	0.3	1.1	0.7	0.6	0.1	0.4	2.6

Gene family cContractions and expansions in orthologous groups

While most orthogroups studied showed no change in the copy-number of protein coding genes, 71~~29~~ groups showed expansions or contractions of gene families predicted using CAFE5 software [26] (61~~27~~ expansions, ~~2~~10 contractions) (**Supplementary File 3~~2~~**). Expanded orthogroups contained proteins involved in important processes in bird development and growth, including gene families involved in cytoskeleton (proteins for feather keratin) (OG00000026~~1~~, OG0000030~~, OG0000048~~), reproduction (involved in spermatogenesis/spermiogenesis) (OG0000005~~, OG0000883, OG0000065, OG0000973~~), response to stress (~~OG0000980~~OG0000111), and immunity (OG0000001~~, OG0000127, OG0001025~~). Orthogroups OG0000005 ~~and OG0000883 show~~shows an expansion of the turkey PHD finger protein 7 (PHF7) gene, which has been shown to be a highly duplicated gene family in the chicken genome [27]~~[23]~~. The contracted gene families include one immunoglobin (OG0000001), a homeobox B8 (OG0000526) gene family, and an olfactory receptor gene family (OG0000407) ~~and one inositol receptor like protein (OG0000070)~~.

Structural variation between parental haplotypes

The F1 and parental short reads were mapped back to the corresponding assembly with the percentage of mapped reads ranging from 98.73 - 98.91%. Heterozygosity in the F1 assembly was 0.173% (1 heterozygous SNP per 577 bp), while for the ~~parent assemblies~~paternal and maternal genomes lower heterozygosity of 0.117% (parent 1) and 0.107% (parent 2) were found, respectively. This shows that both parental lines generally have low heterozygosity, resulting in a rather low heterozygosity in the F1 as well.

Structural variation

The F1 and ~~parent assemblies~~the paternal haplotypes are completely co-linear **Supplementary File 1: Figure S2**). There are no large structural differences (>1 Mbps) between the two parental haplotypes except for a 1.47 Mbp inversion on chromosome 1 (74.28 – 75.74 Mb, **Supplementary File 4~~3~~**)

comprising 25 protein coding genes and 15 lncRNA genes. **Table 3** shows an overview of the number and cumulative length of each type of structural variation.

Table 5: Structural variation between the two parental haplotype assemblies. The parent 1 assembly was used as reference and the parent 2 assembly used as the query. Copygain: Copy gain in the query genome, copyloss: copy loss in the query genome

Variation type	Count	Length Parent1	Length Parent2
Syntenic regions	85	990,480,776	989,217,672
Inversions	19	1,728,932	1,525,862
Translocations	68	895,801	867,550
Duplications	397	870,354	3,179,922
Copy gains	40	-	305,148
Copy losses	58	1,268,056	-

In total, 231 large structural variations (>10 kb) have been identified between the two parental haplotypes (**Supplementary File 54**). From these, 81 affect the coding sequence of protein coding genes (**Supplementary File 4**), of which 40 have a 1:1 ortholog in chicken. Interestingly, an inversion affecting the coding sequence of the *BLB2* gene, this gene is (duplicated within MHC-B region in chicken playing a crucial role in disease resistance or susceptibility [28]associated with obesity in mice [24]) was found to be homozygous in parent 2 compared to parent 1 (**Supplementary File 1: Figure S3**).2 and heterozygous in parent 1. We further identified duplications in the parent 2 haplotype comprising the *TRIM36*, *GRIA2* and *MAN2B2* gene. Specifically, the parent 2 haplotype exhibits a 20 kb duplication of the 3' end of *MAN2B2* (**Supplementary File 1: Figure S4**), a gene which in pigs is associated with ovulation rate [29][25]. In addition, a 34 Kbp duplication affecting the *GEMIN8* gene in parent 12 was identified (**Supplementary File 1: Figure S5**). The *GEMIN8* gene product is part of the survival motor neuron (SMN) complex. Moreover, a 53 Kbp duplication was found affecting the 3' end of the *RIMKLB* gene (**Supplementary File 1: Figure S6**), resulting in a copy number of 3 in parent 1 but a copy number of >520 in parent 2. In addition, a 100 kb translocation that comprises the *RALYL* gene was identified. The translocated region is found at around 68.2 Mbp on chromosome 5 in parent 1, while it is found at a position around 90.1 Mbp on the same chromosome in parent 2. Finally, an inversion on chromosome 30 of length 187 kb comprises two protein coding genes and one lncRNA.

A full overview of structural variation between the parental haplotypes is provided in **Supplementary File 54.**

Loss of function variation

The most common effect of selection is to alter gene expression, leading to phenotypic changes. However, a small proportion of phenotypic variation is due to impaired gene functioning [30][26]. We assessed the presence of loss-of-function variation (LoF), specifically stop-gained variants affecting genes in either of the two parental haplotypes (**Supplementary File 65**). In total, 138 stop-gained variants affecting 92 genes between the parent1 and parent2 haplotypes were identified. Genes carrying LoF mutations that are especially noteworthy include the *RYR2* gene, which is affected by four LoF variant in parent 2, likely leading to an impaired RYR2 protein. Mutations in the *RYR2* gene are associated with sudden death syndrome in broiler chickens [31][27]. A second gene worth highlighting is *LRRC41* which, in the parent 2 haplotype, contains a stop-gained variant. Knockouts of this gene lead to increased lean body mass in mice and hence this gene poses an interesting candidate for selection for body weight in turkey [32].

Mapping of SNP-chip markers

SNP-chips are useful to study variation (single nucleotide polymorphisms, SNPs) between individuals and are widely applied in genomic selection. We mapped SNP-chip markers from a 65K SNP array (64,800 SNPs; Illumina, Inc.) to Mgal_WU_HG_1.0 (**Supplementary File 1: Table S53**) using a custom SNP mapping pipeline (see methods). We mapped 64,536 (99.4%) of the markers to Mgal_WU_HG_1.0. From these, 1,532 markers that were located on unplaced contigs in Turkey_5.1 are now mapped to specific chromosomes in Mgal_WU_HG_1.0, and 415 markers were placed on the new chromosomes 31-35, indicating a higher completeness. More specifically, we were able to place a significant number of new markers, especially on chromosomes 1 (412), 27 (120), 31 (192), and Z (594).

Distinct genomic landscapes of turkey micro and macrochromosomes

Avian genomes are known to vary greatly in genomic features, especially between the micro and macrochromosomes [33]. We evaluated the genomic landscape of the turkey chromosomes in terms of repeat content, gene density, and gene expression between macro (>40 Mbp), intermediate (>40 Mbp, <20 Mbp), and micro (<20 Mbp) chromosomes. We found that the repeat content of each repeat class in macro, micro and intermediate chromosomes varied highly along the chromosome (**Supplementary File 1: Figures S7~~3~~-S14, Supplementary File 2~~0~~**). Macrochromosomes are enriched for DNA transposons ($p<0.01$) and LINE elements ($p=0.0281$) compared to the intermediate and microchromosomes (**Supplementary File 1: Figure S7~~3~~-S8~~4~~**). In addition, LINE CR1 elements are especially enriched at the tails of macrochromosomes. Microchromosomes are enriched for low complexity ($p<0.01$, **Supplementary File 1: Figure S9~~5~~**), simple ($p<0.01$, **Supplementary File 1: Figure S11~~7~~**), and unknown repeats ($p=0.062$, **Supplementary File 1: Figure S14~~0~~**) compared to intermediate and macrochromosomes, the latter especially at the tails of the chromosomes.

In order to assess whether there was a distinction between the type of genes (e.g. tissue specific or housekeeping) in chromosome types, we analysed RNA-seq datasets from 16 tissues (mapping rates in **Supplementary File 1: Table S6~~4~~**). Microchromosomes showed on average higher gene expression than macro and intermediate chromosomes (**Figure 3A**), as well as having a higher relative abundance of housekeeping genes, defined here as genes expressed in at least 13 out of the 16 studied tissues included in this study (**Figure 3B**).

Figure 3: A) Overview of gene expression in macro, intermediate and micro chromosomes. B) Relative abundance of tissue specific genes in each chromosome class. Microchromosomes show higher relative abundance of housekeeping genes when compared with macro and intermediate chromosomes. Number of tissues tested: 16. Housekeeping genes:

expressed in at least 13 tissues; less specific genes: expressed in at least 5 tissues and fewer than 13 tissues; specific: expressed in 2 to 5 tissues; more specific: expressed in one or two tissues.

Conserved synteny within the Galliformes clade

We performed synteny analysis to assess chromosomal and structural rearrangements within a wide range of avian species. Four Galliformes were included: turkey, chicken, Japanese quail, and helmeted guineafowl. Furthermore, two Passeriformes, zebra finch and great tit, and emu, a species from the Casuariiformes order were included. The multi-species synteny plot shows a high degree of synteny between the avian species both on the macro and the microchromosomes, despite the large evolutionary distances (**Figure 4**), supported by recent findings [34, 35]~~[34]~~.

Figure 4: Chromosomal rearrangements across several avian species. Pairwise synteny comparison across 7 birds shows several chromosomal rearrangements. Grey segments represent conserved synteny. Species: turkey (*Meleagris gallopavo*), chicken (*Gallus gallus*), Japanese quail (*Coturnix japonica*), helmeted guineafowl (*Numida meleagris*), great tit (*Parus major*), zebra finch (*Taeniopygia guttata*), and emu (*Dromaius novaehollandiae*).

Of all chromosomes, it is evident that especially the Z chromosome has been prone to large chromosomal rearrangements between avian orders (**Figure 5**) [36]. Interestingly, we found a large inversion of around 19 Mbp on the turkey Z chromosome not found in the other Galliformes and songbirds [5] (**Supplementary File 1: Figure S15**). The inversion was supported by a normal alignment at the approximate breakpoints (**Supplementary File 1: Table S7 - Figure S16**) and by the HiC contact map (**Supplementary File 1: Figure S17**). This is especially striking since rearrangements on the Z chromosome are uncommon within the Galliformes. One region at the tail of the chicken Z chromosome lacks synteny with other Galliformes altogether [37]. This region is enriched in repeat sequences in both chicken and turkey (**Supplementary File 1: Figure S18~~1~~**), as described previously in Bellott et al 2020 [37].

Figure 5: Chromosome Z rearrangements across 7 avian species. Pairwise synteny comparison of the Z chromosome across avian species reveals a large inversion in turkey. Grey segments represent conserved synteny. Species: turkey (*Meleagris gallopavo*), chicken (*Gallus gallus*), Japanese quail (*Coturnix japonica*), helmeted guineafowl (*Numida meleagris*), great tit (*Parus major*), zebra finch (*Taeniopygia guttata*), and emu (*Dromaius novaehollandiae*)

Discussion

We present a new, chromosome-level, high quality reference assembly for *Meleagris gallopavo*, Mgal_WU_HG_1.0. The trio binning approach has been proven to be a robust method to characterize the two haplotypes of F1 individuals~~-~~ [13]~~[12]~~. The ~~quality of the assemblies~~chromosome level assembly (**Supplementary File 1: Table S1**) presented in this study confirms the value of this method in not only providing a quality assembly but also in uncovering structural genomic variation. The Mgal_WU_HG_1.0 assembly is a large improvement over the previous turkey assembly, Turkey_5.1 [6]~~[5]~~. The assembly is now comparable in quality and completeness to the chicken reference genome (GRCg6a) and to the recently available GRCg7 genomes.~~-~~ Note that we sequenced a male animal and we are therefore lacking the W chromosome. One major limitation of previous turkey assemblies was that they relied on assumptions on high turkey-chicken retained synteny to achieve a chromosome-level assembly. Such assumptions can result in bias, especially when comparing turkey to chicken. Mgal_WU_HG_1.0 does not rely on such comparisons.

Formatted: Font: Bold

Combining long reads and genome-wide chromatin interaction data (Hi-C) enables the capture of chromosome arms in a single contig, resulting in a highly continuous and contiguous chromosome-level assembly. Furthermore, long reads can span long repetitive regions including DNA transposons and LINE elements, as well as large structural variants. Centromeres, however, are too long to traverse reliably in most cases. Thanks to these recent sequencing technologies, we are able to correct a number of wrongly oriented contigs in Turkey_5.1, a phenomenon often observed in short-read based

assemblies. The improvements in genome quality, completeness and continuity allow for a more thorough annotation of repeats and gene models. The increase in complete BUSCO genes in Mgal_WU_HG_1.0, compared to Turkey_5.1, indicates a much-improved gene space in the current genome assembly, comparable to the latest chicken genome builds.

Improving genome assemblies improves all analyses that depend on them. One of the reasons to improve the turkey assembly was to better map SNP-chip markers to the genome. SNP-chips are widely used in genomic selection and a better genome representation and gene annotation directly impacts its use for breeding. Specifically, the new turkey genome build overcomes the lack of SNPs mapped to gene-dense microchromosomes, as 85.3% of the SNP markers previously mapped to unplaced scaffolds on Turkey_5.1 are now mapped to chromosomes on Mgal_WU_HG_1.0, especially improving the representation of microchromosomes 31 to 35.

Turkey breeding is done on pure elite lines which can be selected for different purposes. In our study, one parent was from a female breeding line, with more focus on egg production and conformation, whereas the other parent was from a male breeding line focussing on growth and production traits. In producing a commercial product, lines are crossed to produce hybrid offspring that shows the benefit of the breeding goals of both parental lines. In addition, the hybrid offspring benefits from hybrid vigour, resulting from two relatively differentiated lines. For the trio-binning method, having parents that are genetically distinct helps in resolving the haplotypes. Nevertheless, in this study, we present two high quality parental haplotype assemblies where the low heterozygosity of the parents presented no obstacle to resolving the parental haplotypes.

Among the remaining challenges in variation analysis is the characterization of structural variants. The challenge is two-fold. First, these large-scale variants are often not robustly detected using short-read sequencing. Second, individuals usually have sequence that is population specific, and which may not

be present in a reference assembly. This can make such large insertions hard to characterize, even by re-sequencing. In the process of assembling Mgal_WU_HG_1.0 we now have reference assemblies for two distinct breeding lines, which should greatly aid in variation analysis. Even though such large structural variants appear to be uncommon between breeding lines, we demonstrate how genes potentially important in breeding may be affected. These genes can be further prioritized in routine genomic breeding practice.

As more genomes are characterized with high accuracy and at a chromosome-level, comparative genomics is increasingly used to study the function of genes and variants, including copy number variants. The new Mgal_WU_HG_1.0 genome assembly was applied to identify orthogroups that have expanded or contracted in turkey compared to other avian species. Expanded orthogroups included various distinct keratin families, encoding major structural proteins of feathers and claws [38]~~[28]~~. One gene family comprising the PHD Finger Protein 7 (PHF7) was significantly expanded in turkey. *PHF7* acts during spermiogenesis for histone-to-histone protamine exchange and is a determinant of male fertility in Drosophila and mouse [39]~~[29]~~, and highly expressed in rooster testis [40]~~[30]~~. This gene family was found to be expanded in chicken as well, with distinct gene clusters on five chromosomes [27]~~[23]~~. In addition, genes related to immunity and response to stress are expanded in turkey. Further research is needed to disentangle the exact function of these complex gene families.

A characteristic of avian genomes is that they comprise a huge range of chromosome sizes. Interestingly, bird genome organization may be ancestral to all vertebrates [41]~~[31]~~. Among the peculiar outcomes is a wide range in e.g. recombination rates, GC-bias, gene densities and variation density throughout the genome [33]~~[32]~~. The distinct nature of these features is particularly difficult to study in microchromosomes as they have proven so difficult to characterize. The distinct patters of both gene density and repeat content between the macro and microchromosomes have been described previously by Kapusta et al. 2017 [42]. The Mgal_WU_HG_1.0 assembly though, has a better

representation of the microchromosomes, allowing a better understanding of functional aspects of genes and other genome elements. We have shown that the microchromosomes have a unique repeat landscape enriched for low complexity, simple, and unknown repeats, especially at the tails of the chromosomes. Together these efforts provide new insights in microchromosome composition and evolution.

Bird genomes have very high retained synteny [43]~~[33]~~. This pattern was confirmed in our analysis of the conserved synteny between several Galliformes (turkey, chicken, Japanese quail, helmeted guineafowl) and three outgroups (zebra finch, great tit, emu). Despite the long divergence time that separates turkey and chicken [2]~~[2]~~, both species have relatively similar karyotypes confirmed by the high structural continuity and relatively little rearrangements between the two birds, even in the microchromosomes. The latter is noteworthy because of the very high recombination rates generally observed in microchromosomes [44]~~[34]~~, which would suggest that a higher rate of chromosomal rearrangements might be expected but is not observed. Expanding observations to other Galliformes suggest similar degrees of conserved synteny, although comparisons for micro-chromosomes are less accurate due to the more incomplete assembly of these other Galliform species

The Z chromosome presents a moderate yet striking deviation from the observed evolutionary stability. This chromosome exhibits a few rearrangements within the Galliformes and, in line with the findings of Zhang et al. (2011), we observed and validated a large inversion in the turkey Z chromosome [5]. As with the Mgal_WU_HG_1.0 assembly the exact breakpoints of this 19 Mbp inversion on the Z chromosome can now be pinpointed. This inversion is unique for the turkey lineage, and not found in any of the other Galliformes.

In conclusion, the new turkey genome here presented (Mgal_WU_HG_1.0) (and the two parental haplotype assemblies) represents a substantial improvement over the previous assembly and is an important resource with many applications in research and in the turkey breeding industry.

Methods

Data and Assembly

To create a high-quality chromosome level genome assembly of *Meleagris gallopavo*, three individuals were sequenced using the trio binning approach - two parents and one F1. The two parents come from two distinct commercial lines from Hendrix Genetics, one male line (parent1) and one female line (parent2). The F1 turkey was sequenced by Dovetail Genomics using PacBio single-molecule real-time (SMRT) sequencing technology (PacBio Sequel System, RRID:SCR_017989) with a total depth of 270X. We generated short read sequencing data from the F1 (90.4X coverage) and both parents (35.4X, and 39.7X coverage) on an Illumina HiSeq 4000 (HiSeq 4000 System, RRID:SCR_016386). In addition, Hi-C data was generated with a coverage of 32X. An initial assembly was created by Dovetail Genomics using wtdgb2 (WTDBG, RRID:SCR_017225) [14]~~[13]~~, polished with the PacBio long reads using wtpoa-cns, and scaffolded using the Dovetail *De Novo* Assembly Process, which uses Chicago® and Dovetail Hi-C proximation ligation methods and the HiRise™ scaffolder as described in [15]~~[14]~~.

Polishing

Pilon v1.23 (Pilon, RRID:SCR_014731) [45] ~~[36]~~ was used to polish SNPs and indels based on the short Illumina reads from the F1 (twice with parameters--diploid --mindepth 0.7 --fix bases --changes), and indels with the Illumina reads from parent2 because of the higher coverage compared to parent1 (--fix indels).

Scaffolding

We scaffolded the F1 assembly received by Dovetail Genomics using the Hi-C reads and the PacBio long reads, both from the F1. The Hi-C reads were mapped to the polished assembly based on the Arima Mapping pipeline [46]~~[37]~~, using BWA-MEM v0.7.17 (BWA, RRID:SCR_010910) [47] ~~[38]~~ with default parameters. The filter_five_end.pl script was used to filter and keep the 5'-end. After filtering, the reads are sorted and paired using the two_read_bam_combiner.pl script. This results in a sorted, paired-end BAM file that has been filtered by mapping quality (mapping quality filter =10). Picard Tools v2.23.4 (Picard, RRID:SCR_006525) [48] ~~[39]~~ - AddOrReplaceReadGroups and MarkDuplicates was used to add a read group and remove duplicates. The mapped Hi-C reads were used to scaffold the assembly with SALSA v2.2 (SALSA, RRID:SCR_022013) [16]~~[15]~~, which is a scaffolder that uses long range contact information (Hi-C) with parameters -e "GATC". Redundans v0.14a [17]~~[40]~~ was used to scaffold the assembly with the PacBio reads with length >40 Kbp and remove redundant contigs from the final assembly. The parameters -l <long reads> --nogaplosing --noscaffolding were used (--noscaffolding skips short read scaffolding). QV values are calculated using Merqury [20].

Hi-C validation - mis-assemblies

To validate our F1 assembly and look for mis-assemblies we used Hi-C contact maps.

Juicer v1.6 (Juicer, RRID:SCR_017226) [49] ~~[41]~~ was used to generate Hi-C contact maps from the Hi-C reads (**Supplementary File 1: Figure S1**) and 3D-DNA v180922, a 3D de novo assembly pipeline (3D de novo assembly, RRID:SCR_017227) ~~[42]~~, to scaffold our assembly. Juicebox v1.11.08 (Juicebox, RRID:SCR_021172) [50] ~~[43]~~ was used to visualize the Hi-C contact map and identify mis-assemblies. Each breakpoint in the macrochromosomes was manually checked with Juicebox and JBrowse 1.16.9 (JBrowse, RRID:SCR_001004) [51] ~~[44]~~ to visualize the PacBio read coverage at the breakpoints.

Haplotype assemblies using trio-binning

TrioCanu (a module from the Canu assembler, v2.1.1) (Canu, RRID:SCR_015880) [13][12] was used to bin the parental reads to construct parental haplotype assemblies. TrioCanu was run with the short reads from each parent and the F1 PacBio reads with the following options: -p asm genomesize-1.1g. The corrected reads from TrioCanu were mapped to the Triocanu assembly with Minimap2 v2.17-r941 (Minimap2, RRID:SCR_018550) [52][45], options -x map-pb (mapping PacBio). LRScaff v1.1.10 [18][17] was used to scaffold each parent assembly. For both parents the scaffolding was done with these parameters: min_contig_length = 500, identity = 1, min_overlap_length = 400, max_overhang_length = 500, max_end_length = 500, min_supported_links = 2, iqr_time = 3. Duplicated sequences were removed using seqkit.

RagTag v1.1.1 [19][46] was used for reference-guided scaffolding of each parental assembly, using the F1 assembly as reference. The scaffold module from RagTag was used with default parameters.

Field Code Changed

Formatted: English (United States)

Completeness

BUSCO

BUSCO v4.1.2 (BUSCO, RRID:SCR_015008) [21] [19] was run to assess the completeness of the assembly in terms of gene space. BUSCO was run in the genome mode (-m genome) and with the vertebrae (vertebrata_odb10) and aves (aves_odb10) datasets (using the flag -l <dataset>).

Genome comparison - alignment

Genome assembly alignments were generated using D-GENIES v1.3.0 (D-GENIES, RRID:SCR_018967) [53][47], using minimap2 as the aligner. The chromosomes were sorted on length, and noise (short repeat alignments) was removed from the alignment plot.

Structural variation (parents)

Structural variation between the two parental haplotypes was discovered using SyRI v1.5.4 [54][48]. First, we aligned the two haplotype assemblies using minimap2 with settings -ax asm5 –eqx. Next, we

used SyRI to identify structural variation using the minimap2 alignment. Results were plotted using plotsr tool v0.5.3 [55][49]. Large structural variants were manually validated in JBrowse 1.16.9 [51][44].

Remapping and variant calling

The short Illumina reads from the F1 individual were mapped back to the assembly using BWA-MEM v0.7.17 (BWA, RRID:SCR_010910) [47][38]. Samblaster v0.1.26 (SAMBLASTER, RRID:SCR_000468) [56] [50] was used to mark duplicates and Samtools v1.14 (SAMTOOLS, RRID:SCR_002105) [57] [51] to sort and index the BAM files. Freebayes v1.3.1 (FreeBayes, RRID:SCR_010761) [58] [52] was used for variant calling with: --use-best-n-alleles 4 --min-base-quality 10 --min-alternate-fraction 0.2 --haplotype-length 0 --ploidy 2 --min-alternate-count 2. The vcffilter module from vcflib v0.00.2019.07.10 [59][53] was used to discard variants with low phred quality score (<20). Tabix, a module from htslib v1.9 (SAMTOOLS, RRID:SCR_002105) [59] [54] was used to index the VCF files. The stats module from BCFtools v1.9 (SAMtools/BCFtools, RRID:SCR_005227) [60] [55] was used to compute summary statistics of the variant calling. The same process was followed to call variants for each parent. Alignment quality control statistics were computed with QualimMap v.2.2.2-dev (QualiMap, RRID:SCR_001209) [61][56].

SNP-Chip

In order to map SNP markers from the 65K single nucleotide polymorphism (SNP) array (65,000 SNP; Illumina, Inc.) to the new genome build we first aligned the two genome builds (Turkey_5.1 and Mgal_WU_HG_1.0) using nucmer v4.0.0rc1 (MUMmer, RRID:SCR_018171) [62][57]. Next we converted the delta file to a chain file using mugsy v1.2.3 delta2maf and maf-convert (Mugsy, RRID:SCR_001414) [63][58]. We used CrossMap v0.6.1 (CrossMap, RRID:SCR_001173) [59] to identify SNP locations on the query Mgal_WU_HG_1.0 assembly. We further performed a blastn v2.11.0+

search (BLASTN, RRID:SCR_001598) [64] [60] to identify the locations of SNPs that could not be mapped from the previous build using the SNPs probe sequences.

Annotation and repeats

The genome was annotated with the ENSEMBL annotation pipeline and is available as part of the Ensembl Rapid Release (Ensembl, RRID:SCR_002344) [23][21]. The transcriptome and proteome evidence used in the annotation are listed in **Supplementary File 76**. We used a custom python script to query the Ensembl rapid release homologue gene page to identify Turkey_5.1 and GRCg6a homologues of all the Mgal_WU_HG_1.0 genes. The BuildDatabase tool from RepeatModeler v1.0.11 (RepeatModeler, RRID:SCR_015027) [22] [20] was used to build a de novo repeat library from our assembly using the Recon and RepeatScout tools. RepeatMasker v4.0.7 (RepeatMasker, RRID:SCR_012954) [65] [61] was used to identify repeats together with the custom build repeat library from RepeatModeler.

Orthologues

The proteomes of five bird species were used to infer orthogroups (option -og) using OrthoFinder v2.5.4 (OrthoFinder, RRID:SCR_017118) [66][62]. The proteomes of the following assemblies were downloaded from Ensembl release 106: turkey - Turkey_5.1; chicken - GRCg6a; Japanese quail - Coturnix_japonica_2.0; helmeted guineafowl - NumMel1.0; zebra finch - bTaeGut1_v1.p. The proteomes for Mgal_WU_HG_1.0 (turkey) and GRCg7b (chicken) were downloaded from the Ensembl rapid release (March 2022). For each orthogroup, the protein isoform with the best alignment based on species similarity, score and expect value was chosen. Turkey-specific orthogroups were analysed by running BLASTp v2.11.0+ (BLASTP, RRID:SCR_001010) [64] [60] against the NR database to identify homologous genes from a wider range of species.

Gene family contractions and expansions of protein-coding gene families

Expansions and contractions of protein-coding gene families were assessed by ~~filtering the OrthoFinder results.~~CAFE5 [26]. ~~If the number of proteins for those species is significantly higher than the number of turkey proteins, we consider a contraction in turkey. On the other hand, if the number of proteins in the non turkey species is significantly lower than the number of turkey genes we consider an expansion in turkey.~~The phylogenetic tree was obtained using the BirdTree database [67].

Distinct genomic landscapes of turkey micro- and macrochromosomes

To better understand the differences between macro (>40 Mbp), intermediate (>40 Mbp, <20 Mbp), and micro (<20 Mbp) chromosomes, we investigated repeat content, gene structure and gene expression. A welch t-test was used to test for difference of repeat content and families between macro- intermediate and microchromosomes.

Repeats

A custom repeat library created with RepeatModeler and custom R scripts were used to investigate the differences in repeat content between macro, intermediate and microchromosomes. Each chromosome was split into bins (each bin corresponding to 2% of the chromosome length), allowing us to compare the chromosomes by relative length. We calculated the average repeat content in each bin. An ideogram of the density of each repeat feature was created for macro, intermediate and microchromosomes with the R v4.0.2 (R Project for Statistical Computing, RRID:SCR_001905) [68] ~~[63]~~ package RIdeogram v0.2.2 [69]~~[64]~~. RIdeogram calculates feature density in sliding windows (100 Kbp for macro and intermediate chromosomes, 50 Kbp for microchromosomes).

Tissue specificity

Expression data for 16 turkey tissues (jejunum, proventriculus, thigh, testis, ileum, pancreas, spleen, breast, brain, heart, thymus, liver, gizzard, duodenum, caecal tonsil, bursa) from a male individual at three developmental stages (14, 21, 28 days post hatch) was downloaded from Bioproject

PRJNA259229. Not all tissues were available at all stages: testis was not available at day 21 and caecal tonsil at day 28. HISAT2 v2.2.1 (HISAT2, RRID:SCR_015530) [70] [65] was used to index the assembly (hisat2-build), and align the RNA-seq reads to the assembly. Stringtie v2.1.7 (StringTie, RRID:SCR_016323) [71] [66] was used to assemble transcripts using the aligned reads and Ensembl gene annotation with options -A and -B. A non-redundant set of transcripts was generated with Stringtie's merge option (--merge), which creates a unified set of transcripts from several samples. Stringtie was run once more, now using this new set of transcripts as the reference annotation file. The resulting table containing the gene abundance of all genes was used in our analysis. We analysed the results through custom R (v4.0.2) scripts. We started by filtering the gene abundance table to keep only the genes that are expressed (FPKM >1). Then we classified genes into housekeeping (expressed in at least 13 tissues), less specific (expressed in at least 5 and in fewer than 13 tissues), specific (expressed in 2 to 5 tissues), and more specific genes (expressed in one or two tissues). The relative abundance of housekeeping/specific genes was calculated by counting the number of genes in these categories in macro, intermediate and microchromosomes and dividing that by the total amount of genes in each chromosome type.

Gene structure

We used RIdeogram v0.2.2 [69][64] and R (v 4.0.2) to compare the gene density between the chromosome classes. RIdeogram calculates gene density in sliding windows, 100 Kbp for macro and intermediate chromosomes, 50 Kbp for microchromosomes. Gene density per megabase was calculated by dividing the number of annotated genes on a chromosome by its length. A welch t-test was used to test for difference of gene densities between macro- intermediate and microchromosomes.

Synteny

The MCscan python pipeline from the JCVI utility libraries v1.1.11 (MCScan, RRID:SCR_017650) [72] [67] was used study chromosomal rearrangements between several bird species: Turkey (*Meleagris gallopavo*), chicken (*Gallus gallus*), Japanese quail (*Coturnix japonica*), helmeted guineafowl (*Numida meleagris*), great tit (*Parus major*), zebra finch (*Taeniopygia guttata*), and emu (*Dromaius novaehollandiae*).

The genome (fasta coding DNA sequence, CDS) and annotation files for these species were obtained from Ensembl release 106. The files for Mgal_WU_HG_1.0 and GRCg7b were obtained from the Ensembl rapid release (April 2022). The annotation file for the emu assembly ZJU1.0 was shared with us from [73][68]. This annotation file, in combination with the FASTA file obtained from NCBI was used to create the CDS fasta file necessary for the pipeline.

We started by trimming the accession IDs in the FASTA file and converting the GFF3 annotation file to BED format. The jcvi.compara.catalog ortholog and jcvi.compara.synteny screen (with parameters --simple) were used to create the necessary input files for plotting. The synteny plots were created with jcvi.graphics.karyotype using parameter --basepair. To validate the chromosome Z inversion, first, we manually checked the inversion breakpoints (reads spanning) using JBrowse 1.16.9.

Data Availability

The genome assemblies and sequencing data have been deposited in ENA under Bioproject accession PRJEB42643. The turkey genome and annotations are available through ENSEML Rapid Release (https://rapid.ensembl.org/Meleagris_gallopavo_GCA_905368555.1/).

Supplementary Files

Supplementary File 1: Table S1: Genome assembly and annotation overview.

Formatted: Font: Not Italic

Supplementary File 1: Table S2: QV values indicating assembly quality and completeness.

Supplementary File 1: Table S3: Protein homology between Mgal_WU_HG_1.0, Turkey_5.1 and chicken (GRCg6a).

Supplementary File 1: Table S42: Blast results of proteins in Mgal_WU_HG_1.0 specific orthogroups.

Supplementary File 1: Table S53: Mapping of 65K markers on Mgal5.1 and Mgal_WU_HG_1.0.

Supplementary File 1: Table S64: Mapping rate of RNA-seq datasets from 16 tissues to Mgal_WU_HG_1.0. Tissues (jejunum, proventriculus, thigh, testis, ileum, pancreas, spleen, breast, brain, heart, thymus, liver, gizzard, duodenum, caecal tonsil, bursa) are from a male individual at three developmental stages (14, 21, 28 days post hatch).

Supplementary File 1: Table S6: Mummer alignment between Turkey5.1 and Mgal_WUR_HG_1.0 of the first and the second breakpoint of the 19.4 Mbp inversion on the Z-chromosome.

Supplementary File 1: Figure S1: Hi-C contact map of the Mgal_WU_HG_1.0 assembly.

Supplementary File 1: Figure S2: Parent 1 vs. parent 2 alignment.

Supplementary File 1: Figure S3: Inversion comprising the start of the BLB2 gene in parent2 compared to the parent1 haplotype.

Supplementary File 1: Figure S4: Duplication affecting the tail of the MAN2B2 gene in parent2 compared to the parent1 haplotype.

Supplementary File 1: Figure S5: Duplication affecting GEMIN8 gene in parent2 compared to the parent1 haplotype.

Supplementary File 1: Figure S6: Duplication affecting RIMKLB gene with higher copy number in parent 2 compared to parent1 haplotype.

Formatted: English (United States)

Supplementary File 1: Figure S7~~3~~: Average DNA repeat content along the chromosomes for macro, intermediate and microcromosomes.

Supplementary File 1: Figure S8~~4~~: Average LINE repeat content along the chromosomes for macro, intermediate and microcromosomes.

Supplementary File 1: Figure S9~~5~~: Average low complexity repeat content along the chromosomes for macro, intermediate and microcromosomes.

Supplementary File 1: Figure S10~~6~~: Average LTR repeat content along the chromosomes for macro, intermediate and microcromosomes.

Supplementary File 1: Figure S11~~7~~: Average simple repeat content along the chromosomes for macro, intermediate and microcromosomes.

Supplementary File 1: Figure S12~~8~~: Average SINE repeat content along the chromosomes for macro, intermediate and microcromosomes.

Supplementary File 1: Figure S13~~9~~: Average snRNA repeat content along the chromosomes for macro, intermediate and microcromosomes.

Supplementary File 1: Figure S14~~0~~: Average unknown repeat content along the chromosomes for macro, intermediate and microcromosomes.

Supplementary File 1: Figure S15: Chromosome Z alignment showing inversion with GRCg7b (A) and Turkey5.1 (B).

Supplementary File 1: Figure S16: Alignment of corrected pacbio reads at the approximate breakpoints of the ~19.4 Mbp inversion on the Z-chromosome.

Supplementary File 1: Figure S17: HiC contact map of the Z chromosome.

Supplementary File 1: Figure S18~~1~~: Schematic view of Gal7b chromosome Z and representation of several biotypes of genes and genomic features (Ensembl, rapid release 15th June 2022, accessed on 27th June 2022).

Supplementary File 2: Repeat annotation.

Formatted: Font: Italic

Supplementary File 3~~2~~: Gene family expansions and contractions.

Supplementary File 4~~3~~: Syri output showing structural variation between the two parent haplotypes.

Supplementary File 5~~4~~: Structural variation ~~table~~ between parent haplotypes.

Supplementary File 6~~5~~: Stop-gained variants identified in either or one of the two parent ~~assemblies~~haplotypes.

~~*Supplementary File 6:* Transcriptome and proteome evidence used for ENSEMBL Annotation.~~

Supplementary File 7: Transcriptome and proteome evidence used for ENSEMBL Annotation.

Competing Interests

J. Mohr and B.J. Wood were employed by Hybrid Turkeys and M.C.A.M Bink was employed by Hendrix Genetics Research. Both institutes are part of one of the funders (Hendrix Genetics). All authors declare that the results are presented in full and as such present no conflict of interest. The other Breed4Food partners Cobb Europe, CRV, Topigs Norsvin, declare to have no competing interests for this study.

Funding

This research was funded by the STW-Breed4Food Partnership, project number 14283: From sequence to phenotype: detecting deleterious variation by prediction of functionality. This study was

financially supported by NWO-TTW and the Breed4Food partners Cobb Europe, CRV, Hendrix Genetics and Topigs Norsvin.

Ethical Statement

Ethical review and approval were not required for sample collection since the data used in this study has been obtained as part of routine data collection from Hybrid Turkeys' breeding programmes, and not specifically for the purpose of this project. Therefore, approval of an ethics committee was not mandatory.

Authors' Contributions

MAMG designed, coordinated, and managed the project; JM and BJW were involved in data collection and preparation; RPMAC was involved in data collection and wet lab work; HJM provided valuable input regarding the analyses and manuscript; CPB and MFLD performed the analysis and drafted the manuscript. All authors read and approved the final manuscript.

Acknowledgements

We are grateful to Luohao Xu (Key Laboratory of Freshwater Fish Reproduction and Development, Southwest University, Chongqing) for sharing the annotation file for ZJU1.0. We thank the ENSEMBL support team for providing details on the annotation.

Abbreviations

BED: Browser Extensible Data; BLAST: Basic Local Alignment Search Tool; BLASTN: BLAST search of nucleotide database(s); BLASTP: BLAST search protein databases using a protein query; bp: base pairs; BUSCO: Benchmarking Universal Single-Copy Orthologs; BWA: Burrows-Wheeler Aligner; CDS: coding

sequence; F1: Filial 1, first offspring from a cross; FPKM: Fragments Per Kilobase Million; GC: guanine-cytosine; GFF3: general feature format, version 3; GWAS: genome wide association studies; Hi-C: chromosome conformation capture; INDEL: insertion or deletion; Kbp: kilo base pairs; LINE: Long interspersed nuclear elements; lnRNA: long non-coding RNA; LoF: loss of function; Mbp: megabase pairs; NCBI: National Center for Biotechnology Information; PacBio: Pacific Biosciences; SMN: survival motor neuron; SMRT: single molecule real time; SNP: single nucleotide polymorphism; VCF: variant call format.

References

~~[1] Association of Poultry Processors and Poultry Trade in the EU Countries (AVEC), "2021 Annual report," 2021. [Online]. Available: https://avec-poultry.eu/wp-content/uploads/2021/09/6226-AVEC-annual-report-2021_64.pdf.~~

~~[2] D. Chen *et al.*, "Divergence time estimation of Galliformes based on the best gene shopping scheme of ultraconserved elements," *BMC Ecol. Evol.*, vol. 21, no. 1, Dec. 2021, doi: 10.1186/s12862-021-01935-1.~~

~~[3] D. K. Griffin, L. B. W. Robertson, H. G. Tempest, and B. M. Skinner, "The evolution of the avian genome as revealed by comparative molecular cytogenetics," *Cytogenet. Genome Res.*, vol. 117, no. 1–4, pp. 64–77, 2007, doi: 10.1159/000103166.~~

~~[4] D. K. Griffin *et al.*, "Whole genome comparative studies between chicken and turkey and their implications for avian genome evolution," 2008, doi: 10.1186/1471-2164-9-168.~~

~~[5] R. A. Dalloul *et al.*, "Multi-platform next-generation sequencing of the domestic Turkey (Meleagris gallopavo): Genome assembly and analysis," *PLoS Biol.*, vol. 8, no. 9, p. e1000475, Sep. 2010, doi: 10.1371/journal.pbio.1000475.~~

~~[6] V. Peona *et al.*, "Identifying the causes and consequences of assembly gaps using a multiplatform genome assembly of a bird-of-paradise," *Mol. Ecol. Resour.*, vol. 21, no. 1, pp. 263–286, Jan. 2021, doi: 10.1111/1755-0998.13252.~~

~~[7] T. Meuwissen, B. Hayes, and M. Goddard, "Genomic selection: A paradigm shift in animal breeding," *Anim. Front.*, vol. 6, no. 1, pp. 6–14, Jan. 2016, doi: 10.2527/AF.2016-0002.~~

~~[8] C. Rexroad *et al.*, "Genome to phenome: Improving animal health, production, and well-being - A new USDA blueprint for animal genome research 2018-2027," *Front. Genet.*, vol. 10, no. MAY, 2019, doi: 10.3389/FGENE.2019.00327/FULL.~~

~~[9] K. M. Morris *et al.*, "The quail genome: Insights into social behaviour, seasonal biology and infectious disease response," *BMC Biol.*, vol. 18, no. 1, Feb. 2020, doi: 10.1186/s12915-020-0743-4.~~

~~[10] K. P. Oh, C. L. Aldridge, J. S. Forbey, C. Y. Dadabay, S. J. Oyler-Mccance, and C. Baer, "Conservation Genomics in the Sagebrush Sea: Population Divergence, Demographic History, and Local Adaptation in Sage-Grouse (Centrocercus spp.)," *Genome Biol. Evol.*, vol. 11, no. 7, pp. 2023–2034, Jul. 2019, doi: 10.1093/GBE/EVZ112.~~

~~[11] Q. K. Shen *et al.*, "Genomic Analyses of Unveil Helmeted Guinea Fowl (Numida meleagris) Domestication in West Africa," *Genome Biol. Evol.*, vol. 13, no. 6, Jun. 2021, doi: 10.1093/GBE/EVAB090.~~

~~[12] S. Koren *et al.*, "De novo assembly of haplotype-resolved genomes with trio binning," *Nat. Biotechnol.*, vol. 36, no. 12, pp. 1174–1182, 2018, doi: 10.1038/nbt.4277.~~

[13] J. Ruan and H. Li, "Fast and accurate long-read assembly with wtdbg2," *Nat. Methods*, vol. 17, no. 2, pp. 155–158, Dec. 2020, doi: 10.1038/s41592-019-0669-3.
[14] N. H. Putnam *et al.*, "Chromosome-scale shotgun assembly using an in vitro method for long-range linkage arXiv : 1502 . 05331v1 [q-bio . GN] 18 Feb 2015," *Genome Res.*, vol. 26, pp. 342–350, 2016, doi: 10.1101/gr.193474.115.Freely.
[15] J. Ghurye, M. Pop, S. Koren, D. Bickhart, and C. S. Chin, "Scaffolding of long read assemblies using long range contact information," *BMC Genomics*, vol. 18, no. 1, p. 527, Jul. 2017, doi: 10.1186/s12864-017-3879-z.
[16] L. P. Pryszcz, T. Gabald, and T. Gabaldón, "Redundans: An assembly pipeline for highly heterozygous genomes," *Nucleic Acids Res.*, vol. 44, no. 12, p. e113, 2016, doi: 10.1093/nar/gkw294.
[17] M. Qin *et al.*, "LRScaf: Improving Draft Genomes Using Long Noisy Reads," *bioRxiv*, pp. 1–12, 2018, doi: 10.1101/374868.
[18] M. Alonge *et al.*, "Automated assembly scaffolding elevates a new tomato system for high-throughput genome editing," p. 2021.11.18.469135, 2021.
[19] M. Seppey, M. Manni, and E. M. Zdobnov, *BUSCO: Assessing Genome Assembly and Annotation Completeness BT - Gene Prediction: Methods and Protocols*. 2019.
[20] J. M. Flynn *et al.*, "RepeatModeler2 for automated genomic discovery of transposable element families," *Proc. Natl. Acad. Sci. U. S. A.*, vol. 117, no. 17, pp. 9451–9457, 2020, doi: 10.1073/pnas.1921046117.
[21] F. Cunningham *et al.*, "Ensembl 2022," *Nucleic Acids Res.*, vol. 50, no. D1, pp. D988–D995, 2022, doi: 10.1093/nar/gkab1049.
[22] D. M. Emms and S. Kelly, "OrthoFinder: solving fundamental biases in whole genome comparisons dramatically improves orthogroup inference accuracy," *Genome Biol.*, vol. 16, no. 1, pp. 1–14, 2015, doi: 10.1186/s13059-015-0721-2.
[23] S. Fouchécourt *et al.*, "Expanding duplication of the testis PHD Finger Protein 7 (PHF7) gene in the chicken genome," *Genomics*, vol. 114, no. 4, p. 110411, Jul. 2022, doi: 10.1016/j.ygeno.2022.110411.
[24] C. J. Bult *et al.*, "Mouse Genome Database (MGD) 2019," *Nucleic Acids Res.*, vol. 47, no. D1, pp. D801–D806, Jan. 2019, doi: 10.1093/NAR/GKY1056.
[25] E. M. Campbell, D. J. Nonneman, L. A. Kuehn, and G. A. Rohrer, "Genetic variation in the mannosidase 2B2 gene and its association with ovulation rate in pigs," *Anim. Genet.*, vol. 39, no. 5, pp. 515–519, Oct. 2008, doi: 10.1111/J.1365-2052.2008.01763.X.
[26] M. Georges, C. Charlier, and B. Hayes, "Harnessing genomic information for livestock improvement," *Nat. Rev. Genet. 2018 203*, vol. 20, no. 3, pp. 135–156, Dec. 2018, doi: 10.1038/s41576-018-0082-2.
[27] M. Basaki, M. R. Tabandeh, M. Aminlari, K. Asasi, E. Mohsenifard, and B. Abdi Hachesoo, "Sequence and expression analysis of cardiac ryanodine receptor 2 in broilers that died from sudden death syndrome," *Avian Pathol.*, vol. 48, no. 5, pp. 444–453, 2019, doi: 10.1080/03079457.2019.1618439.
[28] M. J. Greenwold *et al.*, "Dynamic evolution of the alpha (α) and beta (β) keratins has accompanied integument diversification and the adaptation of birds into novel lifestyles," *BMC Evol. Biol.*, vol. 14, no. 1, 2014, doi: 10.1186/S12862-014-0249-1.
[29] X. R. Wang, L. Bin Ling, H. H. Huang, J. J. Lin, S. D. Fugmann, and S. Y. Yang, "Evidence for parallel evolution of a gene involved in the regulation of spermatogenesis," *Proc. R. Soc. B Biol. Sci.*, vol. 284, no. 1855, May 2017, doi: 10.1098/RSPB.2017.0324.
[30] S. Fouchécourt *et al.*, "An evolutionary approach to recover genes predominantly expressed in the testes of the zebrafish, chicken and mouse," *BMC Evol. Biol.*, vol. 19, no. 1, pp. 1–15, Jul. 2019, doi: 10.1186/S12862-019-1462-8/FIGURES/4.
[31] I. Braasch *et al.*, "The spotted gar genome illuminates vertebrate evolution and facilitates human-teleost comparisons," *Nat. Genet.*, vol. 48, no. 4, pp. 427–437, Mar. 2016, doi:

10.1038/ng.3526.
[32] P. D. Waters *et al.*, “Microchromosomes are building blocks of bird, reptile, and mammal chromosomes,” *Proc. Natl. Acad. Sci. U. S. A.*, vol. 118, no. 45, Nov. 2021, doi: 10.1073/PNAS.2112494118/-/DCSUPPLEMENTAL.
[33] G. Zhang *et al.*, “Comparative genomics reveals insights into avian genome evolution and adaptation,” *Science (80-.).*, vol. 346, no. 6215, pp. 1311–1320, Dec. 2014, doi: 10.1126/SCIENCE.1251385.
[34] M. L. Aslam, J. W. M. Bastiaansen, R. P. M. A. Crooijmans, A. Vereijken, H. J. Megens, and M. A. M. Groenen, “A SNP based linkage map of the turkey genome reveals multiple intrachromosomal rearrangements between the Turkey and Chicken genomes,” *BMC Genomics*, vol. 11, no. 1, pp. 1–11, Nov. 2010, doi: 10.1186/1471-2164-11-647/FIGURES/3.
[35] Y. Zhang *et al.*, “A comparative physical map reveals the pattern of chromosomal evolution between the turkey (Meleagris gallopavo) and chicken (Gallus gallus) genomes,” *BMC Genomics*, vol. 12, p. 447, Sep. 2011, doi: 10.1186/1471-2164-12-447.
[36] B. J. Walker *et al.*, “Pilon: An integrated tool for comprehensive microbial variant detection and genome assembly improvement,” *PLoS One*, vol. 9, no. 11, 2014, doi: 10.1371/journal.pone.0112963.
[37] Arima Genomics, “Arima Genomics Mapping Pipeline,” 2019. https://github.com/ArimaGenomics/mapping_pipeline.
[38] H. Li and R. Durbin, “Fast and accurate short read alignment with Burrows – Wheeler transform,” vol. 25, no. 14, pp. 1754–1760, 2009, doi: 10.1093/bioinformatics/btp324.
[39] “Picard toolkit,” *Broad Institute, GitHub repository*. Broad Institute, 2019.
[40] L. P. Pryszcz and T. Gabaldón, “Redundans: An assembly pipeline for highly heterozygous genomes,” *Nucleic Acids Res.*, vol. 44, no. 12, p. e113, 2016, doi: 10.1093/nar/gkw294.
[41] N. C. Durand *et al.*, “Juicer Provides a One-Click System for Analyzing Loop-Resolution Hi-C Experiments,” *Cell Syst.*, vol. 3, no. 1, pp. 95–98, 2016, doi: 10.1016/j.cels.2016.07.002.
[42] O. Dudchenko *et al.*, “De novo assembly of the Aedes aegypti genome using Hi-C yields chromosome-length scaffolds,” *Science (80-.).*, vol. 356, no. 6333, pp. 92–95, 2017, doi: 10.1126/science.aal3327.
[43] N. C. Durand *et al.*, “Juicebox Provides a Visualization System for Hi-C Contact Maps with Unlimited Zoom,” *Cell Syst.*, vol. 3, no. 1, pp. 99–101, 2016, doi: 10.1016/j.cels.2015.07.012.
[44] R. Buels *et al.*, “JBrowse: a dynamic web platform for genome visualization and analysis,” *Genome Biol.*, vol. 17, no. 1, Apr. 2016, doi: 10.1186/S13059-016-0924-1.
[45] H. Li, “Minimap2 : pairwise alignment for nucleotide sequences,” vol. 34, no. May, pp. 3094–3100, 2018, doi: 10.1093/bioinformatics/bty191.
[46] M. Alonge *et al.*, “Fast and accurate reference-guided scaffolding of draft genomes,” *bioRxiv*, pp. 1–17, 2019, doi: 10.1101/519637.
[47] F. Cabanettes and C. Klopp, “D-GENIES: dot plot large genomes in an interactive, efficient and simple way,” *PeerJ*, vol. 6, no. 6, 2018, doi: 10.7717/PEERJ.4958.
[48] M. Goel, H. Sun, W. B. Jiao, and K. Schneeberger, “SyRI: finding genomic rearrangements and local sequence differences from whole-genome assemblies,” *Genome Biol.*, vol. 20, no. 1, Dec. 2019, doi: 10.1186/S13059-019-1911-0.
[49] M. Goel and K. Schneeberger, “plotsr: Visualising structural similarities and rearrangements between multiple genomes,” *Bioinformatics*, vol. 38, no. 10, pp. 2922–2926, May 2022, doi: 10.1093/BIOINFORMATICS/BTAC196.
[50] G. G. Faust and I. M. Hall, “SAMBLASTER: Fast duplicate marking and structural variant read extraction,” *Bioinformatics*, vol. 30, no. 17, pp. 2503–2505, 2014, doi: 10.1093/bioinformatics/btu314.
[51] P. Danecek *et al.*, “Twelve years of SAMtools and BCFtools,” *Gigascience*, vol. 10, no. 2, pp. 1–4, Jan. 2021, doi: 10.1093/GIGASCIENCE/GIAB008.
[52] E. Garrison and G. Marth, “Haplotype-based variant detection from short-read sequencing,”

pp. 1–9, 2012, [Online]. Available: http://arxiv.org/abs/1207.3907.
[53] E. Garrison, Z. N. Kronenberg, E. T. Dawson, B. S. Pedersen, and P. Prins, "Vcflib and tools for processing the VCF variant call format," *bioRxiv*, p. 2021.05.21.445151, May 2021, doi: 10.1101/2021.05.21.445151.
[54] J. K. Bonfield *et al.*, "HTSlib: C library for reading/writing high-throughput sequencing data," *Gigascience*, vol. 10, no. 2, pp. 1–6, Jan. 2021, doi: 10.1093/GIGASCIENCE/GIAB007.
[55] H. Li *et al.*, "The Sequence Alignment/Map format and SAMtools," *Bioinformatics*, vol. 25, no. 16, pp. 2078–2079, 2009, doi: 10.1093/bioinformatics/btp352.
[56] K. Okonechnikov, A. Conesa, and F. García-Alcalde, "Qualimap 2: Advanced multi-sample quality control for high-throughput sequencing data," *Bioinformatics*, vol. 32, no. 2, pp. 292–294, 2016, doi: 10.1093/bioinformatics/btv566.
[57] G. Marçais, A. L. Delcher, A. M. Phillippy, R. Coston, S. L. Salzberg, and A. Zimin, "MUMmer4: A fast and versatile genome alignment system," *PLoS Comput. Biol.*, vol. 14, no. 1, pp. 1–14, 2018, doi: 10.1371/journal.pcbi.1005944.
[58] S. V. Angiuoli and S. L. Salzberg, "Mugsy: fast multiple alignment of closely related whole genomes," *Bioinformatics*, vol. 27, no. 3, pp. 334–342, Feb. 2011, doi: 10.1093/BIOINFORMATICS/BTQ665.
[59] H. Zhao, Z. Sun, J. Wang, H. Huang, J. P. Kocher, and L. Wang, "CrossMap: A versatile tool for coordinate conversion between genome assemblies," *Bioinformatics*, vol. 30, no. 7, pp. 1006–1007, 2014, doi: 10.1093/bioinformatics/btt730.
[60] C. Camacho *et al.*, "BLAST+: Architecture and applications," *BMC Bioinformatics*, vol. 10, no. 1, pp. 1–9, Dec. 2009, doi: 10.1186/1471-2105-10-421/FIGURES/4.
[61] A. Smith, R. Hubley, and P. Green, "RepeatMasker Open-4.0." http://www.repeatmasker.org.
[62] D. M. Emms and S. Kelly, "OrthoFinder: Phylogenetic orthology inference for comparative genomics," *Genome Biol.*, vol. 20, no. 1, pp. 1–14, Nov. 2019, doi: 10.1186/S13059-019-1832-Y/FIGURES/5.
[63] R Core Team, "R: A Language and Environment for Statistical Computing." Vienna, Austria, 2020, [Online]. Available: https://www.r-project.org/.
[64] Z. Hao *et al.*, "RIdeogram: Drawing SVG graphics to visualize and map genome-wide data on the idiograms," *PeerJ Comput. Sci.*, vol. 6, pp. 1–11, 2020, doi: 10.7717/PEERJ-CS.251.
[65] D. Kim, B. Langmead, and S. L. Salzberg, "HISAT: a fast spliced aligner with low memory requirements," *Nat. Methods*, vol. 12, no. 4, pp. 357–360, Mar. 2015, doi: 10.1038/NMETH.3317.
[66] M. Pertea, G. M. Pertea, C. M. Antonescu, T. C. Chang, J. T. Mendell, and S. L. Salzberg, "StringTie enables improved reconstruction of a transcriptome from RNA-seq reads," *Nat. Biotechnol.*, vol. 33, no. 3, p. 290, 2015, doi: 10.1038/NBT.3122.
[67] H. Tang, J. E. Bowers, X. Wang, R. Ming, M. Alam, and A. H. Paterson, "Synteny and Collinearity in Plant Genomes," *Science (80 .).*, no. April, pp. 486–489, 2008, [Online]. Available: http://www.sciencemag.org/content/320/5875/486.full.pdf.
[68] Z. Huang *et al.*, "Recurrent chromosome reshuffling and the evolution of neo-sex chromosomes in parrots," *Nat. Commun.*, vol. 13, no. 1, pp. 1–11, 2022, doi: 10.1038/s41467-022-28585-1.

1. (AVEC), A.o.P.P.a.P.T.i.t.E.C., *2021 Annual report.* 2021.

Formatted: Space After: 0 pt

Formatted: Normal, Indent: Left: 0", First line: 0"

2. Chen, D., et al., *Divergence time estimation of Galliformes based on the best gene shopping scheme of ultraconserved elements.* Bmc Ecology and Evolution, 2021. **21**(1).

3. Griffin, D., et al., *The evolution of the avian genome as revealed by comparative molecular cytogenetics.* Chromosome Research, 2007. **15**: p. 29-29.

4. Griffin, D.K., et al., *Whole genome comparative studies between chicken and turkey and their implications for avian genome evolution.* Bmc Genomics, 2008. **9**.

5. Zhang, Y., et al., *A comparative physical map reveals the pattern of chromosomal evolution between the turkey (Meleagris gallopavo) and chicken (Gallus gallus) genomes.* Bmc Genomics, 2011. **12**.

6. Dalloul, R.A., et al., *Multi-Platform Next-Generation Sequencing of the Domestic Turkey (Meleagris gallopavo): Genome Assembly and Analysis.* Plos Biology, 2010. **8**(9).

7. Peona, V., et al., *Identifying the causes and consequences of assembly gaps using a multiplatform genome assembly of a bird-of-paradise.* Molecular Ecology Resources, 2021. **21**(1): p. 263-286.

8. Meuwissen, T., B. Hayes, and M. Goddard, *Genomic selection: A paradigm shift in animal breeding.* Animal Frontiers, 2016. **6**(1): p. 6-14.

9. Rexroad, C., et al., *Genome to Phenome: Improving Animal Health, Production, and Well-Being - A New USDA Blueprint for Animal Genome Research 2018-2027.* Frontiers in Genetics, 2019. **10**.

10. Morris, K.M., et al., *The quail genome: insights into social behaviour, seasonal biology and infectious disease response.* Bmc Biology, 2020. **18**(1).

11. Oh, K.P., et al., *Conservation Genomics in the Sagebrush Sea: Population Divergence, Demographic History, and Local Adaptation in Sage-Grouse (Centrocercus spp.).* Genome Biology and Evolution, 2019. **11**(7): p. 2023-2034.

12. Shen, Q.K., et al., *Genomic Analyses of Unveil Helmeted Guinea Fowl (Numida meleagris) Domestication in West Africa.* Genome Biology and Evolution, 2021. **13**(6).

13. Koren, S., et al., *De novo assembly of haplotype-resolved genomes with trio binning.* Nature Biotechnology, 2018. **36**(12): p. 1174-+.

14. Ruan, J. and H. Li, *Fast and accurate long-read assembly with wtdbg2.* Nature Methods, 2020. **17**(2): p. 155-+.

15. Putnam, N.H., et al., *Chromosome-scale shotgun assembly using an in vitro method for long-range linkage.* Genome Research, 2016. **26**(3): p. 342-350.

16. Ghurye, J., et al., *Scaffolding of long read assemblies using long range contact information.* Bmc Genomics, 2017. **18**.

17. Pryszcz, L.P. and T. Gabaldon, *Redundans: an assembly pipeline for highly heterozygous genomes.* Nucleic Acids Research, 2016. **44**(12).

18. Qin, M., et al., *LRScaf: improving draft genomes using long noisy reads.* Bmc Genomics, 2019. **20**(1).

19. Alonge, M., et al., *RaGOO: fast and accurate reference-guided scaffolding of draft genomes.* Genome Biology, 2019. **20**(1).

20. Rhie, A., et al., *Merqury: reference-free quality, completeness, and phasing assessment for genome assemblies.* Genome Biology, 2020. **21**(1).

21. Simao, F.A., et al., *BUSCO: assessing genome assembly and annotation completeness with single-copy orthologs.* Bioinformatics, 2015. **31**(19): p. 3210-3212.

22. Flynn, J.M., et al., *RepeatModeler2 for automated genomic discovery of transposable element families.* Proceedings of the National Academy of Sciences of the United States of America, 2020. **117**(17): p. 9451-9457.

23. Cunningham, F., et al., *Ensembl 2022.* Nucleic Acids Research, 2022. **50**(D1): p. D988-D995.

24. Emms, D.M. and S. Kelly, *OrthoFinder: solving fundamental biases in whole genome comparisons dramatically improves orthogroup inference accuracy.* Genome Biology, 2015. **16**.

25. Warren, W.C., et al., *The genome of a songbird.* Nature, 2010. **464**(7289): p. 757-762.

26. Mendes, F.K., et al., *CAFE 5 models variation in evolutionary rates among gene families.* Bioinformatics, 2020. **36**(22-23): p. 5516-5518.

27. Fouchecourt, S., et al., *Expanding duplication of the testis PHD Finger Protein 7 (PHF7) gene in the chicken genome.* Genomics, 2022. **114**(4).

28. Kaufman, J., *Innate immune genes of the chicken MHC and related regions.* Immunogenetics, 2022. **74**(1): p. 167-177.

29. Campbell, E.M., et al., *Genetic variation in the mannosidase 2B2 gene and its association with ovulation rate in pigs.* Animal Genetics, 2008. **39**(5): p. 515-519.

30. Georges, M., C. Charlier, and B. Hayes, *Harnessing genomic information for livestock improvement.* Nature Reviews Genetics, 2019. **20**(3): p. 135-156.

31. Basaki, M., et al., *Sequence and expression analysis of cardiac ryanodine receptor 2 in broilers that died from sudden death syndrome.* Avian Pathology, 2019. **48**(5): p. 444-453.

32. Bellott, D.W., et al., *Avian W and mammalian Y chromosomes convergently retained dosage-sensitive regulators.* Nature Genetics, 2017. **49**(3): p. 387-394.

33. Li, Y.I., et al., *Rapid Evolution of Beta-Keratin Genes Contribute to Phenotypic Differences That Distinguish Turtles and Birds from Other Reptiles.* Genome Biology and Evolution, 2013. **5**(5): p. 923-933.

34. Wang, X.R., et al., *Evidence for parallel evolution of a gene involved in the regulation of spermatogenesis.* Proceedings of the Royal Society B-Biological Sciences, 2017. **284**(1855).

35. Fouchecourt, S., et al., *An evolutionary approach to recover genes predominantly expressed in the testes of the zebrafish, chicken and mouse.* Bmc Evolutionary Biology, 2019. **19**.

36. Braasch, I., et al., *The spotted gar genome illuminates vertebrate evolution and facilitates human-teleost comparisons (vol 48, pg 427, 2016).* Nature Genetics, 2016. **48**(6): p. 700-700.

37. Waters, P.D., et al., *Microchromosomes are building blocks of bird, reptile, and mammal chromosomes.* Proc Natl Acad Sci U S A, 2021. **118**(45).

38. Zhang, G.J., et al., *Comparative genomics reveals insights into avian genome evolution and adaptation.* Science, 2014. **346**(6215): p. 1311-1320.

39. Aslam, M.L., et al., *A SNP based linkage map of the turkey genome reveals multiple intrachromosomal rearrangements between the Turkey and Chicken genomes.* Bmc Genomics, 2010. **11**.

40. Walker, B.J., et al., *Pilon: An Integrated Tool for Comprehensive Microbial Variant Detection and Genome Assembly Improvement.* Plos One, 2014. **9**(11).

41. Genomics, A. *Arima Genomics Pipeline*. 2019; Available from: https://github.com/ArimaGenomics/mapping_pipeline.

42. Li, H. and R. Durbin, *Fast and accurate short read alignment with Burrows-Wheeler transform.* Bioinformatics, 2009. **25**(14): p. 1754-1760.

43. Institute, B. *Picard: A set of Java command line tools for manipulating high-throughput sequencing data (HTS) data and formats*. 2022; Available from: http://broadinstitute.github.io/picard/.

44. Durand, N.C., et al., *Juicer Provides a One-Click System for Analyzing Loop-Resolution Hi-C Experiments.* Cell Systems, 2016. **3**(1): p. 95-98.

45. Durand, N.C., et al., *Juicebox Provides a Visualization System for Hi-C Contact Maps with Unlimited Zoom.* Cell Systems, 2016. **3**(1): p. 99-101.

46. Buels, R., et al., *JBrowse: a dynamic web platform for genome visualization and analysis.* Genome Biology, 2016. **17**.

47. Li, H., *Minimap2: pairwise alignment for nucleotide sequences.* Bioinformatics, 2018. **34**(18): p. 3094-3100.

48. Cabanettes, F. and C. Klopp, *D-GENIES: dot plot large genomes in an interactive, efficient and simple way.* Peerj, 2018. **6**.

49. Goel, M., et al., *SyRI: finding genomic rearrangements and local sequence differences from whole-genome assemblies.* Genome Biology, 2019. **20**(1).

50. Goel, M. and K. Schneeberger, *plotsr: visualizing structural similarities and rearrangements between multiple genomes (vol 38, pg 2922, 2022).* Bioinformatics, 2022.

51. Faust, G.G. and I.M. Hall, *SAMBLASTER: fast duplicate marking and structural variant read extraction.* Bioinformatics, 2014. **30**(17): p. 2503-2505.

52. Li, H., et al., *The Sequence Alignment/Map format and SAMtools.* Bioinformatics, 2009. **25**(16): p. 2078-2079.

53. E. Garrison and G. Marth, *Haplotype-based variant detection from short-read sequencing.* 2012.

54. Bonfield, J.K., et al., *HTSlib: C library for reading/writing high-throughput sequencing data.* Gigascience, 2021. **10**(2).

55. Danecek, P., et al., *Twelve years of SAMtools and BCFtools.* Gigascience, 2021. **10**(2).

56. Okonechnikov, K., A. Conesa, and F. Garcia-Alcalde, *Qualimap 2: advanced multi-sample quality control for high-throughput sequencing data.* Bioinformatics, 2016. **32**(2): p. 292-294.

57. Marcais, G., et al., *MUMmer4: A fast and versatile genome alignment system.* Plos Computational Biology, 2018. **14**(1).

58. Angiuoli, S.V. and S.L. Salzberg, *Mugsy: fast multiple alignment of closely related whole genomes.* Bioinformatics, 2011. **27**(3): p. 334-342.

59. Camacho, C., et al., *BLAST plus : architecture and applications.* Bmc Bioinformatics, 2009. **10**.

60. A. Smith, R. Hubley, and P. Green, *RepeatMasker Open-4.0.* 2013-2015.

61. Emms, D.M. and S. Kelly, *OrthoFinder: phylogenetic orthology inference for comparative genomics.* Genome Biology, 2019. **20**(1).

62. Jetz, W., et al., *The global diversity of birds in space and time.* Nature, 2012. **491**(7424): p. 444-448.

63. Team, R.C. *R: A Language and Environment for Statistical Computing.* 2020; Available from: https://www.r-project.org/.

64. Hao, Z.D., et al., *RIdeogram: drawing SVG graphics to visualize and map genome-wide data on the idiograms.* Peerj Computer Science, 2020.

65. Kim, D., B. Landmead, and S.L. Salzberg, *HISAT: a fast spliced aligner with low memory requirements.* Nature Methods, 2015. **12**(4): p. 357-U121.

66. Pertea, M., et al., *StringTie enables improved reconstruction of a transcriptome from RNA-seq reads.* Nature Biotechnology, 2015. **33**(3): p. 290-+.

67. Wang, Y.P., et al., *MCScanX: a toolkit for detection and evolutionary analysis of gene synteny and collinearity.* Nucleic Acids Research, 2012. **40**(7).

68. Liu, J., et al., *A new emu genome illuminates the evolution of genome configuration and nuclear architecture of avian chromosomes.* Genome Research, 2021. **31**(3): p. 497-511.

1. (AVEC), A.o.P.P.a.P.T.i.t.E.C., *2021 Annual report.* 2021.
2. Chen, D., et al., *Divergence time estimation of Galliformes based on the best gene shopping scheme of ultraconserved elements.* Bmc Ecology and Evolution, 2021. **21**(1).
3. Griffin, D., et al., *The evolution of the avian genome as revealed by comparative molecular cytogenetics.* Chromosome Research, 2007. **15**: p. 29-29.
4. Griffin, D.K., et al., *Whole genome comparative studies between chicken and turkey and their implications for avian genome evolution.* Bmc Genomics, 2008. **9**.
5. Zhang, Y., et al., *A comparative physical map reveals the pattern of chromosomal evolution between the turkey (Meleagris gallopavo) and chicken (Gallus gallus) genomes.* Bmc Genomics, 2011. **12**.
6. Dalloul, R.A., et al., *Multi-Platform Next-Generation Sequencing of the Domestic Turkey (Meleagris gallopavo): Genome Assembly and Analysis.* Plos Biology, 2010. **8**(9).
7. Peona, V., et al., *Identifying the causes and consequences of assembly gaps using a multiplatform genome assembly of a bird-of-paradise.* Molecular Ecology Resources, 2021. **21**(1): p. 263-286.
8. Meuwissen, T., B. Hayes, and M. Goddard, *Genomic selection: A paradigm shift in animal breeding.* Animal Frontiers, 2016. **6**(1): p. 6-14.
9. Rexroad, C., et al., *Genome to Phenome: Improving Animal Health, Production, and Well-Being - A New USDA Blueprint for Animal Genome Research 2018-2027.* Frontiers in Genetics, 2019. **10**.
10. Morris, K.M., et al., *The quail genome: insights into social behaviour, seasonal biology and infectious disease response.* Bmc Biology, 2020. **18**(1).
11. Oh, K.P., et al., *Conservation Genomics in the Sagebrush Sea: Population Divergence, Demographic History, and Local Adaptation in Sage-Grouse (Centrocercus spp.).* Genome Biology and Evolution, 2019. **11**(7): p. 2023-2034.
12. Shen, Q.K., et al., *Genomic Analyses of Unveil Helmeted Guinea Fowl (Numida meleagris) Domestication in West Africa.* Genome Biology and Evolution, 2021. **13**(6).

13. Koren, S., et al., *De novo assembly of haplotype-resolved genomes with trio binning.* Nature Biotechnology, 2018. **36**(12): p. 1174-+.
14. Ruan, J. and H. Li, *Fast and accurate long-read assembly with wtdbg2.* Nature Methods, 2020. **17**(2): p. 155-+.
15. Putnam, N.H., et al., *Chromosome-scale shotgun assembly using an in vitro method for long-range linkage.* Genome Research, 2016. **26**(3): p. 342-350.
16. Ghurye, J., et al., *Scaffolding of long read assemblies using long range contact information.* Bmc Genomics, 2017. **18**.
17. Pryszcz, L.P. and T. Gabaldon, *Redundans: an assembly pipeline for highly heterozygous genomes.* Nucleic Acids Research, 2016. **44**(12).
18. Qin, M., et al., *LRScaf: improving draft genomes using long noisy reads.* Bmc Genomics, 2019. **20**(1).
19. Alonge, M., et al., *RaGOO: fast and accurate reference-guided scaffolding of draft genomes.* Genome Biology, 2019. **20**(1).
20. Rhie, A., et al., *Merqury: reference-free quality, completeness, and phasing assessment for genome assemblies.* Genome Biology, 2020. **21**(1).
21. Simao, F.A., et al., *BUSCO: assessing genome assembly and annotation completeness with single-copy orthologs.* Bioinformatics, 2015. **31**(19): p. 3210-3212.
22. Flynn, J.M., et al., *RepeatModeler2 for automated genomic discovery of transposable element families.* Proceedings of the National Academy of Sciences of the United States of America, 2020. **117**(17): p. 9451-9457.
23. Cunningham, F., et al., *Ensembl 2022.* Nucleic Acids Research, 2022. **50**(D1): p. D988-D995.
24. Emms, D.M. and S. Kelly, *OrthoFinder: solving fundamental biases in whole genome comparisons dramatically improves orthogroup inference accuracy.* Genome Biology, 2015. **16**.
25. Warren, W.C., et al., *The genome of a songbird.* Nature, 2010. **464**(7289): p. 757-762.
26. Mendes, F.K., et al., *CAFE 5 models variation in evolutionary rates among gene families.* Bioinformatics, 2020. **36**(22-23): p. 5516-5518.
27. Fouchecourt, S., et al., *Expanding duplication of the testis PHD Finger Protein 7 (PHF7) gene in the chicken genome.* Genomics, 2022. **114**(4).
28. Kaufman, J., *Innate immune genes of the chicken MHC and related regions.* Immunogenetics, 2022. **74**(1): p. 167-177.
29. Campbell, E.M., et al., *Genetic variation in the mannosidase 2B2 gene and its association with ovulation rate in pigs.* Animal Genetics, 2008. **39**(5): p. 515-519.
30. Georges, M., C. Charlier, and B. Hayes, *Harnessing genomic information for livestock improvement.* Nature Reviews Genetics, 2019. **20**(3): p. 135-156.
31. Basaki, M., et al., *Sequence and expression analysis of cardiac ryanodine receptor 2 in broilers that died from sudden death syndrome.* Avian Pathology, 2019. **48**(5): p. 444-453.
32. Bult, C.J., et al., *Mouse Genome Database (MGD) 2019.* Nucleic Acids Research, 2019. **47**(D1): p. D801-D806.
33. Waters, P.D., et al., *Microchromosomes are building blocks of bird, reptile, and mammal chromosomes.* Proc Natl Acad Sci U S A, 2021. **118**(45).
34. O'Connor, R.E., et al., *Patterns of microchromosome organization remain highly conserved throughout avian evolution.* Chromosoma, 2019. **128**(1): p. 21-29.
35. Shibusawa, M., et al., *Karyotypic evolution in the Galliformes: An examination of the process of karyotypic evolution by comparison of the molecular cytogenetic findings with the molecular phylogeny.* Cytogenetic and Genome Research, 2004. **106**(1): p. 111-119.
36. Xu, L.H., et al., *Dynamic evolutionary history and gene content of sex chromosomes across diverse songbirds.* Nature Ecology & Evolution, 2019. **3**(5): p. 834-844.
37. Bellott, D.W., et al., *Avian W and mammalian Y chromosomes convergently retained dosage-sensitive regulators.* Nature Genetics, 2017. **49**(3): p. 387-394.

38. Li, Y.I., et al., *Rapid Evolution of Beta-Keratin Genes Contribute to Phenotypic Differences That Distinguish Turtles and Birds from Other Reptiles.* Genome Biology and Evolution, 2013. **5**(5): p. 923-933.
39. Wang, X.R., et al., *Evidence for parallel evolution of a gene involved in the regulation of spermatogenesis.* Proceedings of the Royal Society B-Biological Sciences, 2017. **284**(1855).
40. Fouchecourt, S., et al., *An evolutionary approach to recover genes predominantly expressed in the testes of the zebrafish, chicken and mouse.* Bmc Evolutionary Biology, 2019. **19**.
41. Braasch, I., et al., *The spotted gar genome illuminates vertebrate evolution and facilitates human-teleost comparisons (vol 48, pg 427, 2016).* Nature Genetics, 2016. **48**(6): p. 700-700.
42. Kapusta, A. and A. Suh, *Evolution of bird genomes-a transposon's-eye view.* Annals of the New York Academy of Sciences, 2017. **1389**(1): p. 164-185.
43. Zhang, G.J., et al., *Comparative genomics reveals insights into avian genome evolution and adaptation.* Science, 2014. **346**(6215): p. 1311-1320.
44. Aslam, M.L., et al., *A SNP based linkage map of the turkey genome reveals multiple intrachromosomal rearrangements between the Turkey and Chicken genomes.* Bmc Genomics, 2010. **11**.
45. Walker, B.J., et al., *Pilon: An Integrated Tool for Comprehensive Microbial Variant Detection and Genome Assembly Improvement.* Plos One, 2014. **9**(11).
46. Genomics, A. *Arima Genomics Pipeline*. 2019; Available from: https://github.com/ArimaGenomics/mapping_pipeline.
47. Li, H. and R. Durbin, *Fast and accurate short read alignment with Burrows-Wheeler transform.* Bioinformatics, 2009. **25**(14): p. 1754-1760.
48. Institute, B. *Picard: A set of Java command line tools for manipulating high-throughput sequencing data (HTS) data and formats*. 2022; Available from: http://broadinstitute.github.io/picard/.
49. Durand, N.C., et al., *Juicer Provides a One-Click System for Analyzing Loop-Resolution Hi-C Experiments.* Cell Systems, 2016. **3**(1): p. 95-98.
50. Durand, N.C., et al., *Juicebox Provides a Visualization System for Hi-C Contact Maps with Unlimited Zoom.* Cell Systems, 2016. **3**(1): p. 99-101.
51. Buels, R., et al., *JBrowse: a dynamic web platform for genome visualization and analysis.* Genome Biology, 2016. **17**.
52. Li, H., *Minimap2: pairwise alignment for nucleotide sequences.* Bioinformatics, 2018. **34**(18): p. 3094-3100.
53. Cabanettes, F. and C. Klopp, *D-GENIES: dot plot large genomes in an interactive, efficient and simple way.* Peerj, 2018. **6**.
54. Goel, M., et al., *SyRI: finding genomic rearrangements and local sequence differences from whole-genome assemblies.* Genome Biology, 2019. **20**(1).
55. Goel, M. and K. Schneeberger, *plotsr: visualizing structural similarities and rearrangements between multiple genomes (vol 38, pg 2922, 2022).* Bioinformatics, 2022.
56. Faust, G.G. and I.M. Hall, *SAMBLASTER: fast duplicate marking and structural variant read extraction.* Bioinformatics, 2014. **30**(17): p. 2503-2505.
57. Li, H., et al., *The Sequence Alignment/Map format and SAMtools.* Bioinformatics, 2009. **25**(16): p. 2078-2079.
58. E. Garrison and G. Marth, *Haplotype-based variant detection from short-read sequencing.* 2012.
59. Bonfield, J.K., et al., *HTSlib: C library for reading/writing high-throughput sequencing data.* Gigascience, 2021. **10**(2).
60. Danecek, P., et al., *Twelve years of SAMtools and BCFtools.* Gigascience, 2021. **10**(2).
61. Okonechnikov, K., A. Conesa, and F. Garcia-Alcalde, *Qualimap 2: advanced multi-sample quality control for high-throughput sequencing data.* Bioinformatics, 2016. **32**(2): p. 292-294.

62. Marcais, G., et al., *MUMmer4: A fast and versatile genome alignment system.* Plos Computational Biology, 2018. **14**(1).
63. Angiuoli, S.V. and S.L. Salzberg, *Mugsy: fast multiple alignment of closely related whole genomes.* Bioinformatics, 2011. **27**(3): p. 334-342.
64. Camacho, C., et al., *BLAST plus : architecture and applications.* Bmc Bioinformatics, 2009. **10**.
65. A. Smith, R. Hubley, and P. Green, *RepeatMasker Open-4.0.* 2013-2015.
66. Emms, D.M. and S. Kelly, *OrthoFinder: phylogenetic orthology inference for comparative genomics.* Genome Biology, 2019. **20**(1).
67. Jetz, W., et al., *The global diversity of birds in space and time.* Nature, 2012. **491**(7424): p. 444-448.
68. Team, R.C. *R: A Language and Environment for Statistical Computing*. 2020; Available from: https://www.r-project.org/.
69. Hao, Z.D., et al., *RIdeogram: drawing SVG graphics to visualize and map genome-wide data on the idiograms.* Peerj Computer Science, 2020.
70. Kim, D., B. Landmead, and S.L. Salzberg, *HISAT: a fast spliced aligner with low memory requirements.* Nature Methods, 2015. **12**(4): p. 357-U121.
71. Pertea, M., et al., *StringTie enables improved reconstruction of a transcriptome from RNA-seq reads.* Nature Biotechnology, 2015. **33**(3): p. 290-+.
72. Wang, Y.P., et al., *MCScanX: a toolkit for detection and evolutionary analysis of gene synteny and collinearity.* Nucleic Acids Research, 2012. **40**(7).
73. Liu, J., et al., *A new emu genome illuminates the evolution of genome configuration and nuclear architecture of avian chromosomes.* Genome Research, 2021. **31**(3): p. 497-511.

Figure 1

Click here to access/download;Figure;Figure1.tif

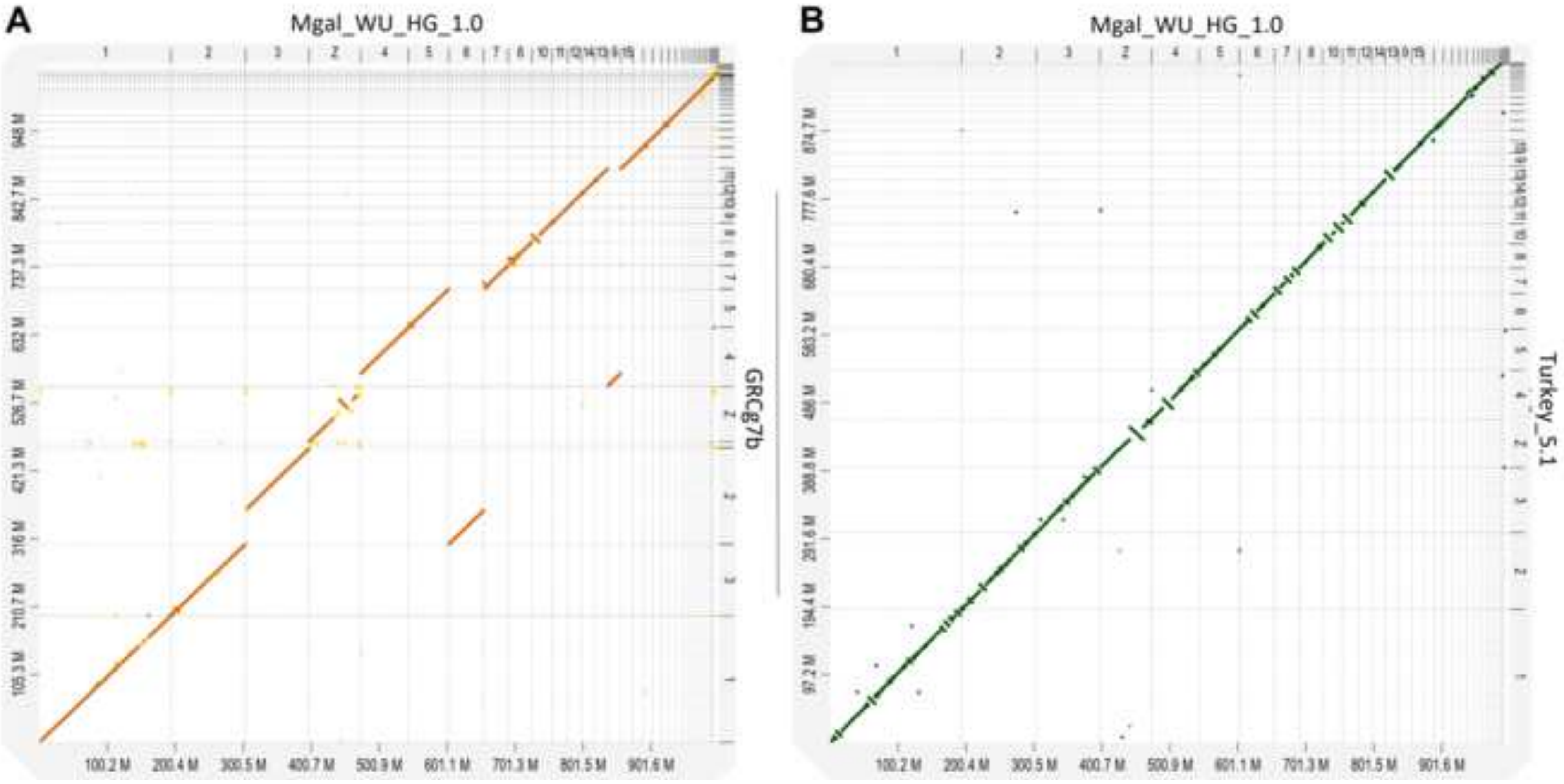

Figure 2

Click here to access/download;Figure;Figure2.tif

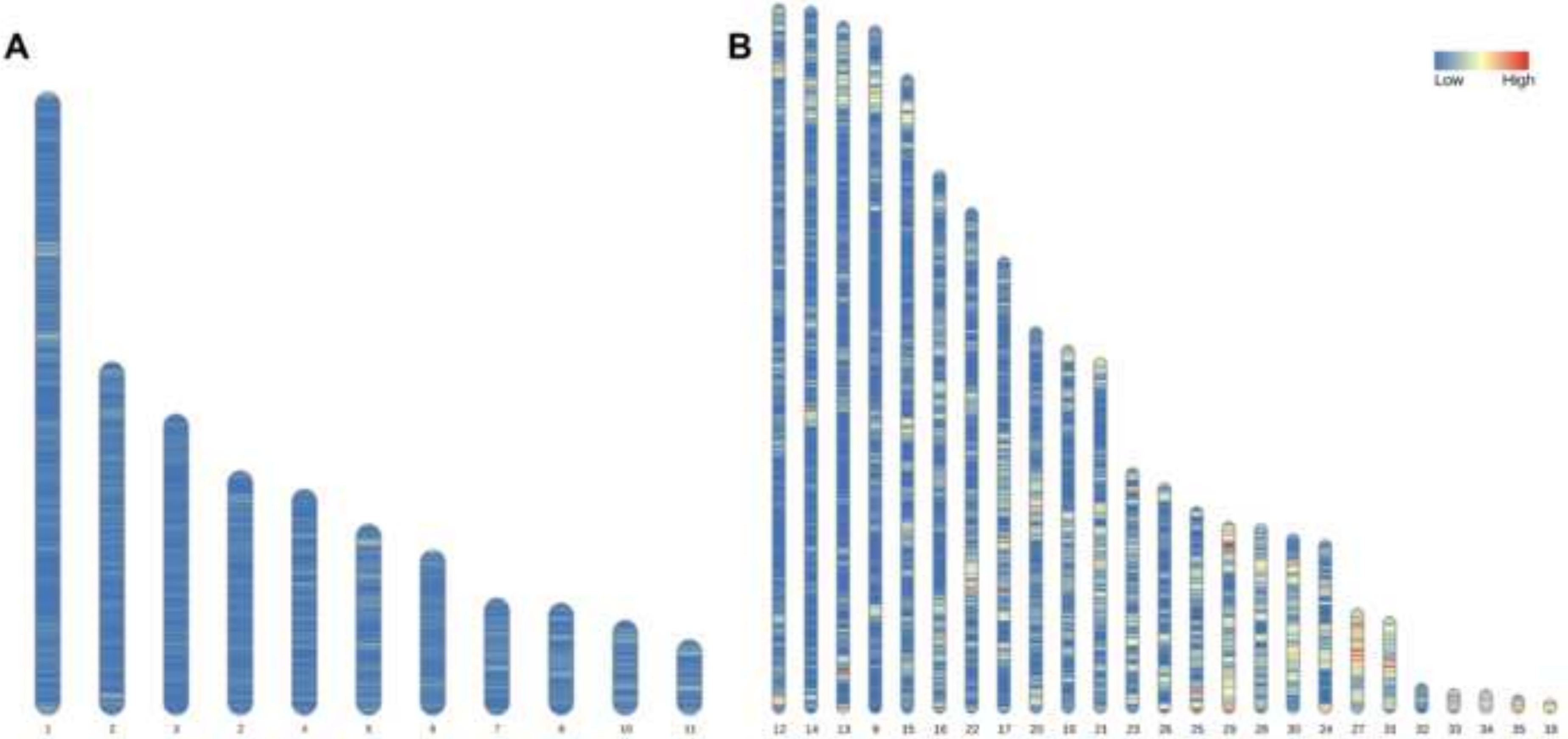

Figure 3

Click here to access/download;Figure;Figure3.tif

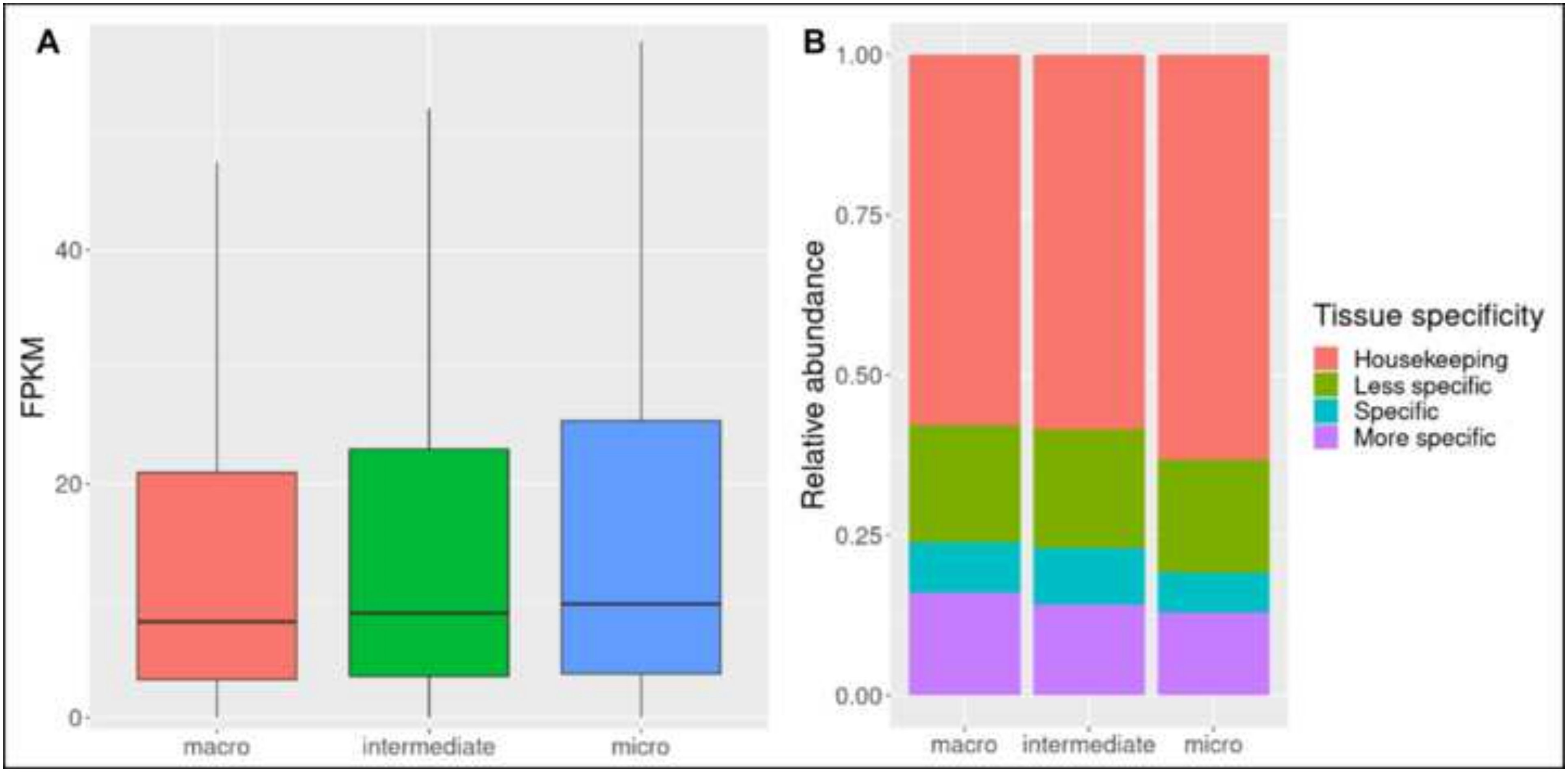

Figure 4

Click here to access/download;Figure;Figure4.pdf

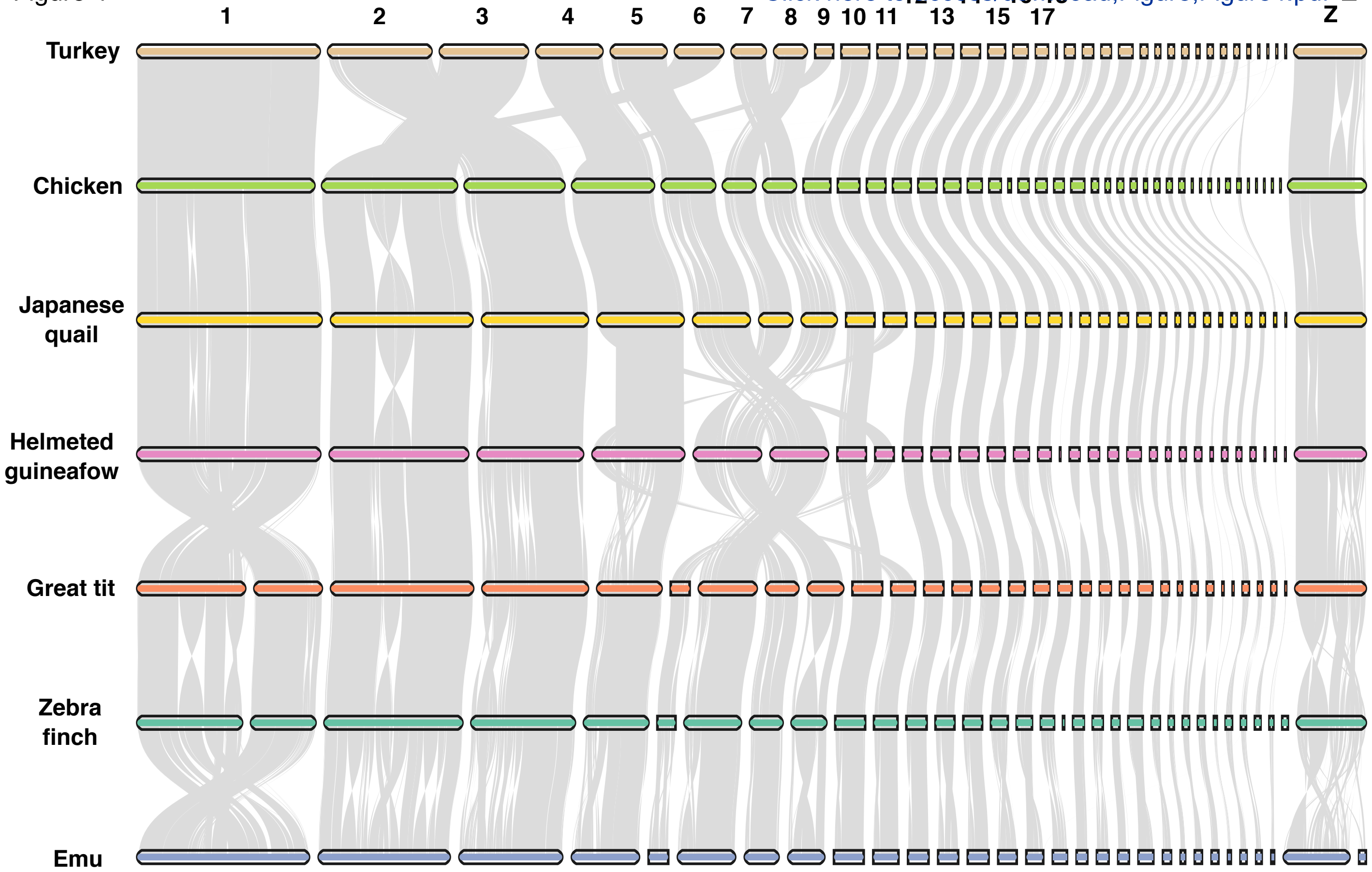

Figure 5

Click here to access/download;Figure;Figure5.pdf

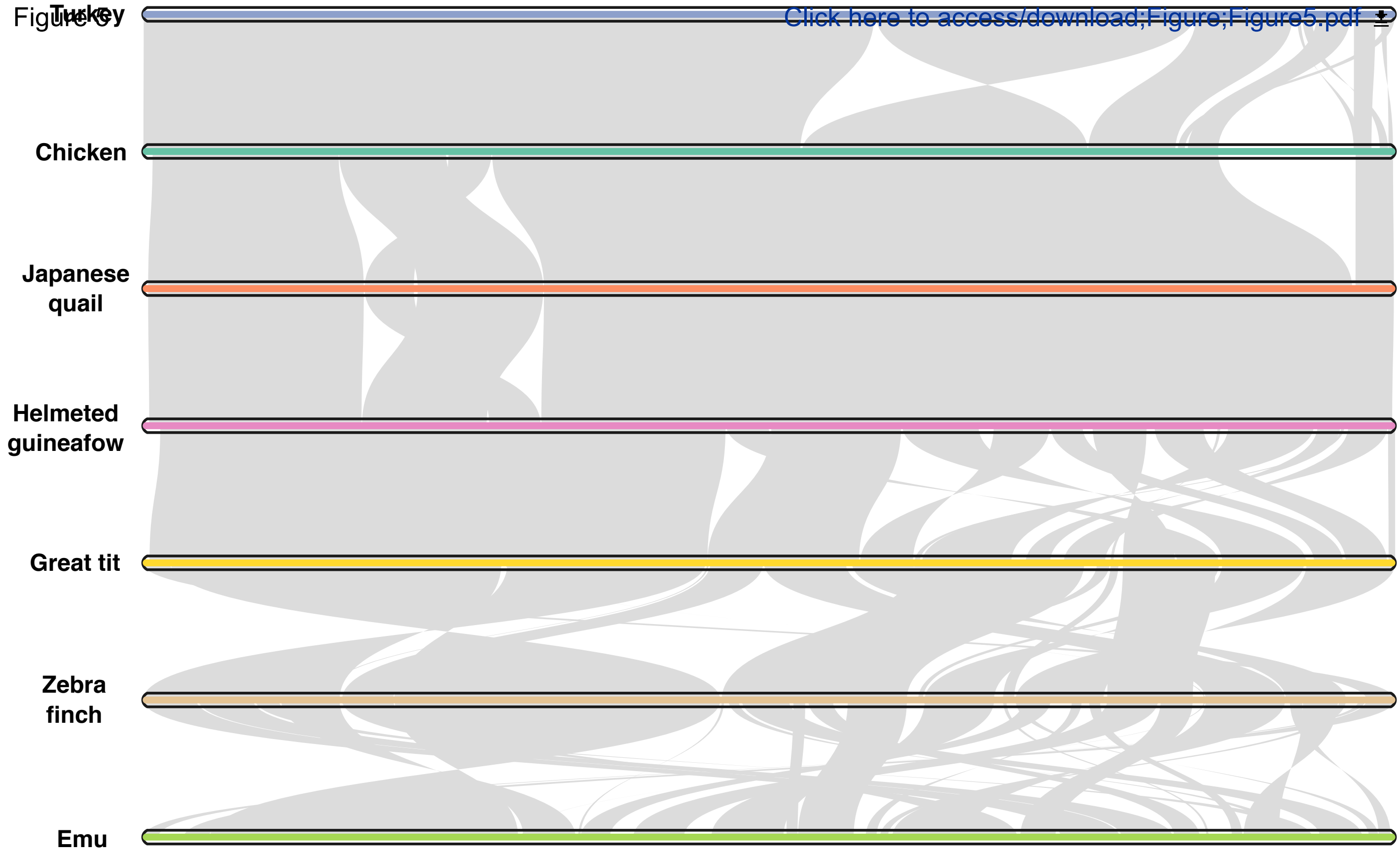

Supplementary File 1

Click here to access/download
Supplementary Material
SupplementaryFile1.docx

Supplementary File 2

Click here to access/download
Supplementary Material
SupplementaryFile2.xlsx

Supplementary File 3

Click here to access/download
Supplementary Material
SupplementaryFile3.xlsx

Supplementary File 4

Click here to access/download
Supplementary Material
SupplementaryFile4.pdf

Supplementary File 5

Click here to access/download
Supplementary Material
SupplementaryFile5.xlsx

Supplementary File 6

Click here to access/download
Supplementary Material
SupplementaryFile6.xlsx

Supplementary File 7

Click here to access/download
Supplementary Material
SupplementaryFile7.xlsx
