# Supplementary material for: A new haplotype-resolved turkey genome to enable turkey genetics and genomics research
Source: Gigascience. 2023 Jul 21;12:giad051. doi: 10.1093/gigascience/giad051 (PMC10360393; doi:10.1093/gigascience/giad051)
Supplement: giad051_GIGA-D-22-00193_Revision_3 [file giad051_giga-d-22-00193_revision_3.pdf]

GigaScience

A new haplotype-resolved turkey genome to enable turkey genetics and genomics research

--Manuscript Draft--

**Manuscript Number:**	GIGA-D-22-00193R3
**Full Title:**	A new haplotype-resolved turkey genome to enable turkey genetics and genomics research
**Article Type:**	Research
**Funding Information:**	Stichting voor de Technische Wetenschappen (14283) — Mr Martien A.M. Groenen
**Abstract:**	Background<br>The domesticated turkey (Meleagris gallopavo) is a species of significant agricultural importance and is the second largest contributor, behind broiler chickens, to world poultry meat production. The previous genome is of draft quality and partly based on the chicken (Gallus gallus) genome. A high-quality reference genome of Meleagris gallopavo is essential for turkey genomics and genetics research and the breeding industry.<br>Results<br>By adopting the trio-binning approach, we were able to assemble a high-quality chromosome-level F1 assembly and two parental haplotype assemblies, leveraging long-read technologies and genome-wide chromatin interaction data (Hi-C). From a total of 40 chromosomes (2n=80), we capture 35 chromosomes in a single scaffold, and show much improved genome completeness and continuity compared to the old assembly build. The three assemblies are of higher quality than the previous draft quality assembly and comparable to the chicken assemblies (GRCg7) shown by the largest contig N50 (26.6 Mb) and comparable BUSCO gene set completeness scores (96-97%). Comparative analyses confirms a previously identified inversion of around 19 Mbp on the Z chromosome not found in other Galliformes. Structural variation between the parent haplotypes were identified which pose potential new target genes for breeding.<br>Conclusions<br>We contribute a new high-quality turkey genome at chromosome-level, benefiting turkey genetics and other avian genomics research as well as turkey breeding industry.<br>.
**Corresponding Author:**	Martijn Derks, Ph.D.<br>Wageningen University & Research<br>Wageningen, NETHERLANDS
**Corresponding Author Secondary Information:**	
**Corresponding Author's Institution:**	Wageningen University & Research
**Corresponding Author's Secondary Institution:**	
**First Author:**	Carolina Pita Barros
**First Author Secondary Information:**	
**Order of Authors:**	Carolina Pita Barros
	Martijn Derks, Ph.D.
	Jeff Mohr
	Benjamin Wood
	Richard P.M.A. Crooijmans
	Hendrik-Jan Megens

Powered by Editorial Manager® and ProduXion Manager® from Aries Systems Corporation

	Marco C.A.M. Bink
	Martien A.M. Groenen, Professor
**Order of Authors Secondary Information:**	
**Response to Reviewers:**	Dear Editor,<br>We would like to thank again the reviewer for his/her final helpful comments. We have revised the manuscript according to the reviewers suggestions. We hope that this manuscript is now ready to be accepted for publication.<br>Best Regards,<br>The authors.<br><br>Reviewer #2: A new chicken genome has been published during the revision, I suggest the authors revise some parts of the manuscript: e.g. L66, L78, L83-85<br><br>Yes, we include the new chicken genome now in the manuscript. Hence, these parts have been revised. The new chicken genome is now discussed in the manuscript at lines L67-68, L86, L318, L365.<br><br>L103, please make it clear only the F1 was sequenced with long-read.<br><br>This is now made clear. Line 106-109.<br><br>L117-142, those results are very interesting, but perhaps the language can be more concise.<br><br>We shortened the paragraph and wrote more concise in this paragraph, L122-144.<br><br>L231-236, this paragraph is not important, please either move them to supplementary material or remove them. In general, this manuscript can be much more streamlined.<br><br>This paragraph has been removed. Moreover we have streamlined the manuscript better at 141-148, 171-172, 194-196.<br><br>L310-315, this part has also been reported by Huang et al. 2023 PNAS, so this is not a novel finding. Please either streamline or remove it.<br><br>We now indicate that our findings are similar to those reported in the study from Huang et al. “Similar to findings in the chicken genome by Huang et al. 2023 [8], microchromosomes showed on average higher gene expression than macro and intermediate chromosomes (Figure 3A)” Line 318.<br><br>L327, ref 36 is not a "recent" finding.<br><br>This has been changed.
**Additional Information:**	
**Question**	**Response**
Are you submitting this manuscript to a special series or article collection?	No
**Experimental design and statistics**<br><br>Full details of the experimental design and statistical methods used should be given in the Methods section, as detailed in our Minimum Standards Reporting Checklist. Information essential to interpreting the data presented should be made available in the figure legends.	Yes

Powered by Editorial Manager® and ProduXion Manager® from Aries Systems Corporation

Have you included all the information requested in your manuscript?	
**Resources**<br><br>A description of all resources used, including antibodies, cell lines, animals and software tools, with enough information to allow them to be uniquely identified, should be included in the Methods section. Authors are strongly encouraged to cite Research Resource Identifiers (RRIDs) for antibodies, model organisms and tools, where possible.<br><br>Have you included the information requested as detailed in our Minimum Standards Reporting Checklist?	Yes
**Availability of data and materials**<br><br>All datasets and code on which the conclusions of the paper rely must be either included in your submission or deposited in publicly available repositories (where available and ethically appropriate), referencing such data using a unique identifier in the references and in the "Availability of Data and Materials" section of your manuscript.<br><br>Have you have met the above requirement as detailed in our Minimum Standards Reporting Checklist?	Yes

Manuscript

Click here to access/download;Manuscript;Manuscript_Turkey_Genome_W

A new haplotype-resolved turkey genome to enable turkey genetics and genomics research

Carolina P. Barros[1], Martijn F.L. Derks[1*], Jeff Mohr[2], Benjamin Wood[2,3], Richard P.M.A. Crooijmans[1], Hendrik-Jan Megens[1], Marco C.A.M. Bink[4], Martien A.M. Groenen[1]

[1]Wageningen University and Research, Wageningen, Netherlands

[2]Hybrid Turkeys, Kitchener, ON, Canada

[3]School of Veterinary Science, University of Queensland, Gatton, QLD, Australia

[4]Hendrix Genetics Research, Technology & Services, Boxmeer, Netherlands

* Correspondence:

Martijn F.L. Derks

martijn.derks@wur.nl

ORCID iDs:

Martijn Derks [0000-0003-1317-5654]; Richard P M A Crooijmans [0000-0001-8108-9972]; Hendrik-Jan Megens [0000-0002-3619-7533]; Marco C A M Bink [0000-0002-1278-2092]; Martien A M Groenen [0000-0003-1317-5654].

Background

The domesticated turkey (*Meleagris gallopavo*) is a species of significant agricultural importance and is the second largest contributor, behind broiler chickens, to world poultry meat production. The previous genome is of draft quality and partly based on the chicken (*Gallus gallus*) genome. A high-quality reference genome of *Meleagris gallopavo* is essential for turkey genomics and genetics research and the breeding industry.

Results

By adopting the trio-binning approach, we were able to assemble a high-quality chromosome-level F1 assembly and two parental haplotype assemblies, leveraging long-read technologies and genome-wide chromatin interaction data (Hi-C). From a total of 40 chromosomes (2n=80), we capture 35 chromosomes in a single scaffold, and show much improved genome completeness and continuity compared to the old assembly build. The three assemblies are of higher quality than the previous draft quality assembly and comparable to the chicken assemblies (GRCg7) shown by the largest contig N50 (26.6 Mb) and comparable BUSCO gene set completeness scores (96-97%). Comparative analyses confirms a previously identified large inversion of around 19 Mbp on the Z chromosome not found in other Galliformes. Structural variation between the parent haplotypes were identified which pose potential new target genes for breeding.

Conclusions

We contribute a new high-quality turkey genome at chromosome-level, benefiting turkey genetics and other avian genomics research as well as turkey breeding industry.

Keywords: Genome assembly, Turkey genomics, trio-binning, animal breeding

Introduction

The domesticated turkey (*Meleagris gallopavo*, NCBI:txid9103) is an important agricultural species and the second largest contributor to world poultry production [1]. The turkey is a member of the Phasianidae family within the order Galliformes. Turkeys and chickens diverged about 25-40 million years ago [2]. Despite the relative long divergence time, the genome synteny and karyotype of both are highly conserved [3]. The turkey has 2n=80 compared to the chicken with 2n=78. The turkey karyotype consists of 7 macrochromosomes (>50 Mb), four intermediate chromosomes (>20 Mb, <40 Mb), and the rest being microchromosomes (<20Mb). The turkey karyotype is very similar to the chicken, except that chicken chromosome 2 is homologous to two turkey chromosomes (chromosomes 3 and 6) and chicken chromosome 4 is homologous to turkey chromosomes 4 and 9 [4]. Zhang et al. (2011) identified a large inversion on the Turkey lineage compared to chicken [5]. In addition, a high degree of synteny has also been observed between the chicken and turkey genomes [6].

The first turkey genome assembly (UMD2), published in 2010 [6], was among the first to be done almost exclusively based on second generation sequencing data, and by current standards would be considered of draft quality given the low contig N50 (27.1 kb) and lack of long read sequences [7]. The authors produced a chromosome level assembly and assembled 30 autosomal and two sex chromosomes. The assembly included linkage data based on a low-density genetic map and the placement of scaffolds to chromosomes relied considerably on conserved synteny assumptions with the better assembled chicken (*Gallus gallus*) genome. However, that version of the chicken genome had many microchromosomes missing altogether or only partially characterized. Avian microchromosomes have proved to be difficult to assemble even today [7]. Reliance on an incomplete chicken genome and the general difficulty in assembling the avian microchromosomes resulted in a poor representation of microchromosomes in that first UMD2 turkey genome. An updated version of

the turkey genome (Turkey_5.1; GCA_000146605.4) has been available since 2019, though it still shows low gene completeness and an incomplete set of microchromosomes.

The problems in characterizing microchromosomes are partly due to sequence characteristics, i.e., high GC and repeat content in microchromosomes, and partly due to their extremely small size and lack of genetic linkage group markers to differentiate the microchromosomes from other chromosomes [7]. Hence, ongoing efforts in producing high quality assemblies of the microchromosomes in many avian genomes have been unsuccessful due to above mentioned causes. However, a novel chicken reference genome containing all autosomes and both sex chromosomes was published, with all gaps closed except for the W [8].

High quality genome sequences are an essential resource for research and applications in the life sciences. In domestic animal breeding, genome wide marker panels are routinely used to support genomic selection and this significantly accelerates genetic progress [9]. An improved genome sequence facilitates ongoing genomic breeding programs. Furthermore, an improved genome assembly will greatly enhance functional interpretation of genomic variation in those breeding populations. For instance, improved annotation of (non)-coding genes benefits the functional interpretation of genome wide association studies (GWAS), and aids in identifying targets for gene editing [10].

Currently, more species in the Galliformes have high quality long-read based assemblies, including the Chicken [8], Japanese quail [11], Gunnison sage-grouse [12], and the helmeted guineafowl [13], allowing for comparative studies within the Galliformes and an in-depth comparison between the two most important avian agricultural species (chicken and turkey).

Third generation sequencing techniques have made it possible to produce high quality chromosome-based assemblies. The chicken individual broiler (GRCg7b) and layer (GRCg7w) assemblies and more recently the complete chicken genome assembly [8] have been produced from long read sequencing techniques. These new chicken assemblies show superior metrics of quality and completeness to previous genome assemblies. In this study we use a relatively new technique, the trio-binning approach, to construct high quality haplotype-resolved turkey assemblies [14]. A similar approach was also applied to create the recent chicken genome assemblies. In the trio-binning approach, short reads from each parent are used to resolve the F1 long reads into groups of long reads belonging to each parent. Each haplotype is then assembled independently resulting in three high quality genome assemblies, one from both parental haplotypes, and one F1 assembly (the primary assembly). This approach is especially powerful to assess structural variation between the parental haplotypes and works well with high heterozygosity rates as this aides in the resolution of the parent haplotypes in the F1 assembly.

In this study our aims were to use the trio-binning approach to produce a chromosome-level turkey assembly (F1), and two parental haplotype assemblies. We further aim to compare the two parental haplotypes to identify structural differences. A good reference genome is essential for many research and commercial applications. In this study we highlight how our new turkey genome can benefit both research and the breeding industry.

Results

Data and assembly of Mgal_WUR_HG_1.0

We used a trio-sequencing [14] approach to assemble the diploid genome of a male turkey. The F1 was sequenced with long reads, while the parents were sequenced with short reads to employ the trio-binning. The two parental animals derive from two distinct commercial lines from the breeding

company Hybrid Turkeys, a Hendrix Genetics company. The F1 animal was sequenced with a depth of 270x using PacBio single-molecule real-time (SMRT) sequencing technology. Approximately 12.25 million subreads were produced with a mean length of 22.5 kb, and N50 read length of 32.5 kb. Reads were assembled using wtdgb2 assembler [15] resulting in an initial assembly comprising of 315 contigs with an N50 of 26.68 Mb. The assembly was further scaffolded using Hi-C with HiRise [16]. Additional scaffolding was performed using SALSA (with Hi-C) [17] and Redundans [18]. The scaffolded assembly was subsequently polished with short reads (three rounds) to produce a final chromosome-level assembly consisting of 151 scaffolds and 232 contigs with a scaffold N50 of 70 Mbp and contig N50 of 26.55 Mbp (**Table 1**). This captures the majority of the chromosomes in a single scaffold and chromosome arms in a single contig (**Supplementary File 1: Table S1**). The Hi-C contact map can be found in **Supplementary File 1: Figure S1**.

<u>Telomeres and centromeres</u>

Telomeres and centromeres are generally enriched for simple repeats. Telomeric repeats (TTAGGG) were identified on the tail(s) of 18 chromosomes, supporting further completeness of the genome assembly (**Supplementary File 1: Figure S2**). A 41bp TM repeat was previously identified in turkey to be abundant in centromeric and (sub)telomeric regions especially on the microchromosomes [19]. Zhang et al. 2011 showed that there is a clear trend in turkey towards telocentric chromosomes, meaning that the centromere is located very close to the end of the chromosome and that the p arms would not, or barely, be visible. [5]. The only clearly metacentric chromosomes in turkey are chromosome 1 and the sex chromosomes. We predict that the centromere of chromosome 1 is located at 74.12-74.16 Mb enriched for TTAGGG and TM repeats (**Supplementary File 1: Figure S2**). Overall, we observe an enrichment of TM repeat clusters at the tails of chromosomes and in the microchromosomes. Furthermore, we identified clusters of TM repeats on one tail of marcochromosomes 2,3,4 which are likely telocentric with very short p-arms [5]. The enrichment of TM repeats at one chromosome tail indicate that the centromeric regions of these chromosomes likely

comprise of clusters of the 41bp TM repeat, while the other tail comprises telomeric repeats (**Supplementary File 1: Figure S2**). In addition, we identified clusters of TM repeats at the the tail(s) of intermediate chromosomes 8,9,10 and micro chromosomes 12,20,24,25,26,29,30,31 (**Supplementary File 1: Figure S2**). The intermediate chromosomes 7 to 14 are all predicted to be telocentric [5]. We found that chromosome 8,9,10, and 12 show enrichment of TM repeats on one tail of the chromosome, likely indicating centromeric regions at the chromosome ends. The majority of the michromosomes have at least telomeric repeats at one tail of the chromosome and several exhibit TM repeat clusters at the other end, supporting the likely telocentric structure of these microchromosomes.

<u>Haplotype assemblies</u>

As part of the trio-binning approach, both parental haplotypes were assembled with TrioCanu [14]. We were able to map 110X of the PacBio reads to parent 1 and 137X of the PacBio reads to parent 2, resulting in two parental haplotype assemblies with contig N50 of 9,174,806 bp and 19,855,975 bp for parents 1 and 2, respectively. We performed further scaffolding using LRscaf [20] and anchored the assemblies to the F1 assembly using RagTag [21]. The QV values indicate high quality and compleness of the assemblies evaluated by Mercury [22] (**Supplementary File 1: Table S2**). The final statistics of the assemblies are shown in **Table 1**.

Table 1: Assembly statistics. Summary statistics for the new Mgal_WU_HG_1.0 and parental assemblies, and comparison with previous turkey assembly (Turkey_5.1) and recent broiler assembly (GRCg7b).

	Mgal_WU_HG_1.0	Turkey_5.1	GRCg7b	Parent 1	Parent 2
**Total sequence length (bp)**	1,001,818,376	1,115,474,681	1,053,332,251	1,051,251,094	1,085,657,715
**Length ungapped (bp)**	1,001,806,830	1,080,180,254	1,049,948,333	1,050,601,018	1,085,166,758
**No. of scaffolds**	151	187,695	214	415	489
**No. of unplaced scaffolds**	115	187,662	172	379	453
**No. of chromosomes**	36	33	42	36	36
**Scaffold N50 (bp)**	70,339,173	3,898,092	90,861,225	71,046,337	71,481,950
**Scaffold L50**	5	80	4	4	4
**No. of contigs**	232	250,220	677	738	675
**Contig N50 (bp)**	26,554,504	27,076	18,834,961	9,174806	19,817,032
**Contig L50**	12	11,318	18	34	13

<u>Assembly accuracy and completeness</u>

The completeness and accuracy of the assemblies were assessed using BUSCO [23] and whole-genome alignments. All three assemblies contained over 96% of the expected avian and vertebrate gene sets, comparable to the GRCg6a and GRCg7b chicken genomes and covering 5.4% more gene space compared to the previous turkey genome assembly (Turkey_5.1), as shown in Table 2.

Table 2: Assembly completeness measured in BUSCO scores. Percentage of aligned genes for the vertebrae (n=3354) and avian (n=8338) gene set in the turkey and chicken assemblies.

	**Mgal_WU_HG_1.0**		**Turkey_5.1**		**GRCg7b**		**Parent 1**		**Parent 2**	
	Avian	*Vertebrate*	*Avian*	*Vertebrate*	*Avian*	*Vertebrate*	*Avian*	*Vertebrate*	*Avian*	*Vertebrate*
**Complete**	96.7	96.4	91.3	88.4	97.0	96.5	96.6	96.0	96.8	96.4
**Complete and single-copy**	96.4	95.9	91.1	87.9	96.7	95.7	94.8	93.9	94.1	93.2
**Complete and duplicated**	0.3	0.5	0.2	0.5	0.3	0.8	1.8	2.1	2.7	3.2
**Fragmented**	0.9	1.0	4.1	5.8	0.9	1.2	0.9	1.1	0.9	1.0
**Missing**	2.4	2.6	4.6	5.8	2.1	2.3	2.5	2.9	2.3	2.6

The turkey genome is highly congruent with the chicken genome (**Figure 1A**), indicating a high degree of conserved synteny. The main exception was a large ~19 Mbp inversion on the Z-chromosome (approximate coordinates ~44,493,000-63,950,000 bp). This inversion was also not present in the previous turkey build, Turkey_5.1, as seen in the alignment (**Figure 1B**). The alignment further shows that in the Turkey_5.1 assembly many contigs were placed in the wrong orientation (resulting in a "zigzag" alignment pattern).

Figure 1: Genome-wide alignment plots. A) Mgal_WU_HG_1.0 aligned with GRCg7b. Alignment shows high structural coherence between both genomes. B) Mgal_WU_HG_1.0 aligned with the old turkey genome build Turkey_5.1. Alignment shows multiple contigs that were placed in the wrong orientation in the previous Turkey_5.1 build.

Repeat and gene annotation

Repeat content

We annotated the repeats using a custom repeat library built using RepeatModeler [24]. Repeats were found to cover 10.45% of the genome. The most common were LINE elements, covering 6.35% of the genome. Furthermore, 0.76% of bases were DNA transposons, 0.53% long terminal repeats (LTRs),

and 1.58% low complexity and simple repeats. The remaining 1.23% of the repeats remained unclassified. A complete overview of the repeats per chromosome is listed in **Supplementary File 2**.

Gene Annotation

The Ensembl annotation pipeline was used to annotate Mgal_WU_HG_1.0 [25]. The present annotation includes fewer annotated genes compared to Turkey_5.1 and the chicken annotations, but does include more non-coding genes, as shown in Table 3. As expected, microchromosomes show higher gene density compared to macro and intermediate chromosomes (P<0.00001, Figure 2). The density generally increases with decreasing microchromosome size.

Table 3: Annotation statistics for the turkey (Mgal_WU_HG_1.0, Turkey_5.1) and chicken (GRCg6a, GRCg7b) genomes. BUSCO scores show percentage of aligned proteins for the avian (n=8338) and vertebrate (n=3354) protein set in the turkey and chicken assemblies.

Annotation	Mgal_WU_HG_1.0	Turkey_5.1	GRCg6a	GRCg7b
Coding genes	16,127	16,226	16,878	17,007
Non-coding genes	7,736	1,585	7,166	13,040
Small non-coding genes	504	543	1,525	1,089
Long non-coding genes	7,228	1,038	5,506	11,946
Misc non-coding genes	4	4	135	5
Pseudogenes	45	159	312	61
Gene transcripts	53,441	30,708	39,288	72,689
**Completeness BUSCO (avian / vertebrate)**				
% Complete	97.9/97.0	87.5/80.8	95.1/93.8	98.3/97.0
% Fragmented	0.6/1.1	5.2/10.3	2.0/2.9	0.5/1.0
% Missing	1.5/1.9	7.3/8.9	2.9/3.3	1.2/2.0

We identified chicken and Turkey_5.1 homologues of the Mgal_WU_HG_1.0 genes (**Supplementary File 1: Table S3**). The majority of the protein-coding genes have a 1:1 orthologue in the Turkey_5.1 (82.4%) or in the GRCg6a (86.3%) genome assemblies. The higher number of genes orthologous to the most recent chicken assemblies supports our assertion of a significant improvement of assembly and annotation quality compared to Turkey_5.1

Figure 2: Ideogram showing gene density. A) macro (1-6, Z) and intermediate chromosomes (7,8, 10, 11). B) micro chromosomes (9,12-35) in the Mgal_WU_HG_1.0 genome.

Gene family analysis

OrthoFinder [26] was used to infer orthogroups from the following set of bird species - turkey, chicken , Japanese quail (*Coturnix japonica*) [11], helmeted guineafowl (*Numida meleagris*) [13] and zebra finch (*Taeniopygia guttata*) [27]. From the 16,127 protein-coding genes in the Mgal_WU_HG_1.0 gene set, 98% were found to be in an orthogroup. This was the highest percentage of any of the species tested (**Table 4**). Of the 15,417 orthogroups found, 91% include Mgal_WU_HG_1.0 genes. There are also 10 orthogroups that contain only Mgal_WU_HG_1.0 genes, of which two have homologs in the nr database (*MANBAL* and *POL3*) (**Supplementary File 1: Table S4**).

Table 4: Number of orthogroups found and proportion of genes assigned to each orthogroup per species. Species included: turkey (Mgal_WU_HG_1.0, Turkey_5.1), chicken (GRCg6a, GRCg7b), Japanese quail (Coturnix_japonica_2.0), helmeted guineafowl (NumMel1.0), and zebra finch (bTaeGut1_v1.p)

Species assembly	Mgal_WU_HG_1.0	Turkey_5.1	GRCg6a	GRCg7b	Coturnix_japonica_2.0	NumMel1.0	bTaeGut1_v1.p
**No genes**	16127	16226	16878	17007	15732	15661	16619
**No genes in orthogroups**	15843	15365	16359	16583	15342	15306	15971
**No unassigned genes**	284	861	519	424	390	355	648
**Genes in orthogroups (%)**	98.2	94.7	96.9	97.5	97.5	97.7	96.1
**Unassigned genes (%)**	1.8	5.3	3.1	2.5	2.5	2.3	3.9
**No orthogroups containing species**	14033	13350	13800	14156	13801	13695	13390
**Orthogroups containing species (%)**	91	86.6	89.5	91.8	89.5	88.8	86.9
**No species-specific orthogroups**	10	63	23	24	4	7	110
**No genes in species-specific orthogroups**	50	178	120	95	9	67	428
**Genes in species-specific orthogroups (%)**	0.3	1.1	0.7	0.6	0.1	0.4	2.6

<u>Contractions and expansions in orthologous groups</u>

While most orthogroups studied showed no change in the copy-number of protein coding genes, 71 groups showed expansions or contractions of gene families predicted using CAFE5 software [28] (61 expansions, 10 contractions) (**Supplementary File 3**). Expanded orthogroups contained proteins involved in important processes in bird development and growth, including gene families involved in cytoskeleton (proteins for feather keratin) (OG0000026, OG0000030), reproduction (involved in spermatogenesis/spermiogenesis) (OG0000005), response to stress (OG0000111), and immunity (OG0000001). Orthogroups OG0000005 shows an expansion of the turkey PHD finger protein 7 (PHF7) gene, which has been shown to be a highly duplicated gene family in the chicken genome [29]. The

contracted gene families include one immunoglobin (OG0000001), a homeobox B8 (OG0000526) gene family, and an olfactory receptor gene family (OG0000407) .

Structural variation between parental haplotypes

Structural variation

The F1 and the paternal haplotypes are completely co-linear **Supplementary File 1: Figure S3**). There are no large structural differences (>1 Mbps) between the two parental haplotypes except for a 1.47 Mbp inversion on chromosome 1 (74.28 – 75.74 Mb, **Supplementary File 4**) comprising 25 protein coding genes and 15 lncRNA genes. This inversion is in the centromeric region of chromosome 1, shown by an excess of telomeric and TM repeats between 74.12-74.16 Mb. **Table 3** shows an overview of the number and cumulative length of each type of structural variation.

Table 5: Structural variation between the two parental haplotype assemblies. The parent 1 assembly was used as reference and the parent 2 assembly used as the query. Copygain: Copy gain in the query genome, copyloss: copy loss in the query genome.

Variation type	Count	Length Parent1	Length Parent2
Syntenic regions	85	990,480,776	989,217,672
Inversions	19	1,728,932	1,525,862
Translocations	68	895,801	867,550
Duplications	397	870,354	3,179,922
Copy gains	40	-	305,148
Copy losses	58	1,268,056	-

Copy gains are regions that have extra copies in the parent2 haplotype, while copy losses show regions with fewer copies in parent2 (and thereby higher copies in parent1). The distribution of copygains and copylosses are in **Supplementary File 1: Figure S4.** In total, 231 large structural variations (>10 kb) have been identified between the two parental haplotypes (**Supplementary File 5)**. From these, 81 affect the coding sequence of protein coding genes, of which 40 have a 1:1 ortholog in chicken. Interestingly, an inversion affecting the coding sequence of the *BLB2* gene, this gene is duplicated

within MHC-B region in chicken playing a crucial role in disease resistance or susceptibility [30] was found in parent 2 compared to parent 1 (**Supplementary File 1: Figure S5**). We further identified duplications in the parent 2 haplotype comprising the *TRIM36*, *GRIA2* and *MAN2B2* gene. Specifically, the parent 2 haplotype exhibits a 20 kb duplication of the 3' end of *MAN2B2* (**Supplementary File 1: Figure S6**), a gene which in pigs is associated with ovulation rate [31]. In addition, a 34 Kbp duplication affecing the *GEMIN8* gene in parent 1 was identified (**Supplementary File 1: Figure S7**). The *GEMIN8* gene product is part of the survival motor neuron (SMN) complex. Moreover, a 53 Kbp duplication was found affecting the 3' end of the *RIMKLB* gene (**Supplementary File 1: Figure S8**), resulting in a copy number of 3 in parent 1 but a copy number of >5 in parent 2. In addition, a 100 kb translocation that comprises the *RALYL* gene was identified. The translocated region is found at around 68.2 Mbp on chromosome 5 in parent 1, while it is found at a position around 90.1 Mbp on the same chromosome in parent 2. Finally, an inversion on chromosome 30 of length 187 kb comprises two protein coding genes and one lncRNA.

A full overview of structural variation between the parental haplotypes is provided in **Supplementary File 5.**

Loss of function variation

The most common effect of selection is to alter gene expression, leading to phenotypic changes. However, a small proportion of phenotypic variation is due to impaired gene functioning [32]. We assessed the presence of loss-of-function variation (LoF), specifically stop-gained variants affecting genes in either of the two parental haplotypes (**Supplementary File 6**). In total, 138 stop-gained variants affecting 92 genes between the parent1 and parent2 haplotypes were identified. Genes carrying LoF mutations that are especially noteworthy include the *RYR2* gene, which is affected by four LoF variant in parent 2, likely leading to an impaired RYR2 protein. Mutations in the *RYR2* gene are associated with stress in broiler chickens [33]. A second gene worth highlighting is *LRRC41* which, in the parent 2 haplotype, contains a stop-gained variant. Knockouts of this gene lead to increased lean

body mass in mice and hence this gene poses an interesting candidate for selection for body weight in turkey [34].

Mapping of SNP-chip markers

SNP-chips are useful to study variation (single nucleotide polymorphisms, SNPs) between individuals and are widely applied in genomic selection. We mapped SNP-chip markers from a 65K SNP array (64,800 SNPs; Illumina, Inc.) to Mgal_WU_HG_1.0 (**Supplementary File 1: Table S5**) using a custom SNP mapping pipeline (see methods). We mapped 64,536 (99.4%) of the markers to Mgal_WU_HG_1.0. From these, 1,532 markers that were located on unplaced contigs in Turkey_5.1 are now mapped to specific chromosomes in Mgal_WU_HG_1.0, and 415 markers were placed on the new chromosomes 31-35, indicating a higher completeness. More specifically, we were able to place a significant number of new markers, especially on chromosomes 1 (412), 27 (120), 31 (192), and Z (594).

Distinct genomic landscapes of turkey micro and macrochromosomes

Avian genomes are known to vary greatly in genomic features, especially between the micro and macrochromosomes [35]. We evaluated the genomic landscape of the turkey chromosomes in terms of repeat content, gene density, and gene expression between macro (>40 Mbp), intermediate (>40 Mbp, <20 Mbp), and micro (<20 Mbp) chromosomes. We found that the repeat content of each repeat class in macro, micro and intermediate chromosomes varied highly along the chromosome (**Supplementary File 1: Figures S9-S16, Supplementary File 2**). Macrochromosomes are enriched for DNA transposons ($p<0.01$) and LINE elements ($p=0.0281$) compared to the intermediate and microchromosomes (**Supplementary File 1: Figure S10-S11**). In addition, LINE CR1 elements are especially enriched at the tails of macrochromosomes. Microchromosomes are enriched for low complexity ($p<0.01$, **Supplementary File 1: Figure S11**), simple ($p<0.01$, **Supplementary File 1: Figure**

S11), and unknown repeats (p=0.062, **Supplementary File 1: Figure S16**) compared to intermediate and macrochromosomes, the latter especially at the tails of the chromosomes.

In order to assess whether there was a distinction between the type of genes (e.g. tissue specific or housekeeping) in chromosome types, we analysed RNA-seq datasets from 16 tissues (mapping rates in **Supplementary File 1: Table S6**). Similar to findings in the chicken genome by Huang et al. 2023 [8], microchromosomes showed on average higher gene expression than macro and intermediate chromosomes (**Figure 3A**), as well as having a higher relative abundance of housekeeping genes, defined here as genes expressed in at least 13 out of the 16 studied tissues included in this study (**Figure 3B**).

Figure 3: A) Overview of gene expression in macro, intermediate and micro chromosomes. B) Relative abundance of tissue specific genes in each chromosome class. Microchromosomes show higher relative abundance of housekeeping genes when compared with macro and intermediate chromosomes. Number of tissues tested: 16. Housekeeping genes: expressed in at least 13 tissues; less specific genes: expressed in at least 5 tissues and fewer than 13 tissues; specific: expressed in 2 to 5 tissues; more specific: expressed in one or two tissues.

Conserved synteny within the Galliformes clade

We performed synteny analysis to assess chromosomal and structural rearrangements within a wide range of avian species. Four Galliformes were included: turkey, chicken, Japanese quail, and helmeted guineafowl. Furthermore, two Passeriformes, zebra finch and great tit, and emu, a species from the Casuariiformes order were included. The multi-species synteny plot shows a high degree of synteny between the avian species both on the macro and the microchromosomes, despite the large evolutionary distances (**Figure 4**), supported by other studies [36, 37].

Figure 4: Chromosomal rearrangements across several avian species. Pairwise synteny comparison across 7 birds shows several chromosomal rearrangements. Grey segments represent conserved synteny. Species: turkey (*Meleagris gallopavo*), chicken (*Gallus gallus*), Japanese quail (*Coturnix japonica*), helmeted guineafowl (*Numida meleagris*), great tit (*Parus major*), zebra finch (*Taeniopygia guttata*), and emu (*Dromaius novaehollandiae*).

Of all chromosomes, it is evident that especially the Z chromosome has been prone to large chromosomal rearrangements between avian orders (**Figure 5**) [38]. Interestingly, we found a large inversion of around 19 Mbp on the turkey Z chromosome not found in the other Galliformes and songbirds [5] (**Supplementary File 1: Figure S17**). The inversion was supported by a normal alignment at the approximate breakpoints (**Supplementary File 1: Table S7 - Figure S18**) and the HiC contact map confirmed the accuracy of the assembly (**Supplementary File 1: Figure S19**). This is especially striking since rearrangements on the Z chromosome are uncommon within the Galliformes. One region at the tail of the chicken Z chromosome lacks synteny with other Galliformes altogether [39]. This region is enriched in repeat sequences in both chicken and turkey (**Supplementary File 1: Figure S20**), as described previously in Bellott et al 2017 [39].

Figure 5: Chromosome Z rearrangements across 7 avian species. Pairwise synteny comparison of the Z chromosome across avian species reveals a large inversion in turkey. Grey segments represent conserved synteny. Species: turkey (*Meleagris gallopavo*), chicken (*Gallus gallus*), Japanese quail (*Coturnix japonica*), helmeted guineafowl (*Numida meleagris*), great tit (*Parus major*), zebra finch (*Taeniopygia guttata*), and emu (*Dromaius novaehollandiae*)

Discussion

We present a new, chromosome-level, high quality reference assembly for *Meleagris gallopavo*, Mgal_WU_HG_1.0. The trio binning approach has been proven to be a robust method to characterize the two haplotypes of F1 individuals [14]. The chromosome level assembly (**Supplementary File 1: Table S1**) presented in this study confirms the value of this method in not only providing a quality assembly but also in uncovering structural genomic variation. The Mgal_WU_HG_1.0 assembly is a

large improvement over the previous turkey assembly, Turkey_5.1 [6]. The assembly is now comparable in quality and completeness to the chicken GRCg7 genomes, but not as complete as the latest chicken genome [8]. Note that we sequenced a male animal and we are therefore lacking the W chromosome. One major limitation of previous turkey assemblies was that they relied on assumptions on high turkey-chicken retained synteny to achieve a chromosome-level assembly. Such assumptions can result in bias, especially when comparing turkey to chicken. Mgal_WU_HG_1.0 does not rely on such comparisons.

Combining long reads and genome-wide chromatin interaction data (Hi-C) enables the capture of chromosome arms in a single contig, resulting in a highly continuous and contiguous chromosome-level assembly. Furthermore, long reads can span long repetitive regions including DNA transposons and LINE elements, as well as large structural variants. We observe an enrichment of telomeric and TM repeats at the tails of chromosomes, likely indicating telomeric and centromeric regions, as the majority of the turkey chromosomes is likely telocentric [5]. We show that the centromeres located at chromosome ends mostly comprise of TM repeat clusters. Thanks to these recent sequencing technologies, we are able to correct a number of wrongly oriented contigs in Turkey_5.1, a phenomenon often observed in short-read based assemblies. The improvements in genome quality, completeness and continuity allow for a more thorough annotation of repeats and gene models. The increase in complete BUSCO genes in Mgal_WU_HG_1.0, compared to Turkey_5.1, indicates a much-improved gene space in the current genome assembly, comparable to the latest chicken genome builds.

Improving genome assemblies improves all analyses that depend on them. One of the reasons to improve the turkey assembly was to better map SNP-chip markers to the genome. SNP-chips are widely used in genomic selection and a better genome representation and gene annotation directly impacts its use for breeding. Specifically, the new turkey genome build overcomes the lack of SNPs

mapped to gene-dense microchromosomes, as 85.3% of the SNP markers previously mapped to unplaced scaffolds on Turkey_5.1 are now mapped to chromosomes on Mgal_WU_HG_1.0, especially improving the representation of microchromosomes 31 to 35.

Turkey breeding is done on pure elite lines which can be selected for different purposes. In our study, one parent was from a female breeding line, with more focus on egg production and conformation, whereas the other parent was from a male breeding line focussing on growth and production traits. In producing a commercial product, lines are crossed to produce hybrid offspring that shows the benefit of the breeding goals of both parental lines. In addition, the hybrid offspring benefits from hybrid vigour, resulting from two relatively differentiated lines. For the trio-binning method, having parents that are genetically distinct helps in resolving the haplotypes. Nevertheless, in this study, we present two high quality parental haplotype assemblies where the low heterozygosity of the parents presented no obstacle to resolving the parental haplotypes.

Interestingly we found specific structural variation in *BLB2* (inversion), *TRIM36*, *GRIA2, MAN2B2 (all duplications)* and a loss-of-function variant in the *LRRC41* gene in the parental haplotype from the male line. An additional duplication of the *GEMIN8* gene was identified in the parental haplotype from the female line. The *BLB2* gene plays an important role in the presentation of extracellular antigen and initiation of an immune response [30]. However the consequence and frequency of the inversion in the parental line remains unclear. The *TRIM36* gene is associated with the spermatozoa acrosome reaction in mice and knockouts are incapable of in-vitro fertilization [40]. Hence, the duplication of this gene in the paternal line might have implications on male fertility that requires further study. The *GRIA2* gene is a excitatory neurotransmitter associated with various neurodevelopmental disorder in humans [41]. The *MAN2B2* gene is associated with ovulation rate in pigs [31] while in human this gene is associated with a disorder of glycosylation [42]. However, the role of this gene and its duplications in avian species remains unclear. The *GEMIN8* gene encodes a protein that is part of the SMN complex,

which is necessary for spliceosomal snRNP assembly in the cytoplasm and pre-mRNA splicing in the nucleus [43]. The *LRRC41* gene is likely knocked-out in the male parental haplotype. Knockout mice of the *LRRC41* gene show increased circulating calcium and glucose levels and increased lean body mass [34]. Therefore this gene is an interesting target gene for breeding and the identified stop-gained mutation likely causes a loss-of-function of the protein in the parental line, thereby enhancing growth. However, to further validate this hypothesis we need to evaluate the frequency in the population and functional consequence of the variant.

Among the remaining challenges in variation analysis is the characterization of structural variants. The challenge is two-fold. First, these large-scale variants are often not robustly detected using short-read sequencing. Second, individuals usually have sequence that is population specific, and which may not be present in a reference assembly. This can make such large insertions hard to characterize, even by re-sequencing. In the process of assembling Mgal_WU_HG_1.0 we now have reference assemblies for two distinct breeding lines, which should greatly aid in variation analysis. Even though such large structural variants appear to be uncommon between breeding lines, we demonstrate how genes potentially important in breeding may be affected. These genes can be further prioritized in routine genomic breeding practice.

As more genomes are characterized with high accuracy and at a chromosome-level, comparative genomics is increasingly used to study the function of genes and variants, including copy number variants. The new Mgal_WU_HG_1.0 genome assembly was applied to identify orthogroups that have expanded or contracted in turkey compared to other avian species. Expanded orthogroups included various distinct keratin families, encoding major structural proteins of feathers and claws [44]. One gene family comprising the PHD Finger Protein 7 (PHF7) was significantly expanded in turkey. *PHF7* acts during spermiogenesis for histone-to-histone protamine exchange and is a determinant of male fertility in Drosophila and mouse [45], and highly expressed in rooster testis [46]. This gene family was

found to be expanded in chicken as well, with distinct gene clusters on five chromosomes [29]. In addition, genes related to immunity and response to stress are expanded in turkey. Further research is needed to disentangle the exact function of these complex gene families.

A characteristic of avian genomes is that they comprise a huge range of chromosome sizes. Interestingly, bird genome organization may be ancestral to all vertebrates [47]. Among the peculiar outcomes is a wide range in e.g. recombination rates, GC-bias, gene densities and variation density throughout the genome [35]. The distinct nature of these features is particularly difficult to study in microchromosomes as they have proven so difficult to characterize. The distinct patters of both gene density and repeat content between the macro and microchromosomes have been described previously by Kapusta et al. 2017 [48]. The Mgal_WU_HG_1.0 assembly though, has a better representation of the microchromosomes, allowing a better understanding of functional aspects of genes and other genome elements. We have shown that the microchromosomes have a unique repeat landscape enriched for low complexity, simple, and unknown repeats, especially at the tails of the chromosomes. Together these efforts provide new insights in microchromosome composition and evolution.

Bird genomes have very high retained synteny [49]. This pattern was confirmed in our analysis of the conserved synteny between several Galliformes (turkey, chicken, Japanese quail, helmeted guineafowl) and three outgroups (zebra finch, great tit, emu). Despite the long divergence time that separates turkey and chicken [2], both species have relatively similar karyotypes confirmed by the high structural continuity and relatively little rearrangements between the two birds, even in the microchromosomes. The latter is noteworthy because of the very high recombination rates generally observed in microchromosomes [50], which would suggest that a higher rate of chromosomal rearrangements might be expected but is not observed. Expanding observations to other Galliformes

suggest similar degrees of conserved synteny, although comparisons for micro-chromosomes are less accurate due to the more incomplete assembly of these other Galliform species

The Z chromosome presents a moderate yet striking deviation from the observed evolutionary stability. This chromosome exhibits a few rearrangements within the Galliformes and, in line with the findings of Zhang et al. (2011), we observed and validated a large inversion in the turkey Z chromosome [5]. As with the Mgal_WU_HG_1.0 assembly the exact breakpoints of this 19 Mbp inversion on the Z chromosome can now be pinpointed. This inversion is unique for the turkey lineage, and not found in any of the other Galliformes.

In conclusion, the new turkey genome here presented (Mgal_WU_HG_1.0) (and the two parental haplotype assemblies) represents a substantial improvement over the previous assembly and is an important resource with many applications in research and in the turkey breeding industry.

Methods

Data and Assembly

To create a high-quality chromosome level genome assembly of *Meleagris gallopavo*, three individuals were sequenced using the trio binning approach - two parents and one F1. The two parents come from two distinct commercial lines from Hendrix Genetics, one male line (parent1) and one female line (parent2). The F1 turkey was sequenced by Dovetail Genomics using PacBio single-molecule real-time (SMRT) sequencing technology (PacBio Sequel System, RRID:SCR_017989) with a total depth of 270X. We generated short read sequencing data from the F1 (90.4X coverage) and both parents (35.4X, and 39.7X coverage) on an Illumina HiSeq 4000 (HiSeq 4000 System, RRID:SCR_016386). In addition, Hi-C data was generated with a coverage of 32X. An initial assembly was created by Dovetail Genomics using wtdgb2 (WTDBG, RRID:SCR_017225) [15], polished with the PacBio long reads using

wtpoa-cns, and scaffolded using the Dovetail *De Novo* Assembly Process, which uses Chicago® and Dovetail Hi-C proximation ligation methods and the HiRise™ scaffolder as described in [16].

Polishing

Pilon v1.23 (Pilon, RRID:SCR_014731) [51] was used to polish SNPs and indels based on the short Illumina reads from the F1 (twice with parameters--diploid --mindepth 0.7 --fix bases --changes), and indels with the Illumina reads from parent2 because of the higher coverage compared to parent1 (--fix indels).

Scaffolding

We scaffolded the F1 assembly received by Dovetail Genomics using the Hi-C reads and the PacBio long reads, both from the F1. The Hi-C reads were mapped to the polished assembly based on the Arima Mapping pipeline [52], using BWA-MEM v0.7.17 (BWA, RRID:SCR_010910) [53] with default parameters. The filter_five_end.pl script was used to filter and keep the 5’-end. After filtering, the reads are sorted and paired using the two_read_bam_combiner.pl script. This results in a sorted, paired-end BAM file that has been filtered by mapping quality (mapping quality filter =10). Picard Tools v2.23.4 (Picard, RRID:SCR_006525) [54] - AddOrReplaceReadGroups and MarkDuplicates was used to add a read group and remove duplicates. The mapped Hi-C reads were used to scaffold the assembly with SALSA v2.2 (SALSA, RRID:SCR_022013) [17], which is a scaffolder that uses long range contact information (Hi-C) with parameters -e "GATC". Redundans v0.14a [18] was used to scaffold the assembly with the PacBio reads with length >40 Kbp and remove redundant contigs from the final assembly. The parameters -l <long reads> --nogaplosing --noscaffolding were used (--noscaffolding skips short read scaffolding). QV values are calculated using Merqury (Merqury, RRID:SCR_022964) [22].

Hi-C validation - mis-assemblies

To validate our F1 assembly and look for mis-assemblies we used Hi-C contact maps. Juicer v1.6 (Juicer, RRID:SCR_017226) [55] was used to generate Hi-C contact maps from the Hi-C reads (**Supplementary File 1: Figure S1**) and 3D-DNA v180922, a 3D de novo assembly pipeline (3D de novo assembly, RRID:SCR_017227), to scaffold our assembly. Juicebox v1.11.08 (Juicebox, RRID:SCR_021172) [56] was used to visualize the Hi-C contact map and identify mis-assemblies. Each breakpoint in the macrochromosomes was manually checked with Juicebox and JBrowse 1.16.9 (JBrowse, RRID:SCR_001004) [57] to visualize the PacBio read coverage at the breakpoints.

Haplotype assemblies using trio-binning

TrioCanu (a module from the Canu assembler, v2.1.1) (Canu, RRID:SCR_015880) [14]was used to bin the parental reads to construct parental haplotype assemblies. TrioCanu was run with the short reads from each parent and the F1 PacBio reads with the following options: -p asm genomesize-1.1g. The corrected reads from TrioCanu were mapped to the Triocanu assembly with Minimap2 v2.17-r941 (Minimap2, RRID:SCR_018550) [58], options -x map-pb (mapping PacBio). LRScaff v1.1.10 [20] was used to scaffold each parent assembly. For both parents the scaffolding was done with these parameters: min_contig_length = 500, identity = 1, min_overlap_length = 400, max_overhang_length = 500, max_end_length = 500, min_supported_links = 2, iqr_time = 3. Duplicated sequences were removed using seqkit. RagTag v1.1.1 [21] was used for reference-guided scaffolding of each parental assembly, using the F1 assembly as reference. The scaffold module from RagTag was used with default parameters.

Completeness

BUSCO

BUSCO v4.1.2 (BUSCO, RRID:SCR_015008) [23] was run to assess the completeness of the assembly in terms of gene space. BUSCO was run in the genome mode (-m genome) and with the vertebrae (vertebrata_odb10) and aves (aves_odb10) datasets (using the flag -l <dataset>).

Genome comparison - alignment

Genome assembly alignments were generated using D-GENIES v1.3.0 (D-GENIES, RRID:SCR_018967) [59], using minimap2 as the aligner. The chromosomes were sorted on length, and noise (short repeat alignments) was removed from the alignment plot.

Structural variation (parents)

Structural variation between the two parental haplotypes was discovered using SyRI v1.5.4 [60]. First, we aligned the two haplotype assemblies using minimap2 with settings -ax asm5 –eqx. Next, we used SyRI to identify structural variation using the minimap2 alignment. Results were plotted using plotsr tool v0.5.3 [61]. Large structural variants were manually validated in JBrowse 1.16.9 [57].

Remapping and variant calling

The short Illumina reads from the F1 individual were mapped back to the assembly using BWA-MEM v0.7.17 (BWA, RRID:SCR_010910) [53]. Samblaster v0.1.26 (SAMBLASTER, RRID:SCR_000468) [62] was used to mark duplicates and Samtools v1.14 (SAMTOOLS, RRID:SCR_002105) [63] to sort and index the BAM files. Freebayes v1.3.1 (FreeBayes, RRID:SCR_010761) [64] was used for variant calling with: --use-best-n-alleles 4 --min-base-quality 10 --min-alternate-fraction 0.2 --haplotype-length 0 --ploidy 2 --min-alternate-count 2. The vcffilter module from vcflib v0.00.2019.07.10 [65] was used to discard variants with low phred quality score (<20). Tabix, a module from htslib v1.9 (SAMTOOLS, RRID:SCR_002105) [65] was used to index the VCF files. The stats module from BCFtools v1.9 (SAMtools/BCFtools, RRID:SCR_005227) [66] was used to compute summary statistics of the variant

calling. The same process was followed to call variants for each parent. Alignment quality control statistics were computed with QualimMap v.2.2.2-dev (QualiMap, RRID:SCR_001209) [67].

SNP-Chip

In order to map SNP markers from the 65K single nucleotide polymorphism (SNP) array (65,000 SNP; Illumina, Inc.) to the new genome build we first aligned the two genome builds (Turkey_5.1 and Mgal_WU_HG_1.0) using nucmer v4.0.0rc1 (MUMmer, RRID:SCR_018171) [68]. Next we converted the delta file to a chain file using mugsy v1.2.3 delta2maf and maf-convert (Mugsy, RRID:SCR_001414) [69]. We used CrossMap v0.6.1 (CrossMap, RRID:SCR_001173) [59] to identify SNP locations on the query Mgal_WU_HG_1.0 assembly. We further performed a blastn v2.11.0+ search (BLASTN, RRID:SCR_001598) [70] to identify the locations of SNPs that could not be mapped from the previous build using the SNPs probe sequences.

Annotation and repeats

Tandem repeats were identified using the TRF tool [71] and telomeric and TM repeats were identified using the tidk package [72]. The genome was annotated with the ENSEMBL annotation pipeline and is available as part of the Ensembl Rapid Release (Ensembl, RRID:SCR_002344) [25]. The transcriptome and proteome evidence used in the annotation are listed in **Supplementary File 7**. We used a custom python script to query the Ensembl rapid release homologue gene page to identify Turkey_5.1 and GRCg6a homologues of all the Mgal_WU_HG_1.0 genes. The BuildDatabase tool from RepeatModeler v1.0.11 (RepeatModeler, RRID:SCR_015027) [24] was used to build a de novo repeat library from our assembly using the Recon and RepeatScout tools. RepeatMasker v4.0.7 (RepeatMasker, RRID:SCR_012954) [73] was used to identify repeats together with the custom build repeat library from RepeatModeler.

Orthologues

The proteomes of five bird species were used to infer orthogroups (option -og) using OrthoFinder v2.5.4 (OrthoFinder, RRID:SCR_017118) [74]. The proteomes of the following assemblies were downloaded from Ensembl release 106: turkey - Turkey_5.1; chicken - GRCg6a; Japanese quail - Coturnix_japonica_2.0; helmeted guineafowl - NumMel1.0; zebra finch - bTaeGut1_v1.p. The proteomes for Mgal_WU_HG_1.0 (turkey) and GRCg7b (chicken) were downloaded from the Ensembl rapid release (March 2022). For each orthogroup, the protein isoform with the best alignment based on species similarity, score and expect value was chosen. Turkey-specific orthogroups were analysed by running BLASTp v2.11.0+ (BLASTP, RRID:SCR_001010) [70] against the NR database to identify homologous genes from a wider range of species.

Gene family contractions and expansions of protein-coding gene families

Expansions and contractions of protein-coding gene families were assessed by CAFE5 [28]. The phylogenetic tree was obtained using the BirdTree database [75].

Distinct genomic landscapes of turkey micro- and macrochromosomes

To better understand the differences between macro (>40 Mbp), intermediate (>40 Mbp, <20 Mbp), and micro (<20 Mbp) chromosomes, we investigated repeat content, gene structure and gene expression. A welch t-test was used to test for difference of repeat content and families between macro- intermediate and microchromosomes.

Repeats

A custom repeat library created with RepeatModeler and custom R scripts were used to investigate the differences in repeat content between macro, intermediate and microchromosomes. Each chromosome was split into bins (each bin corresponding to 2% of the chromosome length), allowing us to compare the chromosomes by relative length. We calculated the average repeat content in each bin. An ideogram of the density of each repeat feature was created for macro, intermediate and

microchromosomes with the R v4.0.2 (R Project for Statistical Computing, RRID:SCR_001905) [76] package RIdeogram v0.2.2 [77]. RIdeogram calculates feature density in sliding windows (100 Kbp for macro and intermediate chromosomes, 50 Kbp for microchromosomes).

Tissue specificity

Expression data for 16 turkey tissues (jejunum, proventriculus, thigh, testis, ileum, pancreas, spleen, breast, brain, heart, thymus, liver, gizzard, duodenum, caecal tonsil, bursa) from a male individual at three developmental stages (14, 21, 28 days post hatch) was downloaded from Bioproject PRJNA259229. Not all tissues were available at all stages: testis was not available at day 21 and caecal tonsil at day 28. HISAT2 v2.2.1 (HISAT2, RRID:SCR_015530) [78] was used to index the assembly (hisat2-build), and align the RNA-seq reads to the assembly. Stringtie v2.1.7 (StringTie, RRID:SCR_016323) [79] was used to assemble transcripts using the aligned reads and Ensembl gene annotation with options -A and -B. A non-redundant set of transcripts was generated with Stringtie's merge option (--merge), which creates a unified set of transcripts from several samples. Stringtie was run once more, now using this new set of transcripts as the reference annotation file. The resulting table containing the gene abundance of all genes was used in our analysis. We analysed the results through custom R (v4.0.2) scripts. We started by filtering the gene abundance table to keep only the genes that are expressed (FPKM >1). Then we classified genes into housekeeping (expressed in at least 13 tissues), less specific (expressed in at least 5 and in fewer than 13 tissues), specific (expressed in 2 to 5 tissues), and more specific genes (expressed in one or two tissues). The relative abundance of housekeeping/specific genes was calculated by counting the number of genes in these categories in macro, intermediate and microchromosomes and dividing that by the total amount of genes in each chromosome type.

Gene structure

We used RIdeogram v0.2.2 [77] and R (v 4.0.2) to compare the gene density between the chromosome classes. RIdeogram calculates gene density in sliding windows, 100 Kbp for macro and intermediate chromosomes, 50 Kbp for microchromosomes. Gene density per megabase was calculated by dividing the number of annotated genes on a chromosome by its length. A welch t-test was used to test for difference of gene densities between macro- intermediate and microchromosomes.

Synteny

The MCscan python pipeline from the JCVI utility libraries v1.1.11 (MCScan, RRID:SCR_017650) [80] was used study chromosomal rearrangements between several bird species: Turkey (*Meleagris gallopavo*), chicken (*Gallus gallus*), Japanese quail (*Coturnix japonica*), helmeted guineafowl (*Numida meleagris*), great tit (*Parus major*), zebra finch (*Taeniopygia guttata*), and emu (*Dromaius novaehollandiae*).

The genome (fasta coding DNA sequence, CDS) and annotation files for these species were obtained from Ensembl release 106. The files for Mgal_WU_HG_1.0 and GRCg7b were obtained from the Ensembl rapid release (April 2022). The annotation file for the emu assembly ZJU1.0 was shared with us from [81]. This annotation file, in combination with the FASTA file obtained from NCBI was used to create the CDS fasta file necessary for the pipeline.

We started by trimming the accession IDs in the FASTA file and converting the GFF3 annotation file to BED format. The jcvi.compara.catalog ortholog and jcvi.compara.synteny screen (with parameters --simple) were used to create the necessary input files for plotting. The synteny plots were created with jcvi.graphics.karyotype using parameter --basepair. To validate the chromosome Z inversion, first, we manually checked the inversion breakpoints (reads spanning) using JBrowse 1.16.9.

Data Availability

The genome assemblies and sequencing data have been deposited in ENA under Bioproject accession PRJEB42643. The turkey genome and annotations are available through ENSEMBL Rapid Release [82] All supporting data and materials are available in the *GigaScience* GigaDB database [83].

Supplementary Files

Supplementary File 1: Table S1: Genome assembly and annotation overview.

Supplementary File 1: Table S2: QV values indicating assembly quality and completeness.

Supplementary File 1: Table S3: Protein homology between Mgal_WU_HG_1.0, Turkey_5.1 and chicken (GRCg6a).

Supplementary File 1: Table S4: Blast results of proteins in Mgal_WU_HG_1.0 specific orthogroups.

Supplementary File 1: Table S5: Mapping of 65K markers on Mgal5.1 and Mgal_WU_HG_1.0.

Supplementary File 1: Table S6: Mapping rate of RNA-seq datasets from 16 tissues to Mgal_WU_HG_1.0. Tissues (jejunum, proventriculus, thigh, testis, ileum, pancreas, spleen, breast, brain, heart, thymus, liver, gizzard, duodenum, caecal tonsil, bursa) are from a male individual at three developmental stages (14, 21, 28 days post hatch).

Supplementary File 1: Table S6: Mummer alignment between Turkey5.1 and Mgal_WUR_HG_1.0 of the first and the second breakpoint of the 19.4 Mbp inversion on the Z-chromosome.

Supplementary File 1: Figure S1: Hi-C contact map of the Mgal_WU_HG_1.0 assembly.

Supplementary File 1: Figure S2: Overview of tandem repeats identified in the turkey genome.

Supplementary File 1: Figure S3: Parent 1 vs. parent 2 alignment.

Supplementary File 1: Figure S4: Distribution of copy gains and copy losses in parent 2 compared to parent 1 haplotype.

Supplementary File 1: Figure S5: Inversion comprising the start of the BLB2 gene in parent2 compared to the parent1 haplotype.

Supplementary File 1: Figure S6: Duplication affecting the tail of the MAN2B2 gene in parent2 compared to the parent1 haplotype.

Supplementary File 1: Figure S7: Duplication affecting GEMIN8 gene in parent2 compared to the parent1 haplotype.

Supplementary File 1: Figure S8: Duplication affecting RIMKLB gene with higher copy number in parent 2 compared to parent1 haplotype.

Supplementary File 1: Figure S9: Average DNA repeat content along the chromosomes for macro, intermediate and microcromosomes.

Supplementary File 1: Figure S10: Average LINE repeat content along the chromosomes for macro, intermediate and microcromosomes.

Supplementary File 1: Figure S11: Average low complexity repeat content along the chromosomes for macro, intermediate and microcromosomes.

Supplementary File 1: Figure S12: Average LTR repeat content along the chromosomes for macro, intermediate and microcromosomes.

Supplementary File 1: Figure S13: Average simple repeat content along the chromosomes for macro, intermediate and microcromosomes.

Supplementary File 1: Figure S14: Average SINE repeat content along the chromosomes for macro, intermediate and microcromosomes.

Supplementary File 1: Figure S15: Average snRNA repeat content along the chromosomes for macro, intermediate and microcromosomes.

Supplementary File 1: Figure S16: Average unknown repeat content along the chromosomes for macro, intermediate and microcromosomes.

Supplementary File 1: Figure S17: Chromosome Z alignment showing inversion with GRCg7b (A) and Turkey5.1 (B).

Supplementary File 1: Figure S18: Alignment of corrected pacbio reads at the approximate breakpoints of the ~19.4 Mbp inversion on the Z-chromosome.

Supplementary File 1: Figure S19: HiC contact map of the Z chromosome.

Supplementary File 1: Figure S20: Schematic view of Gal7b chromosome Z and representation of several biotypes of genes and genomic features (Ensembl, rapid release 15th June 2022, accessed on 27th June 2022).

Supplementary File 2: Repeat annotation.

Supplementary File 3: Gene family expansions and contractions.

Supplementary File 4: Syri output showing structural variation between the two parent haplotypes.

Supplementary File 5: Structural variation between parent haplotypes.

Supplementary File 6: Stop-gained variants identified in either or one of the two parent haplotypes.

Supplementary File 7: Transcriptome and proteome evidence used for ENSEMBL Annotation.

Competing Interests

J. Mohr and B.J. Wood were employed by Hybrid Turkeys and M.C.A.M Bink was employed by Hendrix Genetics Research. Both institutes are part of one of the funders (Hendrix Genetics). All authors declare that the results are presented in full and as such present no conflict of interest. The other Breed4Food partners Cobb Europe, CRV, Topigs Norsvin, declare to have no competing interests for this study.

Funding

This research was funded by the STW-Breed4Food Partnership, project number 14283: From sequence to phenotype: detecting deleterious variation by prediction of functionality. This study was financially supported by NWO-TTW and the Breed4Food partners Cobb Europe, CRV, Hendrix Genetics and Topigs Norsvin.

Ethical Statement

Ethical review and approval were not required for sample collection since the data used in this study has been obtained as part of routine data collection from Hybrid Turkeys' breeding programmes, and not specifically for the purpose of this project. Therefore, approval of an ethics committee was not mandatory.

Authors' Contributions

MAMG designed, coordinated, and managed the project; JM and BJW were involved in data collection and preparation; RPMAC was involved in data collection and wet lab work; HJM provided valuable input regarding the analyses and manuscript; CPB and MFLD performed the analysis and drafted the manuscript. All authors read and approved the final manuscript.

Acknowledgements

We are grateful to Luohao Xu (Key Laboratory of Freshwater Fish Reproduction and Development, Southwest University, Chongqing) for sharing the annotation file for ZJU1.0. We thank the ENSEMBL support team for providing details on the annotation.

List of Abbreviations

BED: Browser Extensible Data; BLAST: Basic Local Alignment Search Tool; BLASTN: BLAST search of nucleotide database(s); BLASTP: BLAST search protein databases using a protein query; bp: base pairs; BUSCO: Benchmarking Universal Single-Copy Orthologs; BWA: Burrows-Wheeler Aligner; CDS: coding sequence; F1: Filial 1, first offspring from a cross; FPKM: Fragments Per Kilobase Million; GC: guanine-cytosine; GFF3: general feature format, version 3; GWAS: genome wide association studies; Hi-C: chromosome conformation capture; INDEL: insertion or deletion; Kbp: kilo base pairs; LINE: Long interspersed nuclear elements; lnRNA: long non-coding RNA; LoF: loss of function; Mbp: megabase pairs; NCBI: National Center for Biotechnology Information; PacBio: Pacific Biosciences; SMN: survival motor neuron; SMRT: single molecule real time; SNP: single nucleotide polymorphism; VCF: variant call format.

References

1. (AVEC), A.o.P.P.a.P.T.i.t.E.C., *2021 Annual report.* 2021.
2. Chen, D., et al., *Divergence time estimation of Galliformes based on the best gene shopping scheme of ultraconserved elements.* Bmc Ecology and Evolution, 2021. **21**(1). doi: 10.1186/s12862-021-01935-1
3. Griffin, D., et al., *The evolution of the avian genome as revealed by comparative molecular cytogenetics.* Chromosome Research, 2007. **15**: p. 29-29.
4. Griffin, D.K., et al., *Whole genome comparative studies between chicken and turkey and their implications for avian genome evolution.* Bmc Genomics, 2008. **9**. doi: 10.1186/1471-2164-9-168
5. Zhang, Y., et al., *A comparative physical map reveals the pattern of chromosomal evolution between the turkey (Meleagris gallopavo) and chicken (Gallus gallus) genomes.* Bmc Genomics, 2011. **12**. doi: 10.1186/1471-2164-12-447
6. Dalloul, R.A., et al., *Multi-Platform Next-Generation Sequencing of the Domestic Turkey (Meleagris gallopavo): Genome Assembly and Analysis.* Plos Biology, 2010. **8**(9):e1000475..
7. Peona, V., et al., *Identifying the causes and consequences of assembly gaps using a multiplatform genome assembly of a bird-of-paradise.* Molecular Ecology Resources, 2021. **21**(1): p. 263-286.
8. Huang, Z., et al., *Evolutionary analysis of a complete chicken genome.* Proc Natl Acad Sci U S A, 2023. **120**(8): p. e2216641120.
9. Meuwissen, T., B. Hayes, and M. Goddard, *Genomic selection: A paradigm shift in animal breeding.* Animal Frontiers, 2016. **6**(1): p. 6-14.
10. Rexroad, C., et al., *Genome to Phenome: Improving Animal Health, Production, and Well-Being - A New USDA Blueprint for Animal Genome Research 2018-2027.* Frontiers in Genetics, 2019. **10**:327.
11. Morris, K.M., et al., *The quail genome: insights into social behaviour, seasonal biology and infectious disease response.* Bmc Biology, 2020. **18**(1):1-8.

12. Oh, K.P., et al., *Conservation Genomics in the Sagebrush Sea: Population Divergence, Demographic History, and Local Adaptation in Sage-Grouse (Centrocercus spp.).* Genome Biology and Evolution, 2019. **11**(7): p. 2023-2034.
13. Shen, Q.K., et al., *Genomic Analyses of Unveil Helmeted Guinea Fowl (Numida meleagris) Domestication in West Africa.* Genome Biology and Evolution, 2021. **13**(6) :evab090.
14. Koren, S., et al., *De novo assembly of haplotype-resolved genomes with trio binning.* Nature Biotechnology, 2018. **36**(12): p. 1174 -82 .
15. Ruan, J. and H. Li, *Fast and accurate long-read assembly with wtdbg2.* Nature Methods, 2020. **17**(2): p. 155-8.
16. Putnam, N.H., et al., *Chromosome-scale shotgun assembly using an in vitro method for long-range linkage.* Genome Research, 2016. **26**(3): p. 342-350.
17. Ghurye, J., et al., *Scaffolding of long read assemblies using long range contact information.* Bmc Genomics, 2017. **18** (1):1-1.
18. Pryszcz, L.P. and T. Gabaldon, *Redundans: an assembly pipeline for highly heterozygous genomes.* Nucleic Acids Research, 2016. **44**(12): e113-e113.
19. Matzke, A.J., et al., *Characterization of a new repetitive sequence that is enriched on microchromosomes of turkey.* Chromosoma, 1992. **102**(1): p. 9-14.
20. Qin, M., et al., *LRScaf: improving draft genomes using long noisy reads.* Bmc Genomics, 2019. **20**(1): 1-12.
21. Alonge, M., et al., *RaGOO: fast and accurate reference-guided scaffolding of draft genomes.* Genome Biology, 2019. **20**(1): 1-7.
22. Rhie, A., et al., *Merqury: reference-free quality, completeness, and phasing assessment for genome assemblies.* Genome Biology, 2020. **21**(1): 1-27.
23. Simao, F.A., et al., *BUSCO: assessing genome assembly and annotation completeness with single-copy orthologs.* Bioinformatics, 2015. **31**(19): p. 3210-3212.
24. Flynn, J.M., et al., *RepeatModeler2 for automated genomic discovery of transposable element families.* Proceedings of the National Academy of Sciences of the United States of America, 2020. **117**(17): p. 9451-9457.
25. Cunningham, F., et al., *Ensembl 2022.* Nucleic Acids Research, 2022. **50**(D1): p. D988-D995.
26. Emms, D.M. and S. Kelly, *OrthoFinder: solving fundamental biases in whole genome comparisons dramatically improves orthogroup inference accuracy.* Genome Biology, 2015. **16** (1), pp.1-14.
27. Warren, W.C., et al., *The genome of a songbird.* Nature, 2010. **464**(7289): p. 757-762.
28. Mendes, F.K., et al., *CAFE 5 models variation in evolutionary rates among gene families.* Bioinformatics, 2020. **36**(22-23): p. 5516-5518.
29. Fouchecourt, S., et al., *Expanding duplication of the testis PHD Finger Protein 7 (PHF7) gene in the chicken genome.* Genomics, 2022. **114**(4): 110411.
30. Kaufman, J., *Innate immune genes of the chicken MHC and related regions.* Immunogenetics, 2022. **74**(1): p. 167-177.
31. Campbell, E.M., et al., *Genetic variation in the mannosidase 2B2 gene and its association with ovulation rate in pigs.* Animal Genetics, 2008. **39**(5): p. 515-519.
32. Georges, M., C. Charlier, and B. Hayes, *Harnessing genomic information for livestock improvement.* Nature Reviews Genetics, 2019. **20**(3): p. 135-156.
33. Basaki, M., et al., *Sequence and expression analysis of cardiac ryanodine receptor 2 in broilers that died from sudden death syndrome.* Avian Pathology, 2019. **48**(5): p. 444-453.
34. Bult, C.J., et al., *Mouse Genome Database (MGD) 2019.* Nucleic Acids Research, 2019. **47**(D1): p. D801-D806.
35. Waters, P.D., et al., *Microchromosomes are building blocks of bird, reptile, and mammal chromosomes.* Proc Natl Acad Sci U S A, 2021. **118**(45): e2112494118.
36. O'Connor, R.E., et al., *Patterns of microchromosome organization remain highly conserved throughout avian evolution.* Chromosoma, 2019. **128**(1): p. 21-29.

37. Shibusawa, M., et al., *Karyotypic evolution in the Galliformes: An examination of the process of karyotypic evolution by comparison of the molecular cytogenetic findings with the molecular phylogeny.* Cytogenetic and Genome Research, 2004. **106**(1): p. 111-119.
38. Xu, L.H., et al., *Dynamic evolutionary history and gene content of sex chromosomes across diverse songbirds.* Nature Ecology & Evolution, 2019. **3**(5): p. 834-844.
39. Bellott, D.W., et al., *Avian W and mammalian Y chromosomes convergently retained dosage-sensitive regulators.* Nature Genetics, 2017. **49**(3): p. 387-394.
40. Mascaro, M., I. Lages, and G. Meroni, *Microtubular TRIM36 E3 Ubiquitin Ligase in Embryonic Development and Spermatogenesis.* Cells, 2022. **11**(2):246..
41. Zhou, B., et al., *Case Report: A Novel De Novo Missense Mutation of the GRIA2 Gene in a Chinese Case of Neurodevelopmental Disorder With Language Impairment.* Front Genet, 2021. **12**: p. 794766.
42. Tian, Q., et al., *Compound heterozygous variants in MAN2B2 identified in a Chinese child with congenital disorders of glycosylation.* Eur J Hum Genet, 2022. 31:1-3.
43. Cauchi, R.J., *SMN and Gemins: 'we are family' ... or are we?: insights into the partnership between Gemins and the spinal muscular atrophy disease protein SMN.* Bioessays, 2010. **32**(12): p. 1077-89.
44. Li, Y.I., et al., *Rapid Evolution of Beta-Keratin Genes Contribute to Phenotypic Differences That Distinguish Turtles and Birds from Other Reptiles.* Genome Biology and Evolution, 2013. **5**(5): p. 923-933.
45. Wang, X.R., et al., *Evidence for parallel evolution of a gene involved in the regulation of spermatogenesis.* Proceedings of the Royal Society B-Biological Sciences, 2017. **284**(1855):20170324.
46. Fouchecourt, S., et al., *An evolutionary approach to recover genes predominantly expressed in the testes of the zebrafish, chicken and mouse.* Bmc Evolutionary Biology, 2019. **19,** pp.1-15.
47. Braasch, I., et al., *The spotted gar genome illuminates vertebrate evolution and facilitates human-teleost comparisons (vol 48, pg 427, 2016).* Nature Genetics, 2016. **48**(6): p. 700-700.
48. Kapusta, A. and A. Suh, *Evolution of bird genomes-a transposon's-eye view.* Annals of the New York Academy of Sciences, 2017. **1389**(1): p. 164-185.
49. Zhang, G.J., et al., *Comparative genomics reveals insights into avian genome evolution and adaptation.* Science, 2014. **346**(6215): p. 1311-1320.
50. Aslam, M.L., et al., *A SNP based linkage map of the turkey genome reveals multiple intrachromosomal rearrangements between the Turkey and Chicken genomes.* Bmc Genomics, 2010. **11** (1) : 1-11.
51. Walker, B.J., et al., *Pilon: An Integrated Tool for Comprehensive Microbial Variant Detection and Genome Assembly Improvement.* Plos One, 2014. **9**(11): e112963.
52. Genomics, A. *Arima Genomics Pipeline*. 2019; Available from: https://github.com/ArimaGenomics/mapping_pipeline.
53. Li, H. and R. Durbin, *Fast and accurate short read alignment with Burrows-Wheeler transform.* Bioinformatics, 2009. **25**(14): p. 1754-1760.
54. Broad Institute. *Picard: A set of Java command line tools for manipulating high-throughput sequencing data (HTS) data and formats*. 2022; Available from: http://broadinstitute.github.io/picard/.
55. Durand, N.C., et al., *Juicer Provides a One-Click System for Analyzing Loop-Resolution Hi-C Experiments.* Cell Systems, 2016. **3**(1): p. 95-98.
56. Durand, N.C., et al., *Juicebox Provides a Visualization System for Hi-C Contact Maps with Unlimited Zoom.* Cell Systems, 2016. **3**(1): p. 99-101.
57. Buels, R., et al., *JBrowse: a dynamic web platform for genome visualization and analysis.* Genome Biology, 2016. **17**(1): 1-12.

58. Li, H., *Minimap2: pairwise alignment for nucleotide sequences.* Bioinformatics, 2018. **34**(18): p. 3094-3100.
59. Cabanettes, F. and C. Klopp, *D-GENIES: dot plot large genomes in an interactive, efficient and simple way.* Peerj, 2018. **6:e4958. doi: 10.7717/peerj.4958.**
60. Goel, M., et al., *SyRI: finding genomic rearrangements and local sequence differences from whole-genome assemblies.* Genome Biology, 2019. **20**(1): 1-13.
61. Goel, M. and K. Schneeberger, *plotsr: visualizing structural similarities and rearrangements between multiple genomes.* Bioinformatics, 2022. 38(10):2922-6.
62. Faust, G.G. and I.M. Hall, *SAMBLASTER: fast duplicate marking and structural variant read extraction.* Bioinformatics, 2014. **30**(17): p. 2503-2505.
63. Li, H., et al., *The Sequence Alignment/Map format and SAMtools.* Bioinformatics, 2009. **25**(16): p. 2078-2079.
64. E. Garrison and G. Marth, *Haplotype-based variant detection from short-read sequencing.* 2012. arXiv:1207.3907
65. Bonfield, J.K., et al., *HTSlib: C library for reading/writing high-throughput sequencing data.* Gigascience, 2021. **10**(2):giab007. doi: 10.1093/gigascience/giab007.
66. Danecek, P., et al., *Twelve years of SAMtools and BCFtools.* Gigascience, 2021. **10**(2):giab008. doi: 10.1093/gigascience/giab008.
67. Okonechnikov, K., A. Conesa, and F. Garcia-Alcalde, *Qualimap 2: advanced multi-sample quality control for high-throughput sequencing data.* Bioinformatics, 2016. **32**(2): p. 292-294.
68. Marcais, G., et al., *MUMmer4: A fast and versatile genome alignment system.* Plos Computational Biology, 2018. **14**(1):e1005944..
69. Angiuoli, S.V. and S.L. Salzberg, *Mugsy: fast multiple alignment of closely related whole genomes.* Bioinformatics, 2011. **27**(3): p. 334-342.
70. Camacho, C., et al., *BLAST plus : architecture and applications.* Bmc Bioinformatics, 2009. **10**.
71. Benson, G., *Tandem repeats finder: a program to analyze DNA sequences.* Nucleic Acids Res, 1999. **27**(2): p. 573-80.
72. Tolit. *A Telomere Identification toolKit (tidk)*. 2018; Available from: https://github.com/tolkit/telomeric-identifier.
73. A. Smith, R. Hubley, and P. Green, *RepeatMasker Open-4.0.* 2013-2015.
74. Emms, D.M. and S. Kelly, *OrthoFinder: phylogenetic orthology inference for comparative genomics.* Genome Biology, 2019. **20**(1):238. doi: 10.1186/s13059-019-1832-y.
75. Jetz, W., et al., *The global diversity of birds in space and time.* Nature, 2012. **491**(7424): p. 444-448.
76. Team, R.C. *R: A Language and Environment for Statistical Computing*. 2020; Available from: https://www.r-project.org/.
77. Hao, Z.D., et al., *RIdeogram: drawing SVG graphics to visualize and map genome-wide data on the idiograms.* Peerj Computer Science, 2020. 20;6:e251.
78. Kim, D., B. Landmead, and S.L. Salzberg, *HISAT: a fast spliced aligner with low memory requirements.* Nature Methods, 2015. **12**(4): p. 357-360.
79. Pertea, M., et al., *StringTie enables improved reconstruction of a transcriptome from RNA-seq reads.* Nature Biotechnology, 2015. **33**(3): p. 290-5. doi: 10.1038/nbt.3122.
80. Wang, Y.P., et al., *MCScanX: a toolkit for detection and evolutionary analysis of gene synteny and collinearity.* Nucleic Acids Research, 2012. **40**(7), pp.e49-e49.
81. Liu, J., et al., *A new emu genome illuminates the evolution of genome configuration and nuclear architecture of avian chromosomes.* Genome Research, 2021. **31**(3): p. 497-511.

82. Ensembl Rapid Release. *Genome annotation for MGAL_WU_HG_1.0*. 2022; Available from: https://rapid.ensembl.org/Meleagris_gallopavo_GCA_905368555.1/

83. Barros CP; Derks M; Mohr J; Wood B; M A Crooijmans RP; Megens H; A M Bink MC; M Groenen MA. Supporting data for "A new haplotype-resolved turkey genome to enable turkey

genetics and genomics research" GigaScience Database 2023.
http://dx.doi.org/10.5524/102394

Figure 1

Click here to access/download;Figure;Figure1.tif

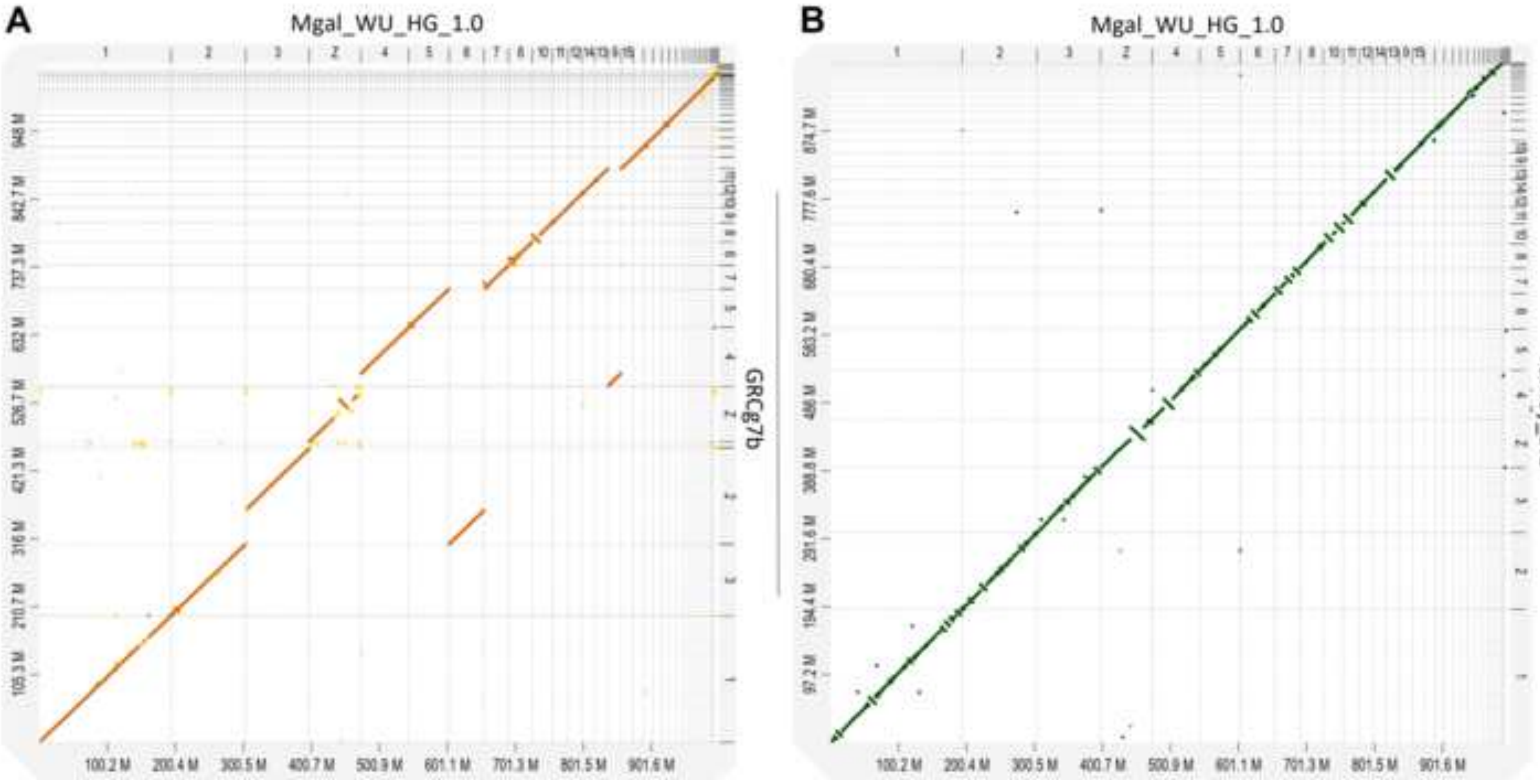

Figure 2

Click here to access/download;Figure;Figure2.tif

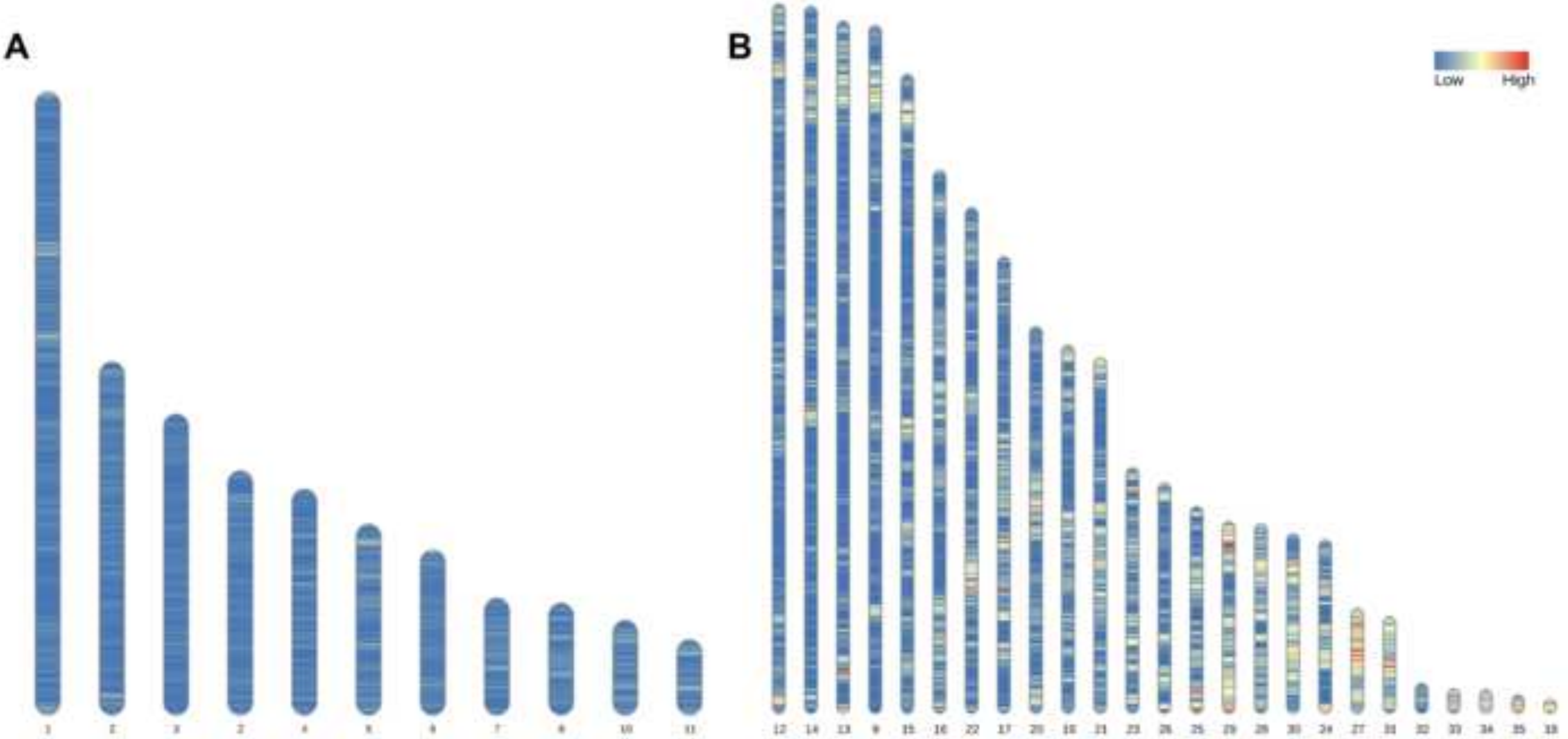

Figure 3

Click here to access/download;Figure;Figure3.tif

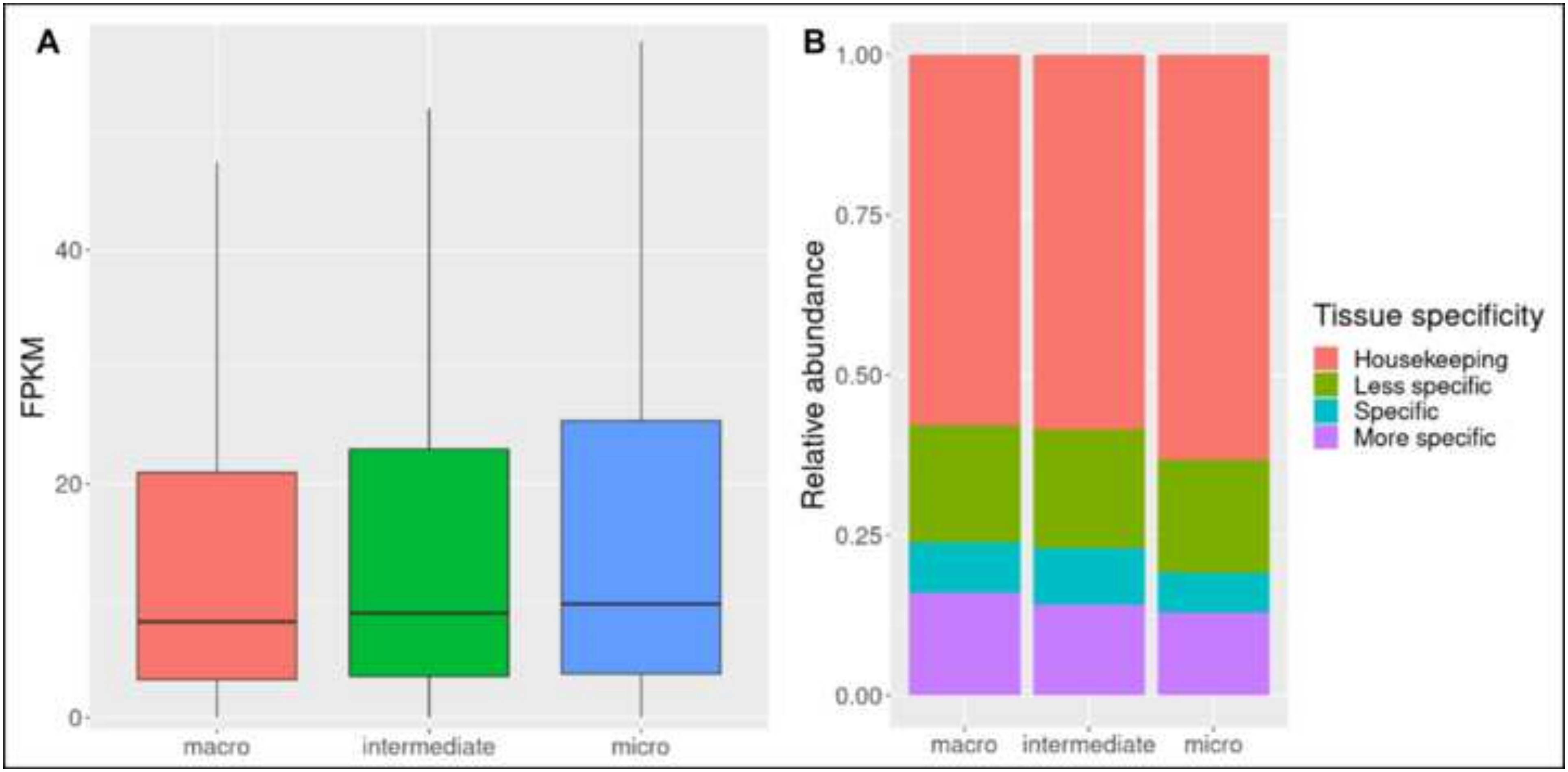

Figure 4

Click here to access/download;Figure;Figure4.pdf

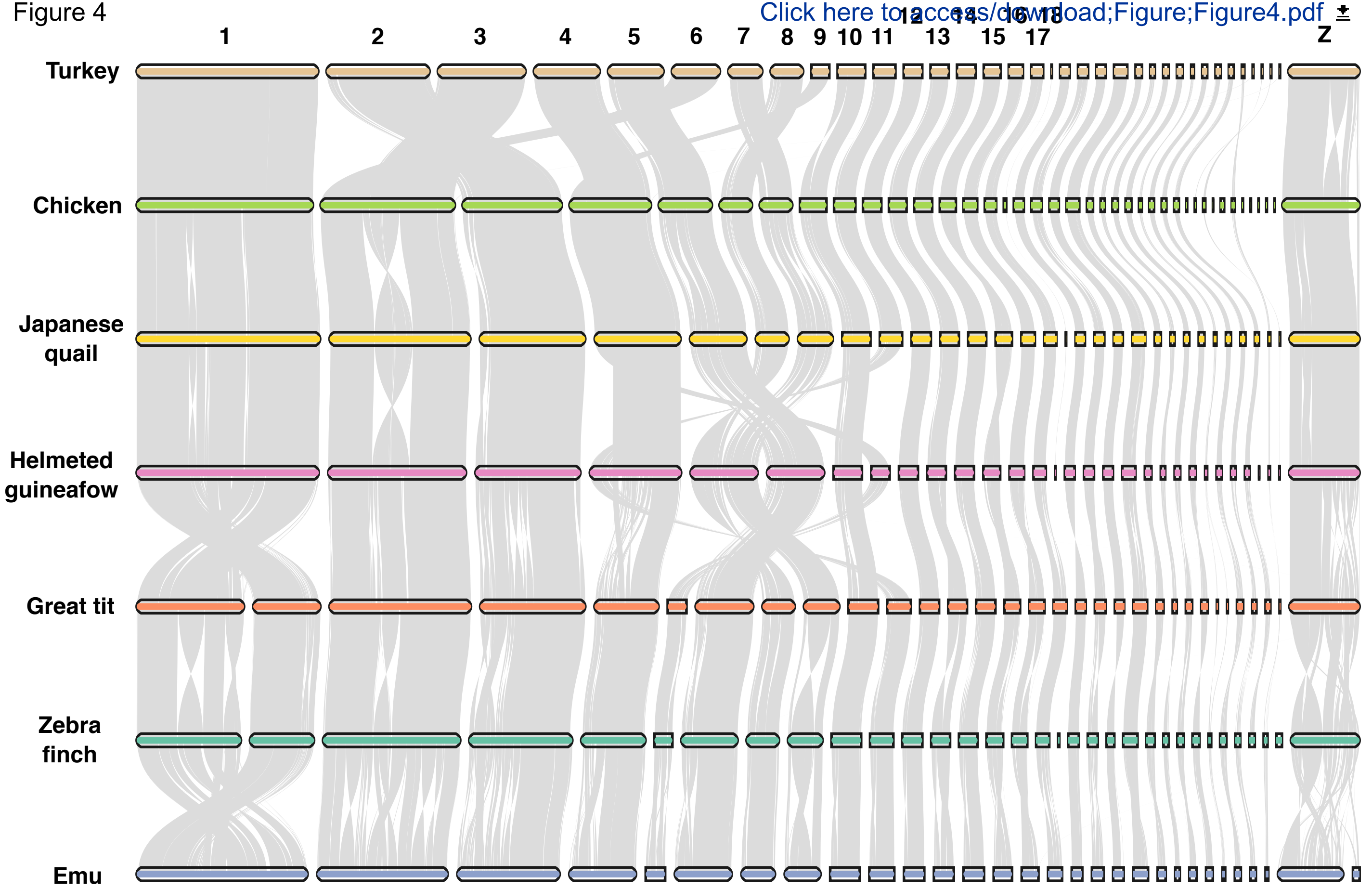

Click here to access/download;Figure;Figure5.pdf

Figure 5

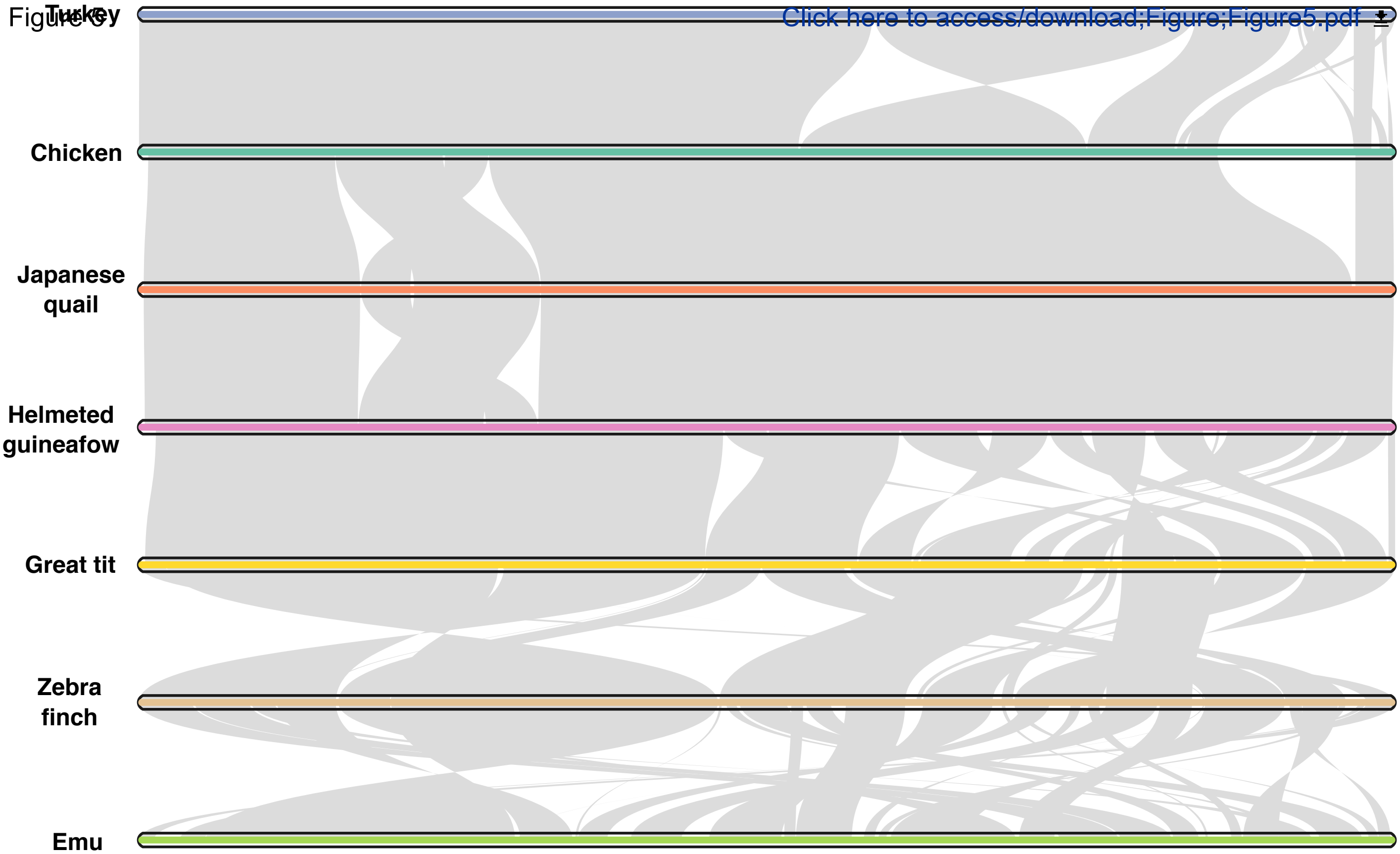

Supplementary File 1

Click here to access/download
Supplementary Material
SupplementaryFile1.docx

Supplementary File 2

Click here to access/download
Supplementary Material
SupplementaryFile2.xlsx

Supplementary File 3

Click here to access/download

Supplementary Material

SupplementaryFile3.xlsx

Supplementary File 4

Click here to access/download
Supplementary Material
SupplementaryFile4.pdf

Supplementary File 5

Click here to access/download
Supplementary Material
SupplementaryFile5.xlsx

Supplementary File 6

Click here to access/download

Supplementary Material

SupplementaryFile6.xlsx

Supplementary File 7

Click here to access/download
Supplementary Material
SupplementaryFile7.xlsx
