# Supplementary material for: A new haplotype-resolved turkey genome to enable turkey genetics and genomics research
Source: Gigascience. 2023 Jul 21;12:giad051. doi: 10.1093/gigascience/giad051 (PMC10360393; doi:10.1093/gigascience/giad051)
Supplement: giad051_Supplemental_Files [file giad051_supplemental_files.zip › SupplementaryFile1.docx]

**Table S1**: Genome assembly and annotation overview.

| Chr | Genbank | Size | #Spanned gaps | GC% | #Protein-coding genes | Gene-density per Mb | #Non-coding genes | GRCg7b homolog chr  3  2  4  5  2  7  6  4  8  9  10  11  12  13  14  15  16  17  18  19  20  21  22  23  24  25  26  27  28  34  Undetermined  31  30  Undetermined  Z |
| --- | --- | --- | --- | --- | --- | --- | --- | --- |
| 1 | HG999680.1 | 194,070,912 | 27 | 39.5 | 2172 | 11.19 | 1301 | 1 |
| 2 | HG999681.1 | 109,989,719 | 2 | 39 | 1208 | 10.98 | 794 | 3 |
| 3 | HG999682.1 | 93,699,536 | 2 | 38.5 | 759 | 8.1 | 550 | 2 |
| 4 | HG999684.1 | 70,339,173 | 1 | 38.5 | 760 | 10.8 | 458 | 4 |
| 5 | HG999685.1 | 59,398,638 | 3 | 40 | 970 | 16.33 | 462 | 5 |
| 6 | HG999686.1 | 51,422,372 | 0 | 39.5 | 610 | 11.86 | 354 | 2 |
| 7 | HG999687.1 | 36,365,977 | 3 | 40.5 | 506 | 13.91 | 266 | 7 |
| 8 | HG999688.1 | 34,787,218 | 1 | 41 | 527 | 15.15 | 293 | 6 |
| 9 | HG999694.1 | 19,224,719 | 0 | 42 | 346 | 18 | 191 | 4 |
| 10 | HG999689.1 | 29,439,779 | 1 | 41 | 516 | 17.53 | 229 | 8 |
| 11 | HG999690.1 | 23,442,721 | 0 | 42 | 454 | 19.37 | 210 | 9 |
| 12 | HG999691.1 | 19,827,528 | 1 | 42.5 | 410 | 20.68 | 209 | 10 |
| 13 | HG999693.1 | 19,350,529 | 0 | 41.5 | 373 | 19.28 | 183 | 11 |
| 14 | HG999692.1 | 19,767,664 | 1 | 42.5 | 348 | 17.6 | 167 | 12 |
| 15 | HG999695.1 | 17,861,904 | 0 | 44 | 389 | 21.78 | 160 | 13 |
| 16 | HG999696.1 | 15,176,134 | 1 | 44.5 | 422 | 27.81 | 114 | 14 |
| 17 | HG999698.1 | 12,762,598 | 0 | 44.5 | 376 | 29.46 | 122 | 15 |
| 18 | HG999715.1 | 397,821 | 1 | 53 | 47 | 118.14 | 7 | 16 |
| 19 | HG999700.1 | 10,289,109 | 0 | 47 | 295 | 28.67 | 86 | 17 |
| 20 | HG999699.1 | 10,809,360 | 0 | 46 | 315 | 29.14 | 105 | 18 |
| 21 | HG999701.1 | 9,941,450 | 1 | 46 | 347 | 34.9 | 106 | 19 |
| 22 | HG999697.1 | 14,137,791 | 1 | 45.5 | 362 | 25.61 | 131 | 20 |
| 23 | HG999702.1 | 6,858,058 | 0 | 47 | 260 | 37.91 | 61 | 21 |
| 24 | HG999708.1 | 4,843,517 | 0 | 48.5 | 192 | 39.64 | 64 | 22 |
| 25 | HG999704.1 | 5,777,478 | 0 | 50 | 245 | 42.41 | 64 | 23 |
| 26 | HG999703.1 | 6,438,191 | 0 | 48 | 179 | 27.8 | 78 | 24 |
| 27 | HG999709.1 | 2,935,620 | 7 | 56 | 294 | 100.15 | 38 | 25 |
| 28 | HG999706.1 | 5,298,159 | 0 | 51 | 270 | 50.96 | 60 | 26 |
| 29 | HG999705.1 | 5,353,804 | 3 | 51.5 | 368 | 68.74 | 64 | 27 |
| 30 | HG999707.1 | 5,017,032 | 1 | 52 | 303 | 60.39 | 59 | 28 |
| 31 | HG999710.1 | 2,691,915 | 3 | 57.5 | 203 | 75.41 | 36 | 34 |
| 32 | HG999711.1 | 834,222 | 2 | 56.5 | 24 | 28.77 | 15 | Undetermined |
| 33 | HG999712.1 | 677,051 | 0 | 50 | 54 | 79.76 | 19 | 31 |
| 34 | HG999713.1 | 651,658 | 2 | 56.5 | 54 | 82.87 | 11 | 30 |
| 35 | HG999714.1 | 509,708 | 0 | 63 | 45 | 88.29 | 10 | Undetermined |
| Z | HG999683.1 | 76,077,877 | 7 | 39 | 803 | 10.55 | 523 | Z |

**Table S2:** QV values indicating assembly quality and completeness.

| **Assembly** | **solid k-mers assembly** | **solid k-mers reads** | **%completeness** | **k-mers uniquely in the assembly** | **k-mers in both assembly and the read set** | **QV** | **Error rate** |
| --- | --- | --- | --- | --- | --- | --- | --- |
| parent1 | 947815732 | 997357165 | 95.0327 | 1303996 | 1050586258 | 42.2812 | 5.91E-05 |
| parent2 | 951236015 | 997357165 | 95.3757 | 1184706 | 1085153258 | 42.8387 | 5.20E-05 |
| parent1/parent2 | 984799775 | 997357165 | 98.7409 | 2488702 | 2135739516 | 42.5555 | 5.55E-05 |
| Mgal_WUR_HG_1.0 | 946685368 | 997357165 | 94.9194 | 2859997 | 1001802190 | 38.6604 | 0.00013613 |

**Table S3:** Protein homology between Mgal_WU_HG_1.0, Turkey_5.1 and chicken (GRCg6a).

| **Homologues in:** | **No genes** |
| --- | --- |
| Turkey_5.1 | 13281 |
| GRCg6a | 13915 |
| Turkey_5.1 and GRCg6a | 12372 |
| Total in dataset | 16127 |

**Table S4:** Blast results of proteins in Mgal_WU_HG_1.0 specific orthogroups.

| **Mgal_WU_HG_1.0 specific orthogroups** | **N genes** | **Best hit** | **Species** | **Score** | **expect** |  |
| --- | --- | --- | --- | --- | --- | --- |
| OG0000987 | 11 | E3 ubiquitin-protein ligase Topors-like | Turkey | 46.6 bits (109) | 0.001 |  |
| OG0011308 | 7 | CCNF (cyclin F) | Marbled wood quail | 63.9 bits (154) | 2e-09 |  |
| OG0011309* | 7 | Probable E3 ubiquitin-protein ligase HERC4 isoform X3 | Northern white-cheeked gibbon | 59.3 bits (142) | 2e-09 |  |
| **OG0013797** | **5** | **Protein MANBAL-like** | **Turkey** | **110 bits (275)** | **2e-29** |  |
| OG0013798* | 5 | Wingless-type MMTV integration site family, member 7B | Homo sapiens | 60.5 bits (145) | 1e-8 |  |
| OG0013799* | 5 | hypothetical protein PVAP13_4KG009300 | Switchgrass | 48.5 bits (114) | 5e-04 |  |
| OG0014231* | 4 | hCG1816008 | Homo sapiens | 51.2 bits (121) | 1e-06 |  |
| **OG0015325** | **2** | **POL3 protein / POL2 protein** | **White-crested guan/Pallas's sandgrouse** | **251 bits (642)** | **8e-83** | Catalytic component of DNA polymerase delta (DNA polymerase III) which participates in chromosomal DNA replication |
| OG0015337* | 2 | Hypothetical protein EGK_13122 | Rhesus macaque | 56.6 bits (135) | 2e-08 |  |
| OG0015340 | 2 | Hypothetical protein AXJ14_gp023 | Geobacillus virus E3 | 35.8 bits (81) | 5.6 |  |

***** No significant matches within Aves

**Table S5:** Mapping of 65K markers on Mgal5.1 and Mgal_WU_HG_1.0.

| **Chr** | **# SNPs on Mgal5.1** | **#SNPs lost in Mgal_WU_HG_1.0** | **# SNPs gained in Mgal_WU_HG_1.0** | **# SNPs on Mgal_WU_HG_1.0** |
| --- | --- | --- | --- | --- |
| 1 | 7068 | 44 | 412 | 7436 |
| 2 | 4085 | 44 | 125 | 4166 |
| 3 | 3468 | 48 | 122 | 3542 |
| 4 | 2450 | 41 | 37 | 2446 |
| 5 | 2143 | 13 | 32 | 2162 |
| 6 | 3758 | 16 | 54 | 3796 |
| 7 | 2678 | 20 | 2 | 2660 |
| 8 | 2393 | 11 | 20 | 2402 |
| 9 | 1231 | 21 | 10 | 1220 |
| 10 | 2123 | 17 | 18 | 2124 |
| 11 | 2732 | 57 | 6 | 2681 |
| 12 | 2179 | 6 | 1 | 2174 |
| 13 | 2652 | 11 | 6 | 2647 |
| 14 | 2048 | 8 | 3 | 2043 |
| 15 | 2417 | 15 | 4 | 2406 |
| 16 | 2043 | 14 | 1 | 2030 |
| 17 | 1905 | 55 | 3 | 1853 |
| 18 | 41 | 5 | 3 | 39 |
| 19 | 1400 | 9 | 5 | 1396 |
| 20 | 1416 | 11 | 5 | 1410 |
| 21 | 1447 | 9 | 9 | 1447 |
| 22 | 1629 | 9 | 4 | 1624 |
| 23 | 1013 | 3 | 3 | 1013 |
| 24 | 680 | 5 | 41 | 716 |
| 25 | 798 | 9 | 2 | 791 |
| 26 | 1004 | 5 | 2 | 1001 |
| 27 | 175 | 2 | 120 | 293 |
| 28 | 721 | 4 | 15 | 732 |
| 29 | 692 | 24 | 50 | 718 |
| 30 | 660 | 35 | 14 | 639 |
| 31 | 0 | 0 | 192 | 192 |
| 32 | 0 | 0 | 53 | 53 |
| 33 | 0 | 0 | 82 | 82 |
| 34 | 0 | 0 | 59 | 59 |
| 35 | 0 | 0 | 29 | 29 |
| Z | 3954 | 34 | 594 | 4514 |
| Unplaced | 1796 | 1532 | 682 | 264 |

**Table S6:** Mapping rate of RNA-seq datasets from 16 tissues to Mgal_WU_HG_1.0. Tissues (jejunum, proventriculus, thigh, testis, ileum, pancreas, spleen, breast, brain, heart, thymus, liver, gizzard, duodenum, caecal tonsil, bursa ) are from a male individual at three developmental stages (14, 21, 28 days post hatch).

| Sample ID | Sample information | Total reads | Unmapped | Aligned one time | Aligned multiple | Alignment rate |
| --- | --- | --- | --- | --- | --- | --- |
| SRR1570211 | Gizzard_D14_M | 23328241 | 2234476 | 20808672 | 285093 | 90.42 |
| SRR1570212 | Gizzard_D14_M | 18116364 | 2686852 | 15214616 | 214896 | 85.17 |
| SRR1570213 | Gizzard_D14_M | 10899753 | 1386769 | 8958800 | 554184 | 87.28 |
| SRR1570214 | Gizzard_D14_M | 18071777 | 1525937 | 16293973 | 251867 | 91.56 |
| SRR1570219 | Gizzard_D28_M | 717 | 106 | 598 | 13 | 85.22 |
| SRR1570220 | Gizzard_D28_M | 23507958 | 2783996 | 20389772 | 334190 | 88.16 |
| SRR1570221 | Gizzard_D28_M | 18162922 | 3618220 | 14251886 | 292816 | 80.08 |
| SRR1570222 | Gizzard_D28_M | 13396354 | 1502254 | 11692480 | 201620 | 88.79 |
| SRR1570243 | Thymus_D14_M | 10611043 | 416129 | 10010887 | 184027 | 96.08 |
| SRR1570244 | Thymus_D14_M | 13886003 | 569368 | 13106151 | 210484 | 95.9 |
| SRR1570245 | Thymus_D14_M | 14159584 | 777577 | 13174026 | 207981 | 94.51 |
| SRR1570246 | Thymus_D14_M | 16276374 | 651425 | 15224105 | 400844 | 96 |
| SRR1570250 | Thymus_D28_M | 14738153 | 859306 | 13651411 | 227436 | 94.17 |
| SRR1570251 | Thymus_D28_M | 8304357 | 372628 | 7804905 | 126824 | 95.51 |
| SRR1570272 | Thigh_D14_M | 11067296 | 1863170 | 8385257 | 818869 | 83.17 |
| SRR1570273 | Thigh_D14_M | 11440354 | 1592106 | 8547853 | 1300395 | 86.08 |
| SRR1570274 | Thigh_D14_M | 15900 | 2583 | 11608 | 1709 | 83.75 |
| SRR1570275 | Thigh_D14_M | 15889040 | 2384107 | 11896631 | 1608302 | 85 |
| SRR1570280 | Thigh_D28_M | 8841208 | 1641948 | 6861257 | 338003 | 81.43 |
| SRR1570281 | Thigh_D28_M | 14738588 | 2117385 | 10842910 | 1778293 | 85.63 |
| SRR1570282 | Thigh_D28_M | 11676185 | 1634220 | 8889302 | 1152663 | 86 |
| SRR1570283 | Thigh_D28_M | 15389854 | 2610416 | 11430348 | 1349090 | 83.04 |
| SRR1570284 | Testies_D14_M | 10291093 | 456132 | 9062982 | 771979 | 95.57 |
| SRR1570285 | Testies_D14_M | 16612360 | 806948 | 14561413 | 1243999 | 95.14 |
| SRR1570286 | Testies_D14_M | 18648918 | 938619 | 16095778 | 1614521 | 94.97 |
| SRR1570287 | Testies_D14_M | 15222120 | 757928 | 13410649 | 1053543 | 95.02 |
| SRR1570288 | Testies_D28_M | 17417908 | 820448 | 15459582 | 1137878 | 95.29 |
| SRR1570289 | Testies_D28_M | 12551709 | 619990 | 11105642 | 826077 | 95.06 |
| SRR1570290 | Testies_D28_M | 10954499 | 657102 | 9818545 | 478852 | 94 |
| SRR1570291 | Testies_D28_M | 11176553 | 420155 | 10080114 | 676284 | 96.24 |
| SRR1570312 | Proventriculus_D14_M | 8105954 | 380248 | 7204070 | 521636 | 95.31 |
| SRR1570313 | Proventriculus_D14_M | 9639376 | 1311711 | 7471312 | 856353 | 86.39 |
| SRR1570314 | Proventriculus_D14_M | 13904711 | 2231074 | 10607168 | 1066469 | 83.95 |
| SRR1570315 | Proventriculus_D14_M | 13616829 | 1381221 | 10914618 | 1320990 | 89.86 |
| SRR1570320 | Proventriculus_D28_M | 7126648 | 621878 | 5692213 | 812557 | 91.27 |
| SRR1570321 | Proventriculus_D28_M | 11549691 | 1722063 | 8823933 | 1003695 | 85.09 |
| SRR1570322 | Proventriculus_D28_M | 9965447 | 922162 | 7895811 | 1147474 | 90.75 |
| SRR1570323 | Proventriculus_D28_M | 12625687 | 1218882 | 9948035 | 1458770 | 90.35 |
| SRR1570324 | Proventriculus_D28_M | 12403955 | 1200700 | 9765405 | 1437850 | 90.32 |
| SRR1570345 | Spleen_D14_M | 16333925 | 1111492 | 14961216 | 261217 | 93.2 |
| SRR1570346 | Spleen_D14_M | 11677861 | 786605 | 10702646 | 188610 | 93.26 |
| SRR1570347 | Spleen_D14_M | 11550529 | 745376 | 10621730 | 183423 | 93.55 |
| SRR1570348 | Spleen_D14_M | 14156741 | 947043 | 12084725 | 1124973 | 93.31 |
| SRR1570353 | Spleen_D28_M | 10171696 | 1329324 | 8613366 | 229006 | 86.93 |
| SRR1570354 | Spleen_D28_M | 12616921 | 877116 | 11420809 | 318996 | 93.05 |
| SRR1570355 | Spleen_D28_M | 9668237 | 702020 | 8687793 | 278424 | 92.74 |
| SRR1570376 | Pancreas_D14_M | 17566059 | 1268541 | 11156689 | 5140829 | 92.78 |
| SRR1570377 | Pancreas_D14_M | 20499451 | 1376127 | 13125246 | 5998078 | 93.29 |
| SRR1570378 | Pancreas_D14_M | 17835492 | 1170380 | 11285445 | 5379667 | 93.44 |
| SRR1570379 | Pancreas_D14_M | 22593692 | 1260265 | 14501542 | 6831885 | 94.42 |
| SRR1570384 | Pancreas_D28_M | 17341022 | 910237 | 11001016 | 5429769 | 94.75 |
| SRR1570385 | Pancreas_D28_M | 17155106 | 1209670 | 11934932 | 4010504 | 92.95 |
| SRR1570386 | Pancreas_D28_M | 26155312 | 1591837 | 16297722 | 8265753 | 93.91 |
| SRR1570387 | Pancreas_D28_M | 13217071 | 914113 | 8231127 | 4071831 | 93.08 |
| SRR1570416 | Jejunum_D14_M | 14704077 | 1133087 | 13271434 | 299556 | 92.29 |
| SRR1570417 | Jejunum_D14_M | 21070721 | 3339440 | 17257644 | 473637 | 84.15 |
| SRR1570418 | Jejunum_D14_M | 14991443 | 1306411 | 13319418 | 365614 | 91.29 |
| SRR1570419 | Jejunum_D14_M | 21421194 | 1366889 | 19256822 | 797483 | 93.62 |
| SRR1570424 | Jejunum_D28_M | 19412962 | 1885451 | 17097716 | 429795 | 90.29 |
| SRR1570425 | Jejunum_D28_M | 16745991 | 2301963 | 13910461 | 533567 | 86.25 |
| SRR1570426 | Jejunum_D28_M | 19688505 | 1994163 | 17203910 | 490432 | 89.87 |
| SRR1570427 | Jejunum_D28_M | 26381361 | 2187936 | 23037359 | 1156066 | 91.71 |
| SRR1570448 | Ileum_D14_M | 15339255 | 583691 | 14335954 | 419610 | 96.19 |
| SRR1570449 | Ileum_D14_M | 19774598 | 1374648 | 17890981 | 508969 | 93.05 |
| SRR1570450 | Ileum_D14_M | 12880983 | 868481 | 11759675 | 252827 | 93.26 |
| SRR1570451 | Ileum_D14_M | 17423310 | 1309358 | 15645977 | 467975 | 92.49 |
| SRR1570456 | Ileum_D28_M | 15280824 | 1206563 | 13594433 | 479828 | 92.1 |
| SRR1570457 | Ileum_D28_M | 16085711 | 1807203 | 13973866 | 304642 | 88.77 |
| SRR1570458 | Ileum_D28_M | 17600794 | 1353077 | 15812390 | 435327 | 92.31 |
| SRR1570459 | Ileum_D28_M | 10776435 | 762775 | 9699355 | 314305 | 92.92 |
| SRR1570479 | Heart_D14_M | 18484864 | 5911488 | 12060297 | 513079 | 68.02 |
| SRR1570480 | Heart_D14_M | 22638961 | 6166929 | 15644770 | 827262 | 72.76 |
| SRR1570481 | Heart_D14_M | 17130404 | 5465238 | 11278825 | 386341 | 68.1 |
| SRR1570482 | Heart_D14_M | 14475571 | 5618365 | 8529523 | 327683 | 61.19 |
| SRR1570487 | Heart_D28_M | 15356119 | 4904222 | 10052959 | 398938 | 68.06 |
| SRR1570488 | Heart_D28_M | 17392041 | 5963845 | 10759686 | 668510 | 65.71 |
| SRR1570489 | Heart_D28_M | 18069822 | 3946840 | 12887722 | 1235260 | 78.16 |
| SRR1570509 | Duodenum_D14_M | 25030248 | 3280079 | 21194133 | 556036 | 86.9 |
| SRR1570510 | Duodenum_D14_M | 17888568 | 2593204 | 14870626 | 424738 | 85.5 |
| SRR1570511 | Duodenum_D14_M | 22211069 | 2304579 | 19338228 | 568262 | 89.62 |
| SRR1570512 | Duodenum_D14_M | 27486526 | 3513863 | 23181408 | 791255 | 87.22 |
| SRR1570516 | Duodenum_D28_M | 23819844 | 3175294 | 20057962 | 586588 | 86.67 |
| SRR1570517 | Duodenum_D28_M | 14693978 | 1748896 | 12630782 | 314300 | 88.1 |
| SRR1570518 | Duodenum_D28_M | 9531405 | 2128117 | 7164263 | 239025 | 77.67 |
| SRR1570519 | Duodenum_D28_M | 13079104 | 4875958 | 7794505 | 408641 | 62.72 |
| SRR1570539 | Cecaltonsil_D14_M | 11332863 | 346504 | 10794750 | 191609 | 96.94 |
| SRR1570540 | Cecaltonsil_D14_M | 10877037 | 2118445 | 8556420 | 202172 | 80.52 |
| SRR1570541 | Cecaltonsil_D14_M | 11191194 | 818324 | 10159930 | 212940 | 92.69 |
| SRR1570542 | Cecaltonsil_D14_M | 15017628 | 1216119 | 13485007 | 316502 | 91.9 |
| SRR1570564 | Bursa_D14_M | 11500418 | 973394 | 6994935 | 3532089 | 91.54 |
| SRR1570565 | Bursa_D14_M | 14609285 | 945322 | 12738805 | 925158 | 93.53 |
| SRR1570566 | Bursa_D14_M | 15875406 | 1282244 | 14424983 | 168179 | 91.92 |
| SRR1570567 | Bursa_D14_M | 19308949 | 407448 | 15021488 | 3880013 | 97.89 |
| SRR1570571 | Bursa_D28_M | 14416585 | 653726 | 6947991 | 6814868 | 95.47 |
| SRR1570572 | Bursa_D28_M | 19664715 | 1419200 | 18023354 | 222161 | 92.78 |
| SRR1570593 | Brain_D14_M | 15269278 | 1962359 | 12997128 | 309791 | 87.15 |
| SRR1570594 | Brain_D14_M | 8329351 | 1221333 | 6940949 | 167069 | 85.34 |
| SRR1570595 | Brain_D14_M | 13412813 | 1032836 | 12048509 | 331468 | 92.3 |
| SRR1570600 | Brain_D21_M | 14817236 | 1852543 | 12574139 | 390554 | 87.5 |
| SRR1570601 | Brain_D21_M | 11873796 | 2486871 | 8994456 | 392469 | 79.06 |
| SRR1570602 | Brain_D21_M | 10158280 | 1026149 | 7999945 | 1132186 | 89.9 |
| SRR1570603 | Brain_D21_M | 11744437 | 1564238 | 9936841 | 243358 | 86.68 |
| SRR1570608 | Brain_D28_M | 15973833 | 2784379 | 12801223 | 388231 | 82.57 |
| SRR1570609 | Brain_D28_M | 9987839 | 1525599 | 8299172 | 163068 | 84.73 |
| SRR1570610 | Brain_D28_M | 7989375 | 1075494 | 6706088 | 207793 | 86.54 |
| SRR1570611 | Brain_D28_M | 9486277 | 1462033 | 7778367 | 245877 | 84.59 |
| SRR1570632 | Liver_D14_M | 17769974 | 2318772 | 14927998 | 523204 | 86.95 |
| SRR1570633 | Liver_D14_M | 21123365 | 2923179 | 17649575 | 550611 | 86.16 |
| SRR1570634 | Liver_D14_M | 30567951 | 3602222 | 26265058 | 700671 | 88.22 |
| SRR1570635 | Liver_D14_M | 2616208 | 101662 | 2421888 | 92658 | 96.11 |
| SRR1570640 | Liver_D21_M | 19536192 | 2539960 | 16360833 | 635399 | 87 |
| SRR1570641 | Liver_D21_M | 20350315 | 3066252 | 16779925 | 504138 | 84.93 |
| SRR1570642 | Liver_D21_M | 13524327 | 1548065 | 11557385 | 418877 | 88.55 |
| SRR1570643 | Liver_D21_M | 12398008 | 1599283 | 10530493 | 268232 | 87.1 |
| SRR1570648 | Liver_D28_M | 16727592 | 2127127 | 13992860 | 607605 | 87.28 |
| SRR1570649 | Liver_D28_M | 19984930 | 2424281 | 16983332 | 577317 | 87.87 |
| SRR1570650 | Liver_D28_M | 38345151 | 5100613 | 32261006 | 983532 | 86.7 |
| SRR1570651 | Liver_D28_M | 18992592 | 2101725 | 16257711 | 633156 | 88.93 |
| SRR1570655 | Bursa_D21_M | 14946172 | 1121620 | 13544025 | 280527 | 92.5 |
| SRR1570656 | Bursa_D21_M | 15998205 | 1178033 | 14560230 | 259942 | 92.64 |
| SRR1570657 | Bursa_D21_M | 15924728 | 1250152 | 14418249 | 256327 | 92.15 |
| SRR1570662 | Cecaltonsil_D21_M | 11230735 | 1694509 | 9249767 | 286459 | 84.91 |
| SRR1570663 | Cecaltonsil_D21_M | 23816803 | 2114228 | 19434901 | 2267674 | 91.12 |
| SRR1570664 | Cecaltonsil_D21_M | 12587641 | 2379373 | 9987210 | 221058 | 81.1 |
| SRR1570669 | Duodenum_D21_M | 24752869 | 2697157 | 21455067 | 600645 | 89.1 |
| SRR1570670 | Duodenum_D21_M | 19874160 | 2455661 | 16972372 | 446127 | 87.64 |
| SRR1570671 | Duodenum_D21_M | 16136994 | 2168195 | 13519696 | 449103 | 86.56 |
| SRR1570676 | Gizzard_D21_M | 18722970 | 2236622 | 16273743 | 212605 | 88.05 |
| SRR1570677 | Gizzard_D21_M | 35646337 | 6963092 | 28254097 | 429148 | 80.47 |
| SRR1570678 | Gizzard_D21_M | 12319807 | 2061816 | 10070278 | 187713 | 83.26 |
| SRR1570679 | Gizzard_D21_M | 24944841 | 3909575 | 20674169 | 361097 | 84.33 |
| SRR1570684 | Heart_D21_M | 21086553 | 6969250 | 13551428 | 565875 | 66.95 |
| SRR1570685 | Heart_D21_M | 20450072 | 5222670 | 14568728 | 658674 | 74.46 |
| SRR1570686 | Heart_D21_M | 16172876 | 2563146 | 13081417 | 528313 | 84.15 |
| SRR1570687 | Heart_D21_M | 13381562 | 4116525 | 8891760 | 373277 | 69.24 |
| SRR1570692 | Ileum_D21_M | 16739336 | 1386931 | 15058104 | 294301 | 91.71 |
| SRR1570693 | Ileum_D21_M | 17623899 | 1450681 | 15848254 | 324964 | 91.77 |
| SRR1570694 | Ileum_D21_M | 18413501 | 1449217 | 16620760 | 343524 | 92.13 |
| SRR1570695 | Ileum_D21_M | 15429467 | 1335656 | 13834384 | 259427 | 91.34 |
| SRR1570700 | Jejunum_D21_M | 12573901 | 1072344 | 11177738 | 323819 | 91.47 |
| SRR1570701 | Jejunum_D21_M | 16220020 | 1435092 | 14322270 | 462658 | 91.15 |
| SRR1570702 | Jejunum_D21_M | 16740482 | 1369811 | 14916607 | 454064 | 91.82 |
| SRR1570703 | Jejunum_D21_M | 6697517 | 524194 | 5958999 | 214324 | 92.17 |
| SRR1570725 | Breast_D14_M | 8398350 | 1008640 | 7095218 | 294492 | 87.99 |
| SRR1570726 | Breast_D14_M | 11242282 | 1051823 | 9764034 | 426425 | 90.64 |
| SRR1570727 | Breast_D14_M | 10604446 | 1212365 | 9082105 | 309976 | 88.57 |
| SRR1570728 | Breast_D14_M | 11058025 | 1453627 | 9253025 | 351373 | 86.85 |
| SRR1570733 | Breast_D28_M | 10922458 | 1331535 | 9285913 | 305010 | 87.81 |
| SRR1570734 | Breast_D28_M | 10778225 | 1229415 | 9147354 | 401456 | 88.59 |
| SRR1570735 | Breast_D28_M | 11308550 | 1408348 | 9560154 | 340048 | 87.55 |
| SRR1570736 | Breast_D28_M | 11349746 | 1463991 | 9552223 | 333532 | 87.1 |
| SRR1661425 | Breast_D21_M | 11188693 | 1049761 | 9675323 | 463609 | 90.62 |
| SRR1661426 | Breast_D21_M | 11859847 | 1661955 | 9877255 | 320637 | 85.99 |
| SRR1661427 | Breast_D21_M | 12110962 | 2319775 | 9510160 | 281027 | 80.85 |
| SRR1661428 | Breast_D21_M | 11889516 | 1666043 | 9901052 | 322421 | 85.99 |
| SRR1661433 | Pancreas_D21_M | 17428117 | 1137442 | 11144878 | 5145797 | 93.47 |
| SRR1661434 | Pancreas_D21_M | 14988277 | 873128 | 9836760 | 4278389 | 94.17 |
| SRR1661435 | Pancreas_D21_M | 19066483 | 1394323 | 12196558 | 5475602 | 92.69 |
| SRR1661436 | Pancreas_D21_M | 16883792 | 1087137 | 10824204 | 4972451 | 93.56 |
| SRR1661440 | Proventriculus_D21_M | 10856366 | 2400222 | 6526922 | 1929222 | 77.89 |
| SRR1661441 | Proventriculus_D21_M | 11951154 | 1348025 | 9213772 | 1389357 | 88.72 |
| SRR1661442 | Proventriculus_D21_M | 8496342 | 1030792 | 6626530 | 839020 | 87.87 |
| SRR1661443 | Proventriculus_D21_M | 7611077 | 872185 | 5887181 | 851711 | 88.54 |
| SRR1661447 | Spleen_D21_M | 20932512 | 1702163 | 18438420 | 791929 | 91.87 |
| SRR1661448 | Spleen_D21_M | 12465746 | 1801620 | 10166575 | 497551 | 85.55 |
| SRR1661453 | Thigh_D21_M | 9634946 | 2036072 | 7265832 | 333042 | 78.87 |
| SRR1661454 | Thigh_D21_M | 13366087 | 2349893 | 9731089 | 1285105 | 82.42 |
| SRR1661455 | Thigh_D21_M | 867 | 142 | 646 | 79 | 83.62 |
| SRR1661456 | Thigh_D21_M | 15137603 | 2798014 | 11139170 | 1200419 | 81.52 |
| SRR1661460 | Thymus_D21_M | 15075023 | 910159 | 13886951 | 277913 | 93.96 |
| SRR1661461 | Thymus_D21_M | 12886076 | 777335 | 11905204 | 203537 | 93.97 |
| SRR1661462 | Thymus_D21_M | 13917031 | 976377 | 11936181 | 1004473 | 92.98 |

**Table S7:** Mummer alignment between Turkey5.1 and Mgal_WUR_HG_1.0 of the first and the second breakpoint of the 19.4 Mbp inversion on the Z-chromosome.

| **Turkey5.1_Start** | **Turkey5.1_End** | **Mgal_WUR_HG_1.0_Start** | **Mgal_WUR_HG_1.0_Start** | **Length** | **%Identity** |
| --- | --- | --- | --- | --- | --- |
| **First breakpoint** | | | | | |
| 40121260 | 40127870 | 44295541 | 44302151 | 6611 | 99.98 |
| 40127971 | 40134369 | 44353506 | 44359904 | 6399 | 100 |
| 40134470 | 40144288 | 44483105 | 44492923 | 9819 | 99.99 |
| 40144389 | 40169418 | 44457443 | 44482474 | 25032 | 99.99 |
| 40169519 | 40189304 | 44437652 | 44457441 | 19790 | 99.97 |
| 40189405 | 40209568 | 44417492 | 44437656 | 20165 | 99.99 |
| 40209669 | 40228147 | 44397423 | 44415902 | 18480 | 99.94 |
| 40228248 | 40239787 | 44385088 | 44396628 | 11541 | 99.97 |
| 40239888 | 40248234 | 44376286 | 44384632 | 8347 | 99.98 |
| 40248335 | 40253764 | 44370579 | 44376008 | 5430 | 99.59 |
| 40254062 | 40260253 | 44364370 | 44370560 | 6191 | 99.87 |
| 40260354 | 40264802 | 44359912 | 44364377 | 4466 | 99.57 |
| 40264903 | 40267829 | 63916528 | 63913604 | 2925 | 99.8 |
| 40268541 | 40271908 | 63920831 | 63917458 | 3374 | 99.82 |
| 40272009 | 40280004 | 63913732 | 63905736 | 7997 | 99.99 |
| 40280105 | 40295769 | 63905721 | 63890038 | 15684 | 99.87 |
| 40295870 | 40298111 | 63890011 | 63887770 | 2242 | 100 |
| 40298212 | 40330900 | 63887771 | 63855075 | 32697 | 99.98 |
| 40331001 | 40337675 | 63855083 | 63848409 | 6675 | 99.99 |
| 40337776 | 40346850 | 63848389 | 63839315 | 9075 | 100 |
| 40347377 | 40353592 | 63838440 | 63832218 | 6223 | 99.82 |
| 40355968 | 40359491 | 63831632 | 63828109 | 3524 | 100 |
| 40362300 | 40365901 | 63825734 | 63822131 | 3604 | 99.94 |
| 40366189 | 40378393 | 63822097 | 63809893 | 12205 | 99.99 |
| **Second breakpoint** | | | | | |
| 57336646 | 57365205 | 44619159 | 44590594 | 28566 | 99.98 |
| 57365306 | 57373630 | 44590597 | 44582272 | 8326 | 99.96 |
| 57377541 | 57383567 | 44579735 | 44573709 | 6027 | 100 |
| 57383668 | 57411999 | 44573598 | 44545231 | 28368 | 99.87 |
| 57413030 | 57417984 | 44545231 | 44540276 | 4956 | 99.98 |
| 57418860 | 57447388 | 44539087 | 44510552 | 28536 | 99.97 |
| 57447489 | 57457363 | 44510554 | 44500680 | 9875 | 100 |
| 57461280 | 57465090 | 44498672 | 44494862 | 3811 | 99.97 |
| 57465191 | 57467389 | 63973119 | 63975317 | 2199 | 100 |
| 57467490 | 57472466 | 63975325 | 63980305 | 4981 | 99.88 |
| 57472567 | 57474310 | 63980356 | 63982101 | 1746 | 99.89 |
| 57474413 | 57479621 | 64005086 | 64010284 | 5199 | 99.65 |
| 57478817 | 57480196 | 64074800 | 64073420 | 1381 | 99.13 |
| 57480835 | 57487562 | 63988814 | 63982081 | 6734 | 99.91 |
| 57487663 | 57488898 | 63990070 | 63988835 | 1236 | 99.84 |
| 57488999 | 57495069 | 63995921 | 63989850 | 6072 | 99.51 |


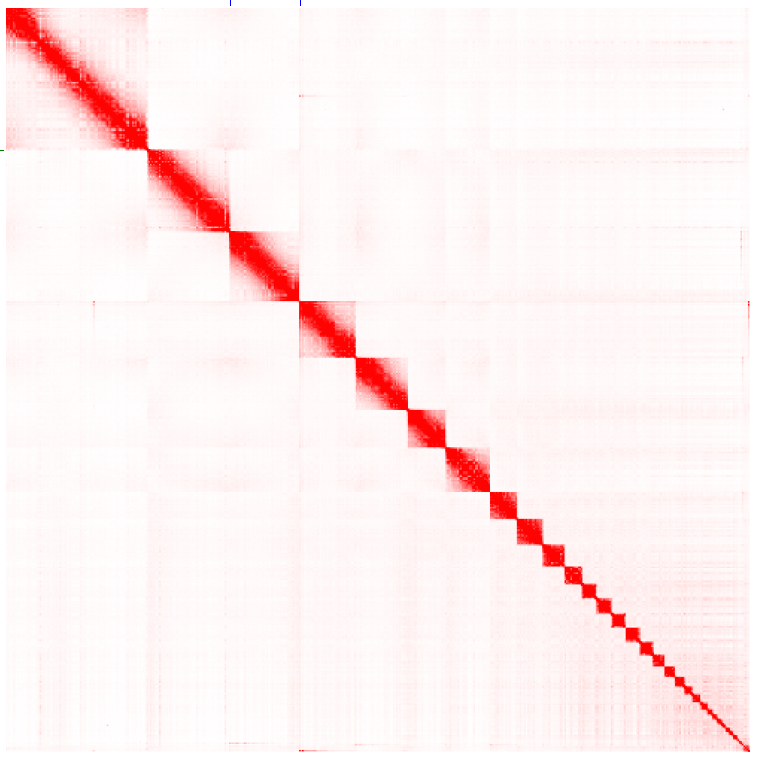


**Figure S1:** Hi-C contact map of the Mgal_WU_HG_1.0 assembly.


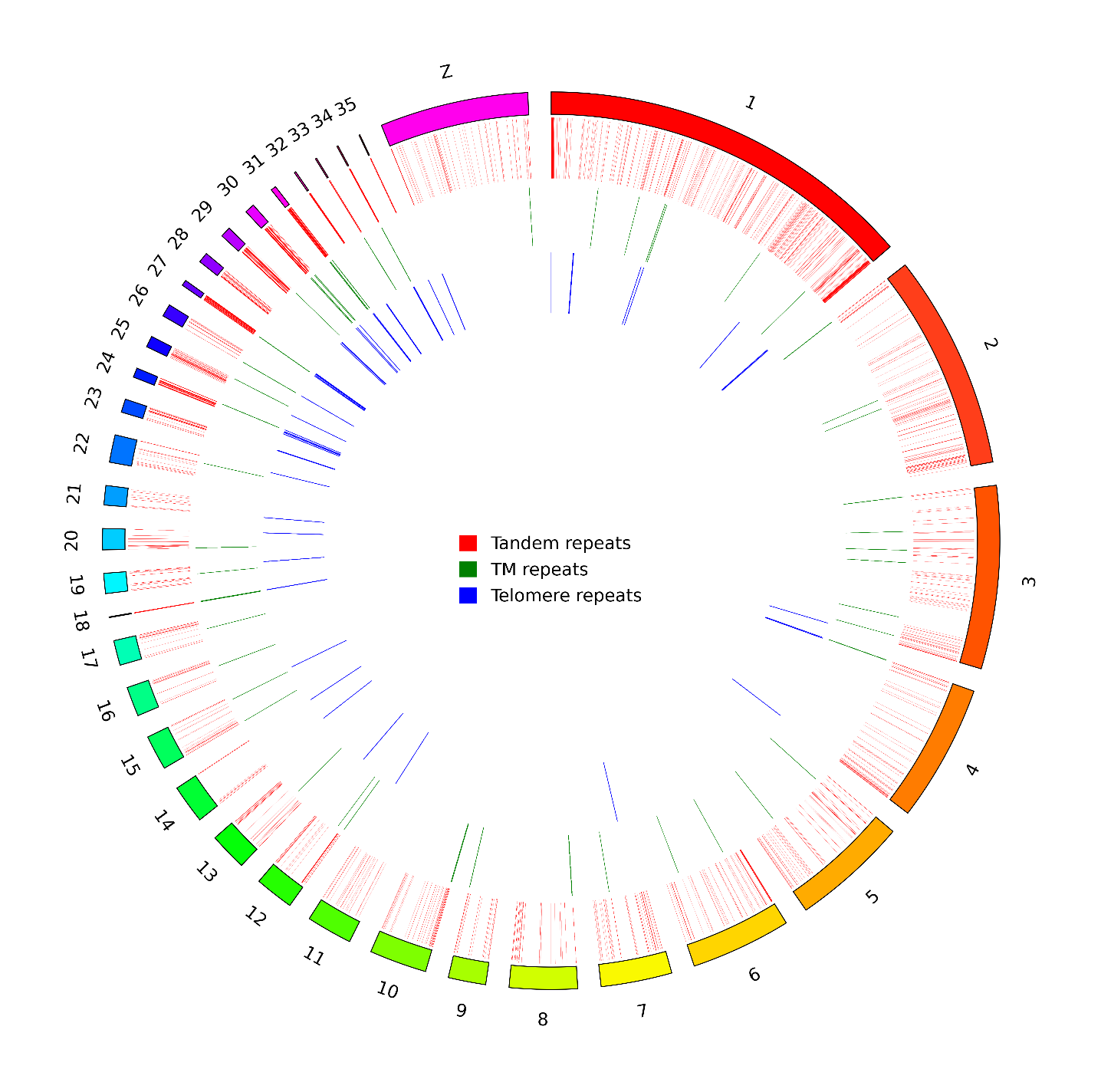


**Figure S2:** Overview of tandem repeats identified in the turkey genome. Red bands show tandem repeat clusters of at least 500bp in size. TM repeats are shown in green and telomeric TTAGGG repeats are shown in blue.


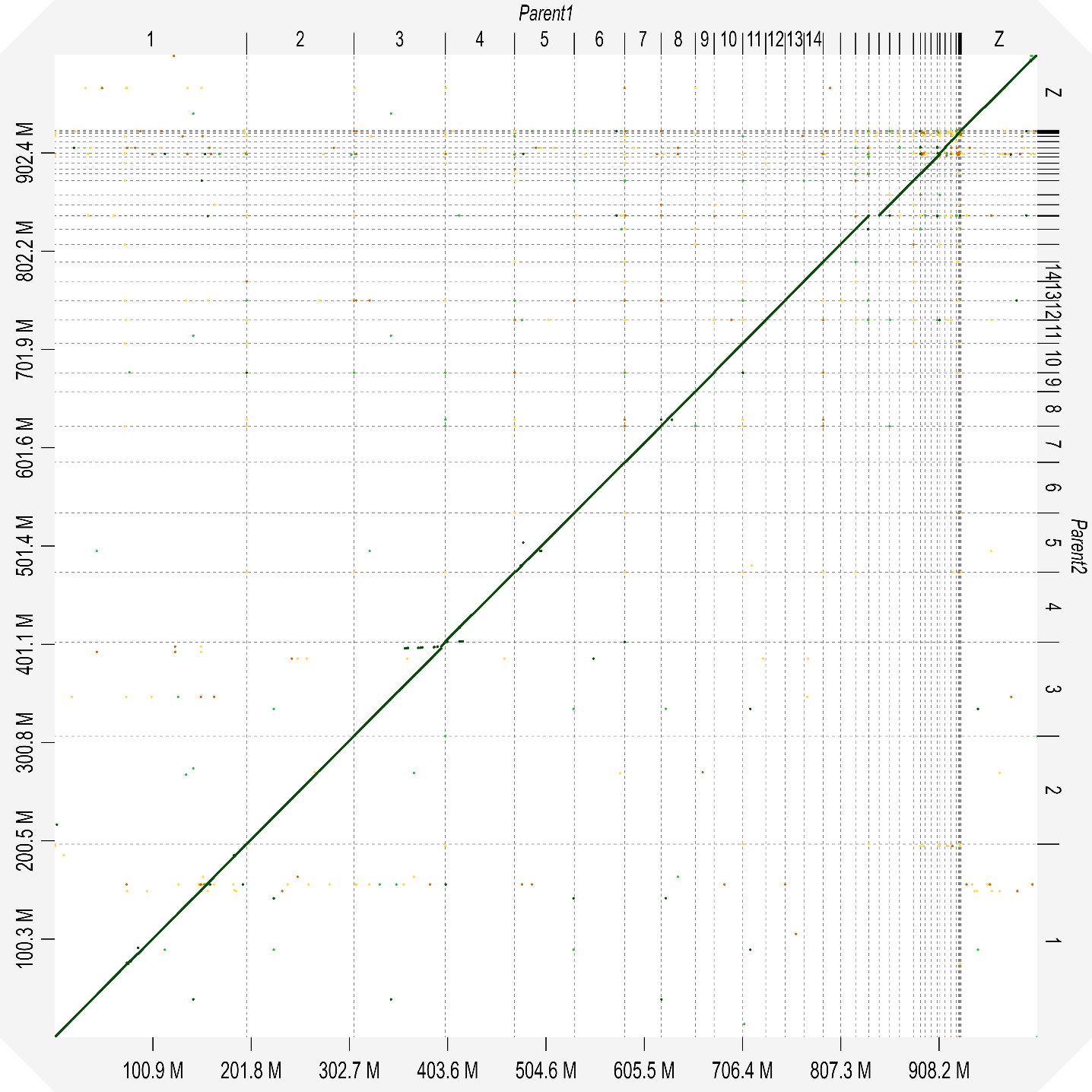


**Figure S3:** Parent 1 vs. parent 2 haplotype alignment.


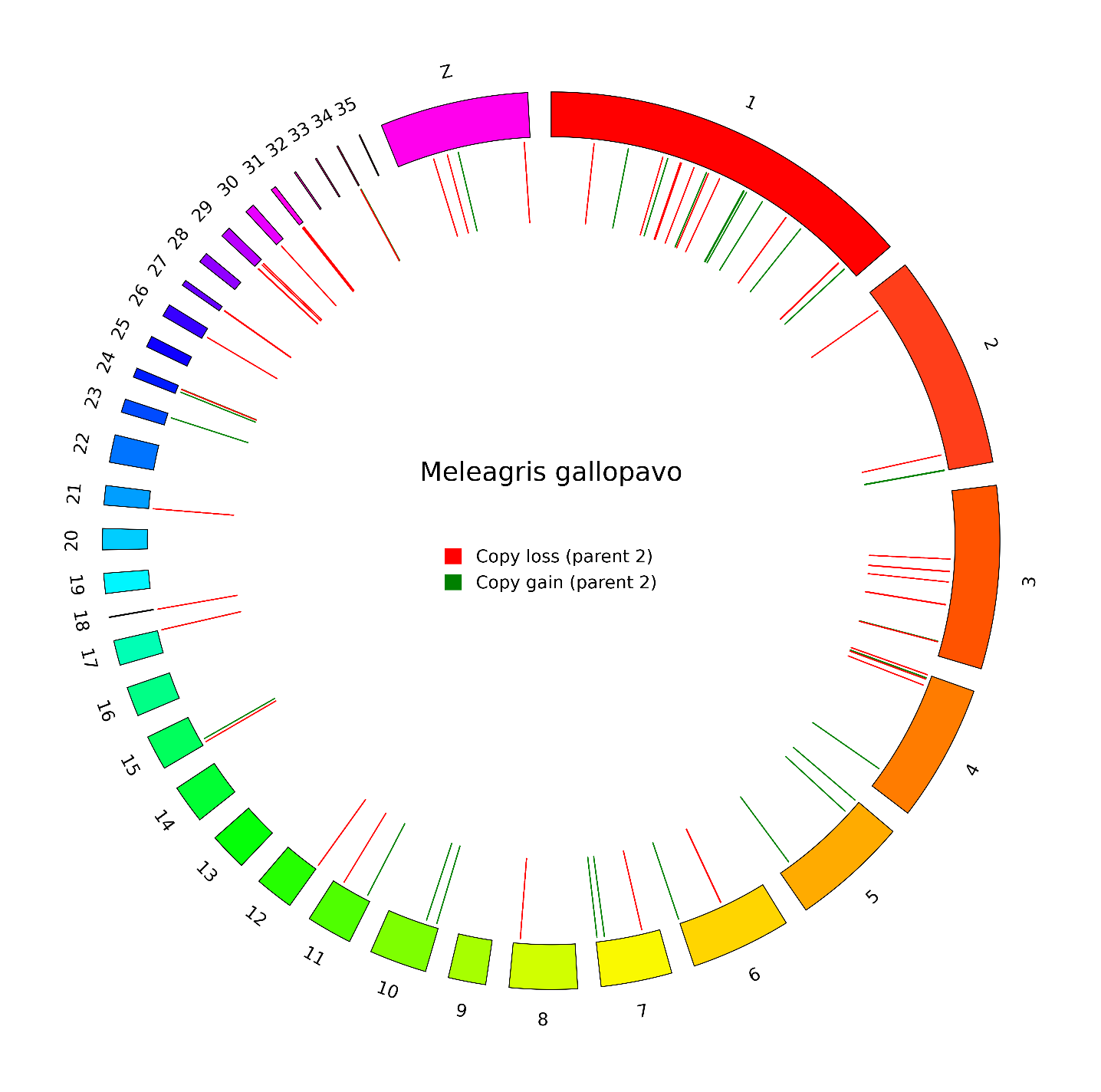


**Figure S4:** Distribution of copy gains and copy losses in parent 2 compared to parent 1 haplotype.


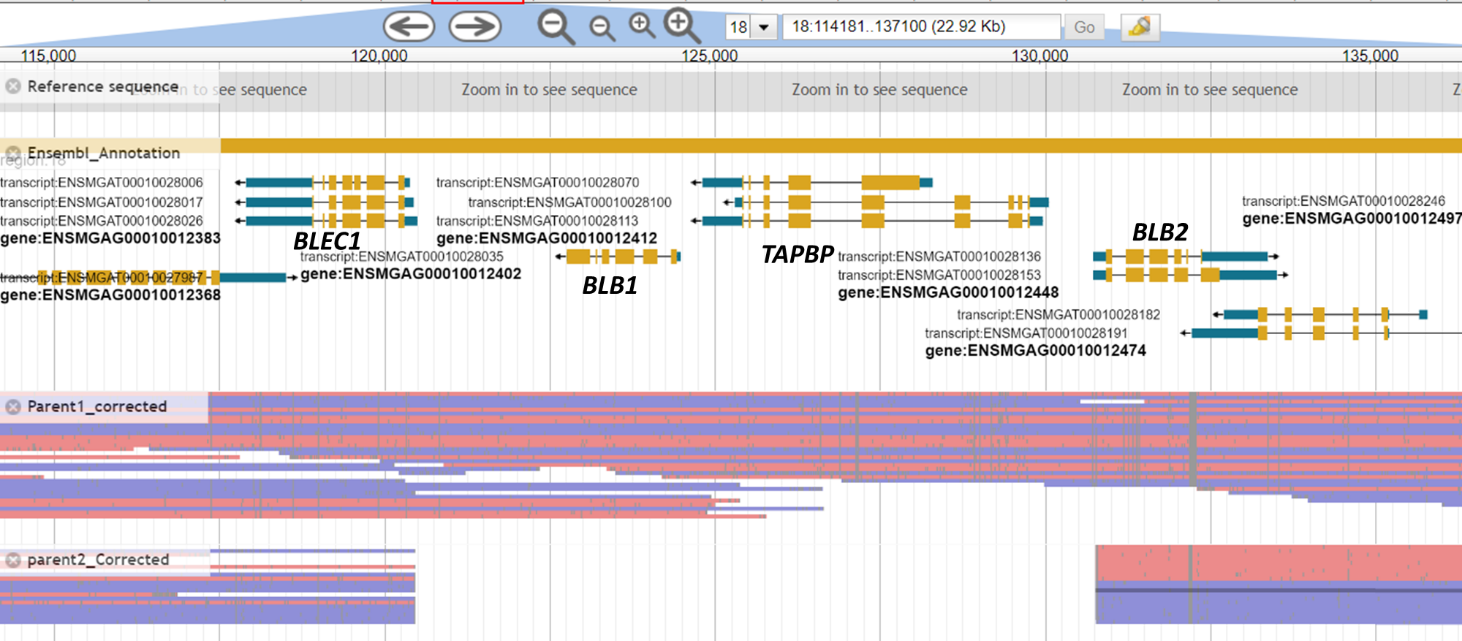


**Figure S5:** Inversion comprising the start of the *BLB2* gene in parent2 compared to the parent1 haplotype.


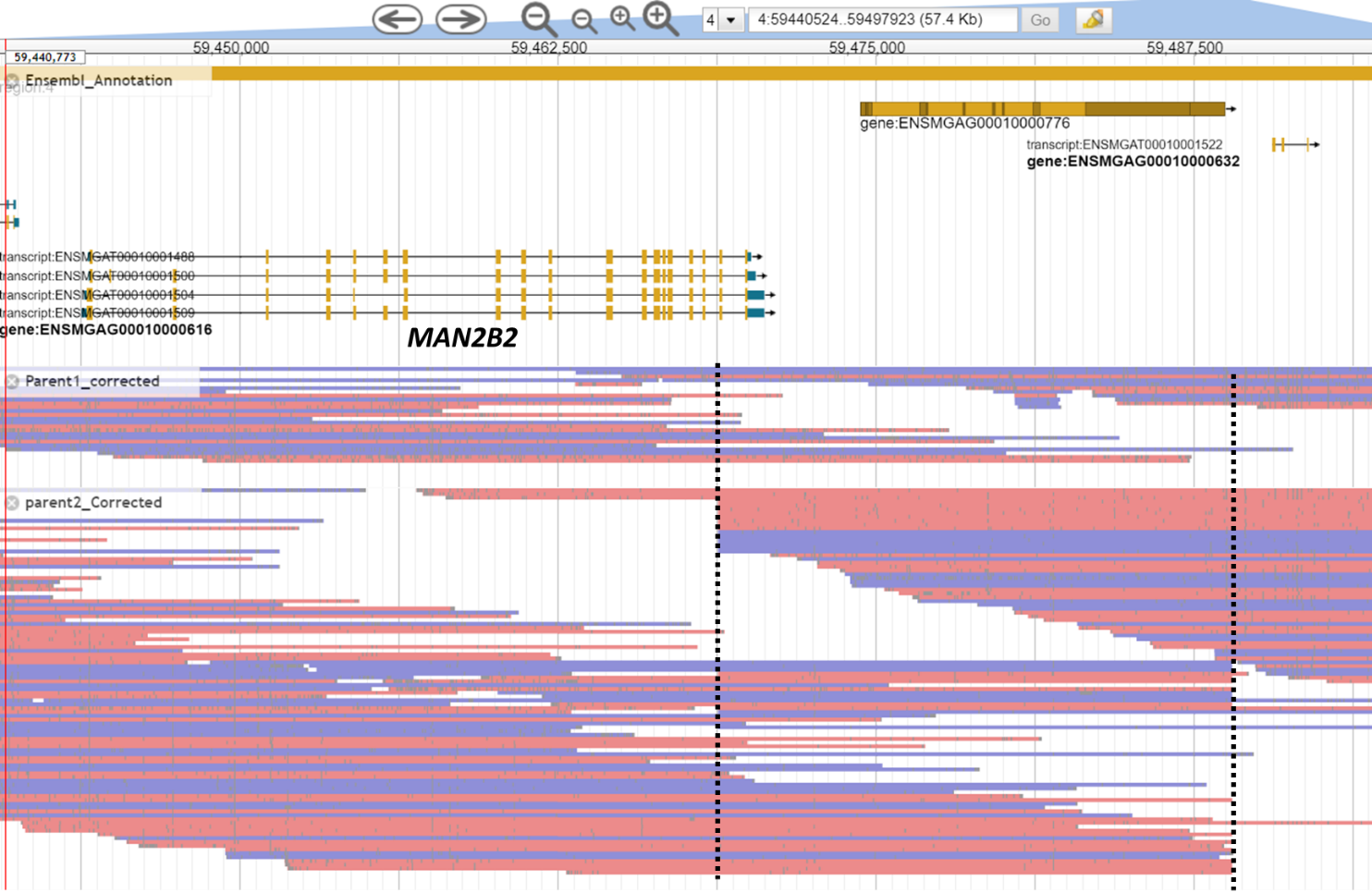


**Figure S6:** Duplication affecting the tail of the *MAN2B2* gene in parent2 compared to the parent1 haplotype.


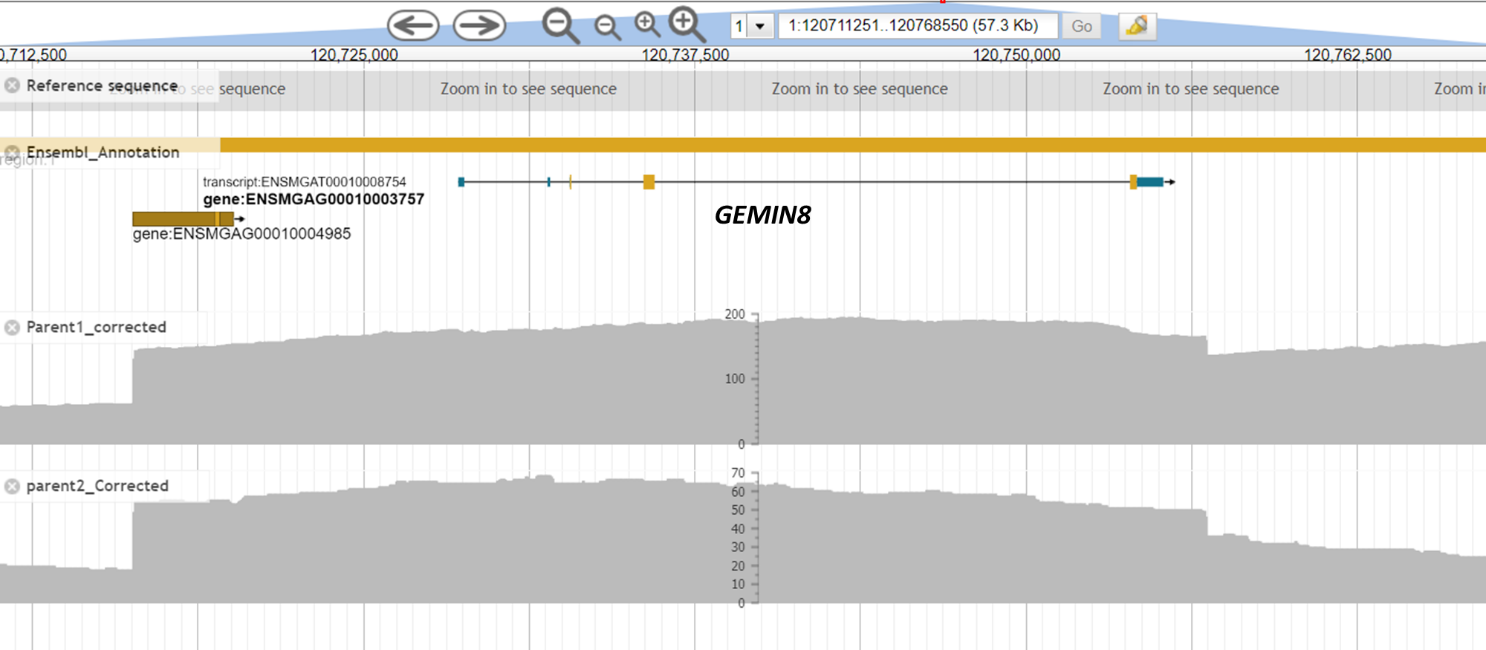


**Figure S7:** Duplication affecting *GEMIN8* gene in parent2 compared to the parent1 haplotype.


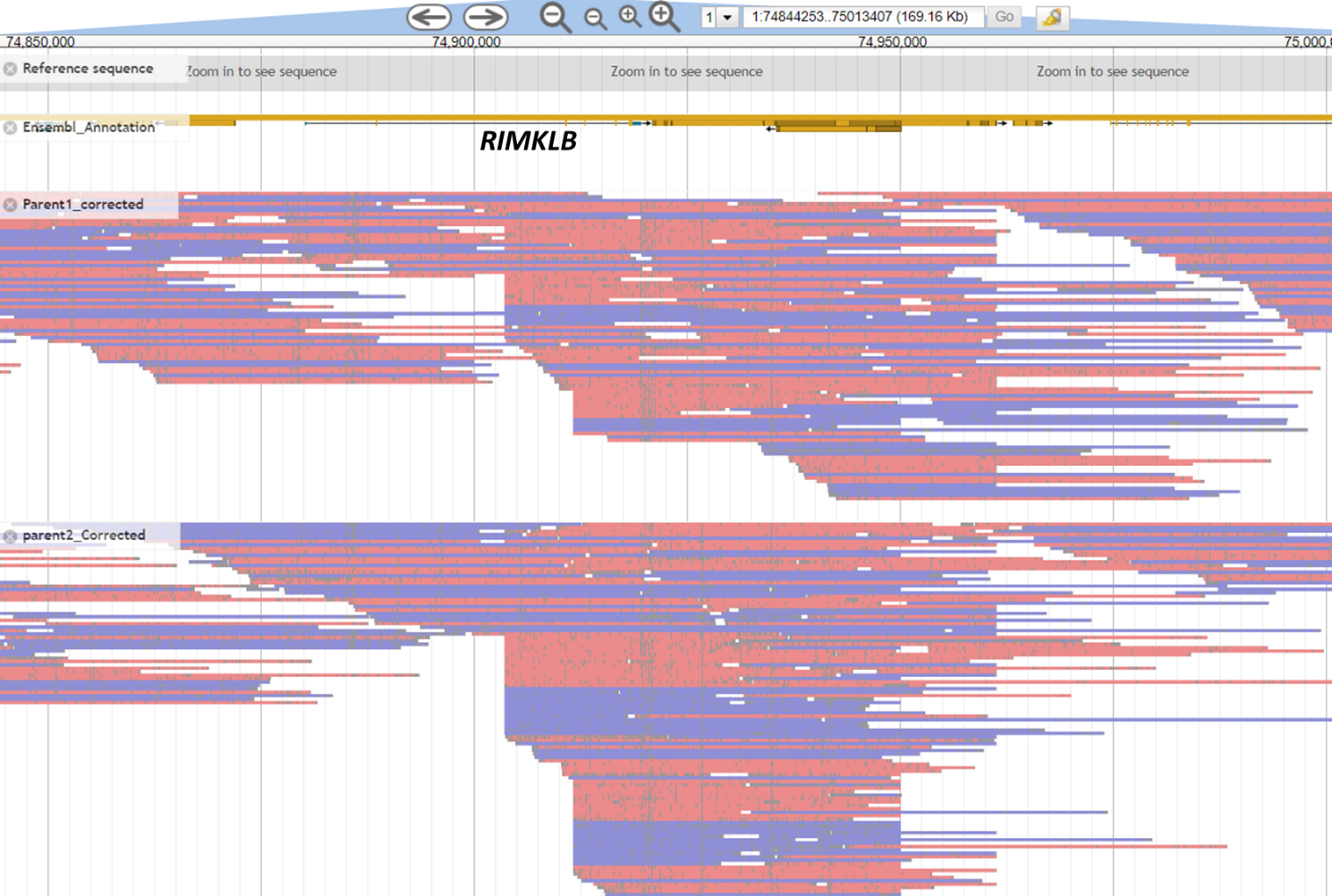


**Figure S8:** Duplication affecting *RIMKLB* gene with higher copy number in parent 2 compared to parent1 haplotype.


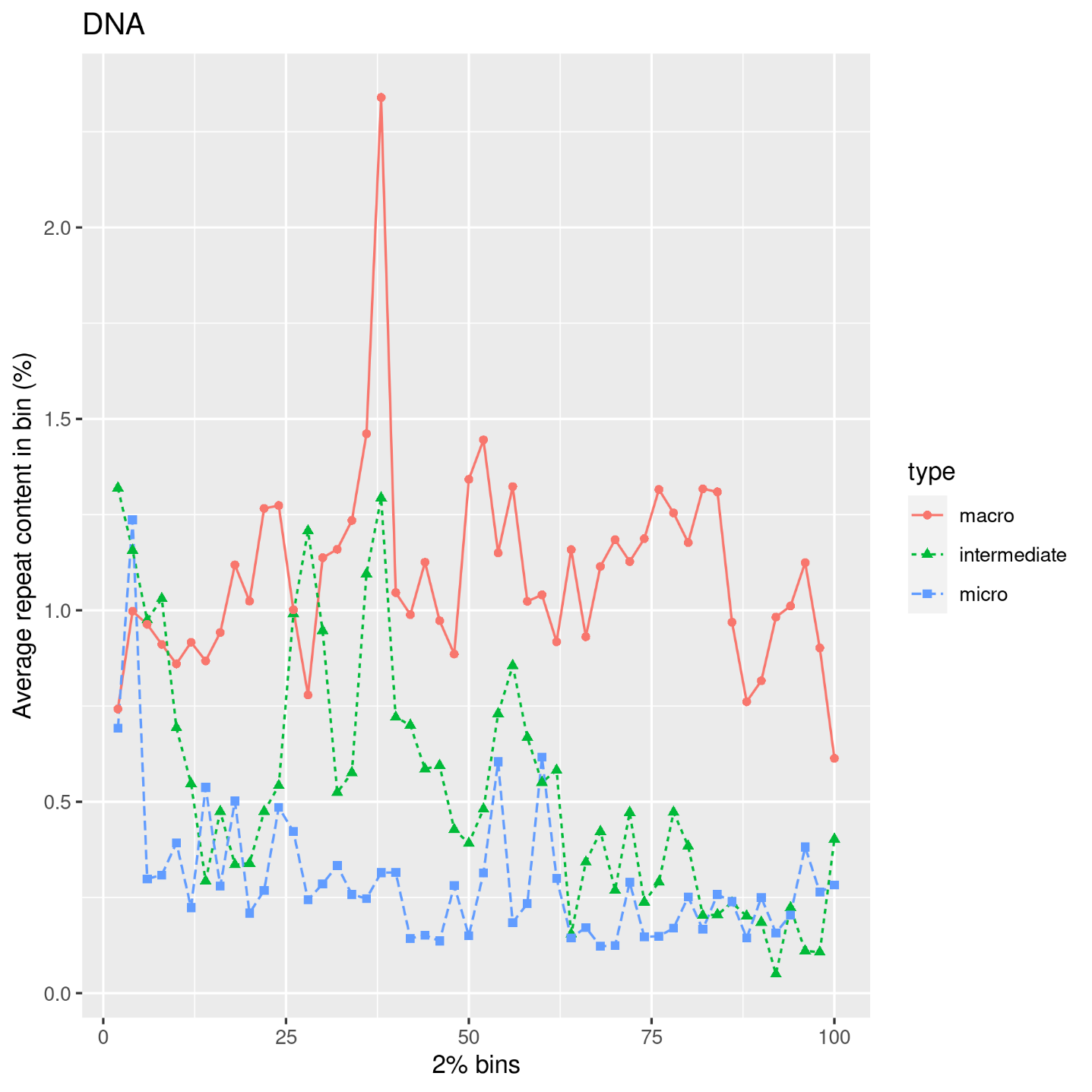


**Figure S9:** Average DNA repeat content along the chromosomes for macro, intermediate and microcromosomes.


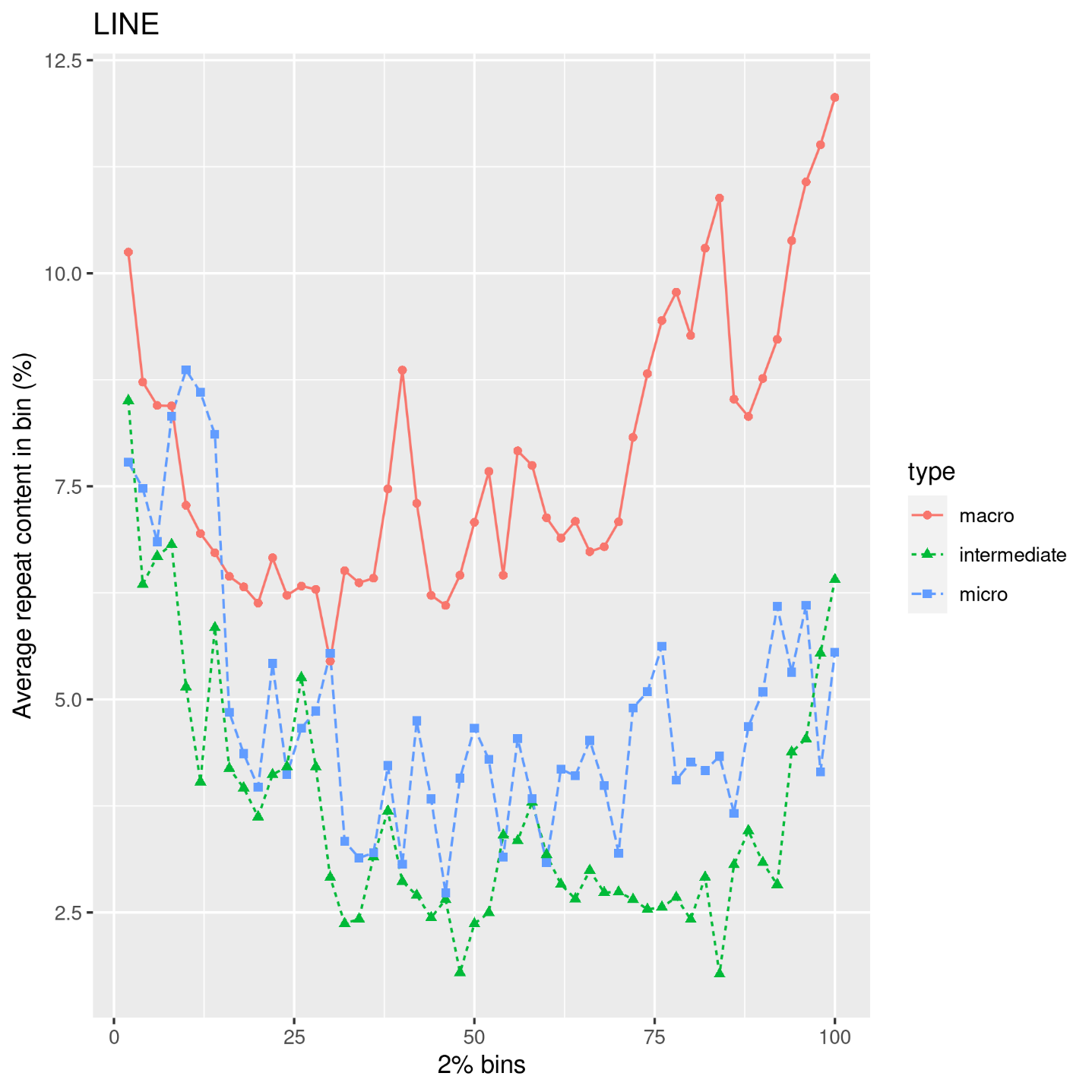


**Figure S10:** Average LINE repeat content along the chromosomes for macro, intermediate and microcromosomes.


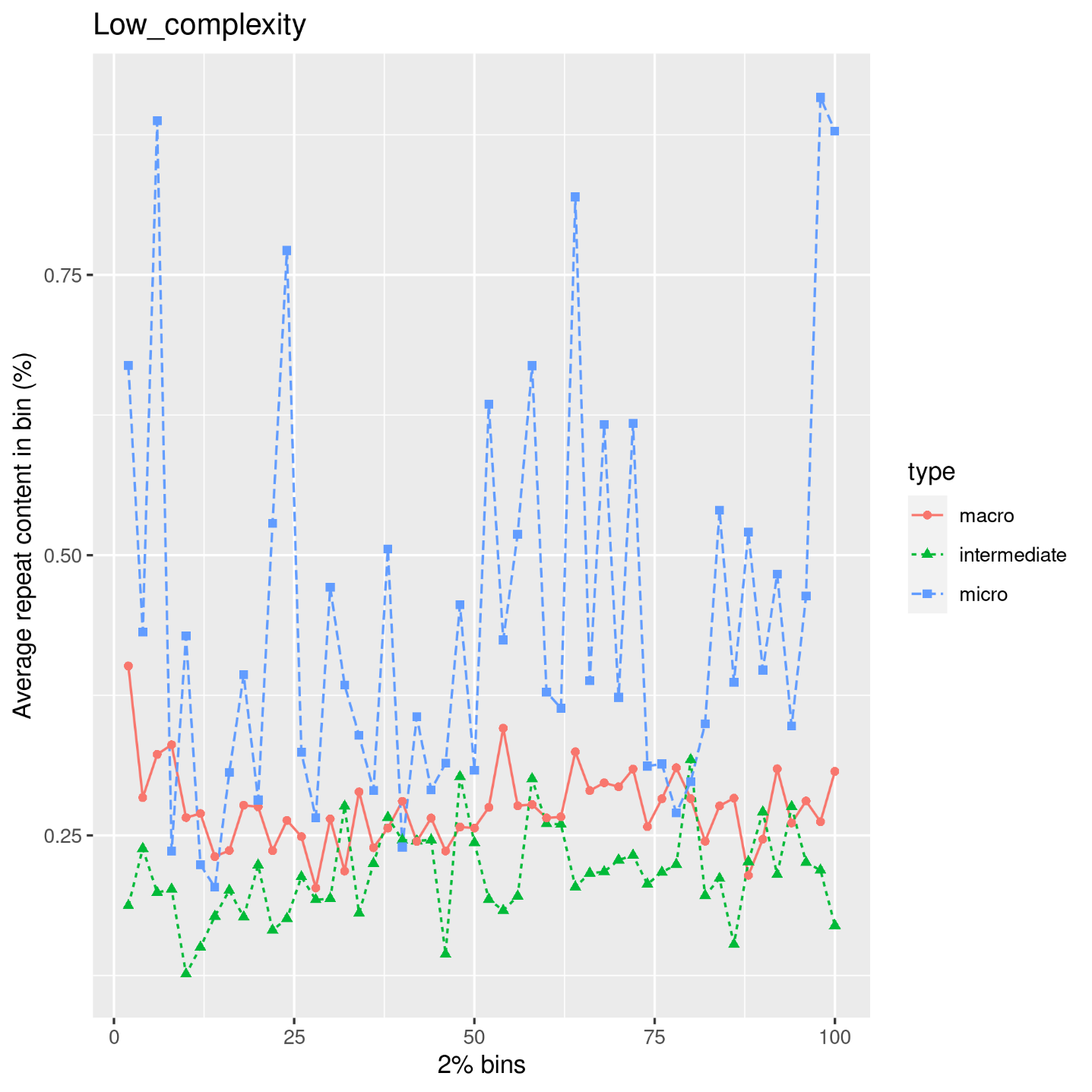


**Figure S11:** Average low complexity repeat content along the chromosomes for macro, intermediate and microcromosomes.


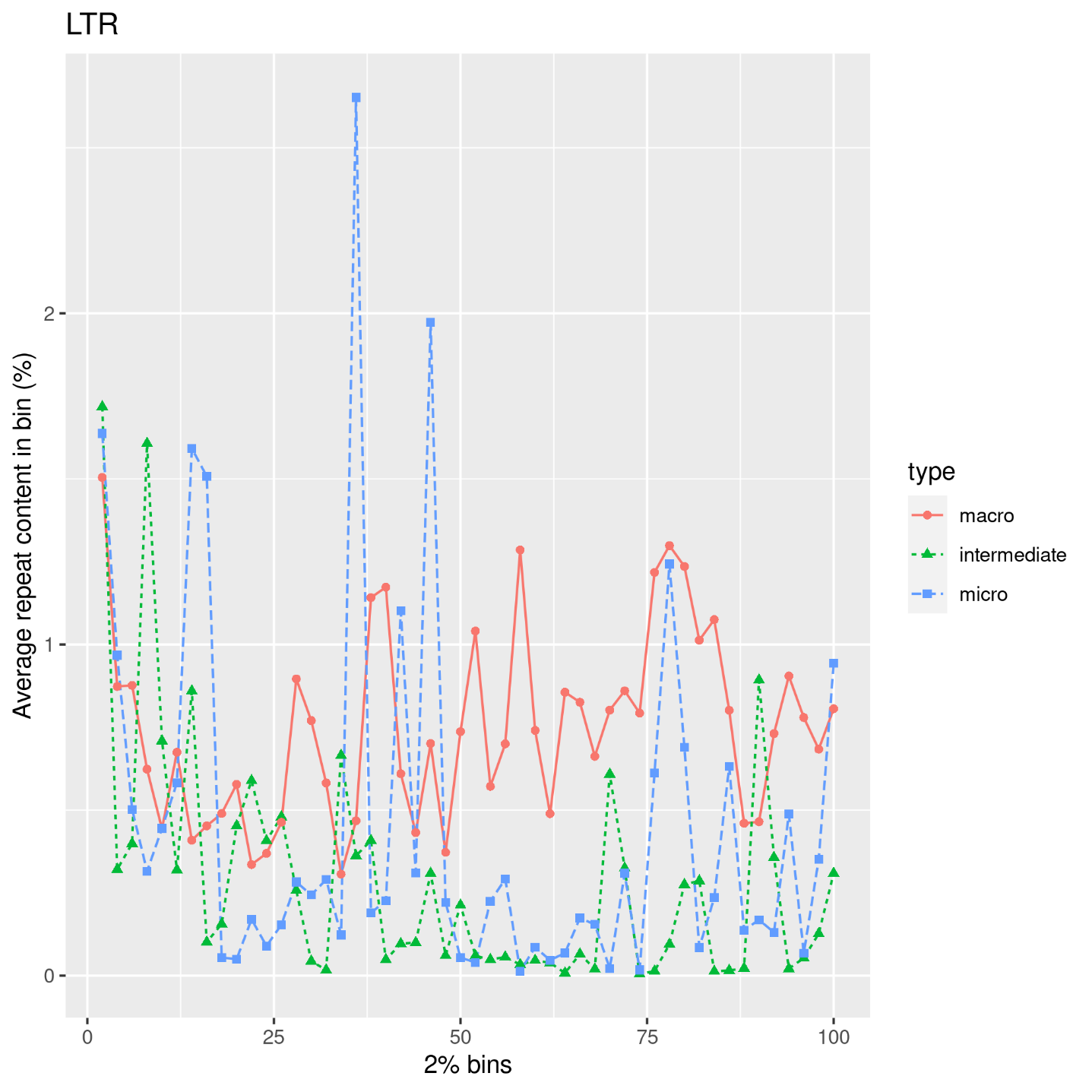


**Figure S12:** Average LTR repeat content along the chromosomes for macro, intermediate and microcromosomes.


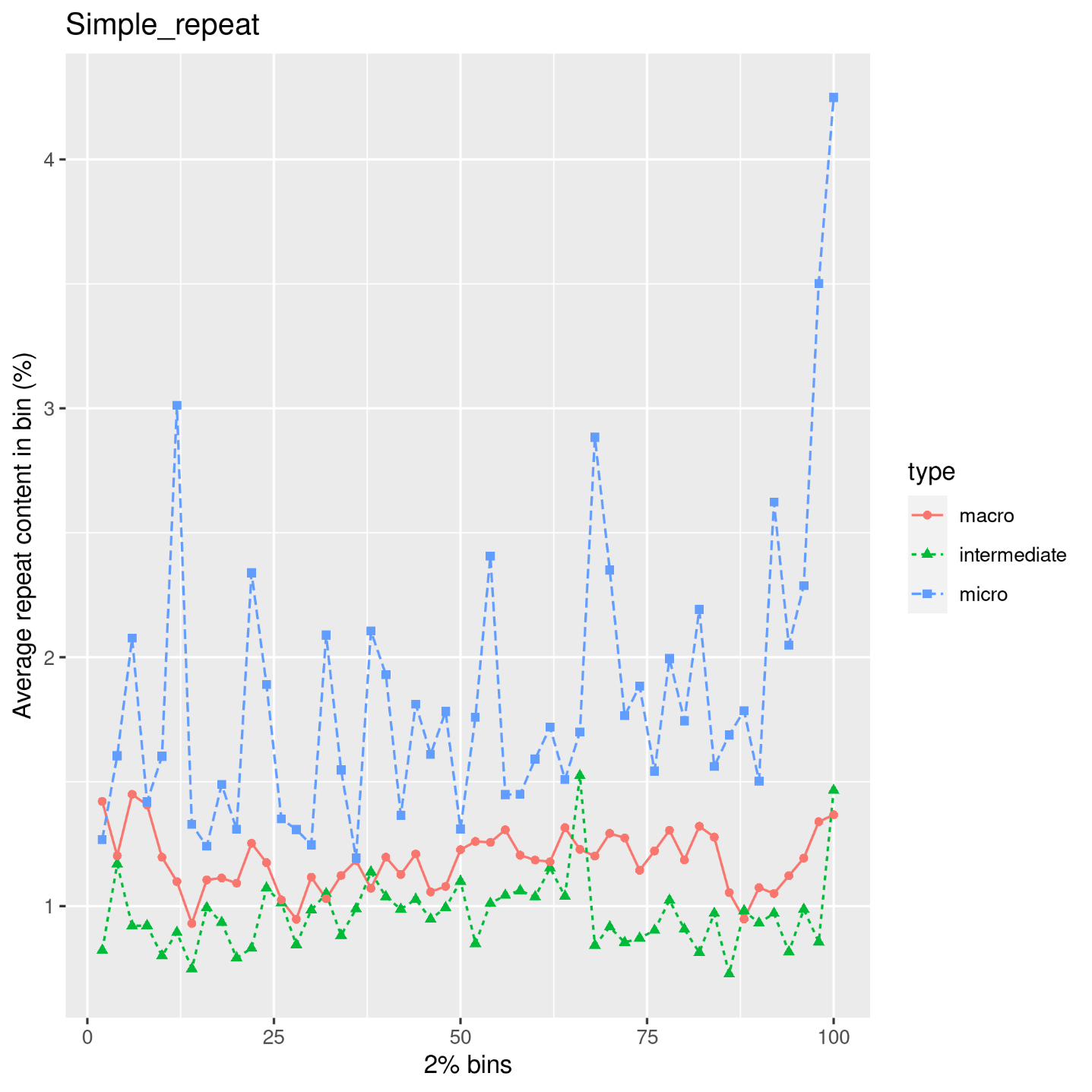


**Figure S13:** Average simple repeat content along the chromosomes for macro, intermediate and microcromosomes.


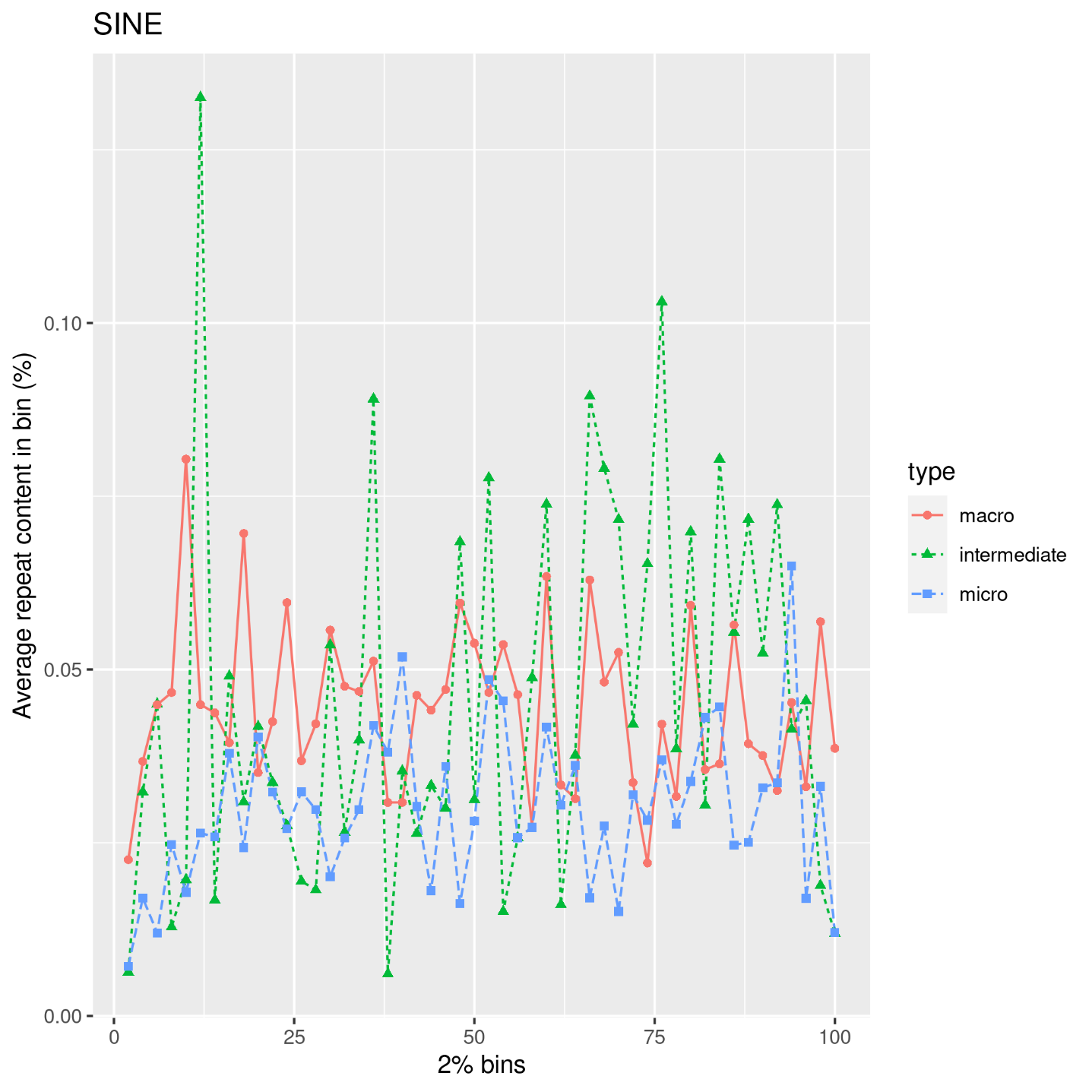


**Figure S14:** Average SINE repeat content along the chromosomes for macro, intermediate and microcromosomes.


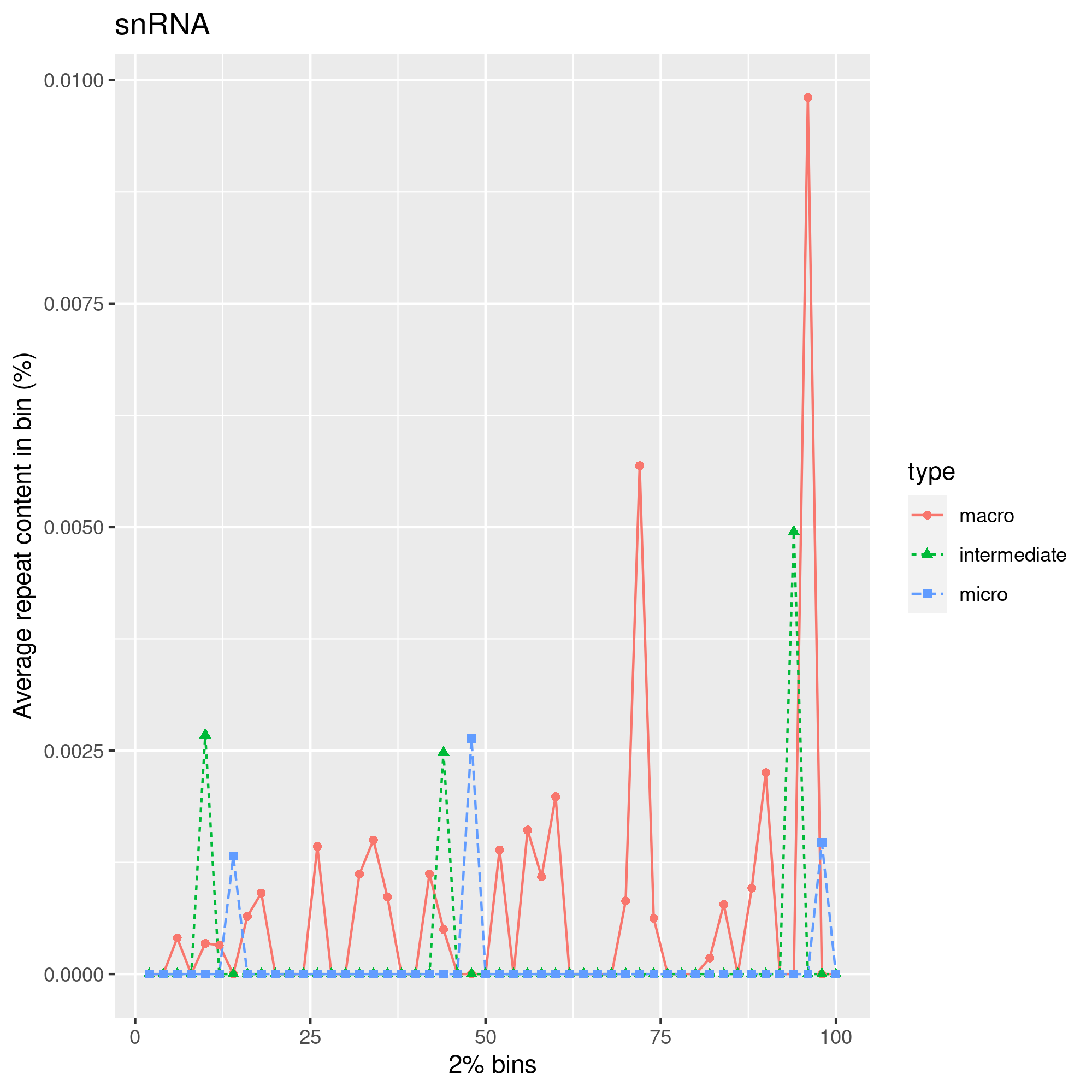


**Figure S15:** Average snRNA repeat content along the chromosomes for macro, intermediate and microcromosomes.


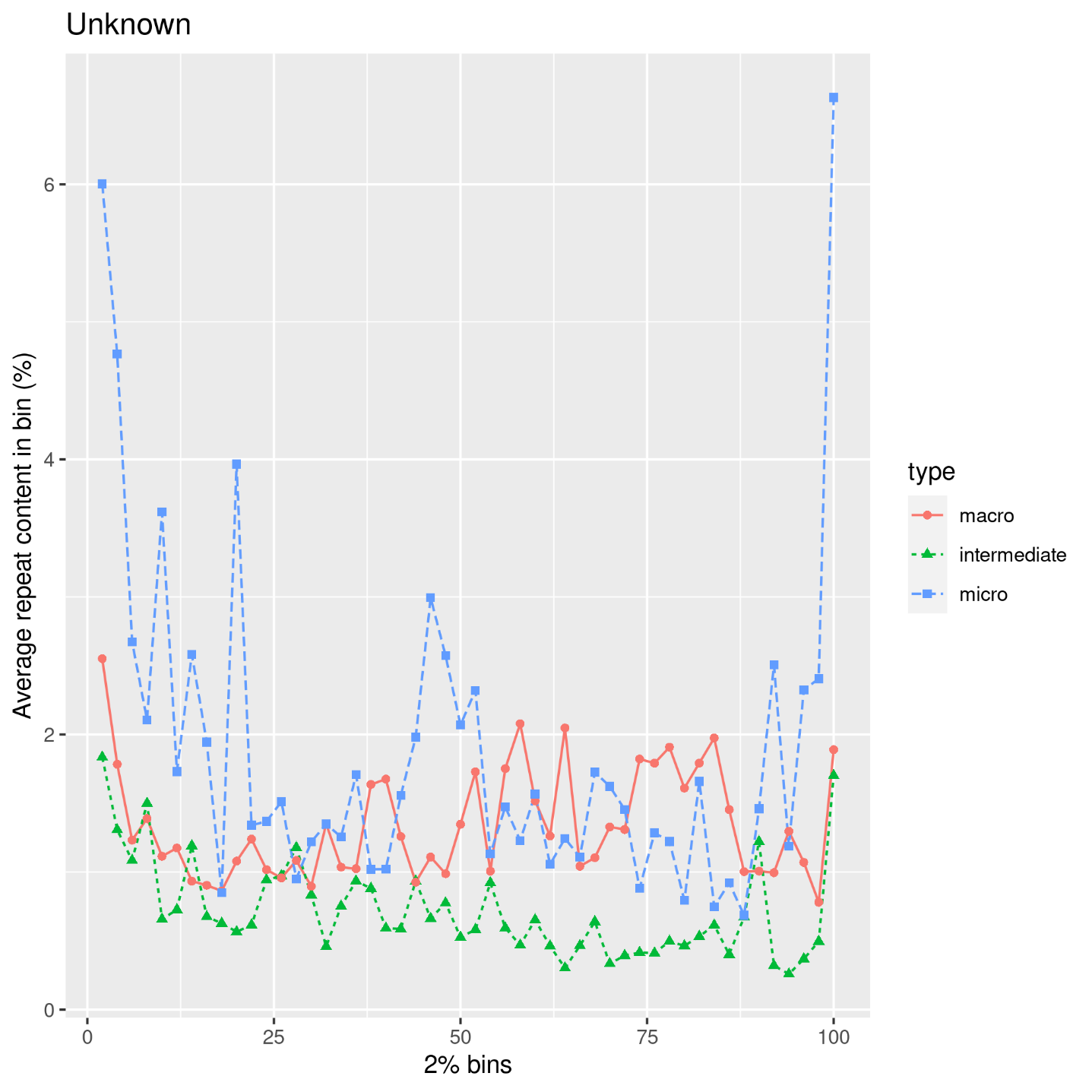


**Figure S16:** Average unknown repeat content along the chromosomes for macro, intermediate and microcromosomes.


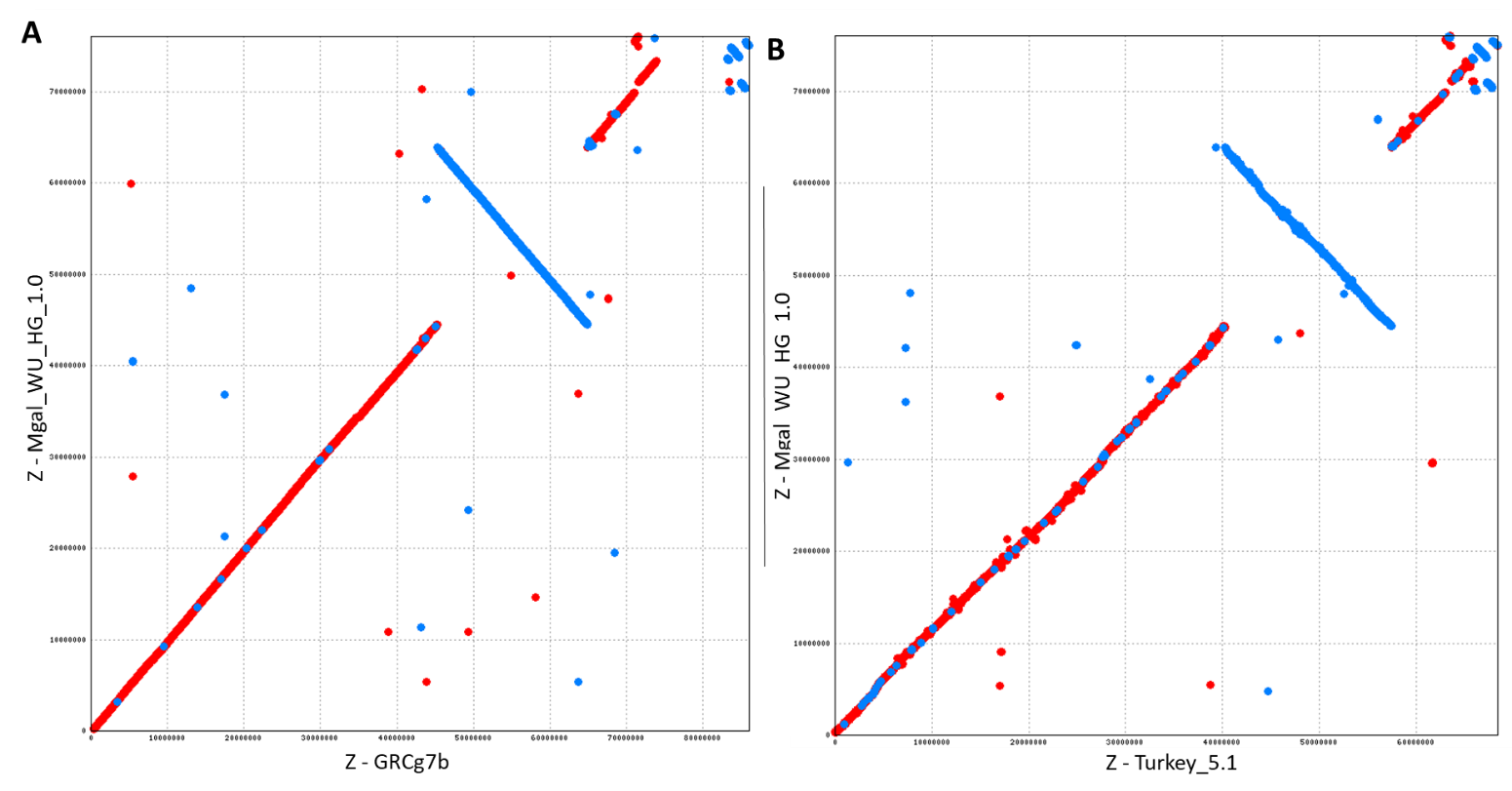


**Figure S17:** Chromosome Z alignment showing inversion with GRCg7b (A) and Turkey5.1 (B).


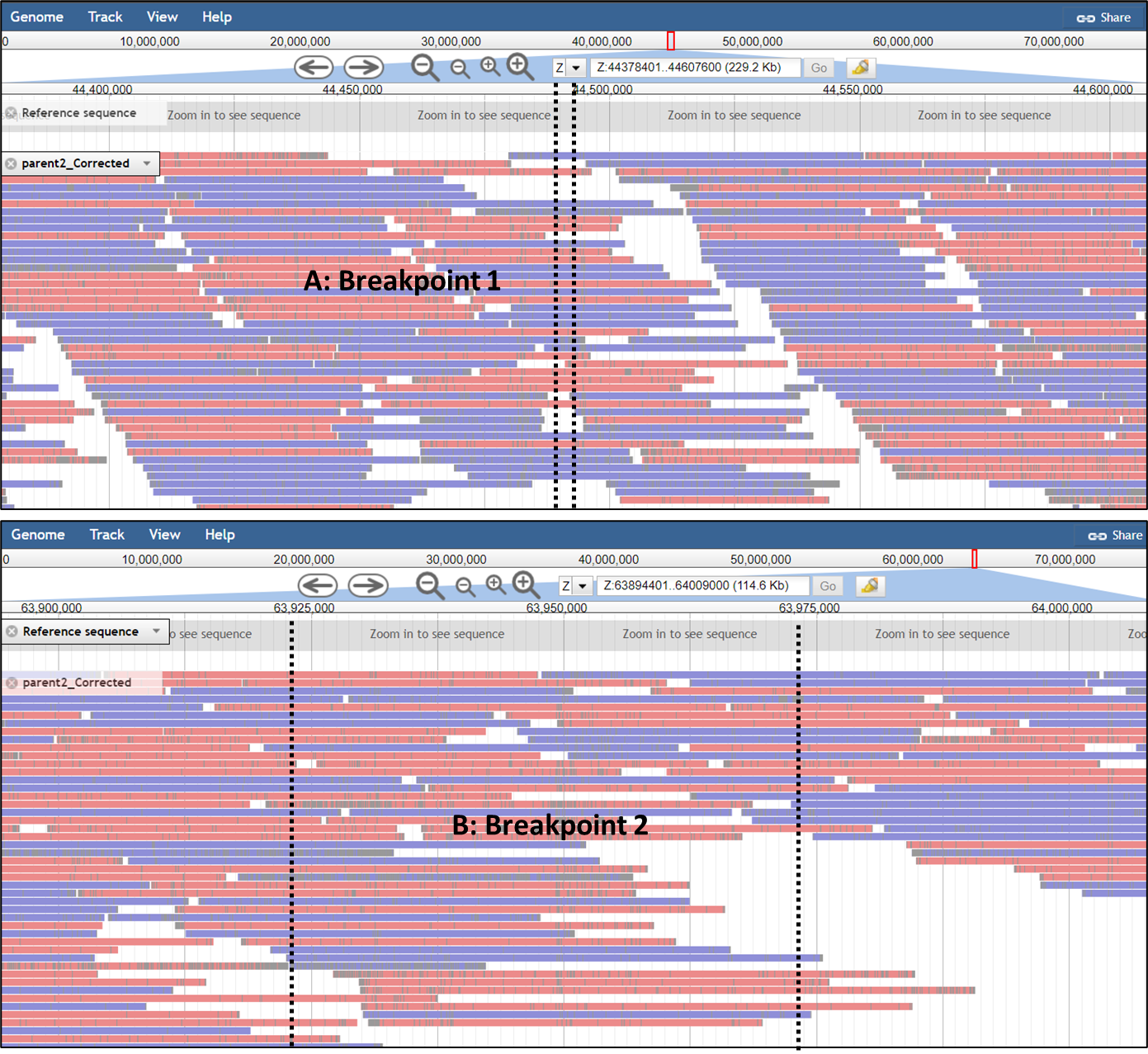


**Figure S18:** Alignment of corrected pacbio reads at the approximate breakpoints of the ~19.4 Mbp inversion on the Z-chromosome.


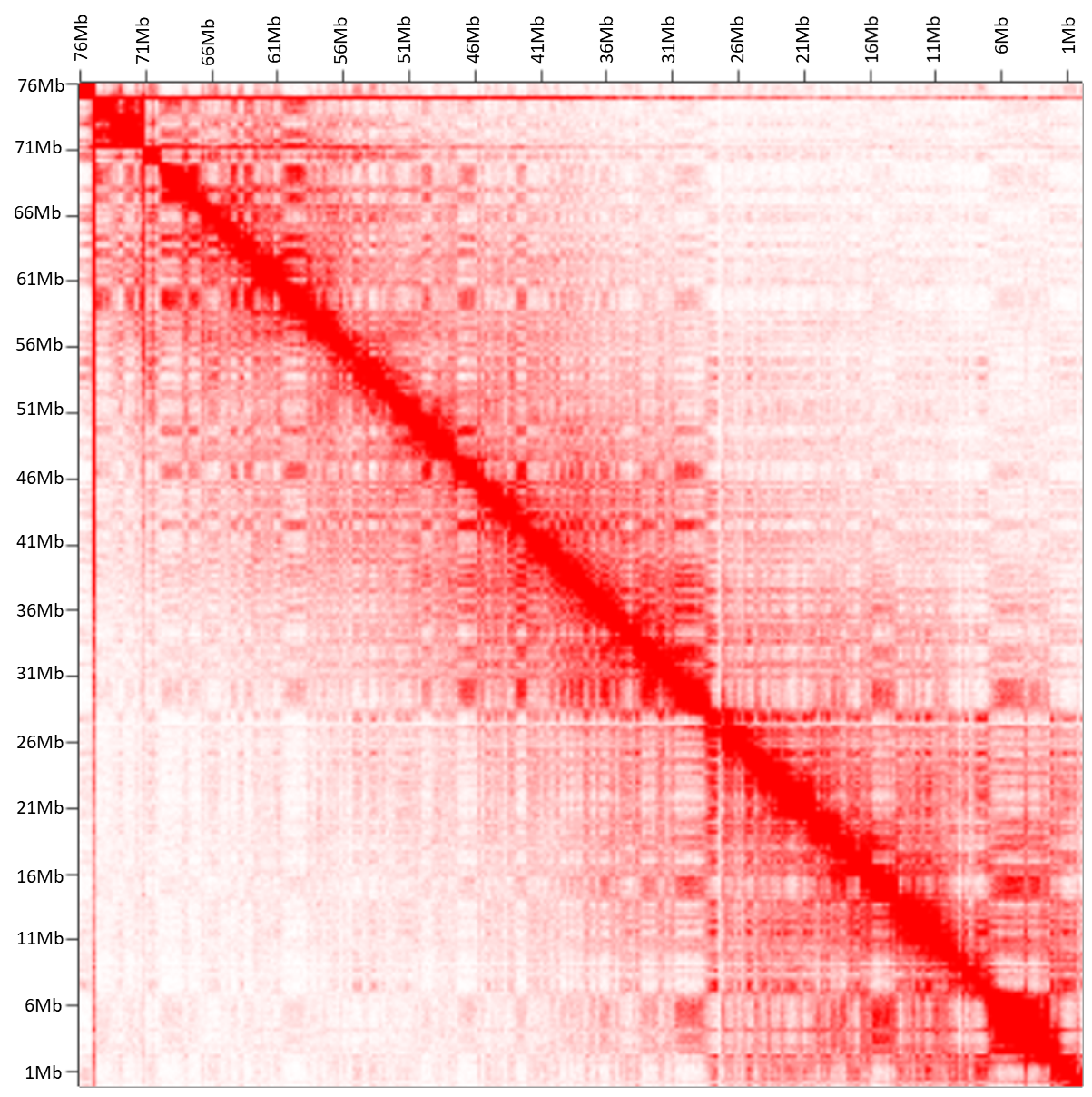


**Figure S19:** HiC contact map of the Z chromosome.


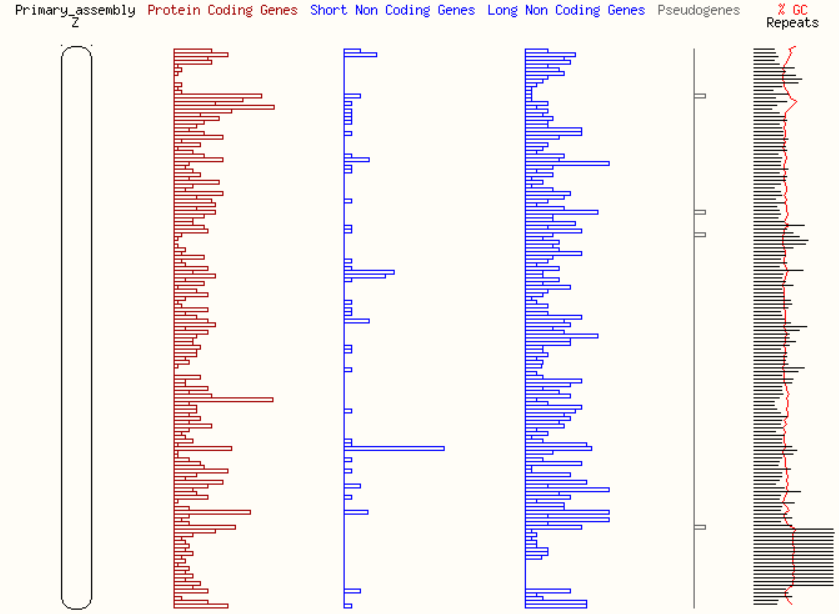


**Figure S20:** Schematic view of Gal7b chromosome Z and representation of several biotypes of genes and genomic features (Ensembl, rapid release 15^th^ June 2022, accessed on 27^th^ June 2022).
