# Supplementary figures and images for: A new haplotype-resolved turkey genome to enable turkey genetics and genomics research
Source: Gigascience. 2023 Jul 21;12:giad051. doi: 10.1093/gigascience/giad051 (PMC10360393; doi:10.1093/gigascience/giad051)

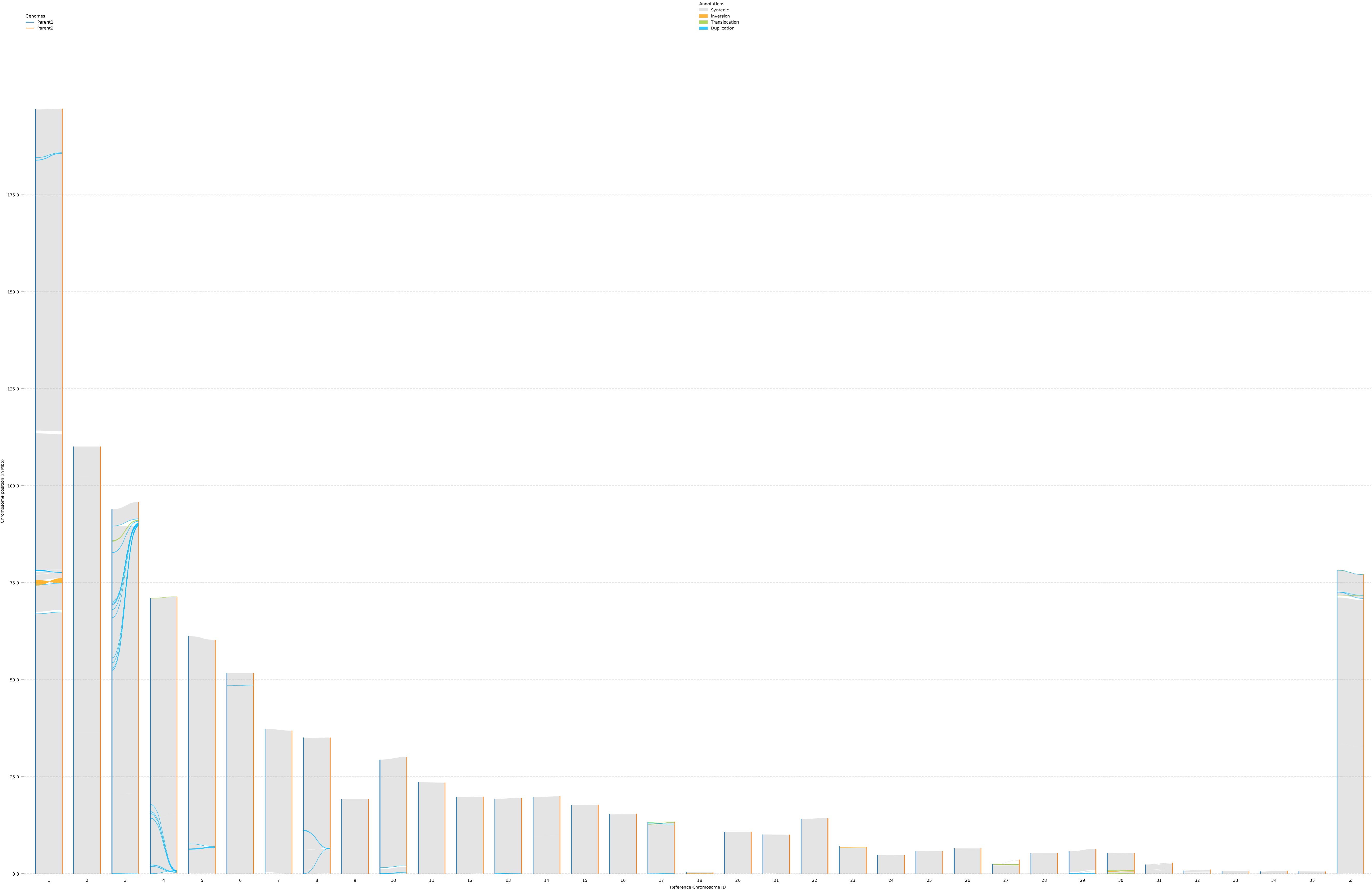

Supplement: giad051_Supplemental_Files [file giad051_supplemental_files.zip › SupplementaryFile4.pdf]
