# Supplementary material for: A new haplotype-resolved turkey genome to enable turkey genetics and genomics research
Source: Gigascience. 2023 Jul 21;12:giad051. doi: 10.1093/gigascience/giad051 (PMC10360393; doi:10.1093/gigascience/giad051)
Supplement: giad051_Response_to_Reviewer_Comments_Original_Submission [file giad051_response_to_reviewer_comments_original_submission.pdf]

Author's Response To Reviewer Comments

Close

Reviewer reports:

Reviewer #1: The turkey has importance for agriculture as it is the second contributor to word poultry meat production. This study completes a chromosome-scale genome assembly with long reads sequencing and use trio-binning approach to generate a haplotype-resolved turkey genome, which give scientific significance to further genetic studies within this species. However, I feel the content within this article need improvement. Some parts were unclear and hard to follow, I list some of them as below. After substantial revisions, I will suggest the publication.

Dear reviewer, thanks for the useful suggestions, we have now addressed your comments as good as possible. We specifically now provide additional metrics to support the quality of our assembly and annotation. For example, we now also include the BUSCO values for the annotation and the QV values of the assemblies. We also now improved our gene family analysis and provided further evidence of the structural variation. We hope that hereby we have sufficiently addressed your comments and concerns.

In abstract,

The sentence "These assemblies cover 35 chromosomes in a single scaffold and show improved genome completeness and continuity" seems weird and hard to understand directly. Please revise it and make it clear.

We agree this sentence was not completely clear, we now updated the sentence to make it more clear and refer to N50 and BUSCO scores to support that. Line X-X

"The three assemblies are of higher quality than the previous draft quality assembly and comparable to the current chicken assemblies (GRCg6a and GRCg7)." Please indicate the parameters used for comparison clearly and how prove them with a higher quality.

Again, we now emphasize on metrics to support our statements.

"Structural variation between the parent haplotypes were identified in genes involved in growth providing new target genes for breeding." The theoretical context of this sentence is not clear, so I suggest more information added to make it clear.

We agree that given we did not do a GWAS, we cannot state that the gene is involved in growth in this line directly. Hence, we chose to put less emphasize on this and rewrite the sentence. We did however include screenshots and additional evidence to support our identied SVs.

Considering no statistic in the conclusion, I suggest the conclusion sentence can be revised as "we contribute a new high-quality turkey genome at chromosome-level, benefiting turkey genetics and other avian genomics research as well as turkey breeding industry."

Thank you for this suggestion, we updated this accordingly.

In the introduction,
"Most of the chromosomes are small microchromosomes, while only a few macrochromosomes are present in the karyotype." Please clearly indicate how many microchormosomes in turkeys and chicken. "most of" is uninformative for readers.

We agree we should be more precise. However, the defeninition of a macro, intermediate or microchromosome is not always clear. Nevertheless, we rephrased this sentence: “The turkey karyotype consists of 7 macrochromosomes (>50 Mb), four intermediate chromosomes (>20 Mb, <40 Mb), and the rest being microchromosomes (<20Mb).”

"and by current standards would be considered of draft quality". What is the current standards? Please indicate it clearly.

We have now emphasized that the genome is of draft quality given the low contig and N50 and lack of third generation long read sequencing data.

"Ongoing efforts in producing high quality assemblies of the microchromosomes in avian genomes have been unsuccessful due to multiple causes" what the multiple causes represent for? Or the features of microchromsomes leads to the unsuccessful assembly as mentioned above?

The main issues are indeed mentioned above in the paragraph (high GC content, repeat content, and lack of linkage group markers). We now mention this at the tail of the paragraph.

"For instance, improved annotation of (non)-coding genes benefits the functional interpretation of genome wide association studies (GWAS), and aids in identifying targets for gene editing", why are non-coding genes (I understand the non-coding genes are referred as regulatory regions, but actually, they are not real genes.) benefits …? Why protein-coding genes (structural genes) can not undertake the roles?

We do not agree that non-coding RNAs are not real genes. Yes, they do not code for a protein, but they are genes often referred to as RNA genes. Yes, protein coding genes are the prime genes to look at when doing finemapping of GWAS results. However, sometimes the causal variant is not within a protein coding gene but is found to be a e.g. long non coding RNA (lncRNA). We therefore also put (non) between parenthesis in the sentence. Together, we are convinced that a better annotation of non-coding genes will benefit studies that do finemapping of causal variants from QTL regions.

"The genome assemblies of turkey (this paper) and chicken, however, are of considerably higher quality compared to other Galliforme species. This provides opportunities for an in-depth comparison between the two most important avian agricultural species." I cannot follow the logic of why the placement of this sentence is here. Obviously, it should be part of discussion after the comparison of turkey genome with other avian genomes.

We agree with your suggestion, and discarded the sentence that was more discussion.

"In this study we use a relatively new technique, the trio-binning approach, to construct high quality haplotype-resolved turkey assemblies." I feel it is necessary to give an explanation of the term "trio-binning approach" as many readers do not understand what is standard for? And the long-reads sequencing technology within it also connect the former theoretical context closely.

We now explain the trio-binning approach in detail at the tail of the paragraph, we feel that we provide sufficient information for the reader to understand the trio-binning approach.

In results,

Have you used other assemblers to complete the genome assembly? Such as flye, or nextdenovo, or mecat2 that may have better performance.

No we did not use other primary assembly tools. One reason is that we already got a good quality primary assembly (contig N50: 26.5 Mb). We did do, however, extensive scaffolding both with HiC (SALSA) and with the pacbio read data (Redundans). We therefore believe the assembly and annotation is of very high (chromosome-level) quality.

Have you ever try 3D-dna for chromosome-scale assembly? which may be better as my experience.

Yes, we have used 3D-dna. However, in our case we had a chromosome-scale assembly after the scaffolding (all chromosomes in 1 scaffold except for a few very small microchromosomes), therefore we did use 3D-dna to further refine our assembly.

The gene annotation should be assessed by BUSCOs.

This is a good suggestion. We have now run BUSCO on our proteins dataset and the other protein datasets as well. Find the results in Table 3. As indicated, the BUSCO scores of our annotation as about similar to the GRCg7b gene annotation and much better compared to Gal6 and the previous turkey genome assembly.

In discussion,

"The quality of the assemblies presented in this study confirms the value of this method in not only providing a quality assembly but also in uncovering structural genomic variation." Please indicate which quality index that reflect your genomic assembly.

We agree that we can be more precise in the quality measures. We therefore now also included the QV values in the supplementary material. Hence, we now stated that we have a chromosome level assembly and added a table to the supplementary file 1 that provides an overview of the assembly and annotation (Table S1).

"Thanks to these recent sequencing technologies, we are able to correct a number of wrongly oriented contigs in Turkey_5.1, a phenomenon often observed in short-read based assemblies." I feel this sentence is not formal in writing.

Short read based assemblies are known to be fragmented and can put contigs in wrong orientation towards each other. We think we describe this accurately in this sentence and we do not see how to improve this.

Reviewer #2: This manuscript by Barros et al. presents a high-quality dipoid turkey genome assembly which shows significant improvement relative to the previous one. This new assembly is timely and will likely be used as the reference turkey genome, but the authors should acknowledge that the W chromosome is absent (because the F1 individual was a male?). This manuscript fits more with "Data Note" than "Research" as I see most results are descriptive and confirmatory.

Dear reviewer 2. We would like to thank you for your useful comments. We believe we have included many additional analysis and support to improve our manuscript and we also believe we now provide sufficient evidence of the quality of our genome assembly and structural variations. We also included in the first paragraph of the discussion that W chromosome is missing because we sequenced a male bird. We do not think this manuscript is more like a Data Note. We performed a thorough analysis of the genome and the two parental haplotypes. First we evaluated the quality of the assembly and annotation, which we believe is very important in a genome paper, especially given the agricultural importance of this species. Moreover, we provide now more details and evidence on the structural variation between the parent haplotypes and we provide a more in depth gene family (using CAFE5) and marco-micro chromosome feature comparisons including statistical tests. We also provide a set of additional evidence supporting the Z-chromosome inversion (HiC map and breakpoint alignments). All in all, we believe that we have much improved the manuscript and we hope we adhere to the comments and suggestions raised by the reviewer.

While the chromosomal assembly is relatively complete, I am concerned whether it still contains assembly errors (because of not being polished by long reads?) which led to fewer genes annotated.

We did actually polish the assembly also with the long reads using wtpoa-cns, which is part of the wtdgb2 assembler. We now included this in the material and methods. In addition we did three round of polishing using the Illumina short reads. We are convinced that our assembly does not contain much errors anymore. This is also reflected by the high genome BUSCO score and high proteome BUSCO score (even higher than Gal6 and comparable to Gal7) given in Table 3.

This assembly metric needs to be taken into accounts if this assembly were to be used as a reference. The authors need to provide the QV value (see the VGP standard), and evaluate indel errors in coding regions.

We ran Mercury to obtain the QV values for the F1 assembly and the two parental assemblies. The QV values are very high. We find a value of 38.7 for the F1 assembly and a value >42 for the parental haplotypes. Also completeness is high >95% for the parental assemblies. The values are given in Supplementary File 1: Table S2.

Some of the results are very brief without showing details or a figure, so difficult for assessment, for instance those SVs affecting genes.

Thank you for this remark, we have elaborated our results of the structural variation and the gene family analysis. We now provide figures showing the structural variation in the supplementary material.

Page 4, "two most important avian agricultural species", I think duck should be the second most important poultry species?

According to citation 1: “Association of Poultry Processors and Poultry Trade in the EU Countries (AVEC)” table 11 and table 13, Turkey is slightly more consumed and bred compared to duck worldwide.

Page 5, I believe the "F1 assembly" refers to the primary assembly or collapsed assembly - please define it more clearly.
We have now indicated that the F1 is the primary assembly.

Page 6, it's unclear how the 36 chromosome models are defined, particularly for small microchromosomes (29-35). According to the karyotype of turkey (2n=80), a few chromosomal models are missing.

Yes, we are still missing a few microchromosomes. We lack linkage data to separate the scaffolds into different chromosomes. We defined the chromosomes 29-35 by size. We also checked homology with the Gal7 genome (Supplementary file 1: Table S1). We now added a table in supplementary material indicating this homology, although there was not always clear homology with Gal7, especially for the very small microchromosomes <1Mb. The last 5 microchromosomes are likely too small to define as sperate chromosomes, and we also lack linkage data to prove these are separate microchromosomes. Hence, the last few “missing” microchromosomes are likely in the assembly as an unplaced scaffold.

Page 6, "This captures the chromosome arms in a single contig" does it apply to all chromosomes? This is unlikely, and data is not shown.

This is a valid point, we changed the sentence accordingly and we have put the number of spanned gaps in the supplementary material (Supplementary File1: Table S1). Most chromosomes have between 0-2 gaps in the scaffold, indicating that yes we do cover most chromosome arms in a single contig (except for chromosome 1, 27 and Z) and for many chromosomes we even span the complete centromere (zero gaps).

Page 6, any idea why the coverage of two parents differs (110X vs. 137X)?
No, we do not know why we have higher coverage for the parent 2 haplotype.

Page 6, "anchored the assemblies to the F1 assembly using RagTag". This suggests and chromosomal assembly of the two haplotypes was not independent, and replied on the F1 assembly. This can potentially lead to missing structural variations between two haplotypes (inversions, translocations).
This is a valid point. Note that we mostly find inversions within single contigs and therefore we do not expect that we miss very large inversions or other structural variation due to the anchoring of the assembly to the F1. We still capture translocations because we do not lose any contigs after anchoring and therefore we will still be able to map all translocations. The anchoring of the contigs is especially useful to provide a clear pairwise comparison of the two parental haplotypes on a chromosome level (as in Supplementary File 4). However, we do see the point that the location of the breakpoints might be incorrect if we anchor the assembly. Nevertheless we think this might only be the case if there are very

large structural differences between the two parental haplotypes, which we did not observe at all, also given that our parental lines are genetically relatively similar.

Page 7, please show more data to support the correct assembly of the chrZ inversion, including Hi-C heatmap, and long-read alignment spanning the inversion breakpoints. Note the Z chromosome inversion has been reported in Zhang et al. 2011 (BMC genomics), which is not cited until in the Discussion.

Thank you for raising this point. We now cite the paper of Zhang et al already in the introduction. In addition, we now provide the chrZ alignment between the old turkey genome and the chicken genome. Also we provide the approximate coordinates of the breakpoint and the HiC contact map of the Z-chromosome and a screenshot of the pacbio read alignment at the approximate breakpoints of the inversion All results support the inversion.

Page 8, it's possible some genes were not annotated because of the presence of indels in coding regions. The genome assembly QV value can be calculated to measure the error frequency (Rhie et al, 2021 Nature).

We now provide the QV value (in Supplementary file 1 Table S3, as described above). The QV values indicate high quality and completeness of all assemblies. Moreover, we ran BUSCO on the gene annotation and we show much higher completeness compared to Gal6 and comparable to the new Gal7 genome build.

Page 8, please provide a statistical result for gene density comparison.
We now perform a statistical test to show significant difference in gene dens
ities. In addition, we provide the gene density per Mb in Table S1. As expected the difference in gene densities between the microchromosomes and the macro-intermediate chromosomes is highly significant as described in the results: “As expected, microchromosomes show higher gene density compared to macro and intermediate chromosomes ($P<0.00001$, Figure 2). The density generally increases with decreasing microchromosome size.”

Page 8, at the bottom, please cite the sources of these bird genomes.
We have now added the citations of the species used in the analysis.

Page 9, "Gene family contractions and expansions". These analyses were a bit crude. " Orthologous groups" is not equivalent to "gene family".
We now provide a more thorough analysis of gene family expansions and contractions using CAFE5. We now also find more gene families that are expended or contracted specifically for the turkey lineage.

Page 10, the phrase "F1 and parent assemblies" is confusing. Both haploid assemblies are derived from the diploid F1. Consider changing to "paternal and maternal genomes".

We changed this according to your suggestion.
Also, as I commented above, both parental chromosomal assemblies are based on the same reference (Mgal_WU_HG_1.0), so the contigs were ordered and placed in the same way.
As mentioned above, the contigs are already very large from the parental haplotypes. So we would be able to identify most of the structural variation, for comparative analysis we thought it would be good anchor the parental haplotypes to the F1 assembly. We still believe we capture the structural variation, despite the anchoring of the scaffolds. Note also that the (structural) variation between the two parent haplotypes is fairly limited, hence, we do not expect extremely large structural differences, and we also have no evidence to support this.
This process could mask the potential non-co-linear segments. For a more appreciated way to independently assemble two chromosome-level assemblies, see the marmoset diploid genome paper (Yang et al., 2021 Nature).

We now provide an overview of the structural variation between the parental haplotypes in the supplementary material.

Page 10, please use a figure to show the SV over the BLB2 gene.

We have included a figure showing the inversion affecting the BLB2 gene.

Page 11, again, please visualize the result on the MAN2B2, GEMIN8, RIMKLB and RALYL cases.

We now provide figures in the supplementary material supporting these structural variation events.

Page 11, "Loss of function variation", I am wondering whether variations mentioned in this part are fixed in the corresponding populations?
Unfortunately we cannot confirm this as we do not have further whole genome sequence data from the populations under study.

Page 11, "Knockouts of this gene lead.." reference is needed.
We now added a reference.

Page 12, "Avian genomes are known to…" references are missing.
We now added a reference.

Page 12, "Distinct genomic landscapes of turkey micro and macrochromosomes", some patterns have been described in the literature, for instance, 10.1111/nyas.13295. Please also perform some statistical analyses to support the claims, not just a figure.
We now performed statistical tests for support the enrichment of specific repeats in macro-and micro chromosomes. In addition, we provide a comprehensive overview of the repeat content per chromosome and per repeat class (Supplementary file 2). We also discuss the scientific paper indicated by the reviewer in the discussion now.

Page 13, "Conserved synteny within the Galliformes clade", please cite 10.1159/000078570 and 10.1007/s00412-018-0685-6

Citations are added

Page 13, "it is evident that especially the Z chromosome" also observed in 10.1038/s41559-019-0850-1
We have now cited this paper.

Page 13, "inversion of around 19 Mbp on the turkey Z" also reported in 10.1186/1471-2164-12-447
We have now cited this paper

Page 14, "tail of the chicken Z chromosome lacks synteny" also reported in 10.1038/nature09172. This means figure S11 does not provide a novel finding.
We agree that this has been described previously, but we think it is important to indicate this because it can improve the interpretation of the alignments. We do, however, now cite this paper in the same sentence.

Page 14, "Combining long reads and genome-wide chromatin interaction data (Hi-C) enables the capture of chromosome arms in a single contig", again, is that correct, chromosome arms in a single contig?
Yes, we have generally between 0 and 3 contigs per chromosome (Supplementary file 1: Table S1). Hence, we are convinced that we capture chromosome arms in a single scaffold for many chromosomes.

Page 18, it's known wtdgb2 assembly tends to contain errors, but it looks the authors did not use long reads for polishing, but only used short reads?
As mentioned above. We did actually polish the assembly also with the long reads using wtpoa-cns, which is part of the wtdgb2 assembler. We now included this in the material and methods. In addition we did three round of polishing using the illumina short reads. We are convinced that our assembly does not contain much errors anymore. This is also reflected by the high genome BUSCO score and high proteome BUSCO score (even higher than e.g. Gal6 and comparable to Gal7).

Page 20, "The corrected reads from TrioCanu were mapped to the Triocanu assembly with Minimap2 v2.17-r941 (Minimap2, RRID:SCR_018550) [45], options -x map-pb", what was is used for?

The option map-bp is used to map back pacbio reads to the assembly. We further used the mapping to validate structural variation in the genome browser JBrowse.

Page 20, "Duplicated sequences were removed." How was this done?
We used seqkit for this, we now added this in the material and methods.

Close
