# Supplementary material for: A new haplotype-resolved turkey genome to enable turkey genetics and genomics research
Source: Gigascience. 2023 Jul 21;12:giad051. doi: 10.1093/gigascience/giad051 (PMC10360393; doi:10.1093/gigascience/giad051)
Supplement: giad051_Response_to_Reviewer_Comments_Revision_1 [file giad051_response_to_reviewer_comments_revision_1.pdf]

Author's Response To Reviewer Comments

Close

Reviewer reports:

Dear Editor,
We would like to thank the reviewers for their helpful comments. We have extensively revised the manuscript especially concerning the centromeric and telomeric regions of the turkey genome. In addition we provide more information and a overview of tandem repeats and other types of structural variation identified between the two parental haplotypes. We hope that this manuscript is now ready to be accepted for publication.

Best Regards,
The authors.

Reviewer #1: The author has carefully amended the work in response to my prior concerns, and the quality of the new version has greatly improved, hence it is suggested that the manuscript be accepted.

We thank reviewer 1 for its constructive review and are happy that he feels the manuscript can be accepted.

Reviewer #2: The manuscript has been improved. After reading the revised manuscript, I have a few more concerns.

Chromosome models. I suggest the chromosome naming should follow chicken's, e.g., chr6 can be chr2a, and the microchromosomes should be named according to chicken homology. I then noticed chr32 and chr35 do not have chicken homology which is very concerning. It is either due to novel chromosomes (very unlikely), or the sequences could be an unlinked contigs. In either scenario, the chromosome models must be clarified. The authors should provide strong evidence to support the chromosome model assembly for chr32 and chr35, e.g. FISH images, Hi-C zoom-in view (Fig. S1 shows the whole genomes where the microchromosome models are not visible), synteny with chicken (note there is a new chicken assembly ASM2420605v1) or zebra finch chromosomes; otherwise, chi32 and chr35 can not be identified as a chromosome.

With the naming of the chromosomes we follow the line of the previous turkey genome assembly. Hence, we do not follow the line of the chicken genome. We believe this is not necessary because chromosome models can be mapped relatively easily nowadays and there are now multiple high quality genomes (also within the Galliformes) that are or will be published. Hence, we do not think it would be necessary to take the chicken as the 'key' reference.
We mapped both chromosomes Chr32 and chr35 to the new chicken genome. Yes, indeed we can only partially map the models to the chicken genome and we find hits with multiple chromosomes using a minimap2 alignment. We do find however an enrichment of hits between chromosome 35 and chromosome 33 (CP100587.1) of the ASM2420605v1 assembly. In addition, for chr32, we find an enrichment of hits with CP100589.2 (chromosome 35) in the new chicken build. We do agree that we can only map the parts of the chromosome models to chicken and therefore it is hard to determine with 100% certainty which chicken microchromosomes are homologous. In the future we will likely release a new build because we are planning to do some ultra-long nanopore sequencing to even resolve the microchromosomes better.

Centromere and telomere. To support complete chromosome assembly, I suggest the authors provide information about the assembly of telomere and centromere sequences, e.g. the presence/absence of TTAGGG at chromosomal ends. Most galliformes microchromosome centromeres are known to contain a 41-bp satellite (10.1139/gen-2022-0012). The authors should investigate whether such centromere

satellites are present in the assembly.

We have extensively investigated the presence of centromeric and telomeric repeats in our assembly discussed under subheading “Telomeres and centromeres” on line 117-142 (and 366-370 in the discussion). Moreover, we provide an overview of the tandem repeat sequences (including telomeric and TM repeats) in supplementary file 1: Figure S2. In short, we show that the tails of the majority of the chromosomes comprises of telomeric or 41bp TM repeat clusters. The 41bp TM repeat was initially discovered by Matzke et al. 1992, 10.1007/BF00352284. Zhang et al 2011 showed that there is a clear trend in turkey towards telocentric chromosomes, only chromosome 1 and the sex chromosomes are likely metacentric. In line with these findings, we observe an enrichment of TM repeats at one end of telocentric chromosomes and telomeric repeats clusters at the other end. This indicates that the centromeric regions of the telocentric chromosomes likely comprise of clusters of the 41bp TM repeat, while the other tail comprises telomeric TTAGGG repeats. Together, we believe we have now extensively investigated telomeric and centromeric regions in the turkey genome and these findings support the completeness (including the telomeric and centromereic region) of the genome assembly.

Data availability. It appears the Hi-C data is not available in NCBI. The raw reads must be provided.

The Hi-C data is uploaded to ENA under accession: ERR11153338
https://www.ebi.ac.uk/ena/browser/view/ERR11153338

In the abstract, there is not such term as "complete scaffold", please remove "complete". Again, I do not see the support for two chromosome models: chr32 and chr35. The chrZ inversion is highlighted in the abstract, but this is not a novel finding - the writing is thus misleading. Instead, the new genome assembly only CONFIRMS this inversion.

Thank you for this suggestion. We have altered the abstract accordingly.

The subtitle "Lineage specific expansion and contraction of protein-coding gene families" is unrelated to the following text.

We agree with this statement point and have changed the subtitle to “Gene family analysis”

"a 1.47 Mbp inversion on chromosome 1" I am wondering if this is the centromere? According to chicken chr1 centromere position, it looks like so.

Yes, this is very close to the predicted centromeric region (1:74.12-74.16 Mb enriched for TTAGGG and TM repeats). This has now been revised and discussed in the main text (Line 242-243).

In the Table 5, the Parent2 has a much large size of gained copy. Please show more details, e.g. chromosomal distribution.

We now include a chromosomal distribution of the CNVs on both parents (Supplementary File 1: Figure S4). Note that, copy gains are regions where parent2 has extra copies, while copy losses are regions where parent2 has less copies. This is now also discussed in more details in the result section, Line 250-251. Note that, duplications, inversions, and translocations between parent1 and parent2 haplotypes are shown in supplementary file 4.

"BLB2", is this gene associated with parent2-specific trait? Similarly, what about TRIM36, GRIA2 and MAN2B2, and LRRC41?

We have now included an extensive discussion about these genes in the discussion section. Line 395-413. As discussed, The LRRC41 gene is of specific interest because it is associated with increased lean body mass in knockout mice.

"The inversion was supported by a normal alignment at the approximate breakpoints (Supplementary File 1: Table S7 - Figure S16) and by the Hi-C contact map". The writing here is unclear. Hi-C data does not

show signal for inversion, instead, it only supports that the assembly is correct.

This has now been revised, Line 339.

Bellott et al 2020 should be Bellott et al 2017.

Corrected

"Centromeres, however, are too long to traverse reliably in most cases". I do not see any analyses on centromeres.

As mentioned before, we have now extensively discussed the centromeres and telomeres and removed this line from the discussion.

PRJEB42643 does not contain Hi-C data.
The Hi-C data is uploaded to ENA under accession: ERR11153338
https://www.ebi.ac.uk/ena/browser/view/ERR11153338

Close
