# Supplementary material for: A new haplotype-resolved turkey genome to enable turkey genetics and genomics research
Source: Gigascience. 2023 Jul 21;12:giad051. doi: 10.1093/gigascience/giad051 (PMC10360393; doi:10.1093/gigascience/giad051)
Supplement: giad051_Reviewer_1_Report_Original_Submission — Yunyun Lv -- 8/22/2022 Reviewed [file giad051_reviewer_1_report_original_submission.pdf]

Reviewer Report

Title: A new haplotype-resolved turkey genome to enable turkey genetics and genomics research

Version: Original Submission **Date:** 8/22/2022

Reviewer name: Yunyun Lv

Reviewer Comments to Author:

The turkey has importance for agriculture as it is the second contributor to word poultry meat production. This study completes a chromosome-scale genome assembly with long reads sequencing and use trio-binning approach to generate a haplotype-resolved turkey genome, which give scientific significance to further genetic studies within this species. However, I feel the content within this article need improvement. Some parts were unclear and hard to follow, I list some of them as below. After substantial revisions, I will suggest the publication.
In abstract,
The sentence "These assemblies cover 35 chromosomes in a single scaffold and show improved genome completeness and continuity" seems weird and hard to understand directly. Please revise it and make it clear.
"The three assemblies are of higher quality than the previous draft quality assembly and comparable to the current chicken assemblies (GRCg6a and GRCg7)." Please indicate the parameters used for comparison clearly and how prove them with a higher quality.
"Structural variation between the parent haplotypes were identified in genes involved in growth providing new target genes for breeding." The theoretical context of this sentence is not clear, so I suggest more information added to make it clear.
Considering no statistic in the conclusion, I suggest the conclusion sentence can be revised as "we contribute a new high-quality turkey genome at chromosome-level, benefiting turkey genetics and other avian genomics research as well as turkey breeding industry."
In the introduction,
"Most of the chromosomes are small microchromosomes, while only a few macrochromosomes are present in the karyotype." Please clearly indicate how many microchormosomes in turkeys and chicken. "most of" is uninformative for readers.
"and by current standards would be considered of draft quality". What is the current standards? Please indicate it clearly.
"Ongoing efforts in producing high quality assemblies of the microchromosomes in avian genomes have been unsuccessful due to multiple causes" what the multiple causes represent for? Or the features of microchromsomes leads to the unsuccessful assembly as mentioned above?
"For instance, improved annotation of (non)-coding genes benefits the functional interpretation of genome wide association studies (GWAS), and aids in identifying targets for gene editing", why are non-coding genes (I understand the non-coding genes are referred as regulatory regions, but actually, they are not real genes.) benefits …? Why protein-coding genes (structural genes) can not undertake the roles?

"The genome assemblies of turkey (this paper) and chicken, however, are of considerably higher quality compared to other Galliforme species. This provides opportunities for an in-depth comparison between the two most important avian agricultural species." I cannot follow the logic of why the placement of this sentence is here. Obviously, it should be part of discussion after the comparison of turkey genome with other avian genomes.
"In this study we use a relatively new technique, the trio-binning approach, to construct high quality haplotype-resolved turkey assemblies." I feel it is necessary to give an explanation of the term "trio-binning approach" as many readers do not understand what is standard for? And the long-reads sequencing technology within it also connect the former theoretical context closely.
In results,
Have you used other assemblers to complete the genome assembly? Such as flye, or nextdenovo, or mecat2 that may have better performance.
Have you ever try 3D-dna for chromosome-scale assembly? which may be better as my experience.
The gene annotation should be assessed by BUSCOs.
In discussion,
"The quality of the assemblies presented in this study confirms the value of this method in not only providing a quality assembly but also in uncovering structural genomic variation." Please indicate which quality index that reflect your genomic assembly.
"Thanks to these recent sequencing technologies, we are able to correct a number of wrongly oriented contigs in Turkey_5.1, a phenomenon often observed in short-read based assemblies." I feel this sentence is not formal in writing.

Methods

Are the methods appropriate to the aims of the study, are they well described, and are necessary controls included? Choose an item.

Conclusions

Are the conclusions adequately supported by the data shown? Choose an item.

Reporting Standards

Does the manuscript adhere to the journal's guidelines on minimum standards of reporting? Choose an item.

Choose an item.

Statistics

Are you able to assess all statistics in the manuscript, including the appropriateness of statistical tests used? Choose an item.

Quality of Written English

Please indicate the quality of language in the manuscript: Choose an item.

Declaration of Competing Interests

Please complete a declaration of competing interests, considering the following questions:

- Have you in the past five years received reimbursements, fees, funding, or salary from an organisation that may in any way gain or lose financially from the publication of this manuscript, either now or in the future?
- Do you hold any stocks or shares in an organisation that may in any way gain or lose financially from the publication of this manuscript, either now or in the future?
- Do you hold or are you currently applying for any patents relating to the content of the manuscript?
- Have you received reimbursements, fees, funding, or salary from an organization that holds or has applied for patents relating to the content of the manuscript?
- Do you have any other financial competing interests?
- Do you have any non-financial competing interests in relation to this paper?

If you can answer no to all of the above, write 'I declare that I have no competing interests' below. If your reply is yes to any, please give details below.

I declare that I have no competing interests

I agree to the open peer review policy of the journal. I understand that my name will be included on my report to the authors and, if the manuscript is accepted for publication, my named report including any attachments I upload will be posted on the website along with the authors' responses. I agree for my report to be made available under an Open Access Creative Commons CC-BY license (http://creativecommons.org/licenses/by/4.0/). I understand that any comments which I do not wish to be included in my named report can be included as confidential comments to the editors, which will not be published.

Choose an item.

To further support our reviewers, we have joined with Publons, where you can gain additional credit to further highlight your hard work (see: https://publons.com/journal/530/gigascience). On publication of this paper, your review will be automatically added to Publons, you can then choose whether or not to claim your Publons credit. I understand this statement.

Yes Choose an item.
