# Supplementary material for: A new haplotype-resolved turkey genome to enable turkey genetics and genomics research
Source: Gigascience. 2023 Jul 21;12:giad051. doi: 10.1093/gigascience/giad051 (PMC10360393; doi:10.1093/gigascience/giad051)
Supplement: giad051_Reviewer_2_Report_Original_Submission — Luohao Xu -- 9/8/2022 Reviewed [file giad051_reviewer_2_report_original_submission.pdf]

Reviewer Report

Title: A new haplotype-resolved turkey genome to enable turkey genetics and genomics research

Version: Original Submission **Date:** 9/8/2022

Reviewer name: Luohao Xu

Reviewer Comments to Author:

This manuscript by Barros et al. presents a high-quality dipoid turkey genome assembly which shows significant improvement relative to the previous one. This new assembly is timely and will likely be used as the reference turkey genome, but the authors should acknowledge that the W chromosome is absent (because the F1 individual was a male?). This manuscript fits more with "Data Note" than "Research" as I see most results are descriptive and confirmatory. While the chromosomal assembly is relatively complete, I am concerned whether it still contains assembly errors (because of not being polished by long reads?) which led to fewer genes annotated. This assembly metric needs to be taken into accounts if this assembly were to be used as a reference. The authors need to provide the QV value (see the VGP standard), and evaluate indel errors in coding regions.
Some of the results are very brief without showing details or a figure, so difficult for assessment, for instance those SVs affecting genes.
Page 4, "two most important avian agricultural species", I think duck should be the second most important poultry species?
Page 5, I believe the "F1 assembly" refers to the primary assembly or collapsed assembly - please define it more clearly.
Page 6, it's unclear how the 36 chromosome models are defined, particularly for small microchromosomes (29-35). According to the karyotype of turkey (2n=80), a few chromosomal models are missing.
Page 6, "This captures the chromosome arms in a single contig" does it apply to all chromosomes? This is unlikely, and data is not shown.
Page 6, any idea why the coverage of two parents differs (110X vs. 137X)?
Page 6, "anchored the assemblies to the F1 assembly using RagTag". This suggests and chromosomal assembly of the two haplotypes was not independent, and replied on the F1 assembly. This can potentially lead to missing structural variations between two haplotypes (inversions, translocations).
Page 7, please show more data to support the correct assembly of the chrZ inversion, including Hi-C heatmap, and long-read alignment spanning the inversion breakpoints. Note the Z chromosome inversion has been reported in Zhang et al. 2011 (BMC genomics), which is not cited until in the Discussion.
Page 8, it's possible some genes were not annotated because of the presence of indels in coding regions. The genome assembly QV value can be calculated to measure the error frequency (Rhie et al, 2021 Nature).
Page 8, please provide a statistical result for gene density comparison.
Page 8, at the bottom, please cite the sources of these bird genomes.

Page 9, "Gene family contractions and expansions". These analyses were a bit crude. " Orthologous groups" is not equivalent to "gene family".
Page 10, the phrase "F1 and parent assemblies" is confusing. Both haploid assemblies are derived from the diploid F1. Consider changing to "paternal and maternal genomes". Also, as I commented above, both parental chromosomal assemblies are based on the same reference (Mgal_WU_HG_1.0), so the contigs were ordered and placed in the same way. This process could mask the potential non-co-linear segments. For a more appreciated way to independently assemble two chromosome-level assemblies, see the marmoset diploid genome paper (Yang et al., 2021 Nature).
Page 10, please use a figure to show the SV over the BLB2 gene.
Page 11, again, please visualize the result on the MAN2B2, GEMIN8, RIMKLB and RALYL cases.
Page 11, "Loss of function variation", I am wondering whether variations mentioned in this part are fixed in the corresponding populations?
Page 11, "Knockouts of this gene lead.." reference is needed.
Page 12, "Avian genomes are known to…" references are missing.
Page 12, "Distinct genomic landscapes of turkey micro and macrochromosomes", some patterns have been described in the literature, for instance, 10.1111/nyas.13295. Please also perform some statistical analyses to support the claims, not just a figure.
Page 13, "Conserved synteny within the Galliformes clade", please cite 10.1159/000078570 and 10.1007/s00412-018-0685-6
Page 13, "it is evident that especially the Z chromosome" also observed in 10.1038/s41559-019-0850-1
Page 13, "inversion of around 19 Mbp on the turkey Z" also reported in 10.1186/1471-2164-12-447
Page 14, "tail of the chicken Z chromosome lacks synteny" also reported in 10.1038/nature09172. This means figure S11 does not provide a novel finding.
Page 14, "Combining long reads and genome-wide chromatin interaction data (Hi-C) enables the capture of chromosome arms in a single contig", again, is that correct, chromosome arms in a single contig?
Page 18, it's known wtdgb2 assembly tends to contain errors, but it looks the authors did not use long reads for polishing, but only used short reads?
Page 20, "The corrected reads from TrioCanu were mapped to the Triocanu assembly with Minimap2 v2.17-r941 (Minimap2, RRID:SCR_018550) [45], options -x map-pb", what was is used for?
Page 20, "Duplicated sequences were removed." How was this done?

Methods

Are the methods appropriate to the aims of the study, are they well described, and are necessary controls included? Choose an item.

Conclusions

Are the conclusions adequately supported by the data shown? Choose an item.

Reporting Standards

Does the manuscript adhere to the journal's guidelines on minimum standards of reporting? Choose an item.

Choose an item.

Statistics

Are you able to assess all statistics in the manuscript, including the appropriateness of statistical tests used? Choose an item.

Quality of Written English

Please indicate the quality of language in the manuscript: Choose an item.

Declaration of Competing Interests

Please complete a declaration of competing interests, considering the following questions:

- Have you in the past five years received reimbursements, fees, funding, or salary from an organisation that may in any way gain or lose financially from the publication of this manuscript, either now or in the future?
- Do you hold any stocks or shares in an organisation that may in any way gain or lose financially from the publication of this manuscript, either now or in the future?
- Do you hold or are you currently applying for any patents relating to the content of the manuscript?
- Have you received reimbursements, fees, funding, or salary from an organization that holds or has applied for patents relating to the content of the manuscript?
- Do you have any other financial competing interests?
- Do you have any non-financial competing interests in relation to this paper?

If you can answer no to all of the above, write 'I declare that I have no competing interests' below. If your reply is yes to any, please give details below.

I declare that I have no competing interests

I agree to the open peer review policy of the journal. I understand that my name will be included on my report to the authors and, if the manuscript is accepted for publication, my named report including any attachments I upload will be posted on the website along with the authors' responses. I agree for my report to be made available under an Open Access Creative Commons CC-BY license (http://creativecommons.org/licenses/by/4.0/). I understand that any comments which I do not wish to be included in my named report can be included as confidential comments to the editors, which will not be published.

Choose an item.

To further support our reviewers, we have joined with Publons, where you can gain additional credit to further highlight your hard work (see: https://publons.com/journal/530/gigascience). On publication of this paper, your review will be automatically added to Publons, you can then choose whether or not to claim your Publons credit. I understand this statement.

Yes Choose an item.
