# Supplementary material for: A new haplotype-resolved turkey genome to enable turkey genetics and genomics research
Source: Gigascience. 2023 Jul 21;12:giad051. doi: 10.1093/gigascience/giad051 (PMC10360393; doi:10.1093/gigascience/giad051)
Supplement: giad051_Reviewer_2_Report_Revision_1 — Luohao Xu -- 12/24/2022 Reviewed [file giad051_reviewer_2_report_revision_1.pdf]

Reviewer Report

Title: A new haplotype-resolved turkey genome to enable turkey genetics and genomics research

Version: Revision 1 **Date:** 12/24/2022

Reviewer name: Luohao Xu

Reviewer Comments to Author:

The manuscript has been improved. After reading the revised manuscript, I have a few more concerns.
Chromosome models. I suggest the chromosome naming should follow chicken's, e.g., chr6 can be chr2a, and the microchromosomes should be named according to chicken homology. I then noticed chr32 and chr35 do not have chicken homology which is very concerning. It is either due to novel chromosomes (very unlikely), or the sequences could be an unlinked contigs. In either scenario, the chromosome models must be clarified. The authors should provide strong evidence to support the chromosome model assembly for chr32 and chr35, e.g. FISH images, Hi-C zoom-in view (Fig. S1 shows the whole genomes where the microchromosome models are not visible), synteny with chicken (note there is a new chicken assembly ASM2420605v1) or zebra finch chromosomes; otherwise, chi32 and chr35 can not be identified as a chromosome.
Centromere and telomere. To support complete chromosome assembly, I suggest the authors provide information about the assembly of telomere and centromere sequences, e.g. the presence/absence of TTAGGG at chromosomal ends. Most galliformes microchromosome centromeres are known to contain a 41-bp satellite (10.1139/gen-2022-0012). The authors should investigate whether such centromere satellites are present in the assembly.
Data availability. It appears the Hi-C data is not available in NCBI. The raw reads must be provided.
In the abstract, there is not such term as "complete scaffold", please remove "complete". Again, I do not see the support for two chromosome models: chr32 and chr35. The chrZ inversion is highlighted in the abstract, but this is not a novel finding - the writing is thus misleading. Instead, the new genome assembly only CONFIRMS this inversion.
The subtitle "Lineage specific expansion and contraction of protein-coding gene families" is unrelated to the following text.
"a 1.47 Mbp inversion on chromosome 1" I am wondering if this is the centromere? According to chicken chr1 centromere position, it looks like so.
In the Table 5, the Parent2 has a much large size of gained copy. Please show more details, e.g. chromosomal distribution
"BLB2", is this gene associated with parent2-specific trait? Similarly, what about TRIM36, GRIA2 and MAN2B2, and LRRC41?
"The inversion was supported by a normal alignment at the approximate breakpoints (Supplementary File 1: Table S7 - Figure S16) and by the HiC contact map". The writing here is unclear. Hi-c data does not show signal for inversion, instead, it only supports that the assembly is correct.
Bellott et al 2020 should be Bellott et al 2017.
"Centromeres, however, are too long to traverse reliably in most cases". I do not see any analyses on

centromeres.
PRJEB42643 does not contain Hi-C data.

Methods

Are the methods appropriate to the aims of the study, are they well described, and are necessary controls included? Choose an item.

Conclusions

Are the conclusions adequately supported by the data shown? Choose an item.

Reporting Standards

Does the manuscript adhere to the journal's guidelines on minimum standards of reporting? Choose an item.

Choose an item.

Statistics

Are you able to assess all statistics in the manuscript, including the appropriateness of statistical tests used? Choose an item.

Quality of Written English

Please indicate the quality of language in the manuscript: Choose an item.

Declaration of Competing Interests

Please complete a declaration of competing interests, considering the following questions:

- Have you in the past five years received reimbursements, fees, funding, or salary from an organisation that may in any way gain or lose financially from the publication of this manuscript, either now or in the future?
- Do you hold any stocks or shares in an organisation that may in any way gain or lose financially from the publication of this manuscript, either now or in the future?
- Do you hold or are you currently applying for any patents relating to the content of the manuscript?
- Have you received reimbursements, fees, funding, or salary from an organization that holds or has applied for patents relating to the content of the manuscript?
- Do you have any other financial competing interests?
- Do you have any non-financial competing interests in relation to this paper?

If you can answer no to all of the above, write 'I declare that I have no competing interests' below. If your reply is yes to any, please give details below.

I declare that I have no competing interests

I agree to the open peer review policy of the journal. I understand that my name will be included on my report to the authors and, if the manuscript is accepted for publication, my named report including any attachments I upload will be posted on the website along with the authors' responses. I agree for my report to be made available under an Open Access Creative Commons CC-BY license (http://creativecommons.org/licenses/by/4.0/). I understand that any comments which I do not wish to be included in my named report can be included as confidential comments to the editors, which will not be published.

Choose an item.

To further support our reviewers, we have joined with Publons, where you can gain additional credit to further highlight your hard work (see: https://publons.com/journal/530/gigascience). On publication of this paper, your review will be automatically added to Publons, you can then choose whether or not to claim your Publons credit. I understand this statement.

Yes Choose an item.
